# C1R, CCL2, and TNFRSF1A Genes in Coronavirus Disease-COVID-19 Pathway Serve as Novel Molecular Biomarkers of GBM Prognosis and Immune Infiltration

**DOI:** 10.1155/2022/8602068

**Published:** 2022-06-18

**Authors:** Xianggang Wang, Guohua Yang, Qingqing Wang, Yilong Zhao, Kaixin Ding, Can Ji, Zongyuan Shi, Huaying Li, Ying Li, Shujing Li

**Affiliations:** ^1^School of Life Science, Bengbu Medical College, 233030 Bengbu City, Anhui Province, China; ^2^Department of Medical Genetics, School of Basic Medical Science, Demonstration Center for Experimental Basic Medicine Education, Wuhan University, 430071 Wuhan City, Hubei Province, China

## Abstract

Glioblastoma multiforme (GBM) is a prevalent intracranial brain tumor associated with a high rate of recurrence and treatment difficulty. The prediction of novel molecular biomarkers through bioinformatics analysis may provide new clues into early detection and eventual treatment of GBM. Here, we used data from the GTEx and TCGA databases to identify 1923 differentially expressed genes (DEGs). GO and KEGG analyses indicated that DEGs were significantly enriched in immune response and coronavirus disease-COVID-19 pathways. Survival analyses revealed a significant correlation between high expression of C1R, CCL2, and TNFRSF1A in the coronavirus disease-COVID-19 pathway and the poor survival in GBM patients. Cell experiments indicated that the mRNA expression levels of C1R, CCL2, and TNFRSF1A in GBM cells were very high. Immune infiltration analysis revealed a significant difference in the proportion of immune cells in tumor and normal tissue, and the expression levels of C1R, CCL2, and TNFRSF1A were associated with immune cell infiltration of GBM. Additionally, the protein-protein interaction networks of C1R, CCL2, and TNFRSF1A involved a total of 65 nodes and 615 edges. These results suggest that C1R, CCL2, and TNFRSF1A may be used as molecular biomarkers of prognosis and immune infiltration in GBM patients in the future.

## 1. Introduction

Glioblastoma multiforme is a prevalent intracranial brain tumor, accounting for about 40%-50% of all intracranial tumors. Not only is GBM common, but it is a cancer with all sorts of frightening features and intimidating labels. First of all, GBM patients have a higher mortality rate. There are 40,000 to 80,000 new cases of GBM in China every year, with up to 30,000 deaths per year. In particular, there has been an increase in cancer patients under the age of 34, whose five-year mortality rate is the third-highest among all cancers, after pancreatic and lung cancer. Secondly, the recurrence rate of GBM is very high. Since glioblastoma is an invasive growth and invades mainly around neurons or along white matter fibers with ill-defined borders, surgery is generally difficult to remove completely. However, unresected glioblastoma is particularly prone to relapse, and relapse is often accompanied by biological malignant progression, from low-grade GBM to high-grade GBM. Third, GBM surgery is complicated. Because glioblastoma is located in the most important and complex brain of the human body, it may lead to disability or death of the patient if it is accidentally damaged in the important tissues in the brain during surgery. The difficulty and risk of surgery are very high. Glioblastoma grows rapidly, with 70% to 80% of patients lasting 3-6 months and only 10% lasting more than 1 year [1, 2]. Generally, patients with glioblastoma after timely surgery can hardly live for more than two years.

With the rapid development of supercomputer technology and the continuous improvement of modern high-throughput sequencing technology, more and more large-scale gene transcriptomics and related clinical databases are freely available, and it has become an increasingly vital and effective method to explore the pathological mechanisms of diseases based on bioinformatics theory. The prediction of novel molecular biomarkers through bioinformatics analysis can provide new insights into early diagnosis, survival prediction, and eventual treatment. At present, new molecular biomarkers of liver hepatocellular carcinoma, breast cancer, lung carcinoma, and adrenocortical carcinoma have been successfully predicted using large-scale clinical databases and bioinformatics tools [3–7]. Compared with other pathological types of glioblastoma multiforme, the majority of GBM patients died from tumor recurrence, with a 5-year survival rate of less than 3%, resulting in insufficient data of patient samples in the TCGA database. Therefore, bioinformatics analysis of GBM patients has rarely been reported.

In this study, we aim to integrate information from the GTEx database and the TCGA database to identify DEGs through full-scale bioinformatics analysis. This may help to find the underlying molecular mechanisms of GBM development and may serve as biomarkers and molecular targets for the diagnosis and prognosis of GBM patients in the immediate future.

## 2. Results

### 2.1. Identification and Screening of 1923 DEGs

**Figure 1:**
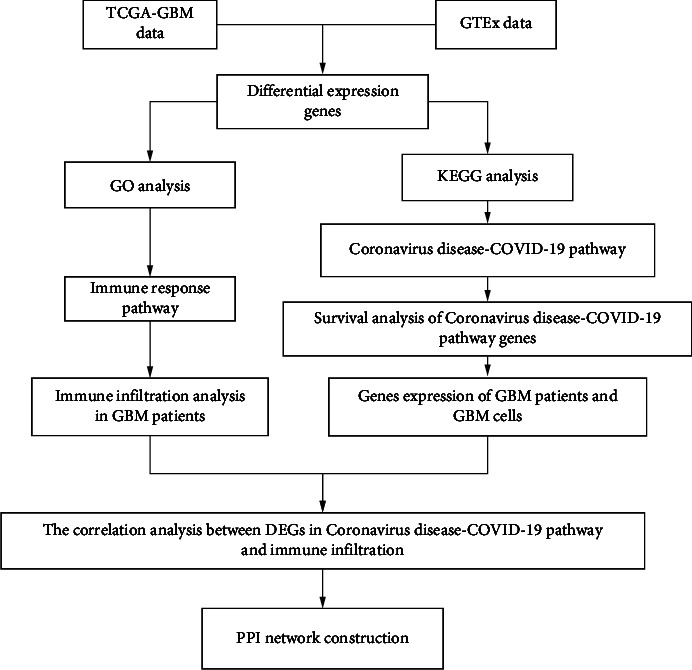
The flow diagram of this study.

**Figure 2:**
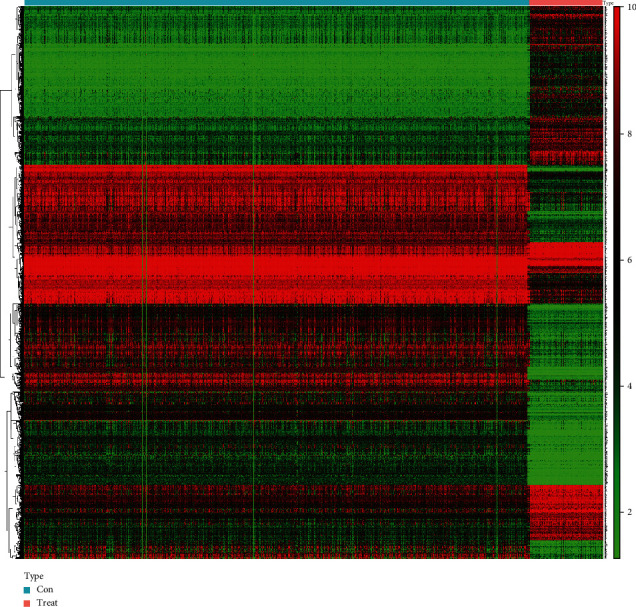
Heat map of DEGs. The red color and green color represented the upregulated and downregulated genes, respectively.

This study was conducted according to the flow chart (Figure 1). The GBM-GTEx and GBM-TCGA databases were used to identify a total of 1923 DEGs. Then, we screened 1118 upregulated DEGs and 805 downregulated DEGs in GBM samples compared with nontumor samples (Figure 2 and Supplementary Table S1).

### 2.2. GO Analysis of DEGs

**Figure 3:**
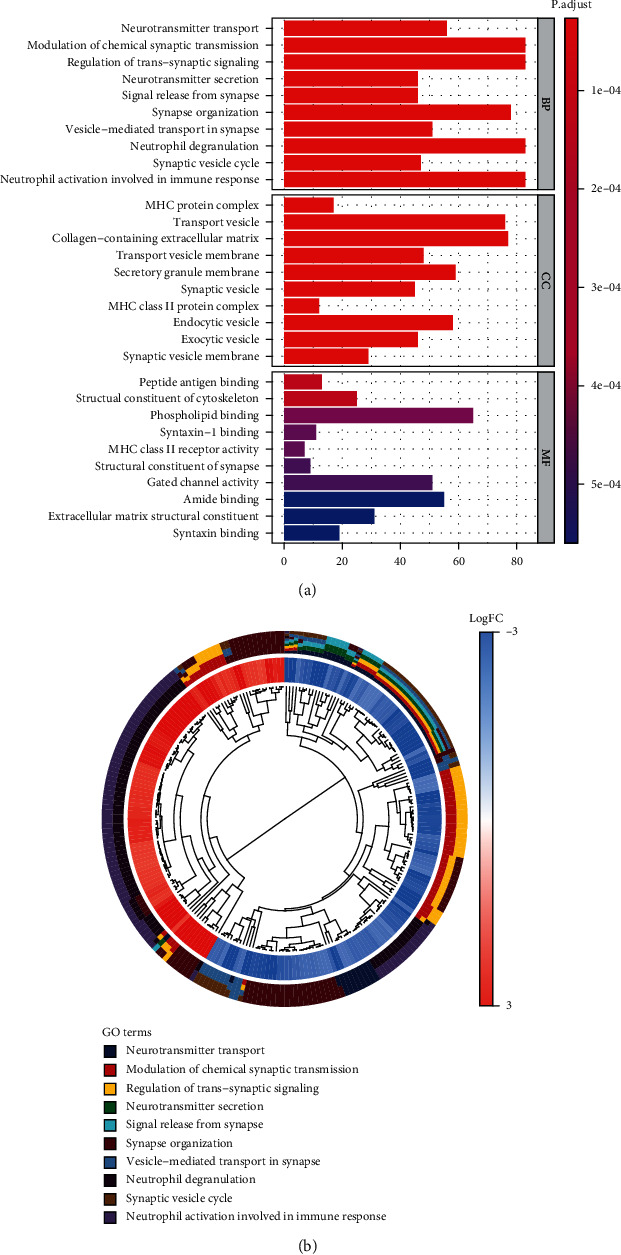
GO enrichment analysis of DEGs. (a) Biological process, cell components, and molecular function enrichment analyses of DEGs. (b) GO cluster analyses.

To accurately study the function of 1923 DEGs in glioblastoma, we performed GO analysis using *R* software. The results showed that the most enriched BP terms were modulation of chemical synaptic transmission, neurotransmitter secretion, neutrophil activation involved in immune response, synapse organization, and so on. CCs analyses revealed that the DEGs were primarily enriched in transport vesicle and collagen-containing. MF analyses revealed that the DEGs were primarily enriched in phospholipid binding, gated channel activity, and so on (Figure 3(a)). Gene cluster analysis on BP modules with the top *P* value indicated that DEGs were significantly related to signal transduction processes and neutrophil activation involved in the immune response pathway (Figure 3(b)).

### 2.3. KEGG Pathway Analysis of DEGs

**Figure 4:**
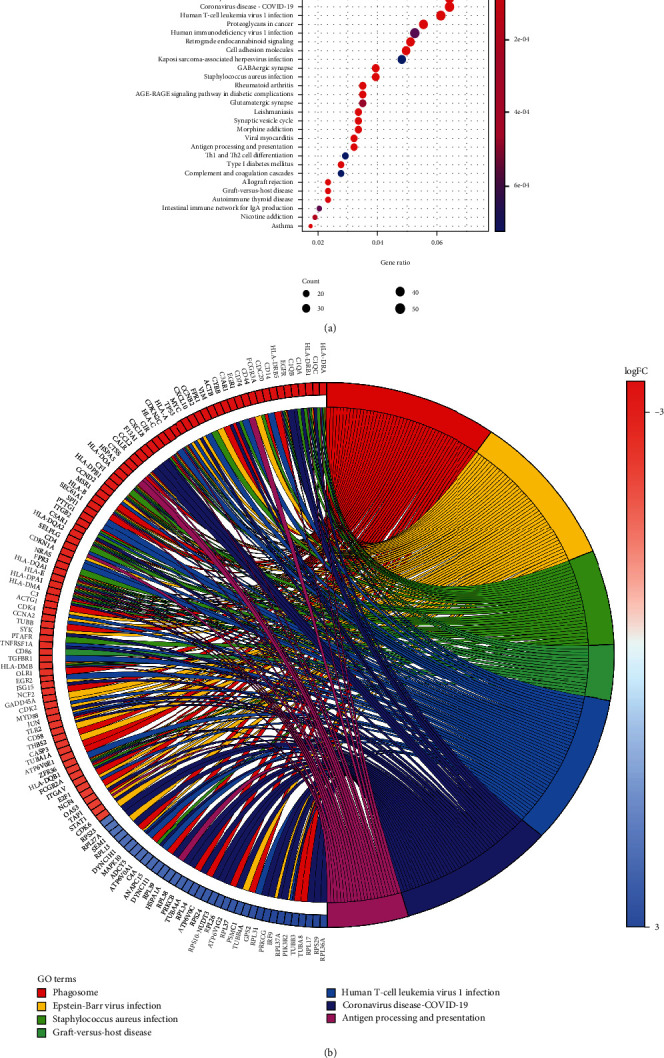
KEGG pathway enrichment analysis of DEGs. (a) KEGG pathway analysis of DEGs. (b) KEGG pathway cluster analyses.

To elucidate the role of 1923 DEGs in glioblastoma, we used the KEGG pathway analysis. The results showed that the coronavirus disease-COVID-19, prion disease, Epstein-Barr virus infection, and phagosome pathways were significantly affected with *P* < 0.001 (Figure 4(a)). Cluster analysis showed that 44 DEGs were significantly enriched in the coronavirus disease-COVID-19 pathway (Figure 4(b)).

### 2.4. Survival Analysis of DEGs

**Figure 5:**
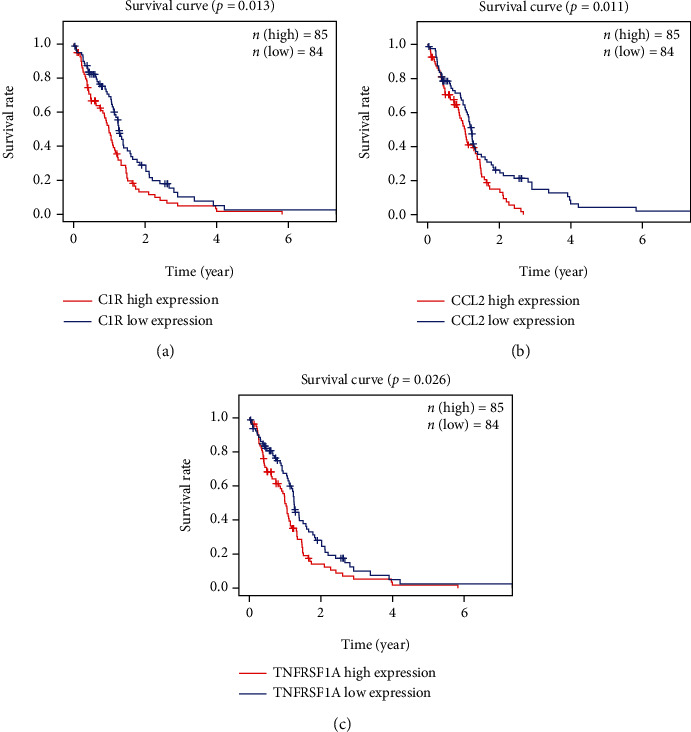
The correlation between the expression levels of three key DEGs and the survival rate of GBM patients: (a) C1R, (b) CCL2, and (c) TNFRSF1A.

To study the prognostic values of DEGs enriched in the pathways of coronavirus disease-COVID-19, we analyzed the overall survival of 169 GBM patients. We found that the high expression of C1R, CCL2, and TNFRSF1A genes was particularly associated with the low survival in GBM patients (Figure 5), while other 41 DEGs enriched in the pathway of coronavirus disease-COVID-19 were not significantly connected with the overall survival of GBM patients (Supplementary Table S1). The results suggested that C1R, CCL2, and TNFRSF1A genes might be potential prognostic factors.

### 2.5. Expression Analysis of DEGs in GBM Patients and Glioblastoma Cells

**Figure 6:**
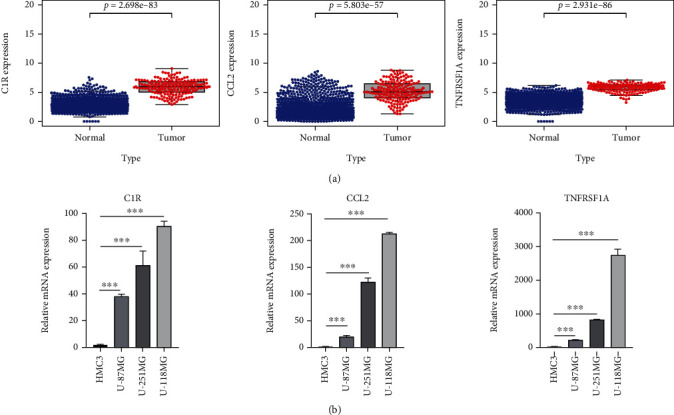
The expression levels of three key DEGs in GBM. (a) C1R, CCL2, and TNFRSF1A expression levels in the normal and GBM tissues. (b) The mRNA levels of C1R, CCL2, and TNFRSF1A in GBM cell lines. The experiments were performed independently three times. ^∗^*P* < 0.05, ^∗∗^*P* < 0.01, ^∗∗∗^*P* < 0.001.

To detect the expression levels of C1R, CCL2, and TNFRSF1A in glioblastoma, we analyzed the mRNA levels of GBM patients and glioblastoma cells (Supplementary Table S2). C1R, CCL2, and TNFRSF1A mRNA levels were significantly higher in GBM samples than in normal samples (Figure 6(a) and Supplementary Table S3), which was verified by the GEPIA online tool (Supplementary Figure S1). Furthermore, the mRNA expression levels of these three genes were positively correlated (Supplementary Figure S2). We also performed experimental validation in the cell lines. Consistently, the mRNA levels of C1R, CCL2, and TNFRSF1A were strongly upregulated in U-87MG, U-251MG, and U-1118MG cells compared with those in HMC3 cells (Figure 6(b)). The results suggested that the expression of C1R, CCL2, and TNFRSF1A might promote the development of glioblastoma.

### 2.6. Immune Infiltration Analysis in GBM Patients

**Figure 7:**
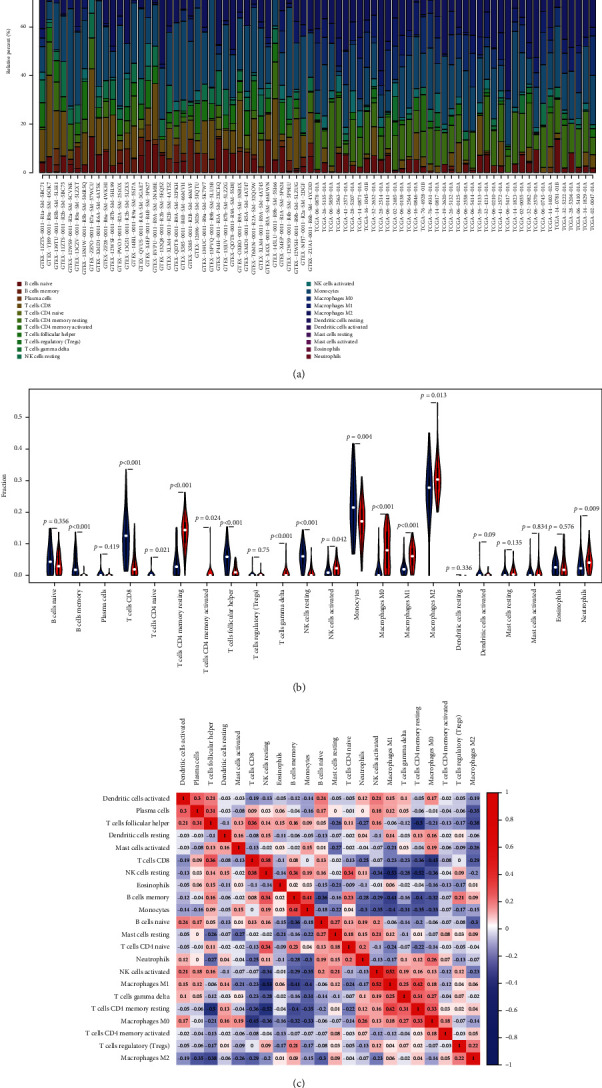
The proportion of immune cell abundance and correlation analysis of immune cells in GBM. (a) The landscape of immune infiltration in GBM and the difference of immune infiltration between tumor tissue and paired normal tissue. (b) Violin plot visualizing differentially infiltrated immune cells. The red color and green color represented normal tissue and tumor tissue, respectively. (c) Correlation heat map depicting correlations between infiltrating immune cells in GBM.

GO analysis suggested that DEGs were significantly associated with the immune response function. To determine which specific immune cells were responsible, we performed tumor cell immune infiltration analysis. The results indicated a significant difference in the proportion of immune cells in tumor and normal tissues. Compared with normal tumor tissues, GBM tissues contained more T cell CD4 memory resting, T cell gamma delta, NK cells activated, macrophage M0, macrophage M1, and macrophage M2, but it contained relative fewer B cell memory, T cell CD8, T cell CD4 naive, T cell follicular helper, and NK cell resting (Figures 7(a) and 7(b)). T cell follicular helper and NK cell resting were indicated a negative correlation with T cell CD4 memory resting (Pearson correlation = −0.50 and Pearson correlation = −0.52), while macrophage M1 and NK cells activated showed a moderate positive correlation (Pearson correlation = 0.52) (Figure 7(c)). In brief, these results suggested that the immune response to GBM was a complex network and was carried out in a rigorous manner.

### 2.7. C1R/CCL2/TNFRSF1A Genes Were Correlated with Immune Infiltration in GBM

**Figure 8:**
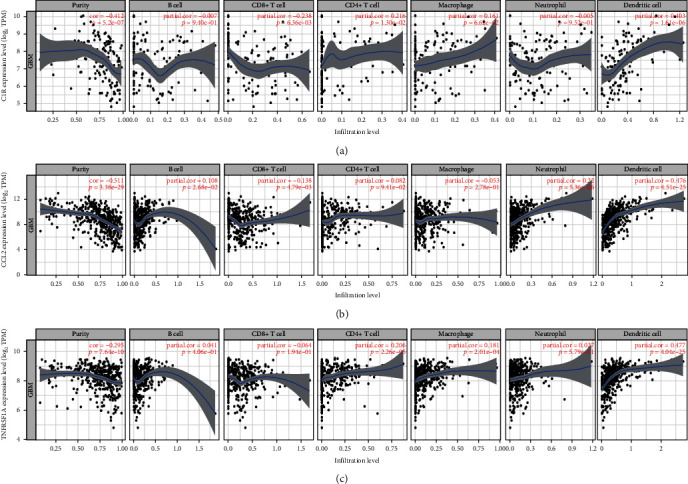
The correlation between three key DEGs and immune infiltration. Scatter plot showing the correlation of C1R (a), CCL2 (b), and TNFRSF1A (c) with immune cells, ^∗^*P* < 0.05.

To determine whether C1R, CCL2, and TNFRSF1A could be used as immunotherapy targets for GBM, we studied the correlation between the mRNA levels of C1R/CCL2/TNFRSF1A and GBM immune infiltration. The C1R expression was found to be negatively correlated with B cells, CD8^+^ T cells, and neutrophil infiltration and positively associated with CD4^+^ T cells, macrophages, and dendritic cell infiltration (Figure 8(a)). The expression of CCL2 was found to be negatively correlated with CD8+ T cells and macrophage infiltration and positively associated with B cells, CD4^+^ T cells, neutrophil, and dendritic cell infiltration (Figure 8(b)). The TNFRSF1A expression was negatively correlated with CD8^+^ T cell infiltration and positively associated with B cells, CD4^+^ T cells, macrophages, neutrophil, and dendritic cell infiltration (Figure 8(c)). These data suggested that there might be a correlation between coronavirus disease-COVID-19 and immune infiltration of GBM.

### 2.8. PPI Network Analysis of C1R/CCL2/TNFRSF1A Genes

**Figure 9:**
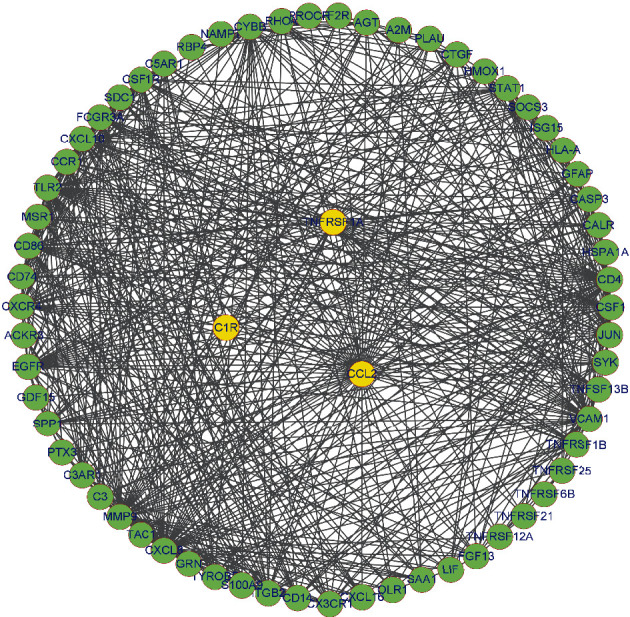
Construction of PPI network. Three key genes were marked with yellow circles. The interacted genes were marked with green circles.

To determine the important genes and key gene modules, the PPI network of C1R, CCL2, and TNFRSF1A genes was constructed, containing 62 nodes and 615 edges. The interacted genes were A2M, ACKR3, AGT, C3, C3AR1, C5AR1, CALR, CASP3, CCR1, CD14, CD4, CD74, CD86, CSF1, CSF1R, CTGF, CX3CR1, CXCL10, CXCL16, CXCL8, CXCR4, CYBB, EGFR, F2R, FCGR3A, FGF13, GDF15, GFAP, GRN, HLA-A, HMOX1, HSPA1A, ISG15, ITGB2, JUN, LIF, MMP9, MSR1, NAMPT, OLR1, PLAU, PROCR, PTX3, RBP4, RHOA, S100A9, SAA1, SDC1, SOCS3, SPP1, STAT1, SYK, TAC1, TLR2, TNFRSF12A, TNFRSF1B, TNFRSF21, TNFRSF25, TNFRSF6B, TNFSF13B, TYROBP, and VCAM1, which were mainly associated with the immune system **(**Figure 9 and Supplementary Table S4).

## 3. Discussion

Glioblastoma multiforme was one of the most deadly brain tumors. Surgical resection was the standard treatment for GBM, followed by chemotherapy with temozolomide (TMZ) and radiotherapy [8]. With the development of gene therapy, immunotherapy, and vaccine therapy, GBM treatment has been improved, but treatment options for controlling GBM recurrence were limited. GBM caused systemic immunosuppression and local immune dysfunction, leading to a more complex relationship between GBM and the peripheral tumor microenvironment (TME) [2, 9]. TME has been found to play a role in tumor genesis, development, and migration, as well as the development of malignancy and therapeutic resistance in a growing number of studies. TME cell composition and immune cell accessibility varied greatly between GBM subtypes and patients. These factors led to the immunosuppression of GBM, which further led to the failure of immunotherapy. The identification of immune genes and immune cell types that were actively involved in TME could help clarify the general mechanism of GBM immune suppression.

Moreover, in studies that have been reported, the onset of COVID-19 caused an activation of the immune system. SARS-COV-2 bound to the host ACE2 receptor through the viral spike protein receptor binding domain (RBD) and then invaded host cells to generate an immune cascade mechanism. Mucosal-associated invariant T (MAIT) cells and *γδ*T cells released inflammatory cytokines to respond to invasion [10]. Early response immune effector cells, including CTL and NK cells, were activated to fight the virus [11]. A number of studies have shown that the sign of COVID-19 was the cytokine storm with elevated levels of interleukin-6 (IL-6), IL-1*β*, complement C1r (C1R), tumor necrosis factor-alpha (TNF-*α*), chemokine (C-C-motif) ligand 2 (CCL2), and granulocyte-macrophage colony-stimulating factor (GM-CSF) [12].

In this study, enrichment pathway analysis indicated that DEGs in glioblastoma were strongly enriched in pathways of immune response and coronavirus disease-COVID-19 (Figures 3 and 4). The high expression levels of C1R, CCL2, and TNFRSF1A in coronavirus disease-COVID-19 pathway were significantly related to the low survival in GBM patients (Figure 5). Moreover, the mRNA expression levels of C1R, CCL2, and TNFRSF1A in glioblastoma cells or in GBM patients were found to be strongly upregulated (Figure 6 and Supplementary Figure S1), and the mRNA expression levels of these three genes were positively associated with each other (Supplementary Figure S2). The results indicated that the expression of C1R, CCL2, and TNFRSF1A might promote the development of glioblastoma and might be used as molecular biomarkers of prognosis and immune infiltration in GBM patients in the future. A recent study found the increase of CCL2 promoted carcinogenesis through esophageal mucosal inflammation [13]. Moreover, CCL2 could promote the survival and proliferation of THP-1, prostate cancer cell line PC3, renal cell carcinoma cell line 786-O and KAKI-1, and lung cancer cell line A549 [14, 15]. The growth of cutaneous squamous cell carcinoma and non-small-cell lung cancer was promoted by tumor-cell-derived complement components C1r and C1s [16]. However, there are few reports about the relationship between these three genes and glioblastoma and how C1R, CCL2, and TNFRSF1A promote the occurrence and development of GBM which requires further research in the future.

Our results also found that C1R, CCL2, and TNFRSF1A gene expression was significantly associated with GBM immune infiltration. The expression of C1R, CCL2, and TNFRSF1A was closely related to B cells, CD8+ T cells, CD4+ T cells, neutrophils, macrophages, and dendritic cell infiltration (Figure 8). Recent studies found that macrophages, monocytes, NK cells, and memory T lymphocytes were activated by the CCL2-CCR2 axis, thereby stimulating the release of proinflammatory cytokines such as tumor necrosis factor- (TNF-) *α*, interleukin-1, and interleukin-6 [17]. Moreover, macrophages were also activated by CCL2 to secrete tissue repair factors, such as transforming growth factor (TGF)–*β*, platelet-derived growth factor (PDGF), and vascular endothelial growth factor (VEGF) [18]. TNFRSF1A encoded a member of the TNF receptor superfamily of proteins. The encoded receptor was found in soluble and membrane-bound forms and interacted with soluble and membrane-bound forms, respectively, of its ligand, TNF-*α* [19, 20]. The binding of membrane-bound TNF-*α* to membrane-bound receptor induced receptor trimerization and activation, playing a function in cell survival, apoptosis, and inflammation. However, it needs further study how C1R, CCL2, and TNFRSF1A affect the immune infiltration of GBM cells.

The novel coronavirus disease outbreak of 2019 (COVID-19) has emerged as the world's most serious public health threat, infecting over 1.7 million people and killing over 100,000. Recent studies have shown that cancer patients are more likely to contract COVID-19 and have higher mortality rates [12]. The novel coronavirus SARS-CoV-2 binds to human angiotensin-converting enzyme II (ACE2) through its expressed S-protein and enters the cell, while ACE2 activates the expression of CCL2, and studies have also shown that the pathogenesis of COVID-19 is closely related to the excessive release of CCL2 [21–23]. Our results found that the expression levels of C1R, CCL2, and TNFRSF1A in GBM cells and patients were very high (Figure 6), and 35 gene biomarkers of immune cells were significantly correlated with C1R, CCL2, and TNFRSF1A (Supplementary Table S5). We believe that the C1R, CCL2, and TNFRSF1A genes in the coronavirus-COVID-19 pathway may be activated in GBM patients, making GBM patients more susceptible to novel coronavirus infection. Some Chinese herbs such as quercetin and baicalin can bind to CCL2 receptor and thus act on immune response signaling pathway, tumor necrosis factor signaling pathway, and influenza A signaling pathway, which may play a dual role in anti-COVID-19 and GBM process.

## 4. Methods

### 4.1. Data Collection and DEG Screening

The GTEx (Gen Tissue Expression) database studied more than 7000 postmortem samples from 449 previously healthy human donors, covering 44 tissues. We downloaded 1151 nontumor samples from the GTEx database to make up for the shortage of normal samples in the TCGA database and downloaded 169 GBM tumor samples and 5 nontumor samples from the TCGA database on the UCSC Xena (http://xena.ucsc.edu/) website. We used limma package of *R* software (version4.0.2) to identify DEGs between nontumor samples and GBM samples. The criteria for DEG screening were *P*.adjust <0.05.

### 4.2. GO and KEGG Pathway Analysis

GO analysis is a common method for functional enrichment studies on a large scale. KEGG is a database with a wide range of functions, storing rich data on biological pathways, genomes, drugs, and diseases. First, the official symbol of DEGs was converted through the org.hs.eg.db package based on *R* software (version 4.0.2). We used the Cluster Profiler package and the Go Plot package for GO and KEGG pathway analyses and cluster analyses, respectively. We used the limma package to identify the differential expression of mRNAs with |log(foldchange)| > 2 thresholds and performed GO and KEGG analysis with the ggplot2 package in *R* software. *P*.adjust <0.05 was considered statistically significant.

### 4.3. Survival Analysis

According to the median value of the gene expression, we divided all samples into low and high expression groups. 169 TCGA tumor samples were evaluated for survival differences (Kaplan-Meier method) with *R* software. *P* < 0.05 was considered statistically significant.

### 4.4. Cell Experiments

U-87MG, U-251MG, U-118MG, and HMC3 cells (iCell Bioscience Inc., Procell Inc., Identified by STR) were cultured on DMEM medium (Gibco, Thermo Fisher Scientific, USA) supplemented with 10% fetal bovine serum (Life Technologies, USA) and 1% streptomycin and penicillin (100 *μ*g/ml streptomycin and 100 U/ml penicillin, Life Technologies) at 37°C in 5% carbon dioxide chamber. Total RNA was extracted from cells for qRT-PCR analyses. The primers were as follows: C1R-fw: gctgcccacaccctgtatc. C1R-rv: gcccaggaacacatccaaagag. CCL2-fw: ctcgctcagccaggtaagg. CCL2-rv: actgtgggtaccacgtctgc. TNFRSF1A-fw: cctggacagaccgagtcc. TNFRSF1A-rv: ctttgtccctggtctcacc.

### 4.5. Immune Infiltration Analysis

The TIMER web server provides a rich resource for comprehensive analysis of immune infiltrations in different cancer types (https://cistrome.shinyapps.io/timer/). TIMER software was used to analyze the correlation between the abundance of 6 immune infiltrates and gene expression. Moreover, we used a two-sided Wilcoxon rank sum test to detect the effect of different somatic copy number changes on tumor invasion levels.

### 4.6. Protein–Protein Interaction Network Construction

STRING was a bioinformatics database that constructed the protein–protein interaction network (PPI) of DEGs based on the predicted and known PPIs. Subsequently, we analyzed the functional interactions among proteins and visualized the PPI network with Cytoscape software (version 3.7.2).

### 4.7. Statistical Analysis

All the data were processed and analyzed by the *R* software (version 4.0.2). Mann–Whitney test and *t*-test were used to compare the two groups of data and the optimal cut-off value generated by the *R* package “SURv Cutpoint” function for survival analyses. Moreover, we divided the expression levels by dichotomy and evaluated the statistical significance of cell line experiments using the *t*-test in the GraphPad Prism version 8 software. *P* < 0.05 was considered statistically significant (^∗^*P* < 0.05, ^∗∗^*P* < 0.01, ^∗∗∗^*P* < 0.001).

## Data Availability

Publicly available datasets were analyzed in this study, and these can be found in Genotype-Tissue Expression Program (https://commonfund.nih.gov/GTEx/) and The Cancer Genome Atlas Program (https://portal.gdc.cancer.gov). The authors confirm that the data supporting the findings of this study are available within the article and its Supplementary materials.

## Supplementary Materials

Supplementary Materials Supplementary Information. The online version contains supplementary material available at Supplementary Figure S1: C1R, CCL2, and TNFRSF1A expression levels in the GBM and normal issues with GEPIA online tool: (a) C1R, (b) CCL2, and (c) TNFRSF1A. Supplementary Figure S2: the correlation between C1R, CCL2, and TNFRSF1A with GEPIA online tool. (a) C1R and TNFRSF1A, (b) C1R and CCL2, (c) CCL2 and TNFRSF1A. Supplementary Table S1: the correlation between the expression level of other DEGs and the survival rate of glioma patients. Supplementary Table S2: the 1923 DEGs between GBM samples and nontumor samples. Supplementary Table S3: the C1R, CCL2, and TNFRSF1A expression levels with the normal and GBM tissues. Supplementary Table S4: the 62 nodes interacting with C1R, CCL2, and TNFRSF1A. Supplementary Table S5: the correlation between three key DEGs and gene biomarkers of immune cells in GBM.
