# Supplementary material for: C1R, CCL2, and TNFRSF1A Genes in Coronavirus Disease-COVID-19 Pathway Serve as Novel Molecular Biomarkers of GBM Prognosis and Immune Infiltration
Source: Dis Markers. 2022 Jun 18;2022:8602068. doi: 10.1155/2022/8602068 (PMC9206210; doi:10.1155/2022/8602068)
Supplement: Supplementary Materials — Supplementary Information. The online version contains supplementary material available at Supplementary Figure S1: C1R, CCL2, and TNFRSF1A expression levels in the GBM and normal issues with GEPIA online tool: (a) C1R, (b) CCL2, and (c) TNFRSF1A. Supplementary Figure S2: the correlation between C1R, CCL2, and TNFRSF1A with GEPIA online tool. (a) C1R and TNFRSF1A, (b) C1R and CCL2, (c) CCL2 and TNFRSF1A. Supplementary Table S1: the correlation between the expression level of other DEGs and the survival rate of glioma patients. Supplementary Table S2: the 1923 DEGs between GBM samples and nontumor samples. Supplementary Table S3: the C1R, CCL2, and TNFRSF1A expression levels with the normal and GBM tissues. Supplementary Table S4: the 62 nodes interacting with C1R, CCL2, and TNFRSF1A. Supplementary Table S5: the correlation between three key DEGs and gene biomarkers of immune cells in GBM. [file 8602068.f1.docx]

## Supplementary Figures


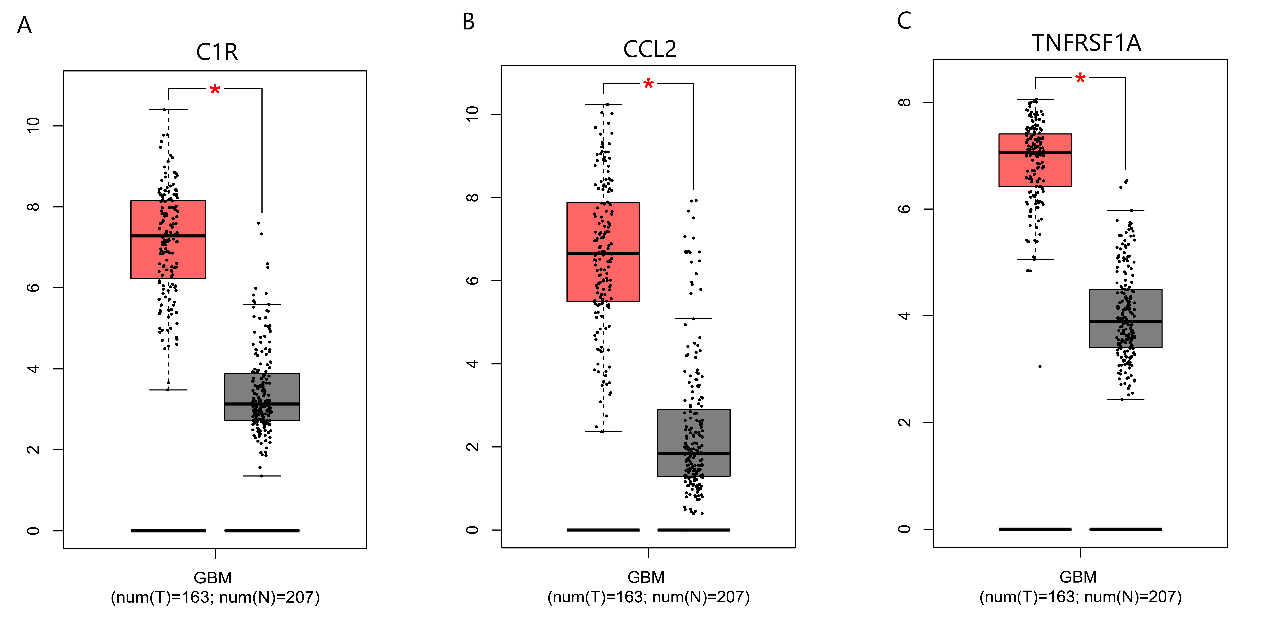


**Supplementary Figure S1.** C1R, CCL2 and TNFRSF1A expression levels in the GBM and normal issues with GEPIA online tool. (A) C1R, (B) CCL2, (C) TNFRSF1A.


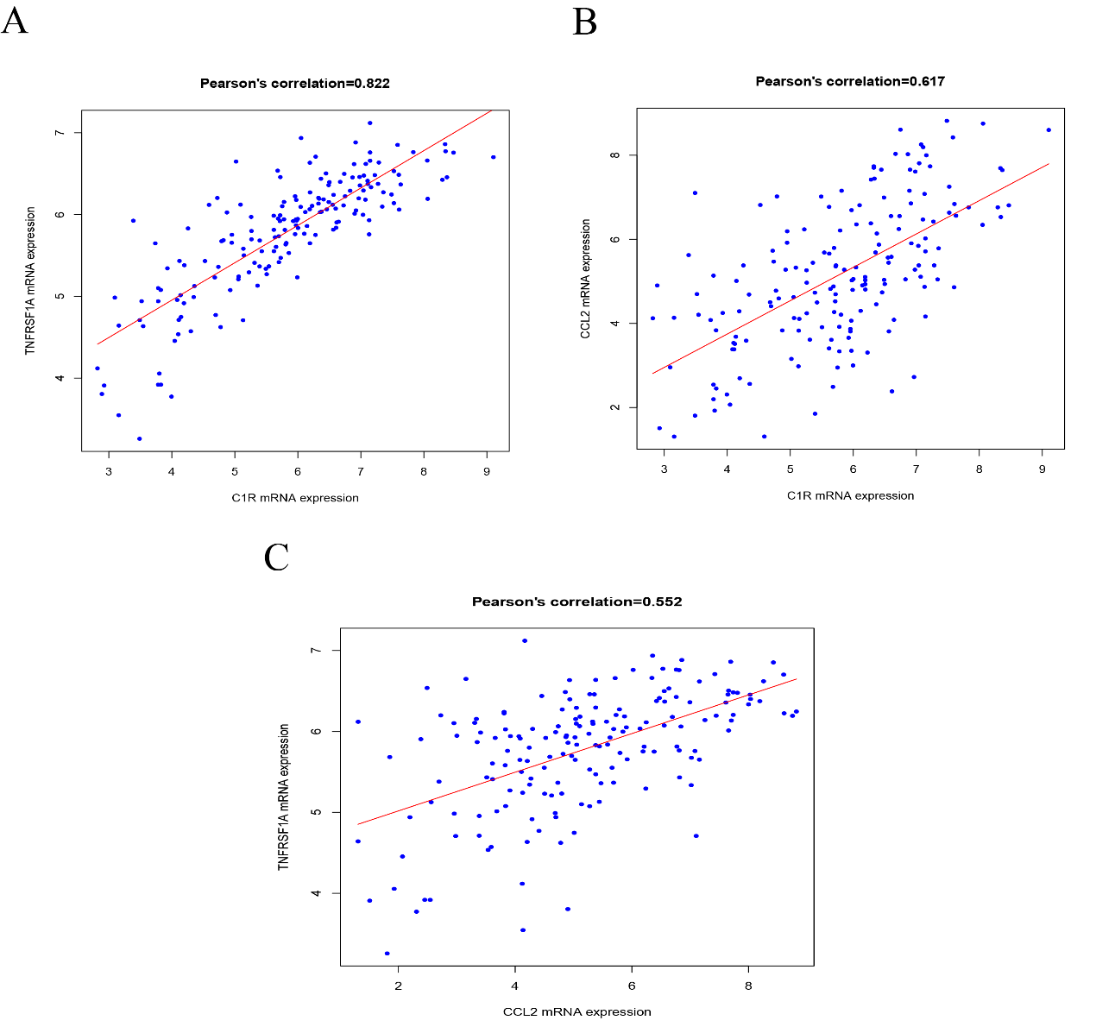


**Supplementary Figure S2.** The correlation between C1R, CCL2 and TNFRSF1A with GEPIA online tool. (A) C1R and TNFRSF1A, (B) C1R and CCL2, (C) CCL2 and TNFRSF1A.

**Supplementary Tables**

**Supplementary Table S1.** The correlation between the expression level of other DEGs and survival rate of glioma patients.

| gene | sample number(high) | | sample number(low) | | Pvalue |
| --- | --- | --- | --- | --- | --- |
| RPL13 | | 85 | | 84 | 0.058655 |
| PIK3R2 | | 85 | | 84 | 0.071534 |
| RPL39 | | 85 | | 84 | 0.084962 |
| MYD88 | | 85 | | 84 | 0.086643 |
| RPS23 | | 85 | | 84 | 0.087497 |
| CXCL8 | | 85 | | 84 | 0.097776 |
| F13A1 | | 85 | | 84 | 0.102036 |
| GNB4 | | 85 | | 84 | 0.118784 |
| FCGR2A | | 85 | | 84 | 0.134288 |
| RPL27A | | 85 | | 84 | 0.178394 |
| C5AR1 | | 85 | | 84 | 0.190441 |
| C1QB | | 85 | | 84 | 0.195721 |
| GNG12 | | 85 | | 84 | 0.199583 |
| RPS29 | | 85 | | 84 | 0.203879 |
| RPL17 | | 85 | | 84 | 0.219142 |
| IRF9 | | 85 | | 84 | 0.23589 |
| C1QC | | 85 | | 84 | 0.237119 |
| RPL37A | | 85 | | 84 | 0.273319 |
| C3 | | 85 | | 84 | 0.359448 |
| ISG15 | | 85 | | 84 | 0.380364 |
| GRIN1 | | 85 | | 84 | 0.380893 |
| TLR2 | | 85 | | 84 | 0.385606 |
| STAT1 | | 85 | | 84 | 0.392095 |
| RYR1 | | 85 | | 84 | 0.392784 |
| PRKCB | | 85 | | 84 | 0.409935 |
| C3AR1 | | 85 | | 84 | 0.457967 |
| RPL36A | | 85 | | 84 | 0.458108 |
| ITPR1 | | 85 | | 84 | 0.471507 |
| RPL26 | | 85 | | 84 | 0.502874 |
| PRKCG | | 85 | | 84 | 0.510986 |
| EGFR | | 85 | | 84 | 0.512175 |
| GNB5 | | 85 | | 84 | 0.527884 |
| CYBB | | 85 | | 84 | 0.54143 |
| KCNJ9 | | 85 | | 84 | 0.579038 |
| JUN | | 85 | | 84 | 0.594791 |
| ADCY5 | | 85 | | 84 | 0.615734 |
| RPL34 | | 85 | | 84 | 0.623154 |
| CXCL10 | | 85 | | 84 | 0.645423 |
| GNAO1 | | 85 | | 84 | 0.667472 |
| CAMK2B | | 85 | | 84 | 0.689835 |
| RPL37 | | 85 | | 84 | 0.704627 |
| SYK | | 85 | | 84 | 0.740521 |
| GRIN2C | | 85 | | 84 | 0.749215 |
| MAPK10 | | 85 | | 84 | 0.749981 |
| RPS24 | | 85 | | 84 | 0.750324 |
| OAS3 | | 85 | | 84 | 0.754124 |
| CAMK2A | | 85 | | 84 | 0.770325 |
| C4A | | 85 | | 84 | 0.783715 |
| RPL38 | | 85 | | 84 | 0.846044 |
| RYR2 | | 85 | | 84 | 0.88817 |
| C1QA | | 85 | | 84 | 0.921639 |
| GNG5 | | 85 | | 84 | 0.94193 |
| RPL31 | | 85 | | 84 | 0.958821 |

**Supplementary Table S2.** The 1923 DEGs between GBM samples and non-tumor samples.

| gene | conMean | treatMean | logFC | Pvalue | P.adjust |  |
| --- | --- | --- | --- | --- | --- | --- |
| FP236383.2 | 10.72866 | 0.368689 | -10.36 | 4.14E-96 | 9.90E-95 |  |
| FP236383.3 | 10.72864 | 0.422214 | -10.3064 | 2.15E-95 | 3.74E-94 |  |
| FP671120.4 | 10.72812 | 0.986513 | -9.74161 | 5.14E-94 | 5.25E-93 |  |
| RNA5-8SN3 | 8.430694 | 0.059555 | -8.37114 | 1.09E-95 | 2.23E-94 |  |
| RNA5-8SN2 | 8.430694 | 0.118897 | -8.3118 | 4.00E-95 | 6.13E-94 |  |
| RNA5-8SN1 | 8.430694 | 0.138192 | -8.2925 | 1.88E-95 | 3.34E-94 |  |
| EEF1G | 8.057277 | 0.208973 | -7.8483 | 3.48E-94 | 3.76E-93 |  |
| NPIPA9 | 7.572817 | 0.057675 | -7.51514 | 3.30E-95 | 5.22E-94 |  |
| RN7SL1 | 7.317841 | 0 | -7.31784 | 5.01E-97 | 2.11E-95 |  |
| AC138969.1 | 7.630914 | 0.347717 | -7.2832 | 1.98E-95 | 3.48E-94 |  |
| MBP | 11.42189 | 4.844158 | -6.57773 | 1.74E-93 | 1.58E-92 |  |
| AC093330.1 | 6.438597 | 0.014902 | -6.42369 | 9.29E-96 | 1.97E-94 |  |
| TOMM6 | 6.227191 | 0 | -6.22719 | 5.01E-97 | 2.11E-95 |  |
| AC011603.2 | 6.209809 | 0.152696 | -6.05711 | 1.30E-91 | 9.13E-91 |  |
| MEG3 | 8.639153 | 2.776021 | -5.86313 | 8.31E-95 | 1.11E-93 |  |
| NPIPA1 | 6.854832 | 1.075965 | -5.77887 | 1.05E-94 | 1.35E-93 |  |
| HEPN1 | 5.563333 | 0 | -5.56333 | 5.02E-97 | 2.11E-95 |  |
| ARL6IP4 | 6.942001 | 1.393683 | -5.54832 | 2.74E-94 | 3.07E-93 |  |
| AC005944.1 | 5.528116 | 0 | -5.52812 | 1.05E-96 | 3.43E-95 |  |
| KLC1 | 7.613921 | 2.106106 | -5.50781 | 1.16E-95 | 2.35E-94 |  |
| EIF3C | 6.996252 | 1.594699 | -5.40155 | 2.31E-94 | 2.63E-93 |  |
| AL354740.1 | 5.446152 | 0.163112 | -5.28304 | 7.53E-95 | 1.03E-93 |  |
| FXYD1 | 6.725627 | 1.461154 | -5.26447 | 1.77E-95 | 3.20E-94 |  |
| AC004057.1 | 7.21691 | 1.964859 | -5.25205 | 5.54E-94 | 5.60E-93 |  |
| AC008738.7 | 5.216249 | 0 | -5.21625 | 2.21E-96 | 6.00E-95 |  |
| AC093484.3 | 5.212229 | 0 | -5.21223 | 2.92E-95 | 4.75E-94 |  |
| NPIPB5 | 6.107247 | 0.962515 | -5.14473 | 3.67E-94 | 3.92E-93 |  |
| BSCL2 | 7.264987 | 2.15884 | -5.10615 | 1.27E-95 | 2.50E-94 |  |
| ADIRF | 5.078672 | 0 | -5.07867 | 5.02E-97 | 2.11E-95 |  |
| RPL21P16 | 8.149288 | 3.12994 | -5.01935 | 3.51E-94 | 3.78E-93 |  |
| AL008729.2 | 5.107346 | 0.114421 | -4.99292 | 2.58E-95 | 4.31E-94 |  |
| POLR2F | 5.394592 | 0.404741 | -4.98985 | 1.06E-95 | 2.18E-94 |  |
| NPIPB3 | 5.501693 | 0.529193 | -4.9725 | 1.85E-94 | 2.19E-93 |  |
| GTF2IP1 | 5.835118 | 0.863042 | -4.97208 | 4.27E-94 | 4.47E-93 |  |
| NPIPB12 | 5.215611 | 0.267668 | -4.94794 | 2.03E-94 | 2.35E-93 |  |
| MAGED4B | 5.505231 | 0.58162 | -4.92361 | 8.81E-94 | 8.55E-93 |  |
| GRIN1 | 6.152017 | 1.251929 | -4.90009 | 7.56E-88 | 3.98E-87 |  |
| RNASEK | 7.511848 | 2.636314 | -4.87553 | 4.61E-95 | 6.86E-94 |  |
| PDXP | 6.10696 | 1.257749 | -4.84921 | 1.27E-95 | 2.50E-94 |  |
| SLC12A5 | 5.976411 | 1.203568 | -4.77284 | 5.90E-94 | 5.93E-93 |  |
| DBNDD2 | 7.217461 | 2.456362 | -4.7611 | 1.85E-95 | 3.30E-94 |  |
| AC007192.2 | 4.744827 | 0 | -4.74483 | 6.70E-96 | 1.48E-94 |  |
| 4-Sep | 8.214864 | 3.485351 | -4.72951 | 3.36E-95 | 5.28E-94 |  |
| SNORD3A | 4.748665 | 0.049977 | -4.69869 | 4.96E-95 | 7.27E-94 |  |
| CCNI2 | 5.282948 | 0.595956 | -4.68699 | 1.28E-95 | 2.52E-94 |  |
| LINC00599 | 5.627789 | 0.972327 | -4.65546 | 4.44E-91 | 2.97E-90 |  |
| CKMT1B | 5.120369 | 0.535394 | -4.58498 | 3.63E-94 | 3.89E-93 |  |
| MATR3 | 4.979704 | 0.398014 | -4.58169 | 1.20E-95 | 2.41E-94 |  |
| SNHG14 | 6.146843 | 1.574238 | -4.57261 | 1.75E-97 | 1.61E-95 |  |
| RNA5SP216 | 4.564625 | 0 | -4.56462 | 1.11E-73 | 3.42E-73 |  |
| AC087393.2 | 4.545192 | 0 | -4.54519 | 6.08E-95 | 8.59E-94 |  |
| U2AF1L5 | 4.606095 | 0.067978 | -4.53812 | 2.52E-95 | 4.22E-94 |  |
| CPLX2 | 6.79384 | 2.270757 | -4.52308 | 2.16E-82 | 8.72E-82 |  |
| TRAPPC5 | 5.252569 | 0.734106 | -4.51846 | 2.18E-95 | 3.78E-94 |  |
| RBFOX3 | 4.989935 | 0.487673 | -4.50226 | 2.32E-91 | 1.59E-90 |  |
| CALY | 5.687308 | 1.199137 | -4.48817 | 2.16E-89 | 1.26E-88 |  |
| NEFM | 5.614884 | 1.15846 | -4.45642 | 4.68E-87 | 2.37E-86 |  |
| AC090498.1 | 8.743724 | 4.291557 | -4.45217 | 3.04E-94 | 3.35E-93 |  |
| ST8SIA3 | 5.266579 | 0.824094 | -4.44249 | 5.91E-90 | 3.59E-89 |  |
| MOBP | 6.442492 | 2.00599 | -4.4365 | 4.18E-79 | 1.51E-78 |  |
| AP1G2 | 5.991278 | 1.559553 | -4.43172 | 3.29E-95 | 5.21E-94 |  |
| AC092683.1 | 4.47777 | 0.047714 | -4.43006 | 5.42E-96 | 1.23E-94 |  |
| AC133550.1 | 4.430121 | 0.001132 | -4.42899 | 5.13E-95 | 7.49E-94 |  |
| ARPP21 | 5.658739 | 1.236167 | -4.42257 | 7.52E-93 | 6.16E-92 |  |
| SERF1B | 5.055908 | 0.675349 | -4.38056 | 7.70E-95 | 1.04E-93 |  |
| PRKCZ | 6.086612 | 1.712323 | -4.37429 | 1.41E-95 | 2.67E-94 |  |
| HERC2P3 | 5.118398 | 0.769097 | -4.3493 | 8.16E-95 | 1.09E-93 |  |
| AC068831.4 | 4.342057 | 0 | -4.34206 | 5.02E-97 | 2.11E-95 |  |
| AC012321.1 | 4.33841 | 0 | -4.33841 | 5.02E-97 | 2.11E-95 |  |
| AL358781.1 | 4.334762 | 0 | -4.33476 | 2.21E-96 | 6.00E-95 |  |
| AL035252.4 | 4.622269 | 0.290403 | -4.33187 | 2.30E-81 | 8.92E-81 |  |
| CABP1 | 5.267488 | 0.948714 | -4.31877 | 1.48E-91 | 1.04E-90 |  |
| AC124068.1 | 4.301348 | 0 | -4.30135 | 5.02E-97 | 2.11E-95 |  |
| EIF4A1 | 6.224334 | 1.924182 | -4.30015 | 4.17E-94 | 4.38E-93 |  |
| CBWD5 | 4.772307 | 0.484723 | -4.28758 | 1.14E-94 | 1.44E-93 |  |
| CES4A | 4.975899 | 0.688329 | -4.28757 | 2.28E-95 | 3.91E-94 |  |
| ANK3 | 5.02521 | 0.739761 | -4.28545 | 1.25E-95 | 2.47E-94 |  |
| U2AF1 | 4.410923 | 0.127088 | -4.28383 | 7.84E-95 | 1.06E-93 |  |
| AC026464.4 | 4.252982 | 0.020794 | -4.23219 | 5.65E-97 | 2.26E-95 |  |
| AIFM3 | 5.270993 | 1.047398 | -4.22359 | 4.34E-94 | 4.54E-93 |  |
| RPL36A | 8.316831 | 4.123303 | -4.19353 | 4.79E-94 | 4.95E-93 |  |
| RPS29 | 9.400092 | 5.209234 | -4.19086 | 4.50E-94 | 4.68E-93 |  |
| RPL17 | 8.918177 | 4.730777 | -4.1874 | 4.78E-94 | 4.95E-93 |  |
| SARNP | 5.097446 | 0.910862 | -4.18658 | 3.14E-94 | 3.43E-93 |  |
| CAPN3 | 5.132636 | 0.955008 | -4.17763 | 9.33E-95 | 1.22E-93 |  |
| AC068385.1 | 4.177536 | 0 | -4.17754 | 4.17E-88 | 2.24E-87 |  |
| POLR2J3 | 4.90626 | 0.736103 | -4.17016 | 1.79E-94 | 2.13E-93 |  |
| PACSIN1 | 5.792445 | 1.629425 | -4.16302 | 2.17E-81 | 8.42E-81 |  |
| ARHGDIG | 6.122048 | 1.964292 | -4.15776 | 6.61E-91 | 4.34E-90 |  |
| NOMO2 | 5.9532 | 1.806271 | -4.14693 | 1.48E-95 | 2.78E-94 |  |
| RUNDC3A | 7.343949 | 3.219705 | -4.12424 | 5.49E-94 | 5.56E-93 |  |
| BOLA2B | 4.680251 | 0.559578 | -4.12067 | 5.24E-95 | 7.63E-94 |  |
| SOX10 | 4.116642 | 0 | -4.11664 | 5.02E-97 | 2.11E-95 |  |
| SNCG | 6.730849 | 2.614385 | -4.11646 | 4.46E-89 | 2.55E-88 |  |
| SPSB3 | 4.812791 | 0.69803 | -4.11476 | 1.85E-95 | 3.31E-94 |  |
| AC006449.7 | 4.114126 | 0 | -4.11413 | 1.05E-96 | 3.43E-95 |  |
| AC234031.1 | 4.177155 | 0.071776 | -4.10538 | 2.11E-94 | 2.43E-93 |  |
| PPP2R2C | 5.59283 | 1.491343 | -4.10149 | 2.70E-94 | 3.03E-93 |  |
| C18orf32 | 6.251073 | 2.150698 | -4.10038 | 3.35E-95 | 5.27E-94 |  |
| ADAP1 | 5.471148 | 1.391607 | -4.07954 | 1.85E-97 | 1.61E-95 |  |
| NPIPB4 | 4.475028 | 0.396327 | -4.0787 | 6.55E-95 | 9.14E-94 |  |
| C17orf49 | 5.124755 | 1.049056 | -4.0757 | 4.46E-94 | 4.65E-93 |  |
| SNAP25 | 8.665121 | 4.596643 | -4.06848 | 7.13E-74 | 2.22E-73 |  |
| MCF2L | 5.925955 | 1.860868 | -4.06509 | 1.41E-97 | 1.61E-95 |  |
| ZACN | 4.224979 | 0.163604 | -4.06137 | 1.60E-96 | 4.71E-95 |  |
| SULT1A3 | 4.05417 | 0.026067 | -4.0281 | 1.13E-95 | 2.31E-94 |  |
| CPNE6 | 5.07053 | 1.042554 | -4.02798 | 5.38E-93 | 4.51E-92 |  |
| STAG3L5P-PVRIG2P-PILRB | 5.034707 | 1.027513 | -4.00719 | 1.77E-94 | 2.10E-93 |  |
| IQSEC3 | 4.891687 | 0.886811 | -4.00488 | 9.46E-94 | 9.09E-93 |  |
| CARMIL2 | 4.355212 | 0.351922 | -4.00329 | 4.99E-95 | 7.31E-94 |  |
| RASA4 | 4.332733 | 0.32975 | -4.00298 | 1.15E-94 | 1.45E-93 |  |
| AL157871.1 | 3.99387 | 0 | -3.99387 | 6.70E-96 | 1.48E-94 |  |
| PPP1R1B | 7.00241 | 3.023426 | -3.97898 | 1.64E-82 | 6.65E-82 |  |
| AP003396.5 | 3.978431 | 0 | -3.97843 | 6.75E-75 | 2.15E-74 |  |
| HBA1 | 6.536729 | 2.558528 | -3.9782 | 7.89E-83 | 3.25E-82 |  |
| PSD | 6.495001 | 2.531139 | -3.96386 | 9.42E-84 | 4.04E-83 |  |
| SMIM11B | 3.949178 | 0.005785 | -3.94339 | 2.08E-96 | 5.77E-95 |  |
| TTC7B | 5.455585 | 1.512325 | -3.94326 | 1.16E-95 | 2.35E-94 |  |
| SYNE1 | 5.335004 | 1.395067 | -3.93994 | 1.31E-95 | 2.56E-94 |  |
| S100A1 | 7.478264 | 3.55359 | -3.92467 | 1.70E-75 | 5.51E-75 |  |
| TSPOAP1 | 5.723424 | 1.800224 | -3.9232 | 7.70E-95 | 1.04E-93 |  |
| GDF1 | 3.917921 | 0 | -3.91792 | 7.27E-97 | 2.68E-95 |  |
| CHMP4A | 5.407482 | 1.490729 | -3.91675 | 2.20E-94 | 2.52E-93 |  |
| AC010503.1 | 3.910741 | 0 | -3.91074 | 1.53E-96 | 4.54E-95 |  |
| JPH3 | 5.161483 | 1.259372 | -3.90211 | 2.61E-88 | 1.42E-87 |  |
| PCP4 | 6.375933 | 2.482414 | -3.89352 | 2.33E-78 | 8.18E-78 |  |
| SRRM3 | 5.170319 | 1.277189 | -3.89313 | 8.34E-89 | 4.68E-88 |  |
| AL353813.1 | 3.88886 | 0 | -3.88886 | 2.21E-96 | 6.00E-95 |  |
| TREX1 | 3.886044 | 0 | -3.88604 | 5.02E-97 | 2.11E-95 |  |
| CORO6 | 4.526983 | 0.647836 | -3.87915 | 1.89E-94 | 2.22E-93 |  |
| AC016026.1 | 3.881088 | 0.004437 | -3.87665 | 3.11E-97 | 1.79E-95 |  |
| DST | 6.776198 | 2.901906 | -3.87429 | 1.30E-95 | 2.55E-94 |  |
| USP32P1 | 4.02039 | 0.147742 | -3.87265 | 6.17E-96 | 1.39E-94 |  |
| SYNPR | 4.814536 | 0.975934 | -3.8386 | 1.96E-82 | 7.93E-82 |  |
| FAM153B | 3.893934 | 0.059418 | -3.83452 | 2.40E-96 | 6.44E-95 |  |
| GATD3B | 5.102449 | 1.287017 | -3.81543 | 7.03E-90 | 4.24E-89 |  |
| FAM153C | 3.858809 | 0.045407 | -3.8134 | 9.82E-95 | 1.28E-93 |  |
| AC159540.2 | 3.817844 | 0.011727 | -3.80612 | 8.14E-95 | 1.09E-93 |  |
| CELF4 | 4.97466 | 1.168945 | -3.80571 | 3.99E-87 | 2.03E-86 |  |
| AC087292.1 | 3.794382 | 0 | -3.79438 | 5.02E-97 | 2.11E-95 |  |
| ABCC8 | 4.852883 | 1.063553 | -3.78933 | 2.21E-94 | 2.53E-93 |  |
| LINC01106 | 3.840513 | 0.059495 | -3.78102 | 1.30E-96 | 4.04E-95 |  |
| VSNL1 | 6.320274 | 2.54018 | -3.78009 | 3.58E-62 | 8.62E-62 |  |
| PSMC1P5 | 4.051093 | 0.289891 | -3.7612 | 5.36E-80 | 1.98E-79 |  |
| PDIA2 | 5.202701 | 1.447132 | -3.75557 | 3.89E-90 | 2.40E-89 |  |
| RBM34 | 4.599792 | 0.847925 | -3.75187 | 2.77E-95 | 4.55E-94 |  |
| CHKB | 5.055435 | 1.309677 | -3.74576 | 4.54E-95 | 6.78E-94 |  |
| VWA5B2 | 4.397175 | 0.662778 | -3.7344 | 1.09E-94 | 1.38E-93 |  |
| GP1BB | 3.732451 | 0 | -3.73245 | 5.96E-74 | 1.86E-73 |  |
| TUBA8 | 3.790842 | 0.063155 | -3.72769 | 2.43E-96 | 6.50E-95 |  |
| MUC20-OT1 | 4.70127 | 1.005801 | -3.69547 | 1.44E-94 | 1.76E-93 |  |
| SPTBN4 | 5.187943 | 1.495258 | -3.69268 | 7.56E-95 | 1.03E-93 |  |
| GUSBP11 | 3.891592 | 0.199904 | -3.69169 | 2.36E-95 | 4.01E-94 |  |
| ANKHD1 | 4.04556 | 0.354448 | -3.69111 | 4.01E-95 | 6.13E-94 |  |
| AC114546.3 | 3.667338 | 0 | -3.66734 | 2.21E-96 | 6.00E-95 |  |
| HSF4 | 5.268548 | 1.601844 | -3.6667 | 1.92E-93 | 1.73E-92 |  |
| UBE2V1 | 5.344054 | 1.677406 | -3.66665 | 3.68E-95 | 5.67E-94 |  |
| CBS | 4.45013 | 0.790152 | -3.65998 | 1.16E-94 | 1.46E-93 |  |
| RPSAP58 | 4.391593 | 0.73332 | -3.65827 | 4.96E-94 | 5.10E-93 |  |
| CKMT1A | 4.080345 | 0.425213 | -3.65513 | 6.08E-92 | 4.47E-91 |  |
| PRODH | 4.897521 | 1.244485 | -3.65304 | 1.84E-93 | 1.67E-92 |  |
| PHYHIP | 6.427106 | 2.775873 | -3.65123 | 6.31E-77 | 2.13E-76 |  |
| AC245595.1 | 4.240892 | 0.594808 | -3.64608 | 7.23E-93 | 5.94E-92 |  |
| UPK3BL1 | 4.229102 | 0.583035 | -3.64607 | 1.46E-94 | 1.78E-93 |  |
| RNU1-4 | 3.683335 | 0.045188 | -3.63815 | 9.96E-91 | 6.46E-90 |  |
| CERS6-AS1 | 3.640423 | 0.002868 | -3.63755 | 3.18E-96 | 8.07E-95 |  |
| AL583722.2 | 3.63258 | 0 | -3.63258 | 1.05E-96 | 3.43E-95 |  |
| SLX1A | 3.640579 | 0.011526 | -3.62905 | 5.08E-96 | 1.17E-94 |  |
| SNCB | 6.827797 | 3.201247 | -3.62655 | 2.51E-72 | 7.48E-72 |  |
| RNU1-3 | 3.677802 | 0.052334 | -3.62547 | 1.36E-90 | 8.75E-90 |  |
| FXYD7 | 5.65356 | 2.031576 | -3.62198 | 9.30E-73 | 2.80E-72 |  |
| AC073864.1 | 3.621435 | 0 | -3.62143 | 7.27E-97 | 2.68E-95 |  |
| TUBB3 | 5.990463 | 2.37433 | -3.61613 | 5.67E-94 | 5.72E-93 |  |
| AC126755.1 | 4.150423 | 0.539779 | -3.61064 | 1.07E-94 | 1.37E-93 |  |
| AMY2B | 4.649792 | 1.040332 | -3.60946 | 2.38E-95 | 4.03E-94 |  |
| YJEFN3 | 5.565675 | 1.958079 | -3.6076 | 2.79E-90 | 1.74E-89 |  |
| MSANTD3-TMEFF1 | 3.675842 | 0.073799 | -3.60204 | 3.22E-96 | 8.12E-95 |  |
| CRYM | 4.937632 | 1.336203 | -3.60143 | 1.88E-69 | 5.23E-69 |  |
| RNU1-1 | 3.678103 | 0.078469 | -3.59963 | 2.12E-90 | 1.33E-89 |  |
| AC009084.3 | 3.568329 | 0 | -3.56833 | 1.05E-96 | 3.43E-95 |  |
| HERC2P9 | 4.925313 | 1.358379 | -3.56693 | 5.50E-95 | 7.94E-94 |  |
| XBP1 | 3.559762 | 0 | -3.55976 | 5.02E-97 | 2.11E-95 |  |
| RPL32P29 | 3.543801 | 0 | -3.5438 | 5.02E-97 | 2.11E-95 |  |
| MYO15B | 5.009815 | 1.47162 | -3.53819 | 9.25E-95 | 1.21E-93 |  |
| RNVU1-18 | 3.678045 | 0.142501 | -3.53554 | 5.61E-89 | 3.18E-88 |  |
| AP001267.1 | 3.540129 | 0.004938 | -3.53519 | 7.85E-97 | 2.83E-95 |  |
| ECHDC2 | 5.658874 | 2.132421 | -3.52645 | 5.50E-94 | 5.56E-93 |  |
| RNU1-2 | 3.683479 | 0.163243 | -3.52024 | 1.15E-88 | 6.42E-88 |  |
| PIK3R2 | 4.023301 | 0.510833 | -3.51247 | 1.19E-94 | 1.49E-93 |  |
| GLS2 | 3.579531 | 0.068415 | -3.51112 | 2.94E-96 | 7.63E-95 |  |
| BCAS1 | 6.444614 | 2.9339 | -3.51071 | 3.14E-68 | 8.47E-68 |  |
| TMEM130 | 5.431065 | 1.920863 | -3.5102 | 7.52E-78 | 2.60E-77 |  |
| LUZP6 | 3.497948 | 0 | -3.49795 | 1.81E-79 | 6.60E-79 |  |
| HHATL | 5.726659 | 2.229926 | -3.49673 | 3.24E-81 | 1.25E-80 |  |
| GNG3 | 6.945617 | 3.450298 | -3.49532 | 1.90E-79 | 6.89E-79 |  |
| KIAA0408 | 3.530437 | 0.036808 | -3.49363 | 1.33E-96 | 4.11E-95 |  |
| CPT1B | 3.896561 | 0.404494 | -3.49207 | 4.19E-95 | 6.37E-94 |  |
| GARS-DT | 4.778521 | 1.28862 | -3.4899 | 6.73E-95 | 9.37E-94 |  |
| RNU1-27P | 3.683433 | 0.198947 | -3.48449 | 4.19E-87 | 2.13E-86 |  |
| HCG25 | 4.291251 | 0.808344 | -3.48291 | 6.57E-94 | 6.50E-93 |  |
| RNU1-28P | 3.677925 | 0.195052 | -3.48287 | 4.09E-87 | 2.08E-86 |  |
| PNCK | 4.805721 | 1.324541 | -3.48118 | 1.34E-92 | 1.05E-91 |  |
| SH3GL3 | 4.712084 | 1.236165 | -3.47592 | 5.01E-92 | 3.72E-91 |  |
| A1BG | 3.775591 | 0.311812 | -3.46378 | 5.99E-95 | 8.52E-94 |  |
| MICAL2 | 4.91848 | 1.455066 | -3.46341 | 1.03E-86 | 5.08E-86 |  |
| KCNT1 | 3.775429 | 0.314021 | -3.46141 | 2.92E-90 | 1.82E-89 |  |
| AK1 | 6.625176 | 3.172361 | -3.45282 | 1.69E-94 | 2.02E-93 |  |
| PLCH2 | 4.039506 | 0.602449 | -3.43706 | 4.94E-94 | 5.08E-93 |  |
| SNCA | 5.954315 | 2.522256 | -3.43206 | 1.27E-91 | 8.99E-91 |  |
| RBFOX1 | 4.159184 | 0.734143 | -3.42504 | 2.71E-86 | 1.31E-85 |  |
| NPM2 | 4.411755 | 0.987708 | -3.42405 | 4.24E-90 | 2.61E-89 |  |
| BBS1 | 4.116713 | 0.703184 | -3.41353 | 3.15E-97 | 1.79E-95 |  |
| CMC2 | 5.650031 | 2.239003 | -3.41103 | 5.63E-95 | 8.09E-94 |  |
| PTGDS | 9.832218 | 6.426488 | -3.40573 | 6.34E-86 | 3.00E-85 |  |
| AC138430.2 | 3.403526 | 0 | -3.40353 | 2.21E-96 | 6.00E-95 |  |
| RGS11 | 5.412794 | 2.014077 | -3.39872 | 4.56E-93 | 3.86E-92 |  |
| CAMK2B | 6.195413 | 2.80092 | -3.39449 | 3.90E-82 | 1.56E-81 |  |
| ANKS1B | 4.805519 | 1.420689 | -3.38483 | 5.43E-95 | 7.85E-94 |  |
| PGAM2 | 3.378505 | 0 | -3.3785 | 5.02E-97 | 2.11E-95 |  |
| DOC2A | 4.392416 | 1.016098 | -3.37632 | 2.09E-85 | 9.64E-85 |  |
| ATP6V1G2-DDX39B | 3.380495 | 0.006833 | -3.37366 | 7.95E-97 | 2.85E-95 |  |
| OVCA2 | 3.368696 | 0 | -3.3687 | 5.02E-97 | 2.11E-95 |  |
| CACNA1A | 4.792435 | 1.426102 | -3.36633 | 5.29E-92 | 3.91E-91 |  |
| MT-ATP8 | 15.07887 | 11.71984 | -3.35903 | ###### | ###### |  |
| SYNGR3 | 5.189142 | 1.838464 | -3.35068 | 1.53E-83 | 6.51E-83 |  |
| NEAT1 | 6.824889 | 3.474533 | -3.35036 | 1.78E-90 | 1.13E-89 |  |
| KCNA1 | 3.719985 | 0.382465 | -3.33752 | 7.44E-90 | 4.48E-89 |  |
| CBWD3 | 3.457114 | 0.119731 | -3.33738 | 1.30E-94 | 1.60E-93 |  |
| RIMS2 | 3.741117 | 0.408532 | -3.33259 | 1.27E-92 | 1.00E-91 |  |
| BICDL1 | 3.929457 | 0.597911 | -3.33155 | 1.07E-93 | 1.01E-92 |  |
| PVALB | 4.046255 | 0.717495 | -3.32876 | 2.04E-60 | 4.76E-60 |  |
| MUSTN1 | 3.374749 | 0.057278 | -3.31747 | 5.29E-95 | 7.68E-94 |  |
| FBXL16 | 7.053943 | 3.738079 | -3.31586 | 2.72E-72 | 8.08E-72 |  |
| GET4 | 4.409994 | 1.095144 | -3.31485 | 3.87E-95 | 5.94E-94 |  |
| SCN1B | 5.918555 | 2.603997 | -3.31456 | 1.17E-92 | 9.27E-92 |  |
| NSG2 | 6.292032 | 2.980459 | -3.31157 | 2.27E-68 | 6.16E-68 |  |
| SPTBN2 | 5.551643 | 2.241285 | -3.31036 | 2.25E-82 | 9.08E-82 |  |
| RASA4B | 3.346278 | 0.037578 | -3.3087 | 1.21E-95 | 2.41E-94 |  |
| RAB26 | 5.150268 | 1.842759 | -3.30751 | 1.38E-85 | 6.44E-85 |  |
| EEF1D | 7.20185 | 3.89555 | -3.3063 | 3.23E-94 | 3.53E-93 |  |
| AC010503.2 | 3.304687 | 0 | -3.30469 | 2.92E-95 | 4.75E-94 |  |
| WNK2 | 4.28009 | 0.986006 | -3.29408 | 8.40E-93 | 6.82E-92 |  |
| TPD52L1 | 5.026893 | 1.738072 | -3.28882 | 2.67E-76 | 8.86E-76 |  |
| NDUFV2 | 5.858337 | 2.570784 | -3.28755 | 1.02E-94 | 1.31E-93 |  |
| DMRTC1B | 3.300388 | 0.012937 | -3.28745 | 1.42E-96 | 4.29E-95 |  |
| AARSD1 | 4.78849 | 1.506759 | -3.28173 | 1.94E-95 | 3.44E-94 |  |
| AC015688.6 | 3.280433 | 0 | -3.28043 | 7.27E-97 | 2.68E-95 |  |
| SULT4A1 | 5.061719 | 1.787053 | -3.27467 | 8.05E-74 | 2.49E-73 |  |
| NDUFB8 | 7.602951 | 4.341323 | -3.26163 | 1.48E-95 | 2.79E-94 |  |
| PLEKHH1 | 5.438405 | 2.178188 | -3.26022 | 2.18E-90 | 1.37E-89 |  |
| AC012615.3 | 3.257938 | 5.60E-05 | -3.25788 | 5.00E-96 | 1.15E-94 |  |
| PPP1R1A | 4.533902 | 1.276298 | -3.2576 | 5.70E-81 | 2.18E-80 |  |
| OPALIN | 4.279442 | 1.028152 | -3.25129 | 2.09E-69 | 5.80E-69 |  |
| PRRT1 | 5.467076 | 2.217987 | -3.24909 | 2.32E-94 | 2.64E-93 |  |
| RTN1 | 7.615606 | 4.375086 | -3.24052 | 1.09E-82 | 4.46E-82 |  |
| EVL | 7.012191 | 3.777137 | -3.23505 | 1.68E-95 | 3.06E-94 |  |
| HAUS7 | 4.358591 | 1.127962 | -3.23063 | 2.34E-94 | 2.66E-93 |  |
| FAM156A | 3.355839 | 0.126826 | -3.22901 | 3.13E-95 | 5.00E-94 |  |
| ASPDH | 4.583949 | 1.355085 | -3.22886 | 2.92E-93 | 2.54E-92 |  |
| PLA2G4B | 3.382871 | 0.156506 | -3.22636 | 1.83E-95 | 3.28E-94 |  |
| SNX15 | 3.589716 | 0.364445 | -3.22527 | 1.19E-94 | 1.49E-93 |  |
| PKD1 | 6.470977 | 3.252831 | -3.21815 | 4.70E-91 | 3.13E-90 |  |
| RPL37A | 10.28219 | 7.070677 | -3.21151 | 3.80E-94 | 4.04E-93 |  |
| NPIPA5 | 4.83338 | 1.6281 | -3.20528 | 8.59E-86 | 4.05E-85 |  |
| CYP46A1 | 4.809307 | 1.605974 | -3.20333 | 1.05E-87 | 5.50E-87 |  |
| UQCRB | 7.598642 | 4.416765 | -3.18188 | 2.18E-95 | 3.78E-94 |  |
| ZDHHC11B | 4.397599 | 1.21799 | -3.17961 | 8.23E-95 | 1.10E-93 |  |
| AL390726.5 | 3.289732 | 0.110232 | -3.1795 | 8.62E-95 | 1.14E-93 |  |
| MICOS10 | 6.414423 | 3.239229 | -3.17519 | 4.42E-95 | 6.62E-94 |  |
| KCNIP2 | 5.19556 | 2.023432 | -3.17213 | 5.28E-82 | 2.10E-81 |  |
| TNFRSF25 | 4.65111 | 1.479072 | -3.17204 | 1.73E-88 | 9.53E-88 |  |
| PLGLB1 | 3.262367 | 0.090463 | -3.1719 | 2.35E-95 | 4.00E-94 |  |
| C2orf74 | 4.991366 | 1.82117 | -3.1702 | 1.33E-95 | 2.58E-94 |  |
| AGAP3 | 7.105267 | 3.938005 | -3.16726 | 1.19E-95 | 2.39E-94 |  |
| MYT1L | 3.671976 | 0.505678 | -3.1663 | 4.88E-87 | 2.47E-86 |  |
| AL162231.1 | 4.574034 | 1.419637 | -3.1544 | 1.02E-93 | 9.70E-93 |  |
| AP003108.2 | 3.323321 | 0.171061 | -3.15226 | 7.23E-95 | 9.94E-94 |  |
| AL138478.1 | 3.149752 | 0 | -3.14975 | 5.02E-97 | 2.11E-95 |  |
| AL732372.2 | 3.196636 | 0.046975 | -3.14966 | 2.13E-96 | 5.91E-95 |  |
| NEIL1 | 4.882036 | 1.732799 | -3.14924 | 6.44E-97 | 2.48E-95 |  |
| EIF3CL | 4.145674 | 0.997009 | -3.14867 | 1.07E-93 | 1.01E-92 |  |
| CPLX1 | 5.866913 | 2.719254 | -3.14766 | 1.78E-77 | 6.06E-77 |  |
| CBWD6 | 3.474122 | 0.327431 | -3.14669 | 1.14E-94 | 1.43E-93 |  |
| FBXO2 | 6.513387 | 3.3746 | -3.13879 | 1.70E-90 | 1.08E-89 |  |
| AATK | 5.507158 | 2.369881 | -3.13728 | 2.80E-88 | 1.52E-87 |  |
| SYT5 | 4.585908 | 1.451382 | -3.13453 | 3.15E-63 | 7.74E-63 |  |
| AC008894.2 | 3.147873 | 0.013849 | -3.13402 | 1.38E-96 | 4.20E-95 |  |
| AP3B2 | 5.320832 | 2.187364 | -3.13347 | 1.11E-92 | 8.84E-92 |  |
| RGPD6 | 3.126326 | 0.002853 | -3.12347 | 1.33E-96 | 4.12E-95 |  |
| RPPH1 | 3.122122 | 0 | -3.12212 | 1.53E-96 | 4.54E-95 |  |
| NRXN3 | 3.836836 | 0.716825 | -3.12001 | 4.07E-93 | 3.47E-92 |  |
| DNLZ | 3.265752 | 0.145877 | -3.11987 | 3.32E-95 | 5.24E-94 |  |
| TBC1D3L | 3.373232 | 0.256104 | -3.11713 | 2.43E-71 | 7.05E-71 |  |
| RAPGEF4 | 5.392452 | 2.280681 | -3.11177 | 6.85E-84 | 2.96E-83 |  |
| LPCAT4 | 5.851366 | 2.739715 | -3.11165 | 1.18E-92 | 9.38E-92 |  |
| MACF1 | 6.209556 | 3.09887 | -3.11069 | 1.37E-95 | 2.62E-94 |  |
| AL445363.3 | 3.338973 | 0.228335 | -3.11064 | 3.35E-94 | 3.63E-93 |  |
| DDX47 | 3.824017 | 0.714309 | -3.10971 | 1.88E-94 | 2.21E-93 |  |
| NDUFA7 | 5.519786 | 2.41617 | -3.10362 | 1.30E-93 | 1.21E-92 |  |
| RAPGEF3 | 4.37989 | 1.276824 | -3.10307 | 2.82E-93 | 2.47E-92 |  |
| FAM153A | 3.142386 | 0.03997 | -3.10242 | 2.89E-96 | 7.55E-95 |  |
| DNM1 | 6.646933 | 3.546268 | -3.10066 | 1.02E-71 | 2.99E-71 |  |
| SLX1A-SULT1A3 | 3.124153 | 0.024674 | -3.09948 | 3.90E-96 | 9.49E-95 |  |
| AL355816.2 | 3.098008 | 0 | -3.09801 | 2.21E-96 | 6.00E-95 |  |
| UNC13C | 3.251362 | 0.153833 | -3.09753 | 8.10E-94 | 7.88E-93 |  |
| DMKN | 3.364345 | 0.267852 | -3.09649 | 5.00E-96 | 1.15E-94 |  |
| RPL23AP42 | 7.851783 | 4.759209 | -3.09257 | 4.90E-94 | 5.06E-93 |  |
| NACA | 8.91913 | 5.829551 | -3.08958 | 5.21E-96 | 1.19E-94 |  |
| HPCA | 6.085882 | 3.000068 | -3.08581 | 1.21E-58 | 2.73E-58 |  |
| AC090984.1 | 3.0819 | 0 | -3.0819 | 2.21E-96 | 6.00E-95 |  |
| DNAJA4 | 5.377534 | 2.295727 | -3.08181 | 1.84E-97 | 1.61E-95 |  |
| BRSK2 | 5.281964 | 2.210624 | -3.07134 | 2.08E-93 | 1.86E-92 |  |
| AC010336.3 | 3.065213 | 0 | -3.06521 | 9.69E-96 | 2.02E-94 |  |
| NEURL1 | 4.23432 | 1.175243 | -3.05908 | 8.71E-85 | 3.91E-84 |  |
| COX20 | 4.96147 | 1.902474 | -3.059 | 1.36E-93 | 1.26E-92 |  |
| IRF9 | 5.098363 | 2.043314 | -3.05505 | 1.18E-93 | 1.11E-92 |  |
| GJB6 | 3.840703 | 0.793309 | -3.04739 | 8.48E-72 | 2.49E-71 |  |
| RAB11FIP4 | 4.465892 | 1.419597 | -3.0463 | 1.68E-93 | 1.54E-92 |  |
| PRCD | 3.471542 | 0.426868 | -3.04467 | 9.16E-95 | 1.20E-93 |  |
| 5-Sep | 7.165383 | 4.123133 | -3.04225 | 9.40E-79 | 3.34E-78 |  |
| ACAD11 | 3.213074 | 0.172415 | -3.04066 | 5.06E-95 | 7.40E-94 |  |
| PTPN5 | 4.30505 | 1.266762 | -3.03829 | 1.79E-56 | 3.91E-56 |  |
| MAN2C1 | 6.253561 | 3.220729 | -3.03283 | 7.56E-95 | 1.03E-93 |  |
| PDE4DIP | 5.902916 | 2.872403 | -3.03051 | 3.28E-97 | 1.82E-95 |  |
| CHD5 | 3.808421 | 0.779217 | -3.0292 | 4.90E-80 | 1.81E-79 |  |
| CBX7 | 5.315411 | 2.287295 | -3.02812 | 7.46E-95 | 1.02E-93 |  |
| AC011462.2 | 3.028797 | 0.00182 | -3.02698 | 3.93E-97 | 1.95E-95 |  |
| MRPL38 | 5.226788 | 2.199845 | -3.02694 | 8.68E-95 | 1.15E-93 |  |
| EGLN2 | 5.285794 | 2.259943 | -3.02585 | 2.39E-95 | 4.05E-94 |  |
| GALNT9 | 3.879924 | 0.857244 | -3.02268 | 1.23E-72 | 3.71E-72 |  |
| OGDHL | 4.34107 | 1.321059 | -3.02001 | 4.96E-87 | 2.51E-86 |  |
| HIST2H2AA3 | 3.468476 | 0.450943 | -3.01753 | 1.05E-83 | 4.51E-83 |  |
| PRKCG | 3.881058 | 0.878835 | -3.00222 | 1.31E-73 | 4.04E-73 |  |
| DMTN | 6.301141 | 3.301008 | -3.00013 | 5.58E-83 | 2.32E-82 |  |
| PTPRN | 5.50089 | 2.500898 | -2.99999 | 1.47E-74 | 4.65E-74 |  |
| HRH3 | 3.560796 | 0.563115 | -2.99768 | 6.12E-83 | 2.53E-82 |  |
| FGF13 | 3.408172 | 0.41108 | -2.99709 | 1.41E-69 | 3.92E-69 |  |
| SRCIN1 | 4.657514 | 1.660527 | -2.99699 | 2.09E-90 | 1.32E-89 |  |
| RPL31 | 9.787109 | 6.79186 | -2.99525 | 1.59E-93 | 1.46E-92 |  |
| VASH1-AS1 | 3.858447 | 0.864218 | -2.99423 | 6.85E-95 | 9.51E-94 |  |
| DGCR6 | 4.66544 | 1.672814 | -2.99263 | 1.46E-94 | 1.78E-93 |  |
| TTC9B | 5.09643 | 2.10544 | -2.99099 | 2.80E-74 | 8.81E-74 |  |
| CASKIN1 | 4.635614 | 1.650658 | -2.98496 | 2.33E-85 | 1.07E-84 |  |
| ADORA2A | 3.018044 | 0.033234 | -2.98481 | 1.17E-96 | 3.71E-95 |  |
| AC008264.2 | 2.999182 | 0.021641 | -2.97754 | 3.46E-96 | 8.58E-95 |  |
| SNAP91 | 4.617475 | 1.6487 | -2.96877 | 1.64E-79 | 5.99E-79 |  |
| MTCP1 | 3.509289 | 0.546081 | -2.96321 | 2.47E-94 | 2.79E-93 |  |
| KIFC2 | 5.963909 | 3.003252 | -2.96066 | 1.13E-83 | 4.84E-83 |  |
| SULT1A1 | 4.264736 | 1.3044 | -2.96034 | 7.30E-93 | 5.99E-92 |  |
| PDE1B | 4.255975 | 1.295678 | -2.9603 | 4.21E-82 | 1.68E-81 |  |
| NDUFA11 | 6.917074 | 3.957139 | -2.95993 | 2.34E-93 | 2.08E-92 |  |
| GSTM2 | 5.95116 | 2.99157 | -2.95959 | 5.38E-94 | 5.47E-93 |  |
| RAB40B | 5.25465 | 2.295949 | -2.9587 | 2.39E-94 | 2.71E-93 |  |
| AL157392.3 | 3.586331 | 0.628038 | -2.95829 | 1.33E-95 | 2.58E-94 |  |
| DDN | 4.393087 | 1.435022 | -2.95806 | 1.76E-24 | 2.54E-24 |  |
| PRRT2 | 5.742752 | 2.785061 | -2.95769 | 4.74E-76 | 1.56E-75 |  |
| PDLIM2 | 4.729152 | 1.778474 | -2.95068 | 8.02E-94 | 7.82E-93 |  |
| AC105052.3 | 3.251799 | 0.305429 | -2.94637 | 5.77E-89 | 3.27E-88 |  |
| SOWAHA | 4.334506 | 1.389066 | -2.94544 | 9.08E-84 | 3.89E-83 |  |
| AF111169.1 | 2.94527 | 0 | -2.94527 | 7.27E-97 | 2.68E-95 |  |
| CELF3 | 5.054602 | 2.110139 | -2.94446 | 7.58E-72 | 2.23E-71 |  |
| RIMS1 | 3.363445 | 0.420902 | -2.94254 | 3.43E-91 | 2.32E-90 |  |
| AL391807.1 | 5.186411 | 2.24629 | -2.94012 | 5.12E-94 | 5.24E-93 |  |
| RELN | 3.274558 | 0.334958 | -2.9396 | 2.12E-89 | 1.24E-88 |  |
| AC008581.1 | 2.936049 | 0 | -2.93605 | 5.02E-97 | 2.11E-95 |  |
| MCF2L2 | 3.184134 | 0.249949 | -2.93419 | 9.46E-96 | 2.00E-94 |  |
| EPHB6 | 4.835646 | 1.90434 | -2.93131 | 6.51E-70 | 1.83E-69 |  |
| ETNPPL | 5.482504 | 2.554767 | -2.92774 | 1.72E-59 | 3.95E-59 |  |
| SLC25A27 | 4.776201 | 1.848555 | -2.92765 | 4.52E-95 | 6.76E-94 |  |
| CELF5 | 4.554272 | 1.626764 | -2.92751 | 4.34E-76 | 1.43E-75 |  |
| RALYL | 3.913352 | 0.985905 | -2.92745 | 4.88E-87 | 2.47E-86 |  |
| PHACTR3 | 5.045538 | 2.11839 | -2.92715 | 1.02E-92 | 8.17E-92 |  |
| LINC00632 | 3.487535 | 0.562074 | -2.92546 | 1.18E-94 | 1.48E-93 |  |
| CDK11A | 4.40707 | 1.482758 | -2.92431 | 1.91E-94 | 2.24E-93 |  |
| AC139256.2 | 3.755292 | 0.834173 | -2.92112 | 1.08E-94 | 1.38E-93 |  |
| HERC2P2 | 5.523051 | 2.603816 | -2.91924 | 1.93E-88 | 1.06E-87 |  |
| SELENOM | 6.313455 | 3.397995 | -2.91546 | 5.09E-91 | 3.38E-90 |  |
| TCEAL6 | 4.899444 | 1.987492 | -2.91195 | 3.22E-83 | 1.35E-82 |  |
| ABHD16A | 4.704417 | 1.792647 | -2.91177 | 4.32E-95 | 6.51E-94 |  |
| AF127577.1 | 2.91079 | 0 | -2.91079 | 2.21E-96 | 6.00E-95 |  |
| CALB1 | 3.387251 | 0.478813 | -2.90844 | 9.35E-81 | 3.54E-80 |  |
| TMSB4XP6 | 2.905523 | 0 | -2.90552 | 1.78E-50 | 3.56E-50 |  |
| RBM4 | 5.219128 | 2.313837 | -2.90529 | 1.39E-94 | 1.70E-93 |  |
| CD22 | 3.45164 | 0.546531 | -2.90511 | 8.58E-87 | 4.27E-86 |  |
| GARNL3 | 4.059608 | 1.155951 | -2.90366 | 1.34E-95 | 2.59E-94 |  |
| C1QTNF5 | 2.902317 | 0 | -2.90232 | 5.02E-97 | 2.11E-95 |  |
| PILRB | 4.124417 | 1.231494 | -2.89292 | 2.58E-86 | 1.25E-85 |  |
| AC015813.2 | 2.932144 | 0.039728 | -2.89242 | 1.95E-95 | 3.44E-94 |  |
| NDRG2 | 9.376559 | 6.48483 | -2.89173 | 7.56E-88 | 3.98E-87 |  |
| WASH6P | 4.512061 | 1.621039 | -2.89102 | 2.83E-94 | 3.15E-93 |  |
| ZNF638 | 5.875921 | 2.985247 | -2.89067 | 1.35E-95 | 2.60E-94 |  |
| GRM4 | 2.920754 | 0.034589 | -2.88617 | 1.40E-95 | 2.67E-94 |  |
| KRTCAP2 | 5.808135 | 2.924792 | -2.88334 | 4.52E-94 | 4.70E-93 |  |
| SYCE1 | 2.90127 | 0.018443 | -2.88283 | 2.50E-97 | 1.71E-95 |  |
| AP003419.1 | 2.892278 | 0.010143 | -2.88213 | 3.13E-93 | 2.71E-92 |  |
| PENK | 3.659693 | 0.778744 | -2.88095 | 5.61E-58 | 1.26E-57 |  |
| DLG2 | 4.117908 | 1.237102 | -2.88081 | 1.33E-93 | 1.24E-92 |  |
| GRIN2C | 3.757596 | 0.876894 | -2.8807 | 7.23E-83 | 2.98E-82 |  |
| HECTD4 | 4.932146 | 2.057894 | -2.87425 | 2.30E-95 | 3.93E-94 |  |
| PTRH1 | 2.917183 | 0.044599 | -2.87258 | 2.46E-96 | 6.56E-95 |  |
| DNAJC25-GNG10 | 2.872109 | 0.000888 | -2.87122 | 1.14E-96 | 3.64E-95 |  |
| TNFRSF6B | 2.868968 | 0 | -2.86897 | 2.07E-88 | 1.14E-87 |  |
| ARHGAP44 | 3.478108 | 0.611222 | -2.86689 | 1.80E-92 | 1.40E-91 |  |
| PAK6 | 2.912826 | 0.050845 | -2.86198 | 6.38E-94 | 6.36E-93 |  |
| TAF1D | 5.769242 | 2.917927 | -2.85132 | 3.44E-95 | 5.36E-94 |  |
| PHLDB1 | 5.922297 | 3.072026 | -2.85027 | 5.73E-95 | 8.20E-94 |  |
| ABCA2 | 7.129513 | 4.283336 | -2.84618 | 2.27E-89 | 1.33E-88 |  |
| SLC1A6 | 3.091851 | 0.246389 | -2.84546 | 4.20E-90 | 2.58E-89 |  |
| BEGAIN | 4.068071 | 1.222766 | -2.84531 | 1.42E-88 | 7.87E-88 |  |
| GOLGA8A | 5.130065 | 2.290052 | -2.84001 | 1.37E-83 | 5.85E-83 |  |
| ALKBH6 | 3.86175 | 1.021928 | -2.83982 | 2.31E-95 | 3.94E-94 |  |
| AL031600.3 | 2.839254 | 0 | -2.83925 | 1.75E-91 | 1.22E-90 |  |
| TPT1-AS1 | 4.210404 | 1.373834 | -2.83657 | 9.89E-95 | 1.28E-93 |  |
| RNF212 | 3.071387 | 0.235039 | -2.83635 | 9.44E-92 | 6.78E-91 |  |
| EGFL8 | 3.868354 | 1.03238 | -2.83597 | 1.18E-94 | 1.48E-93 |  |
| HID1 | 5.177085 | 2.343765 | -2.83332 | 4.54E-94 | 4.72E-93 |  |
| JPH4 | 5.670136 | 2.836971 | -2.83316 | 2.61E-72 | 7.78E-72 |  |
| NDRG4 | 7.683959 | 4.851268 | -2.83269 | 2.07E-83 | 8.75E-83 |  |
| CMC4 | 2.83089 | 0.000472 | -2.83042 | 6.37E-97 | 2.45E-95 |  |
| HAPLN2 | 5.562148 | 2.733544 | -2.8286 | 1.93E-67 | 5.14E-67 |  |
| NSUN5P2 | 3.972341 | 1.145723 | -2.82662 | 3.45E-94 | 3.73E-93 |  |
| TMEM179 | 4.562211 | 1.736444 | -2.82577 | 1.59E-84 | 7.05E-84 |  |
| CLK3 | 4.638699 | 1.813035 | -2.82566 | 1.29E-94 | 1.59E-93 |  |
| WSCD2 | 3.144025 | 0.320928 | -2.8231 | 6.90E-87 | 3.45E-86 |  |
| CELF6 | 2.830322 | 0.008554 | -2.82177 | 1.77E-97 | 1.61E-95 |  |
| MYCBP2 | 4.967126 | 2.148442 | -2.81868 | 3.01E-95 | 4.86E-94 |  |
| FAR2P2 | 3.588908 | 0.771379 | -2.81753 | 1.41E-94 | 1.73E-93 |  |
| CCZ1B | 4.719239 | 1.903586 | -2.81565 | 4.48E-94 | 4.67E-93 |  |
| STRC | 2.82038 | 0.005741 | -2.81464 | 1.57E-97 | 1.61E-95 |  |
| SFI1 | 4.319521 | 1.50584 | -2.81368 | 6.02E-95 | 8.55E-94 |  |
| DOCK9 | 4.655854 | 1.842665 | -2.81319 | 1.81E-95 | 3.26E-94 |  |
| RASGRF1 | 3.509624 | 0.696892 | -2.81273 | 2.59E-82 | 1.04E-81 |  |
| AC125494.1 | 2.846566 | 0.034581 | -2.81198 | 9.29E-96 | 1.97E-94 |  |
| SDR39U1 | 5.464113 | 2.655245 | -2.80887 | 1.06E-94 | 1.35E-93 |  |
| ALDH2 | 6.82692 | 4.018271 | -2.80865 | 3.80E-92 | 2.85E-91 |  |
| PAIP2B | 4.277198 | 1.472899 | -2.8043 | 3.53E-86 | 1.70E-85 |  |
| SYT1 | 5.318657 | 2.515044 | -2.80361 | 7.56E-56 | 1.64E-55 |  |
| INA | 4.828386 | 2.031121 | -2.79727 | 1.63E-62 | 3.96E-62 |  |
| GSTM5 | 4.302611 | 1.519808 | -2.7828 | 1.72E-72 | 5.16E-72 |  |
| LGI1 | 4.317824 | 1.537583 | -2.78024 | 5.67E-88 | 3.01E-87 |  |
| OBSCN | 2.977692 | 0.197812 | -2.77988 | 5.81E-95 | 8.28E-94 |  |
| CEMP1 | 2.779663 | 0 | -2.77966 | 5.02E-97 | 2.11E-95 |  |
| SEMA4D | 4.858068 | 2.079239 | -2.77883 | 5.08E-93 | 4.26E-92 |  |
| FGF17 | 3.228819 | 0.450357 | -2.77846 | 1.93E-86 | 9.40E-86 |  |
| PSMA6 | 5.399212 | 2.621244 | -2.77797 | 1.21E-94 | 1.50E-93 |  |
| OLFM1 | 7.070079 | 4.296739 | -2.77334 | 5.54E-71 | 1.60E-70 |  |
| YPEL4 | 4.132649 | 1.359698 | -2.77295 | 9.29E-94 | 8.96E-93 |  |
| TMEM191A | 3.000706 | 0.228532 | -2.77217 | 1.19E-94 | 1.49E-93 |  |
| SHANK1 | 3.826313 | 1.059231 | -2.76708 | 5.81E-76 | 1.90E-75 |  |
| GPS2 | 5.204392 | 2.437517 | -2.76688 | 2.43E-94 | 2.75E-93 |  |
| AP001107.2 | 2.765221 | 0 | -2.76522 | 3.03E-90 | 1.88E-89 |  |
| TSPYL2 | 6.572713 | 3.808792 | -2.76392 | 2.63E-90 | 1.64E-89 |  |
| PPP1R3E | 4.674035 | 1.911398 | -2.76264 | 1.67E-95 | 3.05E-94 |  |
| CHGB | 5.113226 | 2.352928 | -2.7603 | 2.13E-55 | 4.57E-55 |  |
| CALB2 | 4.343823 | 1.583579 | -2.76024 | 1.79E-66 | 4.65E-66 |  |
| AC100786.2 | 2.800836 | 0.040912 | -2.75992 | 1.33E-76 | 4.44E-76 |  |
| AC005523.2 | 2.758686 | 0 | -2.75869 | 5.02E-97 | 2.11E-95 |  |
| GOLGA8B | 4.713421 | 1.95729 | -2.75613 | 1.17E-85 | 5.45E-85 |  |
| PPFIA4 | 4.497049 | 1.743809 | -2.75324 | 2.05E-76 | 6.82E-76 |  |
| DTNB | 4.730242 | 1.982122 | -2.74812 | 1.32E-95 | 2.57E-94 |  |
| CAMK2A | 5.218145 | 2.470999 | -2.74715 | 2.64E-38 | 4.50E-38 |  |
| MRPS24 | 5.568761 | 2.823872 | -2.74489 | 2.74E-94 | 3.07E-93 |  |
| GOLGA2P7 | 2.98505 | 0.240181 | -2.74487 | 1.12E-94 | 1.41E-93 |  |
| MATK | 3.598934 | 0.855127 | -2.74381 | 7.22E-79 | 2.58E-78 |  |
| HAGHL | 4.690097 | 1.94662 | -2.74348 | 1.37E-93 | 1.27E-92 |  |
| NOMO3 | 3.506829 | 0.764853 | -2.74198 | 1.50E-94 | 1.83E-93 |  |
| UBR4 | 5.558971 | 2.821282 | -2.73769 | 1.27E-95 | 2.50E-94 |  |
| RF00002 | 2.841589 | 0.105404 | -2.73619 | 7.50E-90 | 4.52E-89 |  |
| SCHIP1 | 4.857337 | 2.121289 | -2.73605 | 8.31E-95 | 1.11E-93 |  |
| ZNF23 | 3.037031 | 0.302145 | -2.73489 | 1.89E-95 | 3.35E-94 |  |
| KIF5A | 7.100068 | 4.366266 | -2.7338 | 2.41E-64 | 6.04E-64 |  |
| NGRN | 6.59923 | 3.867264 | -2.73197 | 1.89E-95 | 3.35E-94 |  |
| CLEC2L | 3.383615 | 0.657679 | -2.72594 | 5.73E-85 | 2.60E-84 |  |
| ZDHHC11 | 3.30131 | 0.577047 | -2.72426 | 1.45E-96 | 4.36E-95 |  |
| DBP | 4.660437 | 1.93779 | -2.72265 | 1.48E-94 | 1.80E-93 |  |
| PKP4 | 5.980219 | 3.264749 | -2.71547 | 2.49E-93 | 2.20E-92 |  |
| SRXN1 | 3.480313 | 0.766088 | -2.71422 | 3.92E-95 | 6.01E-94 |  |
| CCDC57 | 4.353188 | 1.639383 | -2.71381 | 6.88E-95 | 9.53E-94 |  |
| ZNF410 | 3.428511 | 0.715725 | -2.71279 | 2.61E-94 | 2.94E-93 |  |
| ABLIM2 | 4.189372 | 1.477551 | -2.71182 | 2.02E-92 | 1.56E-91 |  |
| MICAL3 | 3.787069 | 1.075488 | -2.71158 | 2.62E-95 | 4.36E-94 |  |
| AK5 | 4.375179 | 1.663979 | -2.7112 | 1.94E-57 | 4.30E-57 |  |
| KCNAB2 | 5.256698 | 2.54628 | -2.71042 | 4.46E-83 | 1.86E-82 |  |
| JAKMIP1 | 4.008626 | 1.298865 | -2.70976 | 1.49E-77 | 5.11E-77 |  |
| CNTNAP4 | 3.882916 | 1.174804 | -2.70811 | 4.15E-82 | 1.66E-81 |  |
| ASPHD1 | 5.816672 | 3.109738 | -2.70693 | 1.74E-94 | 2.08E-93 |  |
| CDRT4 | 2.807815 | 0.102862 | -2.70495 | 7.56E-96 | 1.63E-94 |  |
| PPFIA3 | 4.780306 | 2.076435 | -2.70387 | 5.17E-92 | 3.83E-91 |  |
| TMEM151B | 4.156838 | 1.454261 | -2.70258 | 6.33E-71 | 1.82E-70 |  |
| KCNJ9 | 4.551978 | 1.849871 | -2.70211 | 1.57E-80 | 5.89E-80 |  |
| SYP | 6.594153 | 3.895829 | -2.69832 | 2.48E-76 | 8.23E-76 |  |
| SPINT2 | 5.063228 | 2.365422 | -2.69781 | 1.89E-92 | 1.46E-91 |  |
| STMN2 | 6.6007 | 3.903223 | -2.69748 | 7.88E-49 | 1.54E-48 |  |
| NDUFS7 | 6.300514 | 3.607716 | -2.6928 | 6.56E-94 | 6.50E-93 |  |
| AP003064.1 | 2.688102 | 0 | -2.6881 | 4.33E-77 | 1.46E-76 |  |
| TUBB4A | 7.97271 | 5.287637 | -2.68507 | 8.17E-58 | 1.82E-57 |  |
| EPB41L3 | 5.142222 | 2.457727 | -2.6845 | 2.60E-89 | 1.51E-88 |  |
| CCPG1 | 4.387041 | 1.703049 | -2.68399 | 3.04E-95 | 4.90E-94 |  |
| SCRT1 | 3.79359 | 1.109876 | -2.68371 | 8.79E-74 | 2.72E-73 |  |
| AL157935.2 | 3.212698 | 0.532382 | -2.68032 | 3.86E-94 | 4.09E-93 |  |
| RYR2 | 2.83672 | 0.156692 | -2.68003 | 2.62E-93 | 2.31E-92 |  |
| AL023284.4 | 4.222878 | 1.542969 | -2.67991 | 8.51E-90 | 5.10E-89 |  |
| GABARAP | 8.289432 | 5.610669 | -2.67876 | 2.33E-94 | 2.65E-93 |  |
| CADPS | 4.684087 | 2.007257 | -2.67683 | 6.97E-84 | 3.01E-83 |  |
| JMJD7 | 2.712253 | 0.037885 | -2.67437 | 3.16E-96 | 8.03E-95 |  |
| AC245297.2 | 3.191715 | 0.525523 | -2.66619 | 4.03E-94 | 4.24E-93 |  |
| GTF2IP4 | 5.834376 | 3.169599 | -2.66478 | 2.14E-94 | 2.46E-93 |  |
| SH3BP5 | 4.175653 | 1.5109 | -2.66475 | 2.88E-93 | 2.51E-92 |  |
| USP34 | 4.730262 | 2.066057 | -2.66421 | 1.60E-95 | 2.94E-94 |  |
| STXBP1 | 6.660811 | 3.998152 | -2.66266 | 8.46E-80 | 3.11E-79 |  |
| C1QTNF4 | 4.18232 | 1.521817 | -2.6605 | 1.01E-81 | 3.97E-81 |  |
| TIMP3 | 4.375446 | 1.71636 | -2.65909 | 1.93E-86 | 9.40E-86 |  |
| SKP1 | 8.214811 | 5.557454 | -2.65736 | 6.92E-95 | 9.57E-94 |  |
| AC127459.3 | 2.655837 | 0 | -2.65584 | 7.27E-97 | 2.68E-95 |  |
| SLC8A2 | 3.696763 | 1.04144 | -2.65532 | 1.85E-69 | 5.15E-69 |  |
| LAT | 2.78979 | 0.134805 | -2.65498 | 4.48E-95 | 6.71E-94 |  |
| PSMC1 | 5.303379 | 2.651752 | -2.65163 | 2.22E-94 | 2.54E-93 |  |
| CA11 | 7.373936 | 4.723009 | -2.65093 | 1.76E-79 | 6.39E-79 |  |
| TPPP | 5.674479 | 3.023689 | -2.65079 | 1.50E-79 | 5.48E-79 |  |
| TOP3B | 2.690702 | 0.044412 | -2.64629 | 4.57E-96 | 1.07E-94 |  |
| PCSK7 | 3.906425 | 1.260581 | -2.64584 | 4.64E-95 | 6.90E-94 |  |
| BDH1 | 4.573541 | 1.92779 | -2.64575 | 6.14E-95 | 8.66E-94 |  |
| PLEKHA1 | 3.669888 | 1.025481 | -2.64441 | 1.15E-95 | 2.34E-94 |  |
| SERPINI1 | 5.765188 | 3.122135 | -2.64305 | 4.87E-62 | 1.17E-61 |  |
| GALNT17 | 4.064633 | 1.423765 | -2.64087 | 6.50E-64 | 1.61E-63 |  |
| ANKRD19P | 3.271916 | 0.636466 | -2.63545 | 1.65E-89 | 9.69E-89 |  |
| MGST3 | 6.600488 | 3.965208 | -2.63528 | 3.80E-95 | 5.85E-94 |  |
| PMS2P6 | 2.705162 | 0.069923 | -2.63524 | 3.53E-96 | 8.74E-95 |  |
| NPIPA3 | 3.228377 | 0.594955 | -2.63342 | 1.05E-93 | 9.98E-93 |  |
| RPL37 | 8.56669 | 5.933509 | -2.63318 | 1.68E-93 | 1.54E-92 |  |
| FABP6 | 3.241033 | 0.610685 | -2.63035 | 4.69E-86 | 2.24E-85 |  |
| CRIP1 | 3.119093 | 0.488906 | -2.63019 | 1.40E-90 | 8.94E-90 |  |
| NEFL | 4.997554 | 2.368274 | -2.62928 | 1.94E-46 | 3.68E-46 |  |
| MAL2 | 3.304644 | 0.675608 | -2.62904 | 1.81E-74 | 5.71E-74 |  |
| DLGAP3 | 3.931973 | 1.305087 | -2.62689 | 1.31E-74 | 4.14E-74 |  |
| RTEL1 | 2.825395 | 0.199076 | -2.62632 | 1.57E-95 | 2.90E-94 |  |
| ST18 | 3.149631 | 0.523904 | -2.62573 | 4.40E-88 | 2.36E-87 |  |
| WIF1 | 3.349436 | 0.727049 | -2.62239 | 3.76E-61 | 8.88E-61 |  |
| AC146944.4 | 2.874758 | 0.253215 | -2.62154 | 8.55E-95 | 1.13E-93 |  |
| RPL17-C18orf32 | 2.647617 | 0.026926 | -2.62069 | 1.21E-94 | 1.51E-93 |  |
| KCNC1 | 3.762187 | 1.144214 | -2.61797 | 5.41E-75 | 1.73E-74 |  |
| LINC01001 | 2.836777 | 0.218876 | -2.6179 | 8.42E-95 | 1.12E-93 |  |
| KPNA2P3 | 2.614566 | 0 | -2.61457 | 2.92E-95 | 4.75E-94 |  |
| RRP7BP | 3.862879 | 1.248396 | -2.61448 | 1.17E-94 | 1.47E-93 |  |
| NDST2 | 3.277244 | 0.665348 | -2.6119 | 1.92E-95 | 3.40E-94 |  |
| KRT222 | 2.75656 | 0.146885 | -2.60967 | 5.63E-93 | 4.70E-92 |  |
| LRRC37A2 | 3.33273 | 0.723405 | -2.60932 | 7.73E-95 | 1.05E-93 |  |
| PRSS3 | 3.843848 | 1.239542 | -2.60431 | 1.14E-80 | 4.31E-80 |  |
| DDX39B | 6.675538 | 4.073966 | -2.60157 | 2.91E-94 | 3.22E-93 |  |
| SYN2 | 4.884047 | 2.283336 | -2.60071 | 4.40E-60 | 1.02E-59 |  |
| ATP6V1G2 | 6.799481 | 4.20051 | -2.59897 | 7.39E-84 | 3.18E-83 |  |
| AGAP4 | 3.461207 | 0.862758 | -2.59845 | 1.25E-94 | 1.55E-93 |  |
| RPL26 | 9.272186 | 6.675148 | -2.59704 | 2.98E-94 | 3.30E-93 |  |
| ZSWIM8 | 5.860382 | 3.263938 | -2.59644 | 2.40E-95 | 4.06E-94 |  |
| TAGLN3 | 7.032691 | 4.436946 | -2.59575 | 3.24E-67 | 8.58E-67 |  |
| ILK | 5.227353 | 2.633236 | -2.59412 | 1.98E-94 | 2.31E-93 |  |
| L1CAM | 4.432321 | 1.838889 | -2.59343 | 1.47E-56 | 3.22E-56 |  |
| AC138649.1 | 3.197282 | 0.605786 | -2.5915 | 1.78E-93 | 1.62E-92 |  |
| ZNF337 | 3.628653 | 1.038739 | -2.58991 | 3.49E-95 | 5.43E-94 |  |
| IDH3A | 5.13052 | 2.544076 | -2.58644 | 1.21E-95 | 2.41E-94 |  |
| DDX25 | 3.596617 | 1.010406 | -2.58621 | 7.39E-93 | 6.06E-92 |  |
| DIABLO | 4.679344 | 2.093493 | -2.58585 | 4.29E-94 | 4.49E-93 |  |
| LRRC7 | 2.850053 | 0.265891 | -2.58416 | 2.75E-89 | 1.60E-88 |  |
| NDUFA13 | 7.261499 | 4.678549 | -2.58295 | 1.03E-93 | 9.78E-93 |  |
| RPS10-NUDT3 | 2.834151 | 0.252153 | -2.582 | 8.65E-86 | 4.07E-85 |  |
| HPCAL4 | 5.045349 | 2.463583 | -2.58177 | 3.50E-67 | 9.24E-67 |  |
| P2RX5 | 2.813641 | 0.23201 | -2.58163 | 8.97E-94 | 8.69E-93 |  |
| AC091564.4 | 2.577698 | 0 | -2.5777 | 2.21E-96 | 6.00E-95 |  |
| LINC00982 | 3.372127 | 0.796676 | -2.57545 | 7.45E-59 | 1.69E-58 |  |
| ABCB6 | 4.498943 | 1.924068 | -2.57488 | 1.05E-94 | 1.35E-93 |  |
| NAIP | 2.856787 | 0.284111 | -2.57268 | 3.80E-94 | 4.04E-93 |  |
| HDAC10 | 4.19096 | 1.618976 | -2.57198 | 8.08E-94 | 7.87E-93 |  |
| AC010649.1 | 2.632363 | 0.060699 | -2.57166 | 1.41E-65 | 3.61E-65 |  |
| RPS24 | 9.554211 | 6.985249 | -2.56896 | 3.79E-94 | 4.03E-93 |  |
| MTURN | 7.680441 | 5.111518 | -2.56892 | 1.71E-76 | 5.70E-76 |  |
| ARPIN-AP3S2 | 2.77201 | 0.204299 | -2.56771 | 2.31E-95 | 3.94E-94 |  |
| HPCAL1 | 5.906829 | 3.340214 | -2.56662 | 1.48E-86 | 7.25E-86 |  |
| RPH3A | 4.554646 | 1.991605 | -2.56304 | 7.14E-58 | 1.60E-57 |  |
| PRPF40B | 3.700782 | 1.138492 | -2.56229 | 1.33E-95 | 2.58E-94 |  |
| AC034102.4 | 2.562176 | 0 | -2.56218 | 5.02E-97 | 2.11E-95 |  |
| SYNDIG1L | 2.892892 | 0.330972 | -2.56192 | 1.44E-71 | 4.21E-71 |  |
| ATP6V0C | 7.757996 | 5.196362 | -2.56163 | 9.92E-95 | 1.29E-93 |  |
| SMIM11A | 2.577028 | 0.015571 | -2.56146 | 1.88E-95 | 3.35E-94 |  |
| COL7A1 | 3.833243 | 1.274821 | -2.55842 | 4.80E-86 | 2.29E-85 |  |
| TNK2 | 6.188174 | 3.630248 | -2.55793 | 2.43E-92 | 1.86E-91 |  |
| AL136131.3 | 2.557198 | 0 | -2.5572 | 1.83E-81 | 7.13E-81 |  |
| TNNT1 | 3.334144 | 0.778122 | -2.55602 | 5.79E-78 | 2.01E-77 |  |
| RAB3A | 6.233197 | 3.677307 | -2.55589 | 2.02E-66 | 5.25E-66 |  |
| AC011472.3 | 2.553053 | 0 | -2.55305 | 5.02E-97 | 2.11E-95 |  |
| CAMKV | 4.589441 | 2.040129 | -2.54931 | 1.81E-39 | 3.14E-39 |  |
| AC018557.1 | 2.549287 | 0 | -2.54929 | 4.63E-96 | 1.08E-94 |  |
| SST | 4.445413 | 1.896316 | -2.5491 | 7.82E-26 | 1.15E-25 |  |
| PCBP1-AS1 | 3.736328 | 1.191651 | -2.54468 | 2.11E-94 | 2.43E-93 |  |
| TEN1 | 2.664913 | 0.120733 | -2.54418 | 7.89E-95 | 1.06E-93 |  |
| EML2 | 4.983784 | 2.440626 | -2.54316 | 1.21E-95 | 2.42E-94 |  |
| ROGDI | 6.303341 | 3.760328 | -2.54301 | 1.11E-93 | 1.05E-92 |  |
| DGKZ | 6.163107 | 3.622235 | -2.54087 | 7.46E-93 | 6.11E-92 |  |
| PRR4 | 2.988475 | 0.450667 | -2.53781 | 1.12E-94 | 1.42E-93 |  |
| LCNL1 | 3.390726 | 0.853318 | -2.53741 | 6.36E-86 | 3.01E-85 |  |
| SLC2A11 | 3.887381 | 1.350378 | -2.537 | 1.24E-95 | 2.47E-94 |  |
| PKD1P6 | 3.806519 | 1.270369 | -2.53615 | 1.71E-92 | 1.33E-91 |  |
| PINK1 | 6.676 | 4.140044 | -2.53596 | 3.97E-95 | 6.08E-94 |  |
| AC008738.3 | 2.535258 | 0 | -2.53526 | 1.05E-96 | 3.43E-95 |  |
| INO80B-WBP1 | 2.547746 | 0.01265 | -2.5351 | 1.36E-80 | 5.12E-80 |  |
| CATSPER2 | 2.972568 | 0.437775 | -2.53479 | 1.37E-95 | 2.62E-94 |  |
| KIF1A | 7.011617 | 4.478074 | -2.53354 | 7.05E-89 | 3.97E-88 |  |
| GABRD | 4.658164 | 2.124866 | -2.5333 | 3.37E-46 | 6.38E-46 |  |
| COMMD3-BMI1 | 2.537626 | 0.005224 | -2.5324 | 2.06E-96 | 5.73E-95 |  |
| SELENOW | 8.891665 | 6.362182 | -2.52948 | 4.62E-95 | 6.88E-94 |  |
| CUL9 | 4.828671 | 2.299277 | -2.52939 | 2.67E-97 | 1.76E-95 |  |
| YPEL3 | 7.168773 | 4.639684 | -2.52909 | 8.13E-95 | 1.09E-93 |  |
| NECAB2 | 4.537031 | 2.011283 | -2.52575 | 9.53E-42 | 1.70E-41 |  |
| AC025259.1 | 2.52166 | 0.000291 | -2.52137 | 4.30E-96 | 1.02E-94 |  |
| BRF1 | 4.136572 | 1.617449 | -2.51912 | 1.35E-95 | 2.61E-94 |  |
| MTND2P28 | 9.273909 | 6.755751 | -2.51816 | 1.55E-74 | 4.89E-74 |  |
| CBWD1 | 3.367448 | 0.850849 | -2.5166 | 2.99E-94 | 3.30E-93 |  |
| SPTB | 2.756526 | 0.243206 | -2.51332 | 1.17E-92 | 9.31E-92 |  |
| RPL34 | 9.381334 | 6.871067 | -2.51027 | 4.80E-94 | 4.95E-93 |  |
| PLA2G4C | 4.884832 | 2.377005 | -2.50783 | 1.95E-97 | 1.61E-95 |  |
| KIF5C | 6.417083 | 3.915396 | -2.50169 | 2.29E-90 | 1.44E-89 |  |
| NBL1 | 5.336523 | 2.835049 | -2.50147 | 6.32E-85 | 2.85E-84 |  |
| GAD2 | 3.244598 | 0.744683 | -2.49992 | 7.44E-69 | 2.04E-68 |  |
| AC138932.1 | 3.094916 | 0.596627 | -2.49829 | 7.44E-93 | 6.10E-92 |  |
| DOC2B | 3.433467 | 0.935184 | -2.49828 | 1.88E-82 | 7.61E-82 |  |
| LCN12 | 2.977113 | 0.479326 | -2.49779 | 5.70E-91 | 3.78E-90 |  |
| AC093525.6 | 2.693127 | 0.195544 | -2.49758 | 2.16E-94 | 2.48E-93 |  |
| GNAO1 | 6.394453 | 3.901779 | -2.49267 | 1.98E-93 | 1.78E-92 |  |
| AC091053.1 | 2.492004 | 0 | -2.492 | 6.90E-93 | 5.70E-92 |  |
| CCZ1 | 4.535906 | 2.046192 | -2.48971 | 4.40E-94 | 4.60E-93 |  |
| COX6C | 8.101207 | 5.613273 | -2.48793 | 4.23E-95 | 6.42E-94 |  |
| TMCC2 | 4.968387 | 2.48065 | -2.48774 | 9.49E-90 | 5.67E-89 |  |
| LSP1P4 | 3.601945 | 1.114703 | -2.48724 | 8.49E-88 | 4.46E-87 |  |
| CHN2 | 3.862574 | 1.375747 | -2.48683 | 5.15E-91 | 3.42E-90 |  |
| MZT2A | 5.844512 | 3.357699 | -2.48681 | 1.16E-93 | 1.09E-92 |  |
| PPFIA2 | 3.341667 | 0.854891 | -2.48678 | 6.25E-93 | 5.18E-92 |  |
| ABCA5 | 3.551978 | 1.065348 | -2.48663 | 7.87E-95 | 1.06E-93 |  |
| SYT4 | 3.804034 | 1.318027 | -2.48601 | 1.04E-57 | 2.31E-57 |  |
| CYFIP2 | 6.073268 | 3.587885 | -2.48538 | 3.71E-91 | 2.50E-90 |  |
| HERC1 | 4.595777 | 2.110616 | -2.48516 | 1.68E-94 | 2.01E-93 |  |
| NPIPP1 | 5.238127 | 2.753398 | -2.48473 | 6.12E-90 | 3.71E-89 |  |
| SH3GLB2 | 6.296203 | 3.812261 | -2.48394 | 3.94E-94 | 4.16E-93 |  |
| NPIPA8 | 2.527005 | 0.045252 | -2.48175 | 5.27E-84 | 2.28E-83 |  |
| PPP1R16B | 4.553008 | 2.071327 | -2.48168 | 6.12E-73 | 1.85E-72 |  |
| TRIM17 | 2.84648 | 0.36759 | -2.47889 | 2.40E-88 | 1.31E-87 |  |
| AC010653.2 | 2.503996 | 0.027596 | -2.4764 | 4.77E-93 | 4.02E-92 |  |
| NDUFB1 | 7.559342 | 5.083438 | -2.4759 | 3.07E-94 | 3.38E-93 |  |
| MALAT1 | 6.836672 | 4.360794 | -2.47588 | 5.76E-82 | 2.29E-81 |  |
| PI4KAP2 | 3.68579 | 1.213354 | -2.47244 | 6.40E-94 | 6.37E-93 |  |
| BBIP1 | 4.039029 | 1.567099 | -2.47193 | 1.65E-95 | 3.02E-94 |  |
| C9orf24 | 4.03778 | 1.566802 | -2.47098 | 5.44E-62 | 1.31E-61 |  |
| PDE2A | 4.421371 | 1.951364 | -2.47001 | 5.38E-49 | 1.06E-48 |  |
| APOC4-APOC2 | 3.093519 | 0.625455 | -2.46806 | 2.19E-77 | 7.46E-77 |  |
| RPS6KL1 | 4.171085 | 1.704509 | -2.46658 | 7.80E-93 | 6.36E-92 |  |
| NPEPL1 | 4.59664 | 2.134431 | -2.46221 | 1.67E-92 | 1.30E-91 |  |
| MIA2 | 2.797012 | 0.336692 | -2.46032 | 2.03E-95 | 3.55E-94 |  |
| TARBP1 | 4.734627 | 2.278833 | -2.45579 | 1.17E-92 | 9.27E-92 |  |
| SLIRP | 6.440373 | 3.984698 | -2.45568 | 1.33E-94 | 1.64E-93 |  |
| SFTPC | 2.763669 | 0.309106 | -2.45456 | 5.38E-94 | 5.47E-93 |  |
| MAPK8IP2 | 5.612271 | 3.158846 | -2.45343 | 6.86E-83 | 2.84E-82 |  |
| MYH7B | 2.774732 | 0.321747 | -2.45298 | 1.80E-95 | 3.23E-94 |  |
| PLSCR3 | 2.995522 | 0.545547 | -2.44998 | 3.62E-94 | 3.87E-93 |  |
| GABRA2 | 3.230463 | 0.782302 | -2.44816 | 1.21E-78 | 4.27E-78 |  |
| LGI3 | 4.769685 | 2.321769 | -2.44792 | 5.15E-68 | 1.39E-67 |  |
| HDDC2 | 6.259672 | 3.811827 | -2.44785 | 1.52E-94 | 1.85E-93 |  |
| KREMEN1 | 2.443371 | 0 | -2.44337 | 5.02E-97 | 2.11E-95 |  |
| FTCD | 2.71096 | 0.267717 | -2.44324 | 7.83E-93 | 6.38E-92 |  |
| TUBA4A | 5.664203 | 3.2214 | -2.4428 | 8.35E-76 | 2.73E-75 |  |
| LGI4 | 4.845697 | 2.40327 | -2.44243 | 5.73E-78 | 1.99E-77 |  |
| SPTAN1 | 7.880467 | 5.438171 | -2.4423 | 1.60E-93 | 1.47E-92 |  |
| ABTB1 | 5.109721 | 2.670055 | -2.43967 | 7.66E-95 | 1.04E-93 |  |
| L3MBTL1 | 3.428733 | 0.991279 | -2.43745 | 1.21E-94 | 1.51E-93 |  |
| AC015674.1 | 2.436576 | 0 | -2.43658 | 1.34E-86 | 6.57E-86 |  |
| GABRA1 | 3.358581 | 0.922626 | -2.43596 | 3.20E-61 | 7.57E-61 |  |
| SLC27A5 | 4.129904 | 1.694385 | -2.43552 | 1.05E-92 | 8.41E-92 |  |
| EPB41L4A-AS1 | 5.301079 | 2.865698 | -2.43538 | 1.86E-95 | 3.32E-94 |  |
| DISP2 | 3.529052 | 1.093789 | -2.43526 | 4.47E-88 | 2.40E-87 |  |
| MEMO1 | 3.543833 | 1.109699 | -2.43413 | 1.29E-94 | 1.60E-93 |  |
| FAM106A | 2.535571 | 0.101749 | -2.43382 | 1.43E-95 | 2.70E-94 |  |
| C11orf98 | 4.9164 | 2.484512 | -2.43189 | 2.82E-94 | 3.14E-93 |  |
| NRSN1 | 4.437454 | 2.00733 | -2.43012 | 4.19E-67 | 1.11E-66 |  |
| CBWD2 | 3.845937 | 1.416577 | -2.42936 | 2.95E-94 | 3.27E-93 |  |
| KIAA1109 | 4.429842 | 2.001798 | -2.42804 | 1.22E-95 | 2.43E-94 |  |
| AGAP2 | 5.752179 | 3.324341 | -2.42784 | 1.10E-47 | 2.12E-47 |  |
| SPOCK3 | 4.490034 | 2.062218 | -2.42782 | 6.52E-62 | 1.57E-61 |  |
| SPX | 3.674188 | 1.248 | -2.42619 | 9.41E-69 | 2.57E-68 |  |
| TGFBR3L | 3.068096 | 0.643482 | -2.42461 | 2.48E-83 | 1.04E-82 |  |
| AL096711.2 | 2.624526 | 0.200437 | -2.42409 | 9.94E-96 | 2.06E-94 |  |
| DGCR5 | 3.7973 | 1.374039 | -2.42326 | 9.97E-82 | 3.91E-81 |  |
| LINC00869 | 3.679996 | 1.256955 | -2.42304 | 1.90E-94 | 2.23E-93 |  |
| LHPP | 5.92236 | 3.499628 | -2.42273 | 2.72E-77 | 9.23E-77 |  |
| VIPR1 | 2.919955 | 0.498123 | -2.42183 | 7.25E-86 | 3.43E-85 |  |
| AC104072.1 | 3.741188 | 1.321607 | -2.41958 | 1.29E-62 | 3.15E-62 |  |
| CALN1 | 3.033792 | 0.614506 | -2.41929 | 6.19E-85 | 2.80E-84 |  |
| TUT4 | 4.415191 | 1.998766 | -2.41642 | 6.33E-94 | 6.31E-93 |  |
| CYP2E1 | 2.565862 | 0.149953 | -2.41591 | 7.13E-96 | 1.56E-94 |  |
| SLCO1A2 | 3.486653 | 1.071321 | -2.41533 | 3.33E-72 | 9.86E-72 |  |
| MAP4K3-DT | 3.549834 | 1.134747 | -2.41509 | 5.78E-95 | 8.26E-94 |  |
| PRKCB | 4.217923 | 1.80378 | -2.41414 | 2.26E-73 | 6.91E-73 |  |
| ZSCAN18 | 5.845474 | 3.432806 | -2.41267 | 1.53E-95 | 2.85E-94 |  |
| PCBP3 | 3.155775 | 0.743308 | -2.41247 | 1.24E-93 | 1.16E-92 |  |
| CDR1 | 2.580953 | 0.169393 | -2.41156 | 4.12E-94 | 4.33E-93 |  |
| AC012615.2 | 2.407375 | 0 | -2.40737 | 9.17E-83 | 3.76E-82 |  |
| ADARB2 | 2.869566 | 0.463668 | -2.4059 | 1.98E-90 | 1.25E-89 |  |
| CHMP1B-AS1 | 2.405833 | 0 | -2.40583 | 3.35E-93 | 2.89E-92 |  |
| CCDC180 | 2.542852 | 0.137222 | -2.40563 | 9.59E-96 | 2.01E-94 |  |
| FAM204A | 4.270079 | 1.865019 | -2.40506 | 3.00E-95 | 4.84E-94 |  |
| AC138866.1 | 2.60528 | 0.201201 | -2.40408 | 2.45E-91 | 1.68E-90 |  |
| RAPGEFL1 | 4.428791 | 2.025916 | -2.40287 | 3.88E-86 | 1.86E-85 |  |
| KCNC4 | 2.882128 | 0.479867 | -2.40226 | 1.54E-94 | 1.86E-93 |  |
| TPM1 | 5.677883 | 3.277937 | -2.39995 | 2.41E-88 | 1.32E-87 |  |
| NAPB | 5.570461 | 3.1709 | -2.39956 | 8.01E-77 | 2.69E-76 |  |
| BCL11A | 3.252072 | 0.856765 | -2.39531 | 6.19E-60 | 1.43E-59 |  |
| WASH3P | 4.466939 | 2.072185 | -2.39475 | 2.77E-94 | 3.10E-93 |  |
| ETFB | 5.920697 | 3.530416 | -2.39028 | 6.64E-92 | 4.85E-91 |  |
| AC005786.3 | 2.389527 | 0 | -2.38953 | 4.80E-79 | 1.73E-78 |  |
| GCSHP5 | 4.189556 | 1.800567 | -2.38899 | 3.10E-86 | 1.49E-85 |  |
| GABBR1 | 6.548935 | 4.160079 | -2.38886 | 2.11E-82 | 8.54E-82 |  |
| WFDC1 | 2.91381 | 0.525219 | -2.38859 | 7.73E-93 | 6.31E-92 |  |
| LRRC24 | 2.41209 | 0.025006 | -2.38708 | 3.96E-96 | 9.59E-95 |  |
| RSRP1 | 5.843321 | 3.457072 | -2.38625 | 5.55E-94 | 5.60E-93 |  |
| SHTN1 | 5.178262 | 2.793558 | -2.3847 | 2.65E-77 | 9.00E-77 |  |
| RBP4 | 3.621246 | 1.237602 | -2.38364 | 3.08E-37 | 5.19E-37 |  |
| ERCC5 | 4.267402 | 1.885576 | -2.38183 | 2.81E-95 | 4.61E-94 |  |
| RPL38 | 9.117118 | 6.736415 | -2.3807 | 4.02E-94 | 4.24E-93 |  |
| NPIPA7 | 2.40874 | 0.028229 | -2.38051 | 6.79E-95 | 9.43E-94 |  |
| LY6H | 5.320055 | 2.94092 | -2.37914 | 6.10E-33 | 9.77E-33 |  |
| TTLL3 | 4.428326 | 2.052317 | -2.37601 | 2.80E-95 | 4.59E-94 |  |
| AC010132.3 | 2.380309 | 0.006614 | -2.37369 | 5.14E-97 | 2.14E-95 |  |
| SEC31B | 3.608809 | 1.235332 | -2.37348 | 1.18E-89 | 7.03E-89 |  |
| AKAP2 | 2.392111 | 0.019993 | -2.37212 | 3.57E-96 | 8.81E-95 |  |
| SLC6A17 | 3.620382 | 1.250356 | -2.37003 | 2.04E-63 | 5.04E-63 |  |
| PPP1R12C | 6.058497 | 3.689411 | -2.36909 | 1.25E-94 | 1.55E-93 |  |
| ZFR2 | 2.845219 | 0.476432 | -2.36879 | 2.33E-89 | 1.36E-88 |  |
| CCDC85C | 3.767526 | 1.403711 | -2.36382 | 4.97E-95 | 7.28E-94 |  |
| PAM16 | 4.686483 | 2.323724 | -2.36276 | 2.03E-94 | 2.35E-93 |  |
| MAP7D2 | 3.304386 | 0.942455 | -2.36193 | 1.20E-77 | 4.13E-77 |  |
| STX1B | 5.066069 | 2.704639 | -2.36143 | 1.20E-69 | 3.35E-69 |  |
| TOGARAM2 | 2.57275 | 0.211738 | -2.36101 | 7.56E-95 | 1.03E-93 |  |
| TUNAR | 2.746483 | 0.386 | -2.36048 | 2.85E-80 | 1.06E-79 |  |
| TM2D3 | 5.077278 | 2.718339 | -2.35894 | 1.15E-95 | 2.34E-94 |  |
| CAMKK1 | 4.114991 | 1.756177 | -2.35881 | 8.17E-72 | 2.40E-71 |  |
| C1orf61 | 8.7077 | 6.350318 | -2.35738 | 1.41E-76 | 4.71E-76 |  |
| SLC25A48 | 3.234824 | 0.879931 | -2.35489 | 1.93E-72 | 5.77E-72 |  |
| SNRPN | 8.069314 | 5.715804 | -2.35351 | 3.53E-96 | 8.74E-95 |  |
| HSPA1A | 7.11599 | 4.764262 | -2.35173 | 3.79E-62 | 9.14E-62 |  |
| ZFYVE21 | 5.654443 | 3.303208 | -2.35123 | 4.97E-95 | 7.28E-94 |  |
| KCNK1 | 3.98324 | 1.632461 | -2.35078 | 8.92E-71 | 2.56E-70 |  |
| RPL39 | 8.613821 | 6.263905 | -2.34992 | 5.42E-92 | 4.00E-91 |  |
| SV2B | 3.079068 | 0.730877 | -2.34819 | 5.57E-69 | 1.53E-68 |  |
| PITPNM3 | 3.408843 | 1.061144 | -2.3477 | 2.06E-73 | 6.32E-73 |  |
| GABRA5 | 3.094292 | 0.747575 | -2.34672 | 6.87E-42 | 1.23E-41 |  |
| UROS | 5.301118 | 2.95453 | -2.34659 | 3.27E-92 | 2.48E-91 |  |
| PNISR | 5.568739 | 3.222899 | -2.34584 | 6.65E-93 | 5.50E-92 |  |
| KCNH3 | 4.1214 | 1.776163 | -2.34524 | 7.74E-58 | 1.73E-57 |  |
| CBLN1 | 2.810483 | 0.469302 | -2.34118 | 1.22E-60 | 2.85E-60 |  |
| PNMA3 | 3.521311 | 1.180658 | -2.34065 | 4.93E-76 | 1.62E-75 |  |
| KIAA0930 | 6.115969 | 3.780773 | -2.3352 | 2.26E-77 | 7.71E-77 |  |
| MDN1 | 3.99611 | 1.661214 | -2.3349 | 1.34E-95 | 2.58E-94 |  |
| ATP5MPL | 7.107229 | 4.773107 | -2.33412 | 4.72E-95 | 6.98E-94 |  |
| NDUFA6-DT | 3.013235 | 0.679311 | -2.33392 | 1.38E-95 | 2.63E-94 |  |
| DEXI | 4.829757 | 2.49709 | -2.33267 | 3.38E-95 | 5.29E-94 |  |
| STRADA | 4.44935 | 2.11724 | -2.33211 | 6.45E-95 | 9.03E-94 |  |
| ANXA3 | 2.661197 | 0.329622 | -2.33158 | 3.20E-93 | 2.77E-92 |  |
| AC233968.1 | 2.368269 | 0.037512 | -2.33076 | 3.15E-89 | 1.82E-88 |  |
| LINC01123 | 2.372459 | 0.044403 | -2.32806 | 3.34E-93 | 2.89E-92 |  |
| STARD10 | 5.069114 | 2.743475 | -2.32564 | 1.02E-94 | 1.31E-93 |  |
| RNF220 | 5.870791 | 3.545575 | -2.32522 | 1.34E-95 | 2.59E-94 |  |
| RAB4B | 5.306679 | 2.981609 | -2.32507 | 1.94E-93 | 1.75E-92 |  |
| SIGIRR | 4.322429 | 1.998229 | -2.3242 | 3.81E-78 | 1.33E-77 |  |
| GAK | 5.222906 | 2.900648 | -2.32226 | 1.24E-95 | 2.47E-94 |  |
| AC087190.3 | 2.319687 | 0 | -2.31969 | 6.70E-96 | 1.48E-94 |  |
| AKR1C1 | 2.872316 | 0.553665 | -2.31865 | 1.08E-85 | 5.04E-85 |  |
| MIR7-3HG | 3.184913 | 0.866678 | -2.31824 | 1.16E-69 | 3.22E-69 |  |
| DNAJC7 | 5.725055 | 3.407824 | -2.31723 | 1.31E-95 | 2.55E-94 |  |
| SLC6A12 | 3.512322 | 1.195954 | -2.31637 | 3.04E-93 | 2.64E-92 |  |
| ZFYVE28 | 3.649113 | 1.333257 | -2.31586 | 5.91E-95 | 8.41E-94 |  |
| FAM219B | 4.8124 | 2.497644 | -2.31476 | 2.68E-95 | 4.43E-94 |  |
| MAST1 | 4.480437 | 2.165729 | -2.31471 | 6.32E-56 | 1.37E-55 |  |
| DYNC1I1 | 4.315385 | 2.001235 | -2.31415 | 1.49E-68 | 4.06E-68 |  |
| NCDN | 7.002218 | 4.689642 | -2.31258 | 5.57E-68 | 1.50E-67 |  |
| MOK | 3.705471 | 1.394141 | -2.31133 | 1.15E-96 | 3.65E-95 |  |
| CPLX3 | 2.308993 | 7.51E-05 | -2.30892 | 3.85E-97 | 1.95E-95 |  |
| AC011511.4 | 2.311392 | 0.003168 | -2.30822 | 1.76E-96 | 5.07E-95 |  |
| RAP1GAP | 5.693296 | 3.387736 | -2.30556 | 6.72E-72 | 1.98E-71 |  |
| DNAJB6 | 5.829531 | 3.524469 | -2.30506 | 2.72E-94 | 3.05E-93 |  |
| CLK1 | 6.444271 | 4.139229 | -2.30504 | 2.85E-93 | 2.49E-92 |  |
| NUTM2B-AS1 | 2.833693 | 0.529002 | -2.30469 | 7.23E-95 | 9.94E-94 |  |
| WBP1 | 5.990742 | 3.688273 | -2.30247 | 1.49E-93 | 1.37E-92 |  |
| BAIAP2 | 5.2486 | 2.946344 | -2.30226 | 1.80E-87 | 9.31E-87 |  |
| SLC17A7 | 4.648061 | 2.346345 | -2.30172 | 7.48E-14 | 9.48E-14 |  |
| WASH9P | 4.139121 | 1.839817 | -2.2993 | 6.95E-93 | 5.73E-92 |  |
| TTN-AS1 | 2.441049 | 0.141784 | -2.29926 | 1.23E-95 | 2.45E-94 |  |
| FCHO1 | 3.716412 | 1.417762 | -2.29865 | 2.78E-94 | 3.10E-93 |  |
| STXBP5 | 3.386375 | 1.089274 | -2.2971 | 2.44E-93 | 2.16E-92 |  |
| APLP1 | 8.602129 | 6.306161 | -2.29597 | 3.12E-79 | 1.13E-78 |  |
| RALGAPA1 | 3.832718 | 1.537252 | -2.29547 | 1.22E-95 | 2.43E-94 |  |
| SGIP1 | 3.533082 | 1.239917 | -2.29316 | 1.85E-95 | 3.30E-94 |  |
| GABRG1 | 2.818295 | 0.525325 | -2.29297 | 1.86E-79 | 6.77E-79 |  |
| AC012617.1 | 2.696697 | 0.404058 | -2.29264 | 2.22E-94 | 2.54E-93 |  |
| AC024267.6 | 2.327616 | 0.036501 | -2.29111 | 1.49E-95 | 2.79E-94 |  |
| PSMG4 | 3.528219 | 1.23814 | -2.29008 | 1.32E-94 | 1.63E-93 |  |
| CHCHD10 | 6.761727 | 4.474788 | -2.28694 | 6.08E-91 | 4.01E-90 |  |
| GNB5 | 4.194252 | 1.908479 | -2.28577 | 3.31E-92 | 2.50E-91 |  |
| CNDP1 | 4.335635 | 2.050266 | -2.28537 | 3.70E-47 | 7.08E-47 |  |
| ANAPC15 | 4.965168 | 2.680167 | -2.285 | 1.77E-90 | 1.12E-89 |  |
| DLG4 | 6.50612 | 4.223556 | -2.28256 | 7.28E-78 | 2.52E-77 |  |
| MTATP6P1 | 11.88453 | 9.602492 | -2.28204 | 5.89E-89 | 3.33E-88 |  |
| CACNA1B | 2.622791 | 0.342407 | -2.28038 | 5.42E-89 | 3.08E-88 |  |
| MAL | 5.429672 | 3.151511 | -2.27816 | 2.29E-49 | 4.53E-49 |  |
| MIB2 | 5.272989 | 2.995207 | -2.27778 | 1.80E-93 | 1.63E-92 |  |
| SERGEF | 5.675187 | 3.398544 | -2.27664 | 5.43E-95 | 7.85E-94 |  |
| TF | 6.331537 | 4.055109 | -2.27643 | 1.37E-43 | 2.51E-43 |  |
| BCRP3 | 2.639383 | 0.364873 | -2.27451 | 6.09E-93 | 5.06E-92 |  |
| HAPLN4 | 2.279471 | 0.005028 | -2.27444 | 2.66E-96 | 7.06E-95 |  |
| LUC7L3 | 6.535086 | 4.261444 | -2.27364 | 1.14E-91 | 8.12E-91 |  |
| LYRM9 | 4.443219 | 2.169673 | -2.27355 | 2.20E-86 | 1.07E-85 |  |
| TGFB2-OT1 | 2.270403 | 0 | -2.2704 | 5.02E-97 | 2.11E-95 |  |
| GABRB3 | 3.273995 | 1.006576 | -2.26742 | 1.97E-75 | 6.38E-75 |  |
| STMN4 | 6.091092 | 3.82371 | -2.26738 | 2.39E-68 | 6.47E-68 |  |
| TRIM3 | 4.465272 | 2.198742 | -2.26653 | 2.68E-95 | 4.43E-94 |  |
| PCP4L1 | 3.871504 | 1.605564 | -2.26594 | 7.12E-50 | 1.42E-49 |  |
| CHN1 | 6.887093 | 4.621582 | -2.26551 | 1.13E-55 | 2.44E-55 |  |
| AL121845.2 | 2.265249 | 0.000477 | -2.26477 | 2.95E-89 | 1.71E-88 |  |
| COX4I1 | 8.739213 | 6.474792 | -2.26442 | 3.28E-95 | 5.20E-94 |  |
| ARHGEF4 | 5.768459 | 3.504538 | -2.26392 | 7.89E-82 | 3.11E-81 |  |
| MAPK8IP3 | 5.967005 | 3.704309 | -2.2627 | 6.76E-91 | 4.44E-90 |  |
| CAMTA2 | 5.860281 | 3.598077 | -2.2622 | 6.14E-94 | 6.15E-93 |  |
| GABRG2 | 3.34381 | 1.082166 | -2.26164 | 1.08E-57 | 2.40E-57 |  |
| CCDC92 | 5.622968 | 3.363102 | -2.25987 | 2.73E-95 | 4.48E-94 |  |
| CPEB1 | 3.431547 | 1.17336 | -2.25819 | 3.12E-94 | 3.42E-93 |  |
| CA10 | 3.877619 | 1.620954 | -2.25667 | 5.57E-48 | 1.08E-47 |  |
| HERC2 | 4.676855 | 2.420318 | -2.25654 | 1.56E-95 | 2.90E-94 |  |
| BCO2 | 2.689904 | 0.433609 | -2.25629 | 7.66E-95 | 1.04E-93 |  |
| BEX5 | 5.104179 | 2.847948 | -2.25623 | 8.81E-67 | 2.31E-66 |  |
| SYNGR1 | 5.678494 | 3.423464 | -2.25503 | 1.18E-80 | 4.45E-80 |  |
| RGS7 | 3.704086 | 1.449951 | -2.25414 | 1.59E-75 | 5.14E-75 |  |
| GATD3A | 3.415667 | 1.16182 | -2.25385 | 8.01E-78 | 2.77E-77 |  |
| MMP17 | 4.12135 | 1.86938 | -2.25197 | 5.37E-54 | 1.13E-53 |  |
| GABRB2 | 2.826178 | 0.574378 | -2.2518 | 3.80E-69 | 1.05E-68 |  |
| SMN2 | 3.634079 | 1.382607 | -2.25147 | 1.27E-73 | 3.91E-73 |  |
| RASAL1 | 2.899429 | 0.648529 | -2.2509 | 7.43E-67 | 1.95E-66 |  |
| SORBS2 | 3.449598 | 1.198714 | -2.25088 | 9.93E-94 | 9.51E-93 |  |
| HAGH | 5.732403 | 3.482401 | -2.25 | 5.50E-95 | 7.94E-94 |  |
| AP001972.5 | 5.027105 | 2.778487 | -2.24862 | 5.65E-76 | 1.85E-75 |  |
| SPACA6 | 3.896775 | 1.648639 | -2.24814 | 1.28E-93 | 1.19E-92 |  |
| FDX2 | 4.72726 | 2.479869 | -2.24739 | 1.20E-92 | 9.49E-92 |  |
| FAM228B | 4.362425 | 2.115292 | -2.24713 | 3.43E-95 | 5.34E-94 |  |
| LINC02210 | 4.081336 | 1.835116 | -2.24622 | 3.50E-95 | 5.44E-94 |  |
| ATP8A1 | 4.090166 | 1.844149 | -2.24602 | 5.05E-86 | 2.40E-85 |  |
| PDZD7 | 2.678855 | 0.43355 | -2.2453 | 4.51E-94 | 4.69E-93 |  |
| BPTFP1 | 2.245244 | 0.000406 | -2.24484 | 3.53E-85 | 1.61E-84 |  |
| CNTNAP2 | 3.219237 | 0.974413 | -2.24482 | 9.56E-76 | 3.12E-75 |  |
| LMO7 | 3.244197 | 1.000079 | -2.24412 | 4.18E-93 | 3.56E-92 |  |
| AC239809.3 | 2.325784 | 0.081683 | -2.2441 | 5.25E-96 | 1.20E-94 |  |
| ZNF177 | 2.267789 | 0.026135 | -2.24165 | 1.09E-95 | 2.23E-94 |  |
| CCK | 4.077729 | 1.838559 | -2.23917 | 1.44E-20 | 2.00E-20 |  |
| SCN2B | 3.557678 | 1.31962 | -2.23806 | 1.58E-76 | 5.26E-76 |  |
| CMC1 | 3.753143 | 1.518254 | -2.23489 | 1.28E-93 | 1.20E-92 |  |
| ENOSF1 | 3.701209 | 1.467144 | -2.23406 | 1.03E-84 | 4.62E-84 |  |
| ITIH4 | 2.379098 | 0.14575 | -2.23335 | 1.45E-95 | 2.74E-94 |  |
| AL627309.6 | 3.279057 | 1.046607 | -2.23245 | 7.48E-87 | 3.74E-86 |  |
| USP32P2 | 2.240034 | 0.008495 | -2.23154 | 4.35E-95 | 6.55E-94 |  |
| C4A | 4.545726 | 2.314468 | -2.23126 | 1.74E-67 | 4.62E-67 |  |
| PPP1R13B | 3.862765 | 1.632038 | -2.23073 | 4.27E-95 | 6.46E-94 |  |
| MRNIP | 4.533118 | 2.303442 | -2.22968 | 6.88E-95 | 9.53E-94 |  |
| LIME1 | 3.823628 | 1.594132 | -2.2295 | 9.35E-88 | 4.89E-87 |  |
| PCLO | 2.562633 | 0.333151 | -2.22948 | 2.20E-92 | 1.69E-91 |  |
| VSTM2B | 4.095226 | 1.867432 | -2.22779 | 2.39E-61 | 5.66E-61 |  |
| INSYN1 | 5.094525 | 2.866961 | -2.22756 | 7.45E-83 | 3.07E-82 |  |
| STX16-NPEPL1 | 2.836489 | 0.611476 | -2.22501 | 9.67E-87 | 4.80E-86 |  |
| MTG1 | 3.658183 | 1.435902 | -2.22228 | 1.59E-94 | 1.91E-93 |  |
| ATP6V0A1 | 6.337503 | 4.115303 | -2.2222 | 2.29E-95 | 3.91E-94 |  |
| CARTPT | 2.546563 | 0.32446 | -2.2221 | 1.94E-44 | 3.59E-44 |  |
| SNX32 | 3.637932 | 1.416539 | -2.22139 | 1.93E-87 | 9.95E-87 |  |
| FRA10AC1 | 3.821112 | 1.6019 | -2.21921 | 1.40E-95 | 2.67E-94 |  |
| KALRN | 3.481244 | 1.262409 | -2.21883 | 2.88E-72 | 8.56E-72 |  |
| BLOC1S5-TXNDC5 | 2.26103 | 0.042293 | -2.21874 | 1.72E-94 | 2.06E-93 |  |
| LINC01089 | 4.395593 | 2.176907 | -2.21869 | 2.10E-88 | 1.15E-87 |  |
| ADCY5 | 3.801478 | 1.582803 | -2.21868 | 2.31E-69 | 6.40E-69 |  |
| MIR124-2HG | 2.700008 | 0.482209 | -2.2178 | 3.32E-81 | 1.28E-80 |  |
| ACSL6 | 3.859825 | 1.642533 | -2.21729 | 1.02E-85 | 4.77E-85 |  |
| NEMF | 4.170761 | 1.953747 | -2.21701 | 1.30E-97 | 1.61E-95 |  |
| AC104116.1 | 2.215879 | 0 | -2.21588 | 8.12E-74 | 2.52E-73 |  |
| GNAL | 2.975718 | 0.760709 | -2.21501 | 3.95E-85 | 1.80E-84 |  |
| AHI1 | 4.081425 | 1.86804 | -2.21338 | 5.77E-94 | 5.81E-93 |  |
| BCYRN1 | 2.88256 | 0.669874 | -2.21269 | 1.21E-78 | 4.29E-78 |  |
| HERC3 | 4.150787 | 1.939173 | -2.21161 | 2.98E-96 | 7.69E-95 |  |
| MT-RNR1 | 13.196 | 10.98476 | -2.21124 | 2.09E-73 | 6.40E-73 |  |
| LINC01145 | 2.607182 | 0.397149 | -2.21003 | 5.71E-95 | 8.17E-94 |  |
| ARL17A | 2.401817 | 0.19225 | -2.20957 | 9.80E-95 | 1.28E-93 |  |
| SVOP | 2.860443 | 0.651204 | -2.20924 | 1.46E-71 | 4.27E-71 |  |
| FAM95B1 | 2.246848 | 0.04084 | -2.20601 | 9.85E-96 | 2.05E-94 |  |
| RNF207 | 2.601901 | 0.397432 | -2.20447 | 1.54E-90 | 9.80E-90 |  |
| MAPK10 | 5.097068 | 2.893997 | -2.20307 | 4.19E-93 | 3.57E-92 |  |
| PPIP5K1 | 3.688742 | 1.487094 | -2.20165 | 9.37E-95 | 1.23E-93 |  |
| SH2D5 | 2.932809 | 0.733956 | -2.19885 | 1.67E-58 | 3.78E-58 |  |
| AL031710.1 | 2.44007 | 0.243362 | -2.19671 | 1.63E-87 | 8.47E-87 |  |
| PRMT8 | 2.545043 | 0.348823 | -2.19622 | 5.11E-79 | 1.83E-78 |  |
| CACNB3 | 4.473664 | 2.278375 | -2.19529 | 1.52E-75 | 4.93E-75 |  |
| ATP2B3 | 2.612591 | 0.417692 | -2.1949 | 1.00E-80 | 3.79E-80 |  |
| AC005921.2 | 2.194548 | 0 | -2.19455 | 1.64E-70 | 4.67E-70 |  |
| FP565260.6 | 2.757729 | 0.563626 | -2.1941 | 1.50E-95 | 2.80E-94 |  |
| AL732372.3 | 2.196544 | 0.002868 | -2.19368 | 1.36E-95 | 2.61E-94 |  |
| CAMTA1 | 5.180173 | 2.987211 | -2.19296 | 1.77E-95 | 3.19E-94 |  |
| TMEM235 | 2.965224 | 0.773128 | -2.1921 | 1.68E-66 | 4.39E-66 |  |
| NDUFA5 | 5.946313 | 3.755402 | -2.19091 | 3.29E-95 | 5.21E-94 |  |
| PNMT | 3.066093 | 0.87554 | -2.19055 | 1.22E-65 | 3.12E-65 |  |
| ELMO1 | 5.436153 | 3.24577 | -2.19038 | 5.05E-87 | 2.55E-86 |  |
| ZNF280D | 3.787888 | 1.598134 | -2.18975 | 1.53E-95 | 2.85E-94 |  |
| SLC6A15 | 2.640706 | 0.451082 | -2.18962 | 5.89E-86 | 2.79E-85 |  |
| MPRIP | 6.051534 | 3.862118 | -2.18942 | 1.31E-95 | 2.56E-94 |  |
| AC091057.3 | 2.319645 | 0.130725 | -2.18892 | 1.12E-94 | 1.42E-93 |  |
| MRPL53 | 4.678685 | 2.489812 | -2.18887 | 4.23E-93 | 3.60E-92 |  |
| MIAT | 3.942726 | 1.754009 | -2.18872 | 3.14E-59 | 7.18E-59 |  |
| AC239804.1 | 2.199967 | 0.012026 | -2.18794 | 1.37E-96 | 4.19E-95 |  |
| CYP51A1 | 3.861818 | 1.674217 | -2.1876 | 3.40E-93 | 2.93E-92 |  |
| AC005339.1 | 2.187571 | 0 | -2.18757 | 5.45E-94 | 5.53E-93 |  |
| GRM5-AS1 | 2.187205 | 0.002928 | -2.18428 | 1.76E-96 | 5.07E-95 |  |
| DYNC1H1 | 7.121536 | 4.938524 | -2.18301 | 6.93E-95 | 9.58E-94 |  |
| CARNS1 | 4.213159 | 2.031553 | -2.18161 | 6.91E-44 | 1.27E-43 |  |
| PDZD4 | 6.824027 | 4.643185 | -2.18084 | 4.76E-70 | 1.34E-69 |  |
| AL450306.1 | 2.314891 | 0.137979 | -2.17691 | 3.09E-95 | 4.97E-94 |  |
| ACAP3 | 6.296997 | 4.123112 | -2.17389 | 2.28E-75 | 7.35E-75 |  |
| CBFA2T3 | 2.662385 | 0.488698 | -2.17369 | 3.88E-92 | 2.92E-91 |  |
| SH3GL2 | 5.053397 | 2.879873 | -2.17352 | 9.30E-46 | 1.75E-45 |  |
| SLX1B-SULT1A4 | 2.173562 | 0.001327 | -2.17223 | 5.66E-97 | 2.26E-95 |  |
| PYURF | 4.79287 | 2.620853 | -2.17202 | 9.34E-93 | 7.54E-92 |  |
| ANKRD36 | 2.480854 | 0.31147 | -2.16938 | 2.24E-95 | 3.85E-94 |  |
| CRIP3 | 2.974436 | 0.805362 | -2.16907 | 9.68E-91 | 6.28E-90 |  |
| GALT | 4.54381 | 2.37501 | -2.1688 | 7.56E-95 | 1.03E-93 |  |
| SERF1A | 2.316703 | 0.147987 | -2.16872 | 1.95E-94 | 2.28E-93 |  |
| PI4KA | 5.724659 | 3.556432 | -2.16823 | 5.40E-88 | 2.88E-87 |  |
| PANX2 | 3.711573 | 1.543923 | -2.16765 | 3.38E-80 | 1.26E-79 |  |
| SPNS1 | 3.511974 | 1.344868 | -2.16711 | 1.84E-93 | 1.67E-92 |  |
| GOLGA7B | 3.794022 | 1.627296 | -2.16673 | 1.63E-72 | 4.89E-72 |  |
| PTPRM | 4.253379 | 2.086888 | -2.16649 | 4.35E-87 | 2.21E-86 |  |
| KCNAB1 | 3.271883 | 1.105862 | -2.16602 | 1.44E-87 | 7.48E-87 |  |
| FAM57B | 4.001867 | 1.840428 | -2.16144 | 3.32E-70 | 9.38E-70 |  |
| ACTL6B | 3.993036 | 1.835466 | -2.15757 | 3.45E-50 | 6.89E-50 |  |
| TRAPPC12 | 5.159316 | 3.001915 | -2.1574 | 2.37E-95 | 4.01E-94 |  |
| NEFH | 3.591174 | 1.436786 | -2.15439 | 3.69E-62 | 8.89E-62 |  |
| GDPD5 | 3.203951 | 1.052418 | -2.15153 | 2.20E-92 | 1.69E-91 |  |
| AL355472.3 | 2.388805 | 0.237273 | -2.15153 | 5.80E-88 | 3.08E-87 |  |
| AF129408.1 | 2.15147 | 0 | -2.15147 | 1.27E-94 | 1.57E-93 |  |
| NME2 | 6.943952 | 4.793255 | -2.1507 | 1.29E-92 | 1.02E-91 |  |
| AC010624.2 | 2.973698 | 0.823049 | -2.15065 | 1.13E-87 | 5.88E-87 |  |
| DCTN1 | 6.762388 | 4.613803 | -2.14859 | 3.07E-95 | 4.95E-94 |  |
| SLC26A10 | 2.98552 | 0.837033 | -2.14849 | 1.24E-72 | 3.73E-72 |  |
| LRCH4 | 4.221242 | 2.073307 | -2.14794 | 1.17E-93 | 1.09E-92 |  |
| PCSK6 | 2.93221 | 0.787142 | -2.14507 | 2.42E-76 | 8.02E-76 |  |
| KCNN1 | 3.716359 | 1.571413 | -2.14495 | 1.48E-56 | 3.23E-56 |  |
| CNNM1 | 2.44486 | 0.303319 | -2.14154 | 1.96E-86 | 9.52E-86 |  |
| SYN1 | 5.546297 | 3.405832 | -2.14046 | 2.00E-51 | 4.07E-51 |  |
| SRSF5 | 7.473881 | 5.334882 | -2.139 | 2.97E-94 | 3.29E-93 |  |
| WDR27 | 3.416934 | 1.278249 | -2.13869 | 1.02E-95 | 2.11E-94 |  |
| TMEM191C | 2.262087 | 0.124057 | -2.13803 | 1.32E-95 | 2.57E-94 |  |
| PALM | 6.465873 | 4.328158 | -2.13772 | 2.19E-82 | 8.86E-82 |  |
| GSDMB | 3.239093 | 1.101707 | -2.13739 | 3.81E-90 | 2.35E-89 |  |
| KLHL3 | 2.751069 | 0.61447 | -2.1366 | 1.65E-95 | 3.02E-94 |  |
| CDK10 | 5.420738 | 3.28428 | -2.13646 | 4.61E-92 | 3.43E-91 |  |
| NEDD8 | 6.923172 | 4.786949 | -2.13622 | 2.00E-94 | 2.32E-93 |  |
| PLLP | 5.036202 | 2.899986 | -2.13622 | 3.98E-60 | 9.24E-60 |  |
| TTC4 | 3.326289 | 1.191713 | -2.13458 | 1.12E-94 | 1.42E-93 |  |
| RYR1 | 3.110047 | 0.975936 | -2.13411 | 1.98E-92 | 1.53E-91 |  |
| AL160408.2 | 2.133498 | 0 | -2.1335 | 2.12E-76 | 7.04E-76 |  |
| WNT10B | 2.574035 | 0.440887 | -2.13315 | 5.62E-46 | 1.06E-45 |  |
| AL662899.2 | 2.267318 | 0.135096 | -2.13222 | 5.77E-92 | 4.25E-91 |  |
| KCNMA1 | 3.784924 | 1.653354 | -2.13157 | 4.82E-92 | 3.58E-91 |  |
| ITPR1 | 3.885368 | 1.75407 | -2.1313 | 9.89E-76 | 3.22E-75 |  |
| GAD1 | 4.054359 | 1.923214 | -2.13115 | 2.44E-59 | 5.59E-59 |  |
| PACS2 | 5.885729 | 3.755039 | -2.13069 | 4.20E-93 | 3.57E-92 |  |
| AC132217.1 | 2.127097 | 0 | -2.1271 | 2.02E-95 | 3.55E-94 |  |
| SARS2 | 3.663808 | 1.536876 | -2.12693 | 1.21E-94 | 1.50E-93 |  |
| CACNG3 | 2.789718 | 0.663156 | -2.12656 | 5.94E-53 | 1.23E-52 |  |
| CCDC78 | 3.273528 | 1.147308 | -2.12622 | 6.33E-82 | 2.51E-81 |  |
| CCDC88B | 3.775483 | 1.649534 | -2.12595 | 1.38E-90 | 8.83E-90 |  |
| KCNK4 | 2.151635 | 0.026039 | -2.1256 | 1.55E-94 | 1.87E-93 |  |
| FAAH | 4.401727 | 2.276276 | -2.12545 | 1.65E-86 | 8.06E-86 |  |
| SDHC | 5.627267 | 3.502049 | -2.12522 | 5.72E-94 | 5.76E-93 |  |
| SLC6A7 | 2.451438 | 0.326981 | -2.12446 | 6.15E-56 | 1.33E-55 |  |
| SLC6A13 | 2.469037 | 0.345107 | -2.12393 | 4.53E-92 | 3.37E-91 |  |
| AC004466.1 | 2.240678 | 0.117325 | -2.12335 | 9.67E-96 | 2.02E-94 |  |
| TBCD | 5.365035 | 3.241747 | -2.12329 | 2.24E-95 | 3.85E-94 |  |
| OGFOD2 | 2.724343 | 0.601652 | -2.12269 | 7.36E-95 | 1.01E-93 |  |
| NKX6-2 | 3.917373 | 1.796655 | -2.12072 | 4.40E-46 | 8.31E-46 |  |
| SHANK3 | 4.648223 | 2.528346 | -2.11988 | 1.11E-83 | 4.73E-83 |  |
| LRRC37B | 3.943335 | 1.825664 | -2.11767 | 6.47E-95 | 9.04E-94 |  |
| NR4A1 | 4.445583 | 2.329023 | -2.11656 | 6.53E-61 | 1.54E-60 |  |
| AC004890.2 | 3.256317 | 1.141666 | -2.11465 | 1.42E-91 | 9.98E-91 |  |
| NRXN2 | 5.967032 | 3.852533 | -2.1145 | 4.85E-73 | 1.47E-72 |  |
| C12orf76 | 5.180314 | 3.065994 | -2.11432 | 4.75E-95 | 7.02E-94 |  |
| ASIC3 | 4.196328 | 2.08346 | -2.11287 | 3.44E-83 | 1.44E-82 |  |
| SAMD14 | 4.323357 | 2.210826 | -2.11253 | 4.20E-79 | 1.51E-78 |  |
| SMG1P7 | 3.285508 | 1.173648 | -2.11186 | 6.30E-92 | 4.61E-91 |  |
| LUC7L | 5.853897 | 3.743176 | -2.11072 | 5.71E-93 | 4.75E-92 |  |
| AL139300.1 | 2.112501 | 0.002593 | -2.10991 | 1.56E-72 | 4.67E-72 |  |
| FAM86B1 | 2.945996 | 0.836335 | -2.10966 | 1.02E-93 | 9.77E-93 |  |
| PAAF1 | 4.978318 | 2.868968 | -2.10935 | 2.37E-93 | 2.10E-92 |  |
| MTMR3 | 3.333656 | 1.224539 | -2.10912 | 2.71E-95 | 4.47E-94 |  |
| TOMM5 | 5.148916 | 3.041286 | -2.10763 | 8.17E-94 | 7.95E-93 |  |
| RPL13 | 9.609088 | 7.503116 | -2.10597 | 2.85E-93 | 2.49E-92 |  |
| SCLY | 2.578657 | 0.472723 | -2.10593 | 1.85E-94 | 2.19E-93 |  |
| CLK4 | 4.119123 | 2.014502 | -2.10462 | 5.31E-95 | 7.70E-94 |  |
| NDUFA3 | 7.341699 | 5.237582 | -2.10412 | 3.59E-91 | 2.42E-90 |  |
| LINC02193 | 2.159112 | 0.05551 | -2.1036 | 2.61E-94 | 2.94E-93 |  |
| NPIPB14P | 2.306335 | 0.204363 | -2.10197 | 9.20E-95 | 1.21E-93 |  |
| ATOX1 | 5.886571 | 3.786058 | -2.10051 | 4.61E-91 | 3.07E-90 |  |
| BCRP2 | 2.102809 | 0.003851 | -2.09896 | 5.66E-97 | 2.26E-95 |  |
| C2orf92 | 2.904184 | 0.805973 | -2.09821 | 5.12E-94 | 5.24E-93 |  |
| SH3YL1 | 4.229304 | 2.131145 | -2.09816 | 1.01E-94 | 1.31E-93 |  |
| SEM1 | 5.546619 | 3.449411 | -2.09721 | 6.47E-92 | 4.73E-91 |  |
| XIST | 2.734548 | 0.637371 | -2.09718 | 2.14E-29 | 3.28E-29 |  |
| RNF144A-AS1 | 2.322064 | 0.225525 | -2.09654 | 5.37E-91 | 3.56E-90 |  |
| TNNI3K | 2.166511 | 0.071098 | -2.09541 | 2.54E-96 | 6.75E-95 |  |
| RGS14 | 3.937977 | 1.843975 | -2.094 | 1.53E-30 | 2.37E-30 |  |
| AS3MT | 2.321909 | 0.227909 | -2.094 | 4.32E-95 | 6.51E-94 |  |
| SRGAP2B | 3.150327 | 1.057612 | -2.09271 | 3.44E-93 | 2.96E-92 |  |
| LTO1 | 3.76978 | 1.677524 | -2.09226 | 3.52E-95 | 5.46E-94 |  |
| RNU2-2P | 2.091522 | 0 | -2.09152 | 1.29E-72 | 3.89E-72 |  |
| RELL2 | 3.690534 | 1.599209 | -2.09132 | 2.93E-81 | 1.13E-80 |  |
| NAP1L1 | 6.923823 | 4.833721 | -2.0901 | 4.36E-94 | 4.56E-93 |  |
| RPL12P16 | 2.089733 | 0 | -2.08973 | 5.02E-97 | 2.11E-95 |  |
| H3F3A | 6.976217 | 4.886988 | -2.08923 | 1.42E-93 | 1.31E-92 |  |
| LRRFIP2 | 4.837362 | 2.748536 | -2.08883 | 7.74E-97 | 2.81E-95 |  |
| HNRNPUL2-BSCL2 | 2.341566 | 0.252897 | -2.08867 | 7.34E-73 | 2.22E-72 |  |
| C2CD2L | 4.083351 | 1.996802 | -2.08655 | 1.35E-94 | 1.66E-93 |  |
| SGSM2 | 5.867152 | 3.781199 | -2.08595 | 3.20E-88 | 1.73E-87 |  |
| NADSYN1 | 4.278334 | 2.192414 | -2.08592 | 2.79E-94 | 3.12E-93 |  |
| AES | 8.663771 | 6.57978 | -2.08399 | 5.78E-92 | 4.25E-91 |  |
| EPN1 | 5.846839 | 3.763525 | -2.08331 | 3.59E-94 | 3.84E-93 |  |
| DGKA | 3.834537 | 1.751456 | -2.08308 | 9.33E-94 | 8.99E-93 |  |
| PRMT7 | 4.724138 | 2.641924 | -2.08221 | 4.92E-94 | 5.07E-93 |  |
| CHAD | 2.549893 | 0.469223 | -2.08067 | 3.57E-84 | 1.56E-83 |  |
| AKR1C2 | 2.324838 | 0.244671 | -2.08017 | 1.01E-90 | 6.55E-90 |  |
| RTEL1-TNFRSF6B | 2.502038 | 0.422042 | -2.08 | 2.64E-93 | 2.32E-92 |  |
| FKBP1B | 4.607467 | 2.527523 | -2.07994 | 4.91E-79 | 1.76E-78 |  |
| TAC1 | 3.570844 | 1.493065 | -2.07778 | 1.82E-29 | 2.79E-29 |  |
| PAGR1 | 4.823303 | 2.746274 | -2.07703 | 2.66E-82 | 1.07E-81 |  |
| FAM226B | 2.078327 | 0.002584 | -2.07574 | 1.35E-96 | 4.15E-95 |  |
| ASB3 | 3.283796 | 1.208522 | -2.07527 | 3.15E-94 | 3.44E-93 |  |
| GATB | 4.203806 | 2.128781 | -2.07502 | 2.29E-94 | 2.61E-93 |  |
| GTF2I | 5.118333 | 3.045963 | -2.07237 | 2.47E-93 | 2.18E-92 |  |
| CCDC136 | 4.57464 | 2.503062 | -2.07158 | 2.21E-76 | 7.32E-76 |  |
| SGSM1 | 2.7746 | 0.703341 | -2.07126 | 3.31E-89 | 1.90E-88 |  |
| MCTP1 | 2.809947 | 0.739803 | -2.07014 | 3.35E-88 | 1.81E-87 |  |
| STX1A | 4.805228 | 2.736587 | -2.06864 | 2.03E-47 | 3.89E-47 |  |
| C9orf3 | 3.994388 | 1.925936 | -2.06845 | 3.04E-92 | 2.30E-91 |  |
| AL022313.4 | 2.713003 | 0.645284 | -2.06772 | 5.68E-71 | 1.64E-70 |  |
| ZNRF3 | 2.080959 | 0.013735 | -2.06722 | 4.00E-97 | 1.95E-95 |  |
| ADAM11 | 3.501408 | 1.434629 | -2.06678 | 9.13E-58 | 2.03E-57 |  |
| GTF2H4 | 3.57673 | 1.510124 | -2.06661 | 7.56E-95 | 1.03E-93 |  |
| NOL12 | 3.74121 | 1.676894 | -2.06432 | 7.70E-95 | 1.04E-93 |  |
| AC025423.2 | 2.063842 | 0 | -2.06384 | 9.69E-96 | 2.02E-94 |  |
| APBA1 | 3.858269 | 1.794835 | -2.06343 | 9.41E-88 | 4.92E-87 |  |
| MPPE1 | 4.077941 | 2.015183 | -2.06276 | 1.53E-94 | 1.85E-93 |  |
| ITGA9-AS1 | 2.776754 | 0.714178 | -2.06258 | 2.71E-95 | 4.47E-94 |  |
| TCEAL2 | 6.610989 | 4.549858 | -2.06113 | 6.64E-80 | 2.45E-79 |  |
| MOG | 5.364807 | 3.305915 | -2.05889 | 9.00E-31 | 1.40E-30 |  |
| CHGA | 4.038882 | 1.980227 | -2.05865 | 3.50E-32 | 5.54E-32 |  |
| BRSK1 | 6.106808 | 4.049541 | -2.05727 | 8.47E-87 | 4.22E-86 |  |
| LINC00893 | 2.421468 | 0.364318 | -2.05715 | 2.19E-95 | 3.78E-94 |  |
| CAMK4 | 2.687416 | 0.63066 | -2.05676 | 9.25E-77 | 3.10E-76 |  |
| FAM131C | 3.184835 | 1.128238 | -2.0566 | 4.89E-72 | 1.44E-71 |  |
| TACC2 | 3.360131 | 1.3059 | -2.05423 | 1.14E-93 | 1.08E-92 |  |
| AL359764.1 | 2.87905 | 0.824839 | -2.05421 | 7.99E-81 | 3.04E-80 |  |
| AC245033.3 | 2.768395 | 0.715428 | -2.05297 | 3.75E-69 | 1.04E-68 |  |
| MDH1 | 7.435941 | 5.38302 | -2.05292 | 3.28E-92 | 2.48E-91 |  |
| RPL27A | 8.764509 | 6.712102 | -2.05241 | 4.17E-93 | 3.56E-92 |  |
| FAIM2 | 6.784747 | 4.734327 | -2.05042 | 7.89E-81 | 3.00E-80 |  |
| PLPP2 | 3.124924 | 1.076218 | -2.04871 | 2.06E-69 | 5.71E-69 |  |
| STXBP6 | 2.935239 | 0.886644 | -2.0486 | 8.54E-63 | 2.08E-62 |  |
| RPS23 | 9.342795 | 7.294247 | -2.04855 | 4.41E-94 | 4.60E-93 |  |
| ARMCX7P | 2.356625 | 0.308293 | -2.04833 | 8.06E-84 | 3.46E-83 |  |
| NDUFA10 | 5.934704 | 3.886478 | -2.04823 | 1.16E-95 | 2.35E-94 |  |
| HIST2H2AA4 | 2.457405 | 0.409867 | -2.04754 | 9.38E-52 | 1.91E-51 |  |
| CHORDC1 | 3.771926 | 1.724447 | -2.04748 | 2.64E-95 | 4.39E-94 |  |
| FRY | 3.586042 | 1.538905 | -2.04714 | 6.17E-91 | 4.07E-90 |  |
| ZNF692 | 5.266299 | 3.219217 | -2.04708 | 9.80E-86 | 4.60E-85 |  |
| VAMP1 | 4.61715 | 2.572456 | -2.04469 | 1.59E-83 | 6.75E-83 |  |
| CLASRP | 5.449365 | 3.407288 | -2.04208 | 3.63E-94 | 3.89E-93 |  |
| SCAPER | 3.68197 | 1.641062 | -2.04091 | 1.17E-95 | 2.36E-94 |  |
| NKAIN2 | 3.28855 | 1.25022 | -2.03833 | 2.32E-66 | 6.01E-66 |  |
| CCM2 | 5.601853 | 3.563878 | -2.03798 | 4.17E-94 | 4.38E-93 |  |
| ACSBG1 | 4.274993 | 2.238182 | -2.03681 | 3.93E-53 | 8.17E-53 |  |
| SLC7A10 | 2.659602 | 0.623618 | -2.03598 | 9.70E-69 | 2.65E-68 |  |
| KNDC1 | 3.904824 | 1.869242 | -2.03558 | 1.67E-74 | 5.28E-74 |  |
| MYO18A | 4.913441 | 2.878488 | -2.03495 | 2.62E-95 | 4.36E-94 |  |
| FAM47E | 2.58327 | 0.549381 | -2.03389 | 3.35E-90 | 2.08E-89 |  |
| BTNL9 | 2.92647 | 0.895151 | -2.03132 | 2.35E-82 | 9.48E-82 |  |
| RBM5 | 5.853081 | 3.821965 | -2.03112 | 1.90E-92 | 1.47E-91 |  |
| SPIRE2 | 3.51294 | 1.481928 | -2.03101 | 6.53E-87 | 3.28E-86 |  |
| DMXL2 | 4.193652 | 2.163869 | -2.02978 | 3.95E-95 | 6.06E-94 |  |
| AC009084.2 | 2.174257 | 0.14585 | -2.02841 | 9.00E-95 | 1.19E-93 |  |
| STAG3L3 | 2.941696 | 0.913482 | -2.02821 | 5.14E-94 | 5.25E-93 |  |
| MAP3K10 | 5.100841 | 3.074252 | -2.02659 | 6.44E-87 | 3.24E-86 |  |
| KIAA0513 | 4.596239 | 2.570636 | -2.0256 | 2.40E-71 | 6.97E-71 |  |
| ANKRD13D | 4.984385 | 2.959937 | -2.02445 | 4.60E-95 | 6.86E-94 |  |
| ATP1A3 | 6.169085 | 4.145022 | -2.02406 | 8.57E-41 | 1.51E-40 |  |
| ASRGL1 | 5.175214 | 3.152189 | -2.02302 | 1.14E-92 | 9.13E-92 |  |
| DNAAF1 | 2.472915 | 0.450433 | -2.02248 | 1.77E-82 | 7.19E-82 |  |
| ASPSCR1 | 4.225603 | 2.204321 | -2.02128 | 1.41E-93 | 1.30E-92 |  |
| SMYD3 | 3.392322 | 1.373168 | -2.01915 | 2.03E-94 | 2.35E-93 |  |
| SRRM2 | 7.452158 | 5.433143 | -2.01901 | 2.63E-85 | 1.21E-84 |  |
| AL049839.2 | 3.197247 | 1.180438 | -2.01681 | 1.86E-31 | 2.92E-31 |  |
| PHF24 | 3.29897 | 1.283154 | -2.01582 | 2.16E-62 | 5.23E-62 |  |
| NAP1L2 | 4.363219 | 2.350465 | -2.01275 | 6.96E-72 | 2.05E-71 |  |
| NEBL | 4.547204 | 2.535806 | -2.0114 | 1.36E-86 | 6.70E-86 |  |
| ABCA7 | 3.300199 | 1.290346 | -2.00985 | 3.96E-88 | 2.13E-87 |  |
| MCRIP2 | 4.719269 | 2.709666 | -2.0096 | 2.58E-91 | 1.76E-90 |  |
| DEGS2 | 2.407313 | 0.403046 | -2.00427 | 3.57E-92 | 2.69E-91 |  |
| MMP24OS | 5.486207 | 3.48325 | -2.00296 | 2.22E-94 | 2.54E-93 |  |
| TTR | 2.137724 | 0.134772 | -2.00295 | 1.37E-70 | 3.89E-70 |  |
| SCN2A | 3.328526 | 1.32599 | -2.00254 | 5.28E-66 | 1.36E-65 |  |
| ZNF276 | 4.117238 | 2.114781 | -2.00246 | 2.43E-93 | 2.16E-92 |  |
| NEGR1 | 2.868898 | 0.867075 | -2.00182 | 9.64E-82 | 3.79E-81 |  |
| SPEG | 4.327263 | 2.326075 | -2.00119 | 6.80E-87 | 3.41E-86 |  |
| NUMA1 | 5.878264 | 3.877543 | -2.00072 | 6.57E-94 | 6.50E-93 |  |
| NMNAT2 | 3.885921 | 1.885845 | -2.00008 | 6.74E-54 | 1.42E-53 |  |
| CDK6 | 1.21074 | 3.210955 | 2.000215 | 7.77E-82 | 3.07E-81 |  |
| LRRN1 | 2.520813 | 4.521139 | 2.000326 | 4.04E-78 | 1.41E-77 |  |
| LGALS3 | 4.574411 | 6.575796 | 2.001385 | 6.42E-55 | 1.37E-54 |  |
| DCAF12 | 2.376686 | 4.380814 | 2.004128 | 1.22E-97 | 1.61E-95 |  |
| UBALD2 | 3.817587 | 5.822172 | 2.004585 | 7.23E-93 | 5.94E-92 |  |
| CTHRC1 | 1.05017 | 3.055115 | 2.004946 | 1.28E-72 | 3.85E-72 |  |
| MIPEPP3 | 0.429259 | 2.435941 | 2.006682 | 2.92E-97 | 1.79E-95 |  |
| MNDA | 1.27259 | 3.27931 | 2.00672 | 2.54E-79 | 9.21E-79 |  |
| SPRY4 | 2.228463 | 4.236078 | 2.007615 | 2.36E-68 | 6.40E-68 |  |
| P4HA1 | 2.973239 | 4.981151 | 2.007913 | 4.79E-84 | 2.08E-83 |  |
| LDHAP4 | 1.035982 | 3.044794 | 2.008812 | 3.95E-89 | 2.26E-88 |  |
| KIRREL1 | 0.968937 | 2.978777 | 2.00984 | 9.71E-94 | 9.33E-93 |  |
| AP002784.2 | 0.410601 | 2.42223 | 2.011629 | 1.27E-97 | 1.61E-95 |  |
| RNF139 | 2.769275 | 4.781197 | 2.011922 | 2.03E-97 | 1.61E-95 |  |
| MT2A | 7.255944 | 9.268088 | 2.012144 | 7.88E-59 | 1.79E-58 |  |
| PLOD1 | 3.740184 | 5.75375 | 2.013566 | 1.71E-91 | 1.19E-90 |  |
| JAGN1 | 3.14233 | 5.156422 | 2.014092 | 1.29E-97 | 1.61E-95 |  |
| PPP1R18 | 2.992022 | 5.006807 | 2.014785 | 2.03E-88 | 1.12E-87 |  |
| LSM2 | 4.146744 | 6.16182 | 2.015075 | 1.96E-97 | 1.61E-95 |  |
| OST4 | 6.203967 | 8.219404 | 2.015437 | 1.02E-96 | 3.39E-95 |  |
| PRMT6 | 2.140258 | 4.155785 | 2.015528 | 1.98E-97 | 1.61E-95 |  |
| CSF1 | 2.638567 | 4.654277 | 2.015711 | 1.61E-81 | 6.28E-81 |  |
| STAT1 | 3.499641 | 5.517081 | 2.017439 | 7.64E-91 | 4.99E-90 |  |
| SLC35F6 | 2.647065 | 4.664878 | 2.017813 | 1.90E-97 | 1.61E-95 |  |
| AL009174.1 | 1.060057 | 3.079057 | 2.019 | 7.63E-95 | 1.04E-93 |  |
| FTH1P7 | 1.983479 | 4.002641 | 2.019162 | 2.28E-86 | 1.10E-85 |  |
| TGFB2-AS1 | 0.476741 | 2.498002 | 2.02126 | 4.84E-93 | 4.08E-92 |  |
| IFI35 | 2.777122 | 4.799511 | 2.022388 | 1.96E-79 | 7.12E-79 |  |
| TMEM97 | 2.039962 | 4.06238 | 2.022418 | 2.73E-95 | 4.48E-94 |  |
| SF3B5 | 5.402069 | 7.425624 | 2.023555 | 1.74E-97 | 1.61E-95 |  |
| TIMELESS | 1.382109 | 3.4065 | 2.024391 | 5.75E-94 | 5.78E-93 |  |
| RBBP9 | 1.960659 | 3.986655 | 2.025996 | 6.58E-97 | 2.52E-95 |  |
| WEE1 | 1.096526 | 3.122927 | 2.026402 | 1.04E-92 | 8.35E-92 |  |
| DOK5 | 2.478578 | 4.506662 | 2.028084 | 6.44E-79 | 2.30E-78 |  |
| CENPH | 1.133397 | 3.161595 | 2.028197 | 2.01E-95 | 3.53E-94 |  |
| TMED2 | 4.252291 | 6.280508 | 2.028216 | 1.36E-97 | 1.61E-95 |  |
| NPM3 | 2.560068 | 4.589885 | 2.029817 | 2.58E-94 | 2.91E-93 |  |
| CA12 | 1.764453 | 3.794568 | 2.030115 | 1.70E-45 | 3.17E-45 |  |
| INAFM2 | 3.23622 | 5.266496 | 2.030276 | 7.49E-96 | 1.62E-94 |  |
| NEK2 | 0.328284 | 2.360659 | 2.032375 | 1.07E-90 | 6.91E-90 |  |
| RCC1 | 1.667544 | 3.700667 | 2.033123 | 5.01E-94 | 5.14E-93 |  |
| WDR34 | 3.21495 | 5.251864 | 2.036913 | 2.41E-94 | 2.73E-93 |  |
| FUCA2 | 2.826157 | 4.863211 | 2.037054 | 2.73E-94 | 3.06E-93 |  |
| TAP1 | 3.051003 | 5.089418 | 2.038415 | 2.15E-82 | 8.69E-82 |  |
| CAPG | 4.047283 | 6.085738 | 2.038454 | 3.86E-72 | 1.14E-71 |  |
| TIMP2 | 5.321158 | 7.359621 | 2.038463 | 6.33E-83 | 2.62E-82 |  |
| OAS3 | 1.465785 | 3.505385 | 2.0396 | 7.58E-84 | 3.26E-83 |  |
| ARHGEF6 | 2.988429 | 5.028994 | 2.040565 | 8.18E-89 | 4.59E-88 |  |
| PIK3AP1 | 0.732749 | 2.774715 | 2.041967 | 1.89E-86 | 9.23E-86 |  |
| PPIL1 | 3.628578 | 5.675499 | 2.046921 | 2.95E-97 | 1.79E-95 |  |
| ADGRE5 | 1.812504 | 3.860332 | 2.047829 | 1.38E-83 | 5.86E-83 |  |
| BCAT1 | 1.55088 | 3.600256 | 2.049376 | 2.78E-72 | 8.27E-72 |  |
| AC068535.1 | 0.492918 | 2.542803 | 2.049885 | 4.10E-82 | 1.64E-81 |  |
| CDT1 | 0.845586 | 2.898745 | 2.053159 | 2.51E-92 | 1.92E-91 |  |
| ADAM9 | 3.223938 | 5.277896 | 2.053958 | 8.39E-93 | 6.81E-92 |  |
| GANAB | 4.900696 | 6.955444 | 2.054748 | 1.61E-97 | 1.61E-95 |  |
| ZYX | 5.195882 | 7.251663 | 2.055781 | 4.20E-85 | 1.91E-84 |  |
| RAB31 | 4.692602 | 6.749219 | 2.056618 | 2.03E-87 | 1.05E-86 |  |
| NCF4 | 1.290885 | 3.348759 | 2.057874 | 9.90E-81 | 3.75E-80 |  |
| FTH1P11 | 1.637239 | 3.695903 | 2.058665 | 1.39E-85 | 6.46E-85 |  |
| CLCF1 | 0.520086 | 2.580351 | 2.060265 | 3.03E-84 | 1.33E-83 |  |
| JAG1 | 2.161845 | 4.222459 | 2.060615 | 3.16E-86 | 1.52E-85 |  |
| OSMR | 1.682987 | 3.746864 | 2.063877 | 1.67E-67 | 4.44E-67 |  |
| OIP5 | 0.245695 | 2.309764 | 2.064069 | 1.71E-97 | 1.61E-95 |  |
| ACLY | 3.795988 | 5.861763 | 2.065775 | 3.78E-96 | 9.28E-95 |  |
| EZR | 4.462454 | 6.5283 | 2.065846 | 4.70E-68 | 1.26E-67 |  |
| WASF2 | 3.392078 | 5.459119 | 2.067041 | 1.91E-94 | 2.23E-93 |  |
| E2F1 | 1.853698 | 3.92311 | 2.069411 | 4.97E-85 | 2.25E-84 |  |
| LRP10 | 3.161786 | 5.231207 | 2.069421 | 1.57E-89 | 9.25E-89 |  |
| RAC1P2 | 1.700939 | 3.772269 | 2.07133 | 1.38E-92 | 1.08E-91 |  |
| MFNG | 1.451075 | 3.522601 | 2.071526 | 2.89E-89 | 1.68E-88 |  |
| PCLAF | 0.638882 | 2.710743 | 2.071861 | 7.36E-88 | 3.88E-87 |  |
| PLEKHF2 | 1.506032 | 3.577981 | 2.071949 | 4.54E-97 | 2.11E-95 |  |
| B3GNT9 | 1.554215 | 3.627087 | 2.072872 | 3.20E-89 | 1.84E-88 |  |
| CENPF | 0.482244 | 2.556738 | 2.074494 | 1.63E-88 | 9.01E-88 |  |
| LOXL3 | 1.526152 | 3.600744 | 2.074592 | 5.64E-97 | 2.26E-95 |  |
| SAMD9 | 0.372634 | 2.449393 | 2.076759 | 6.90E-94 | 6.81E-93 |  |
| FN1 | 4.675523 | 6.752638 | 2.077115 | 6.20E-69 | 1.70E-68 |  |
| TRIB2 | 3.653158 | 5.730946 | 2.077788 | 4.68E-84 | 2.03E-83 |  |
| ELOVL2 | 2.412592 | 4.49135 | 2.078757 | 2.95E-50 | 5.89E-50 |  |
| S1PR3 | 1.585931 | 3.665779 | 2.079848 | 3.90E-83 | 1.63E-82 |  |
| CD24 | 2.270565 | 4.352555 | 2.081989 | 6.55E-35 | 1.07E-34 |  |
| MTPN | 5.039277 | 7.122026 | 2.08275 | 2.33E-95 | 3.97E-94 |  |
| RPL35P2 | 0.523102 | 2.606696 | 2.083594 | 1.47E-97 | 1.61E-95 |  |
| P2RY12 | 1.926648 | 4.01038 | 2.083731 | 2.29E-51 | 4.65E-51 |  |
| ST14 | 0.500086 | 2.585436 | 2.08535 | 7.53E-88 | 3.96E-87 |  |
| BCL2A1 | 0.838906 | 2.925296 | 2.086391 | 2.36E-68 | 6.40E-68 |  |
| NDC1 | 1.278367 | 3.366588 | 2.088221 | 1.39E-97 | 1.61E-95 |  |
| SAMHD1 | 2.86003 | 4.949573 | 2.089543 | 5.19E-95 | 7.58E-94 |  |
| TGIF2 | 1.053606 | 3.143432 | 2.089826 | 1.01E-96 | 3.35E-95 |  |
| RRP9 | 2.682685 | 4.772918 | 2.090233 | 1.46E-97 | 1.61E-95 |  |
| HILPDA | 3.080642 | 5.171178 | 2.090535 | 2.30E-58 | 5.18E-58 |  |
| RGS19 | 2.211209 | 4.301753 | 2.090544 | 2.28E-94 | 2.60E-93 |  |
| ITGAV | 3.470468 | 5.561374 | 2.090906 | 1.04E-94 | 1.33E-93 |  |
| FCGR2A | 2.039969 | 4.130966 | 2.090998 | 1.46E-60 | 3.42E-60 |  |
| HLA-DQB1 | 2.26802 | 4.359648 | 2.091628 | 9.21E-52 | 1.88E-51 |  |
| SLC35F2 | 0.982065 | 3.074942 | 2.092877 | 1.26E-94 | 1.56E-93 |  |
| GRIK3 | 1.75397 | 3.84744 | 2.09347 | 7.94E-66 | 2.04E-65 |  |
| PLEK | 1.429568 | 3.523768 | 2.0942 | 3.97E-76 | 1.31E-75 |  |
| ZFP36 | 4.361187 | 6.455974 | 2.094787 | 4.92E-51 | 9.94E-51 |  |
| C1orf226 | 1.266363 | 3.361708 | 2.095344 | 3.46E-90 | 2.14E-89 |  |
| EEF1AKMT3 | 1.112145 | 3.207981 | 2.095836 | 4.43E-96 | 1.04E-94 |  |
| ZNF436 | 2.213307 | 4.309483 | 2.096176 | 1.65E-96 | 4.85E-95 |  |
| IER5 | 2.098565 | 4.195747 | 2.097182 | 4.21E-87 | 2.14E-86 |  |
| HCLS1 | 2.242351 | 4.340496 | 2.098145 | 1.15E-67 | 3.07E-67 |  |
| PPT1 | 5.107953 | 7.207853 | 2.099901 | 1.59E-96 | 4.70E-95 |  |
| ALYREF | 3.895706 | 5.995715 | 2.100009 | 7.77E-97 | 2.82E-95 |  |
| MAML2 | 1.451995 | 3.552869 | 2.100874 | 8.17E-97 | 2.87E-95 |  |
| SPTSSA | 3.066363 | 5.168111 | 2.101747 | 1.66E-96 | 4.86E-95 |  |
| TNFRSF1B | 2.11875 | 4.221965 | 2.103215 | 5.04E-74 | 1.57E-73 |  |
| DAP | 3.845913 | 5.95082 | 2.104908 | 1.72E-97 | 1.61E-95 |  |
| PTGFRN | 2.667358 | 4.77306 | 2.105702 | 7.06E-88 | 3.73E-87 |  |
| AL591846.1 | 1.870041 | 3.976133 | 2.106092 | 4.04E-28 | 6.10E-28 |  |
| CD276 | 3.043702 | 5.151202 | 2.1075 | 5.98E-97 | 2.34E-95 |  |
| ATP6V0E1 | 5.343305 | 7.451393 | 2.108088 | 9.47E-95 | 1.24E-93 |  |
| GINS2 | 0.880733 | 2.990786 | 2.110053 | 2.61E-92 | 1.99E-91 |  |
| ABHD4 | 2.926151 | 5.037265 | 2.111114 | 1.04E-94 | 1.34E-93 |  |
| AL391069.2 | 1.158809 | 3.274635 | 2.115825 | 3.01E-95 | 4.86E-94 |  |
| KDELC2 | 1.597259 | 3.714954 | 2.117695 | 2.74E-91 | 1.87E-90 |  |
| MUL1 | 2.914092 | 5.031963 | 2.117872 | 3.04E-97 | 1.79E-95 |  |
| PLA2G4A | 0.891552 | 3.012929 | 2.121377 | 2.51E-92 | 1.92E-91 |  |
| MS4A6A | 2.075269 | 4.19713 | 2.121861 | 1.86E-57 | 4.12E-57 |  |
| SERTAD1 | 2.040782 | 4.162697 | 2.121915 | 8.38E-87 | 4.18E-86 |  |
| RPN1 | 4.705298 | 6.827539 | 2.12224 | 1.32E-97 | 1.61E-95 |  |
| FAM20C | 3.663316 | 5.785738 | 2.122422 | 2.34E-83 | 9.84E-83 |  |
| SLC31A1 | 1.983644 | 4.107755 | 2.124111 | 1.79E-97 | 1.61E-95 |  |
| NOTCH1 | 2.287253 | 4.411774 | 2.124521 | 9.04E-86 | 4.25E-85 |  |
| SLC39A14 | 2.399384 | 4.524459 | 2.125076 | 4.70E-81 | 1.80E-80 |  |
| LMAN2 | 4.281792 | 6.40891 | 2.127119 | 1.70E-97 | 1.61E-95 |  |
| RPL18AP3 | 3.854272 | 5.982598 | 2.128326 | 3.13E-93 | 2.71E-92 |  |
| TUBA1A | 8.390776 | 10.52173 | 2.130951 | 7.41E-81 | 2.82E-80 |  |
| GPR183 | 1.06216 | 3.193664 | 2.131504 | 6.67E-67 | 1.75E-66 |  |
| RARRES3 | 3.740731 | 5.87247 | 2.13174 | 2.12E-62 | 5.12E-62 |  |
| TNFSF13B | 1.020486 | 3.153116 | 2.13263 | 9.78E-88 | 5.11E-87 |  |
| H2AFX | 4.216272 | 6.349345 | 2.133073 | 2.38E-96 | 6.39E-95 |  |
| BTG3 | 2.870435 | 5.004045 | 2.13361 | 7.48E-91 | 4.89E-90 |  |
| FAM181B | 2.93134 | 5.065584 | 2.134244 | 1.13E-74 | 3.58E-74 |  |
| UBE2T | 2.466616 | 4.602816 | 2.1362 | 6.96E-85 | 3.14E-84 |  |
| NCAN | 3.855189 | 5.994002 | 2.138813 | 1.96E-47 | 3.77E-47 |  |
| CASP3 | 2.736247 | 4.875117 | 2.13887 | 2.28E-96 | 6.18E-95 |  |
| S100A3 | 0.502413 | 2.643526 | 2.141113 | 1.46E-84 | 6.48E-84 |  |
| THBS2 | 2.039379 | 4.180665 | 2.141285 | 1.34E-84 | 5.98E-84 |  |
| DPYD | 1.224738 | 3.368054 | 2.143316 | 5.49E-82 | 2.18E-81 |  |
| HOTAIRM1 | 0.582443 | 2.725787 | 2.143345 | 4.64E-71 | 1.34E-70 |  |
| DRAXIN | 0.496289 | 2.641007 | 2.144718 | 9.06E-86 | 4.26E-85 |  |
| LDLRAD3 | 2.544575 | 4.692129 | 2.147554 | 2.69E-92 | 2.05E-91 |  |
| LPL | 3.13931 | 5.288384 | 2.149074 | 2.78E-57 | 6.14E-57 |  |
| MACORIS | 0.955519 | 3.104833 | 2.149314 | 8.48E-83 | 3.48E-82 |  |
| CD58 | 1.673601 | 3.82347 | 2.149868 | 3.30E-89 | 1.90E-88 |  |
| DAD1 | 6.118397 | 8.268363 | 2.149965 | 2.07E-97 | 1.61E-95 |  |
| TLR2 | 1.101056 | 3.251657 | 2.150601 | 1.32E-80 | 4.96E-80 |  |
| ARHGAP18 | 1.024148 | 3.17584 | 2.151692 | 7.66E-93 | 6.26E-92 |  |
| RPS29P16 | 0.525364 | 2.678031 | 2.152667 | ###### | ###### |  |
| SDC3 | 5.080094 | 7.234666 | 2.154572 | 6.56E-77 | 2.21E-76 |  |
| IGSF6 | 0.749975 | 2.905263 | 2.155288 | 3.31E-90 | 2.05E-89 |  |
| LPAR5 | 1.030036 | 3.185555 | 2.155519 | 4.68E-84 | 2.03E-83 |  |
| JUN | 4.715887 | 6.873237 | 2.15735 | 6.14E-79 | 2.20E-78 |  |
| MYD88 | 2.081425 | 4.239644 | 2.15822 | 1.34E-92 | 1.05E-91 |  |
| IDH1 | 3.23704 | 5.395393 | 2.158352 | 3.20E-97 | 1.81E-95 |  |
| TNFRSF21 | 3.364532 | 5.523209 | 2.158677 | 5.15E-78 | 1.79E-77 |  |
| FZD7 | 1.877878 | 4.038334 | 2.160456 | 2.79E-56 | 6.08E-56 |  |
| CDCA4 | 0.849929 | 3.012166 | 2.162237 | 1.50E-97 | 1.61E-95 |  |
| CHPF | 4.152889 | 6.316918 | 2.16403 | 4.32E-95 | 6.51E-94 |  |
| GPC4 | 1.378817 | 3.544221 | 2.165404 | 7.23E-93 | 5.94E-92 |  |
| EIF3I | 5.515123 | 7.681864 | 2.166741 | 1.26E-97 | 1.61E-95 |  |
| SH3PXD2B | 2.071459 | 4.238432 | 2.166973 | 1.03E-90 | 6.66E-90 |  |
| CENPW | 0.979511 | 3.14714 | 2.167629 | 1.06E-94 | 1.36E-93 |  |
| Z74021.1 | 1.174327 | 3.343327 | 2.169 | 1.60E-92 | 1.25E-91 |  |
| SIGLEC8 | 0.911895 | 3.081823 | 2.169928 | 5.21E-77 | 1.76E-76 |  |
| NUF2 | 0.445487 | 2.616629 | 2.171142 | 6.44E-95 | 9.02E-94 |  |
| TMEM60 | 2.814728 | 4.98687 | 2.172141 | 2.10E-97 | 1.61E-95 |  |
| SALL1 | 2.476126 | 4.648867 | 2.172741 | 1.27E-82 | 5.18E-82 |  |
| GEM | 1.969795 | 4.143299 | 2.173504 | 2.66E-74 | 8.38E-74 |  |
| FAM57A | 2.061085 | 4.235596 | 2.174511 | 2.97E-97 | 1.79E-95 |  |
| CDK2 | 1.653654 | 3.828724 | 2.17507 | 8.02E-93 | 6.53E-92 |  |
| PDLIM1 | 2.138054 | 4.316585 | 2.178531 | 9.21E-68 | 2.47E-67 |  |
| ETV4 | 0.650375 | 2.82941 | 2.179035 | 1.11E-77 | 3.81E-77 |  |
| TMIGD3 | 2.107947 | 4.291083 | 2.183136 | 7.59E-67 | 1.99E-66 |  |
| RGS16 | 2.004815 | 4.188074 | 2.183259 | 6.32E-74 | 1.97E-73 |  |
| SEMA5A | 1.519592 | 3.703789 | 2.184197 | 1.06E-83 | 4.51E-83 |  |
| PPIAP31 | 0.982922 | 3.167329 | 2.184407 | 2.78E-97 | 1.79E-95 |  |
| SNORA33 | 0.277527 | 2.46344 | 2.185912 | ###### | ###### |  |
| CNPY3 | 4.033084 | 6.220022 | 2.186938 | 2.23E-97 | 1.62E-95 |  |
| GADD45A | 3.652315 | 5.839945 | 2.18763 | 1.22E-68 | 3.33E-68 |  |
| FTH1P2 | 2.074896 | 4.262744 | 2.187847 | 1.77E-86 | 8.65E-86 |  |
| AC008026.1 | 0.346863 | 2.535551 | 2.188687 | 2.34E-97 | 1.65E-95 |  |
| EVI2B | 1.49379 | 3.68278 | 2.18899 | 1.07E-77 | 3.66E-77 |  |
| FSCN1 | 5.020339 | 7.210863 | 2.190524 | 1.77E-83 | 7.48E-83 |  |
| ALDH1B1 | 1.415337 | 3.605868 | 2.190531 | 1.13E-96 | 3.60E-95 |  |
| NOP10 | 5.306076 | 7.496725 | 2.190649 | 1.72E-97 | 1.61E-95 |  |
| NCF2 | 1.017927 | 3.209099 | 2.191173 | 3.90E-86 | 1.87E-85 |  |
| CAPN5 | 2.534281 | 4.727189 | 2.192908 | 4.88E-95 | 7.18E-94 |  |
| CENPM | 0.705448 | 2.899271 | 2.193823 | 3.89E-93 | 3.34E-92 |  |
| CTSK | 1.937224 | 4.131507 | 2.194282 | 7.97E-84 | 3.42E-83 |  |
| KIF11 | 0.345977 | 2.540608 | 2.194631 | 1.60E-97 | 1.61E-95 |  |
| LIF | 0.387197 | 2.582023 | 2.194826 | 3.47E-77 | 1.18E-76 |  |
| RND3 | 1.984761 | 4.180277 | 2.195516 | 4.81E-85 | 2.19E-84 |  |
| RHOA | 6.51266 | 8.710179 | 2.19752 | 1.65E-97 | 1.61E-95 |  |
| ELK3 | 1.728779 | 3.932009 | 2.20323 | 3.57E-95 | 5.52E-94 |  |
| TMEM158 | 3.232793 | 5.438303 | 2.20551 | 6.25E-56 | 1.35E-55 |  |
| IGSF3 | 1.002089 | 3.207672 | 2.205583 | 4.21E-91 | 2.82E-90 |  |
| LHFPL3 | 2.094209 | 4.300808 | 2.2066 | 6.66E-42 | 1.19E-41 |  |
| AL133260.1 | 0.68586 | 2.893162 | 2.207302 | 1.47E-94 | 1.79E-93 |  |
| SNX7 | 2.174043 | 4.381514 | 2.20747 | 1.50E-95 | 2.81E-94 |  |
| AHR | 0.971522 | 3.179381 | 2.20786 | 8.94E-88 | 4.69E-87 |  |
| OSTC | 3.641592 | 5.849765 | 2.208173 | 3.41E-97 | 1.85E-95 |  |
| SF3B4 | 3.472053 | 5.680888 | 2.208835 | 1.41E-97 | 1.61E-95 |  |
| CD300A | 1.538877 | 3.748741 | 2.209864 | 3.53E-80 | 1.31E-79 |  |
| LRRN4CL | 0.396983 | 2.608132 | 2.211149 | 9.01E-88 | 4.72E-87 |  |
| KDELR1 | 4.601256 | 6.814608 | 2.213352 | 1.24E-97 | 1.61E-95 |  |
| VAT1 | 4.311512 | 6.525372 | 2.213859 | 3.25E-88 | 1.76E-87 |  |
| SLC16A1 | 3.170247 | 5.386957 | 2.21671 | 3.58E-83 | 1.49E-82 |  |
| RGS10 | 2.77382 | 4.991192 | 2.217372 | 1.58E-70 | 4.48E-70 |  |
| AC020898.1 | 0.281783 | 2.499208 | 2.217425 | 1.89E-97 | 1.61E-95 |  |
| SUMO2P1 | 0.782507 | 3.002211 | 2.219704 | 6.23E-98 | 1.61E-95 |  |
| MCM3 | 2.910335 | 5.130726 | 2.22039 | 1.65E-97 | 1.61E-95 |  |
| GNB4 | 1.746081 | 3.96718 | 2.221099 | 5.19E-97 | 2.15E-95 |  |
| MSI1 | 2.046337 | 4.267705 | 2.221368 | 3.66E-92 | 2.75E-91 |  |
| MGP | 3.29087 | 5.512531 | 2.221661 | 1.12E-58 | 2.54E-58 |  |
| ISG15 | 4.175802 | 6.399071 | 2.223268 | 7.10E-62 | 1.70E-61 |  |
| RPL13AP5 | 4.369763 | 6.5948 | 2.225037 | 1.93E-93 | 1.74E-92 |  |
| PRR11 | 0.544643 | 2.771362 | 2.226719 | 2.52E-97 | 1.72E-95 |  |
| NAMPT | 3.415373 | 5.645858 | 2.230485 | 2.58E-69 | 7.16E-69 |  |
| EGR2 | 0.807769 | 3.039203 | 2.231434 | 1.49E-81 | 5.81E-81 |  |
| SOAT1 | 1.367392 | 3.599978 | 2.232586 | 2.05E-96 | 5.73E-95 |  |
| CHPF2 | 2.481747 | 4.718196 | 2.236448 | 2.87E-97 | 1.79E-95 |  |
| OLR1 | 1.772107 | 4.00865 | 2.236543 | 1.54E-60 | 3.60E-60 |  |
| MTCO3P12 | 2.648068 | 4.88486 | 2.236792 | 1.51E-74 | 4.77E-74 |  |
| PYGL | 2.235801 | 4.473036 | 2.237235 | 2.31E-85 | 1.06E-84 |  |
| PTGES3P1 | 2.32654 | 4.564322 | 2.237782 | 3.62E-96 | 8.92E-95 |  |
| AGAP2-AS1 | 1.511127 | 3.75017 | 2.239042 | 2.27E-77 | 7.74E-77 |  |
| MTCO2P12 | 2.452101 | 4.691526 | 2.239425 | 5.77E-83 | 2.39E-82 |  |
| HLA-DMB | 2.171033 | 4.41073 | 2.239697 | 2.57E-67 | 6.83E-67 |  |
| ILF2 | 5.013382 | 7.254859 | 2.241477 | 2.18E-97 | 1.62E-95 |  |
| GPSM3 | 2.30602 | 4.547636 | 2.241617 | 3.82E-86 | 1.83E-85 |  |
| RPE65 | 0.363312 | 2.610568 | 2.247256 | 5.98E-84 | 2.59E-83 |  |
| LINC01736 | 1.395027 | 3.642714 | 2.247687 | 4.16E-68 | 1.12E-67 |  |
| NFIA-AS2 | 0.549829 | 2.79759 | 2.247761 | 3.72E-90 | 2.30E-89 |  |
| AL354892.2 | 0.410895 | 2.660713 | 2.249819 | 1.10E-96 | 3.56E-95 |  |
| CHCHD2 | 7.306317 | 9.557191 | 2.250874 | 1.32E-97 | 1.61E-95 |  |
| NPM1P27 | 1.544014 | 3.794909 | 2.250894 | 5.34E-97 | 2.19E-95 |  |
| SLC2A10 | 0.985609 | 3.236563 | 2.250954 | 1.55E-88 | 8.57E-88 |  |
| HIST1H4I | 0.243537 | 2.49473 | 2.251194 | 3.99E-97 | 1.95E-95 |  |
| RANP1 | 0.235357 | 2.488844 | 2.253487 | 7.08E-98 | 1.61E-95 |  |
| HCK | 1.397958 | 3.651928 | 2.25397 | 1.07E-81 | 4.21E-81 |  |
| APBB1IP | 1.447264 | 3.701578 | 2.254314 | 2.55E-78 | 8.97E-78 |  |
| CEBPD | 3.313977 | 5.568379 | 2.254401 | 9.71E-68 | 2.60E-67 |  |
| CA9 | 0.976842 | 3.232065 | 2.255224 | 1.35E-50 | 2.72E-50 |  |
| SLC35B2 | 3.515165 | 5.770435 | 2.25527 | 3.79E-97 | 1.95E-95 |  |
| FTH1P23 | 0.981451 | 3.237107 | 2.255656 | 2.54E-96 | 6.75E-95 |  |
| UBL5P2 | 0.224609 | 2.481093 | 2.256483 | ###### | ###### |  |
| CHIC2 | 2.141874 | 4.400032 | 2.258159 | 7.53E-97 | 2.74E-95 |  |
| RPL4P4 | 1.564984 | 3.82328 | 2.258296 | 3.46E-96 | 8.58E-95 |  |
| DDOST | 4.69716 | 6.956379 | 2.259219 | 1.25E-97 | 1.61E-95 |  |
| DAG1 | 3.336505 | 5.599476 | 2.262971 | 3.10E-96 | 7.92E-95 |  |
| TEAD2 | 1.459322 | 3.722623 | 2.263301 | 8.27E-86 | 3.90E-85 |  |
| SKP1P1 | 0.913544 | 3.179059 | 2.265515 | 1.40E-97 | 1.61E-95 |  |
| AGT | 6.122202 | 8.38784 | 2.265637 | 1.04E-73 | 3.23E-73 |  |
| AURKA | 0.692755 | 2.959051 | 2.266296 | 6.17E-97 | 2.40E-95 |  |
| CDC42P6 | 0.554342 | 2.82283 | 2.268488 | 4.28E-96 | 1.02E-94 |  |
| TGFBR1 | 1.870878 | 4.140175 | 2.269298 | 6.52E-96 | 1.44E-94 |  |
| EFNB1 | 1.717134 | 3.986588 | 2.269453 | 1.70E-96 | 4.95E-95 |  |
| VANGL2 | 2.21164 | 4.4824 | 2.27076 | 1.17E-88 | 6.49E-88 |  |
| CD86 | 0.831781 | 3.107468 | 2.275688 | 3.93E-84 | 1.71E-83 |  |
| SLN | 1.381349 | 3.657441 | 2.276092 | 2.17E-45 | 4.05E-45 |  |
| FAM129A | 0.671131 | 2.947388 | 2.276258 | 1.12E-92 | 8.92E-92 |  |
| FUCA1 | 2.206497 | 4.483975 | 2.277479 | 3.49E-97 | 1.87E-95 |  |
| RNF122 | 1.459096 | 3.739882 | 2.280786 | 1.53E-75 | 4.97E-75 |  |
| HIF1A | 4.013378 | 6.294844 | 2.281466 | 2.30E-95 | 3.93E-94 |  |
| VAMP5 | 4.408135 | 6.690495 | 2.282359 | 2.50E-74 | 7.85E-74 |  |
| RAP2B | 1.510402 | 3.793143 | 2.282741 | 1.19E-96 | 3.76E-95 |  |
| MCM6 | 2.13418 | 4.418563 | 2.284383 | 1.53E-97 | 1.61E-95 |  |
| RCC2 | 3.23514 | 5.522227 | 2.287087 | 1.92E-97 | 1.61E-95 |  |
| TNFRSF1A | 3.539116 | 5.828917 | 2.289801 | 2.93E-86 | 1.41E-85 |  |
| RPL13AP7 | 0.226568 | 2.519391 | 2.292822 | 4.47E-98 | 1.61E-95 |  |
| FTH1P12 | 0.227781 | 2.524087 | 2.296306 | 4.58E-98 | 1.61E-95 |  |
| RPL10P16 | 2.195062 | 4.493069 | 2.298007 | 3.10E-95 | 4.99E-94 |  |
| SERPINA1 | 1.980675 | 4.280476 | 2.299802 | 2.13E-63 | 5.24E-63 |  |
| PTAFR | 0.708044 | 3.009951 | 2.301906 | 3.28E-89 | 1.89E-88 |  |
| C5orf15 | 3.099725 | 5.40326 | 2.303535 | 2.14E-97 | 1.62E-95 |  |
| TWSG1 | 2.326446 | 4.630645 | 2.304199 | 1.25E-96 | 3.92E-95 |  |
| LUM | 1.059198 | 3.364302 | 2.305105 | 2.31E-74 | 7.28E-74 |  |
| PPIC | 1.490301 | 3.796759 | 2.306458 | 1.17E-92 | 9.27E-92 |  |
| PROCR | 1.363666 | 3.670555 | 2.306889 | 4.11E-91 | 2.76E-90 |  |
| MIR3936 | 0.494439 | 2.802364 | 2.307925 | ###### | ###### |  |
| RAB42 | 0.344635 | 2.653089 | 2.308454 | 2.67E-93 | 2.35E-92 |  |
| RPS20P14 | 0.91937 | 3.227833 | 2.308463 | 1.02E-95 | 2.11E-94 |  |
| SLC16A4 | 1.264765 | 3.573241 | 2.308475 | 7.06E-90 | 4.26E-89 |  |
| SYK | 0.79758 | 3.107416 | 2.309836 | 2.67E-90 | 1.67E-89 |  |
| RFC2 | 2.904144 | 5.218124 | 2.313979 | 1.24E-97 | 1.61E-95 |  |
| NAGA | 2.065129 | 4.381804 | 2.316675 | 2.63E-97 | 1.74E-95 |  |
| GDF15 | 0.688531 | 3.006236 | 2.317704 | 1.81E-78 | 6.38E-78 |  |
| STK17A | 2.194317 | 4.512255 | 2.317939 | 1.81E-95 | 3.24E-94 |  |
| EFEMP1 | 4.304248 | 6.623076 | 2.318827 | 3.33E-54 | 7.05E-54 |  |
| POC1A | 0.744557 | 3.066284 | 2.321727 | 3.82E-97 | 1.95E-95 |  |
| CTSO | 2.535637 | 4.8576 | 2.321962 | 3.12E-94 | 3.42E-93 |  |
| TUBB | 6.373427 | 8.695476 | 2.322049 | 3.21E-95 | 5.12E-94 |  |
| EDNRA | 0.830204 | 3.152453 | 2.322249 | 1.11E-94 | 1.41E-93 |  |
| HOXA5 | 0.240366 | 2.566151 | 2.325786 | 1.67E-85 | 7.75E-85 |  |
| DPY19L1 | 2.713214 | 5.040957 | 2.327743 | 4.00E-93 | 3.42E-92 |  |
| LCP1 | 1.790796 | 4.119567 | 2.328771 | 7.73E-83 | 3.18E-82 |  |
| RPS2P46 | 2.126884 | 4.455917 | 2.329033 | 9.51E-89 | 5.32E-88 |  |
| TMEM176B | 3.796696 | 6.129741 | 2.333045 | 3.55E-66 | 9.17E-66 |  |
| POU3F2 | 2.063145 | 4.396857 | 2.333713 | 1.15E-86 | 5.67E-86 |  |
| TSPAN12 | 1.542534 | 3.87686 | 2.334326 | 4.56E-84 | 1.98E-83 |  |
| FBP1 | 0.703947 | 3.039526 | 2.335579 | 4.97E-93 | 4.17E-92 |  |
| LRRC17 | 1.037113 | 3.372829 | 2.335716 | 1.70E-90 | 1.08E-89 |  |
| CCNA2 | 1.129908 | 3.466266 | 2.336358 | 1.90E-90 | 1.20E-89 |  |
| AC026271.1 | 0.384898 | 2.722143 | 2.337245 | 1.22E-97 | 1.61E-95 |  |
| ARL4C | 3.131946 | 5.472879 | 2.340933 | 7.96E-86 | 3.76E-85 |  |
| AL033519.2 | 0.241038 | 2.586572 | 2.345534 | ###### | 3.40E-98 |  |
| TCF19 | 1.135957 | 3.482449 | 2.346492 | 4.30E-92 | 3.21E-91 |  |
| SLC39A1 | 3.646744 | 5.993631 | 2.346887 | 2.02E-97 | 1.61E-95 |  |
| AL035446.1 | 0.264314 | 2.611789 | 2.347475 | 1.38E-90 | 8.86E-90 |  |
| TMX1 | 2.195016 | 4.544541 | 2.349526 | 1.63E-97 | 1.61E-95 |  |
| GPX8 | 0.404599 | 2.755036 | 2.350437 | 2.17E-91 | 1.49E-90 |  |
| AC024293.1 | 3.656477 | 6.008095 | 2.351618 | 1.80E-95 | 3.23E-94 |  |
| ABCA1 | 1.486566 | 3.838837 | 2.352271 | 2.17E-88 | 1.19E-87 |  |
| AC011495.1 | 0.358215 | 2.710628 | 2.352413 | 1.04E-97 | 1.61E-95 |  |
| RPL12P4 | 1.315155 | 3.668376 | 2.353222 | 2.16E-95 | 3.75E-94 |  |
| HLA-H | 1.718317 | 4.074537 | 2.35622 | 6.12E-84 | 2.65E-83 |  |
| S100A9 | 3.820357 | 6.176876 | 2.356519 | 1.36E-46 | 2.59E-46 |  |
| ANO6 | 1.765436 | 4.12238 | 2.356943 | 1.36E-96 | 4.17E-95 |  |
| DLL3 | 1.915032 | 4.272228 | 2.357197 | 1.35E-50 | 2.70E-50 |  |
| AL049873.1 | 1.051171 | 3.409639 | 2.358468 | 1.14E-95 | 2.32E-94 |  |
| PSPH | 2.727623 | 5.090925 | 2.363302 | 3.95E-97 | 1.95E-95 |  |
| TPST1 | 3.192683 | 5.558519 | 2.365837 | 7.20E-91 | 4.72E-90 |  |
| CDC45 | 0.230289 | 2.597372 | 2.367083 | 1.14E-95 | 2.32E-94 |  |
| RPS7P10 | 0.534861 | 2.903116 | 2.368254 | 1.59E-97 | 1.61E-95 |  |
| SERPINH1 | 3.208313 | 5.577317 | 2.369004 | 7.66E-64 | 1.90E-63 |  |
| TSKU | 1.067645 | 3.436811 | 2.369166 | 2.53E-95 | 4.23E-94 |  |
| CAVIN1 | 3.616532 | 5.985775 | 2.369243 | 4.46E-80 | 1.65E-79 |  |
| SDC1 | 0.652835 | 3.023469 | 2.370633 | 3.16E-89 | 1.82E-88 |  |
| ABCC3 | 0.586325 | 2.961562 | 2.375237 | 2.68E-81 | 1.04E-80 |  |
| RPL14P1 | 2.173122 | 4.548785 | 2.375663 | 3.08E-97 | 1.79E-95 |  |
| NCAPH | 0.360172 | 2.735857 | 2.375684 | 4.65E-96 | 1.08E-94 |  |
| KLF10 | 1.750421 | 4.133985 | 2.383564 | 3.83E-94 | 4.06E-93 |  |
| BCHE | 2.327367 | 4.71421 | 2.386843 | 3.41E-77 | 1.16E-76 |  |
| TRIM21 | 1.589632 | 3.978837 | 2.389205 | 7.30E-93 | 5.99E-92 |  |
| PIGT | 4.125211 | 6.515115 | 2.389904 | 2.71E-97 | 1.77E-95 |  |
| GFAP | 10.15321 | 12.5433 | 2.390084 | 1.70E-41 | 3.03E-41 |  |
| GINS1 | 0.770485 | 3.162335 | 2.39185 | 1.51E-96 | 4.51E-95 |  |
| AL355974.3 | 0.700268 | 3.093269 | 2.393001 | 6.37E-79 | 2.28E-78 |  |
| CDK4 | 3.937972 | 6.337559 | 2.399587 | 3.05E-97 | 1.79E-95 |  |
| CDCA5 | 0.763295 | 3.16356 | 2.400265 | 4.99E-94 | 5.12E-93 |  |
| EHD2 | 2.704489 | 5.105245 | 2.400756 | 7.01E-86 | 3.31E-85 |  |
| AL513165.1 | 1.244012 | 3.64602 | 2.402008 | 7.59E-96 | 1.64E-94 |  |
| CDH2 | 3.277958 | 5.681732 | 2.403774 | 3.48E-94 | 3.75E-93 |  |
| LRRC25 | 0.526615 | 2.931574 | 2.404959 | 8.75E-93 | 7.08E-92 |  |
| ACTG1 | 8.623355 | 11.02987 | 2.406518 | 4.99E-97 | 2.11E-95 |  |
| TRAM1 | 3.598994 | 6.006359 | 2.407365 | 3.10E-97 | 1.79E-95 |  |
| C3 | 4.943753 | 7.352324 | 2.40857 | 8.22E-58 | 1.83E-57 |  |
| MT1XP1 | 0.842035 | 3.253065 | 2.411031 | 4.09E-90 | 2.52E-89 |  |
| RPS7P11 | 0.591864 | 3.004 | 2.412137 | 1.62E-97 | 1.61E-95 |  |
| EIF4A1P10 | 0.240389 | 2.653253 | 2.412864 | 1.21E-97 | 1.61E-95 |  |
| SYDE1 | 1.615134 | 4.029137 | 2.414003 | 4.92E-97 | 2.11E-95 |  |
| CSPG4 | 2.058526 | 4.473437 | 2.41491 | 6.66E-86 | 3.15E-85 |  |
| LFNG | 2.956588 | 5.371754 | 2.415166 | 3.26E-84 | 1.43E-83 |  |
| TSPAN6 | 2.40217 | 4.818475 | 2.416305 | 4.07E-93 | 3.47E-92 |  |
| HLA-DMA | 2.9764 | 5.394119 | 2.41772 | 5.60E-78 | 1.95E-77 |  |
| MS4A7 | 1.30493 | 3.722725 | 2.417795 | 2.48E-80 | 9.24E-80 |  |
| AC026403.1 | 2.500965 | 4.919164 | 2.418199 | 4.51E-75 | 1.44E-74 |  |
| H3F3AP6 | 0.210401 | 2.628722 | 2.418321 | ###### | ###### |  |
| HLA-DPA1 | 3.705278 | 6.125352 | 2.420074 | 5.27E-66 | 1.36E-65 |  |
| AC026401.3 | 1.270282 | 3.691137 | 2.420855 | 8.59E-92 | 6.21E-91 |  |
| EEF1A1P5 | 4.348726 | 6.770253 | 2.421527 | 1.77E-91 | 1.23E-90 |  |
| RDH10 | 1.572427 | 3.99473 | 2.422304 | 8.82E-88 | 4.63E-87 |  |
| AC138207.5 | 0.240472 | 2.668964 | 2.428493 | 9.56E-99 | 4.29E-96 |  |
| GBP1 | 1.988135 | 4.420343 | 2.432208 | 1.24E-76 | 4.14E-76 |  |
| EDEM2 | 2.445479 | 4.880782 | 2.435303 | 2.99E-97 | 1.79E-95 |  |
| HLA-E | 5.813076 | 8.251666 | 2.438589 | 1.13E-86 | 5.58E-86 |  |
| WLS | 4.197746 | 6.638203 | 2.440457 | 1.69E-89 | 9.94E-89 |  |
| AQP4 | 5.65048 | 8.09156 | 2.44108 | 5.97E-63 | 1.46E-62 |  |
| HAS2 | 0.245587 | 2.687402 | 2.441815 | 1.31E-96 | 4.06E-95 |  |
| UCP2 | 2.391587 | 4.83366 | 2.442073 | 1.37E-82 | 5.60E-82 |  |
| CMTM6 | 2.212285 | 4.656429 | 2.444144 | 2.36E-97 | 1.65E-95 |  |
| HMGN4 | 3.545393 | 5.989753 | 2.44436 | 1.24E-97 | 1.61E-95 |  |
| SMO | 1.992581 | 4.438138 | 2.445558 | 3.90E-96 | 9.49E-95 |  |
| CHST14 | 1.551203 | 3.997136 | 2.445933 | 1.42E-97 | 1.61E-95 |  |
| RPS7P1 | 2.463237 | 4.909378 | 2.44614 | 5.95E-97 | 2.34E-95 |  |
| GNS | 3.053747 | 5.502001 | 2.448254 | 1.54E-97 | 1.61E-95 |  |
| FHL3 | 2.264991 | 4.718687 | 2.453696 | 1.55E-96 | 4.59E-95 |  |
| HLA-DQA1 | 1.247984 | 3.702235 | 2.454251 | 1.59E-70 | 4.52E-70 |  |
| SURF4 | 4.214464 | 6.668913 | 2.454449 | 1.51E-97 | 1.61E-95 |  |
| FPR3 | 0.232433 | 2.690788 | 2.458355 | 1.92E-94 | 2.25E-93 |  |
| SPC25 | 0.442538 | 2.903179 | 2.460641 | 1.53E-94 | 1.85E-93 |  |
| MTRNR2L12 | 0.911334 | 3.372598 | 2.461264 | 1.07E-93 | 1.01E-92 |  |
| GLIPR2 | 2.908492 | 5.370168 | 2.461676 | 2.98E-91 | 2.02E-90 |  |
| FOXJ1 | 1.258798 | 3.723592 | 2.464794 | 1.49E-62 | 3.60E-62 |  |
| B4GALT5 | 3.049626 | 5.516801 | 2.467175 | 4.04E-97 | 1.96E-95 |  |
| AIF1 | 3.271776 | 5.739515 | 2.46774 | 1.59E-66 | 4.15E-66 |  |
| NRAS | 2.442604 | 4.91163 | 2.469026 | 1.46E-97 | 1.61E-95 |  |
| PMP2 | 5.765205 | 8.234596 | 2.469391 | 1.00E-69 | 2.79E-69 |  |
| ZWINT | 1.119367 | 3.589057 | 2.469691 | 6.58E-95 | 9.17E-94 |  |
| HSD17B10 | 4.501891 | 6.972031 | 2.47014 | 1.22E-97 | 1.61E-95 |  |
| ARC | 2.518736 | 4.99217 | 2.473433 | 7.35E-58 | 1.64E-57 |  |
| FO393411.1 | 0.403584 | 2.878403 | 2.474819 | 1.10E-97 | 1.61E-95 |  |
| SLC1A5 | 1.17288 | 3.649869 | 2.476989 | 2.21E-88 | 1.21E-87 |  |
| PPP1R14BP3 | 1.075793 | 3.553065 | 2.477272 | 3.12E-97 | 1.79E-95 |  |
| CDKN1A | 3.511533 | 5.989128 | 2.477595 | 3.96E-64 | 9.90E-64 |  |
| PHLDA1 | 2.726229 | 5.205399 | 2.479171 | 1.16E-90 | 7.51E-90 |  |
| IL13RA1 | 2.359165 | 4.840566 | 2.481401 | 1.23E-89 | 7.30E-89 |  |
| POFUT1 | 2.052412 | 4.535037 | 2.482625 | 1.23E-97 | 1.61E-95 |  |
| CCN1 | 3.161885 | 5.645373 | 2.483488 | 9.28E-64 | 2.30E-63 |  |
| LRRC55 | 0.743544 | 3.227846 | 2.484302 | 5.03E-86 | 2.39E-85 |  |
| CA3 | 0.965842 | 3.450188 | 2.484347 | 2.92E-74 | 9.16E-74 |  |
| CPVL | 2.255453 | 4.742743 | 2.48729 | 2.30E-78 | 8.09E-78 |  |
| SOX9 | 3.813156 | 6.303417 | 2.490262 | 2.11E-83 | 8.90E-83 |  |
| XRN2 | 2.962068 | 5.453641 | 2.491573 | 1.22E-97 | 1.61E-95 |  |
| DTX3L | 1.304929 | 3.796601 | 2.491672 | 2.76E-93 | 2.42E-92 |  |
| PSMB8 | 3.353315 | 5.847276 | 2.493961 | 5.18E-90 | 3.16E-89 |  |
| LOXL2 | 1.185341 | 3.682748 | 2.497407 | 8.50E-92 | 6.14E-91 |  |
| CD4 | 2.150702 | 4.648335 | 2.497632 | 3.27E-90 | 2.03E-89 |  |
| CCDC71 | 1.842526 | 4.340952 | 2.498425 | 1.24E-97 | 1.61E-95 |  |
| SNRPGP10 | 2.663474 | 5.163952 | 2.500478 | 9.02E-97 | 3.11E-95 |  |
| GRN | 4.308554 | 6.809949 | 2.501395 | 3.38E-96 | 8.43E-95 |  |
| TNFAIP8L2 | 0.934188 | 3.437456 | 2.503269 | 9.72E-91 | 6.31E-90 |  |
| RPL24P4 | 2.280495 | 4.785032 | 2.504537 | 3.25E-96 | 8.18E-95 |  |
| SELPLG | 1.997844 | 4.503375 | 2.505532 | 4.91E-79 | 1.76E-78 |  |
| KIAA0040 | 1.291487 | 3.799088 | 2.507601 | 1.39E-87 | 7.23E-87 |  |
| RPL10P6 | 0.402981 | 2.913852 | 2.510871 | ###### | 1.82E-97 |  |
| PPIAP29 | 1.079412 | 3.590403 | 2.510991 | 8.02E-97 | 2.85E-95 |  |
| SIGLEC10 | 1.187256 | 3.702669 | 2.515413 | 6.25E-83 | 2.59E-82 |  |
| DOLK | 1.97223 | 4.492205 | 2.519975 | 1.38E-97 | 1.61E-95 |  |
| PFN1 | 6.156089 | 8.678645 | 2.522556 | 1.49E-97 | 1.61E-95 |  |
| HLA-DQA2 | 0.360412 | 2.883964 | 2.523551 | 4.46E-75 | 1.43E-74 |  |
| RPL26P19 | 1.428677 | 3.952642 | 2.523965 | 1.30E-96 | 4.04E-95 |  |
| SELENON | 4.274361 | 6.798712 | 2.52435 | 2.71E-95 | 4.47E-94 |  |
| PROS1 | 1.743436 | 4.272278 | 2.528842 | 2.63E-90 | 1.64E-89 |  |
| RP2 | 1.084208 | 3.613942 | 2.529734 | 1.26E-97 | 1.61E-95 |  |
| AC079250.1 | 0.467778 | 2.998254 | 2.530476 | 1.37E-97 | 1.61E-95 |  |
| RPL24P8 | 0.975512 | 3.51072 | 2.535208 | 3.84E-97 | 1.95E-95 |  |
| DNASE2 | 2.918723 | 5.455162 | 2.536438 | 1.43E-97 | 1.61E-95 |  |
| CAV1 | 2.298846 | 4.836046 | 2.537199 | 1.94E-76 | 6.46E-76 |  |
| NRM | 1.63762 | 4.176876 | 2.539256 | 2.34E-97 | 1.65E-95 |  |
| PTGS1 | 0.864682 | 3.40716 | 2.542479 | 6.85E-89 | 3.86E-88 |  |
| HRH1 | 0.983498 | 3.527241 | 2.543743 | 1.97E-89 | 1.15E-88 |  |
| CKAP4 | 2.633268 | 5.179991 | 2.546722 | 1.69E-97 | 1.61E-95 |  |
| SLC40A1 | 2.023961 | 4.571953 | 2.547992 | 7.72E-92 | 5.61E-91 |  |
| KPNA2 | 3.302619 | 5.851002 | 2.548383 | 5.34E-97 | 2.19E-95 |  |
| MEX3A | 0.501464 | 3.051692 | 2.550228 | 7.37E-96 | 1.60E-94 |  |
| SOX11 | 0.567809 | 3.118619 | 2.55081 | 1.15E-93 | 1.09E-92 |  |
| AC246787.1 | 0.426509 | 2.979037 | 2.552528 | 1.28E-97 | 1.61E-95 |  |
| AC115618.2 | 1.549136 | 4.110758 | 2.561622 | 3.64E-97 | 1.91E-95 |  |
| CCDC167 | 3.566265 | 6.127893 | 2.561629 | 1.00E-96 | 3.34E-95 |  |
| RNASEH2A | 2.291465 | 4.855561 | 2.564096 | 1.51E-97 | 1.61E-95 |  |
| HAVCR2 | 1.555697 | 4.123228 | 2.567532 | 1.19E-86 | 5.90E-86 |  |
| AC114491.1 | 1.628248 | 4.197188 | 2.56894 | 7.44E-95 | 1.02E-93 |  |
| CD99 | 5.607112 | 8.178265 | 2.571153 | 2.09E-84 | 9.26E-84 |  |
| PTPRZ1 | 5.261796 | 7.839325 | 2.577529 | 2.17E-77 | 7.38E-77 |  |
| GNG5 | 4.471362 | 7.050761 | 2.579399 | 5.72E-96 | 1.29E-94 |  |
| EZH2 | 0.924426 | 3.511301 | 2.586876 | 9.35E-94 | 9.01E-93 |  |
| RPS2P5 | 4.994686 | 7.589164 | 2.594478 | 3.99E-82 | 1.59E-81 |  |
| ANGPTL2 | 2.510019 | 5.105205 | 2.595187 | 1.72E-92 | 1.34E-91 |  |
| CENPU | 0.736671 | 3.33382 | 2.597149 | 2.40E-94 | 2.72E-93 |  |
| ABI3 | 1.267052 | 3.868811 | 2.601759 | 7.64E-90 | 4.60E-89 |  |
| C5AR1 | 1.13027 | 3.737504 | 2.607234 | 1.60E-81 | 6.24E-81 |  |
| AC011462.1 | 2.541666 | 5.153632 | 2.611965 | 9.17E-94 | 8.86E-93 |  |
| S100A16 | 5.02217 | 7.634952 | 2.612782 | 1.17E-80 | 4.40E-80 |  |
| AL122020.1 | 0.273163 | 2.887489 | 2.614326 | 5.17E-98 | 1.61E-95 |  |
| ITGB2 | 2.286377 | 4.902847 | 2.61647 | 5.11E-79 | 1.83E-78 |  |
| RPL7AP6 | 1.763524 | 4.381418 | 2.617894 | 3.75E-97 | 1.95E-95 |  |
| CDCA7 | 0.502139 | 3.12057 | 2.618431 | 4.30E-97 | 2.04E-95 |  |
| VCAM1 | 1.084137 | 3.707954 | 2.623817 | 1.03E-81 | 4.04E-81 |  |
| CNGA3 | 0.343421 | 2.967508 | 2.624087 | 5.88E-86 | 2.79E-85 |  |
| ID4 | 4.227836 | 6.858414 | 2.630577 | 2.27E-81 | 8.78E-81 |  |
| BCAN | 5.780743 | 8.412182 | 2.631438 | 8.06E-59 | 1.83E-58 |  |
| AC115837.1 | 0.815966 | 3.448951 | 2.632985 | 1.22E-97 | 1.61E-95 |  |
| AL136454.1 | 1.095251 | 3.728862 | 2.633611 | 4.81E-97 | 2.11E-95 |  |
| RPL35P1 | 0.295826 | 2.929572 | 2.633746 | 4.03E-99 | 1.85E-96 |  |
| UHRF1 | 0.679032 | 3.313847 | 2.634815 | 3.87E-94 | 4.09E-93 |  |
| PTTG1 | 1.909649 | 4.550099 | 2.640451 | 7.87E-90 | 4.73E-89 |  |
| S100A6 | 6.952905 | 9.594791 | 2.641885 | 2.29E-90 | 1.44E-89 |  |
| SPI1 | 2.248619 | 4.890717 | 2.642099 | 5.12E-85 | 2.32E-84 |  |
| SPARC | 7.609917 | 10.25245 | 2.642529 | 2.88E-84 | 1.26E-83 |  |
| GPX1 | 6.026619 | 8.669728 | 2.643109 | 3.44E-97 | 1.85E-95 |  |
| HNRNPCP2 | 0.855907 | 3.500753 | 2.644846 | 8.98E-97 | 3.10E-95 |  |
| RPS3AP5 | 0.606456 | 3.251552 | 2.645096 | 2.30E-97 | 1.64E-95 |  |
| PTTG1IP | 5.185895 | 7.834212 | 2.648318 | 1.74E-97 | 1.61E-95 |  |
| RPL10P9 | 1.768142 | 4.417181 | 2.649039 | 2.55E-55 | 5.48E-55 |  |
| AL450998.1 | 0.258995 | 2.910301 | 2.651307 | 2.37E-99 | 1.15E-96 |  |
| MAGT1 | 2.496531 | 5.150936 | 2.654405 | 9.44E-97 | 3.18E-95 |  |
| CCR1 | 0.733045 | 3.391474 | 2.658428 | 1.66E-90 | 1.06E-89 |  |
| KDELR2 | 3.822095 | 6.482812 | 2.660717 | 1.24E-97 | 1.61E-95 |  |
| AC107983.1 | 0.244998 | 2.905884 | 2.660886 | ###### | ###### |  |
| LAPTM4A | 5.284131 | 7.9459 | 2.661769 | 1.40E-97 | 1.61E-95 |  |
| HIST1H2BK | 2.4372 | 5.109266 | 2.672066 | 1.48E-89 | 8.73E-89 |  |
| LOX | 0.663486 | 3.344977 | 2.681491 | 9.29E-94 | 8.96E-93 |  |
| MS4A4A | 1.123078 | 3.805423 | 2.682345 | 1.00E-83 | 4.28E-83 |  |
| SEC61A1 | 4.497969 | 7.181919 | 2.683949 | 1.66E-97 | 1.61E-95 |  |
| TMEM119 | 1.265486 | 3.952218 | 2.686732 | 1.06E-84 | 4.73E-84 |  |
| SFRP4 | 1.000375 | 3.687806 | 2.68743 | 6.47E-88 | 3.43E-87 |  |
| AL139095.2 | 0.203247 | 2.891453 | 2.688207 | 9.81E-98 | 1.61E-95 |  |
| FTH1P20 | 1.537248 | 4.227205 | 2.689957 | 3.76E-95 | 5.78E-94 |  |
| RPL35P5 | 0.891379 | 3.583264 | 2.691884 | 3.42E-97 | 1.85E-95 |  |
| NAPSB | 1.341115 | 4.03585 | 2.694735 | 6.29E-72 | 1.85E-71 |  |
| KIF4A | 0.224509 | 2.921038 | 2.696529 | 2.06E-97 | 1.61E-95 |  |
| HLA-B | 6.432547 | 9.135564 | 2.703017 | 4.50E-89 | 2.57E-88 |  |
| MSR1 | 1.008644 | 3.712701 | 2.704056 | 6.86E-86 | 3.25E-85 |  |
| AL590867.2 | 2.851787 | 5.55761 | 2.705822 | 1.74E-96 | 5.04E-95 |  |
| SPC24 | 0.338282 | 3.048676 | 2.710393 | 1.82E-95 | 3.27E-94 |  |
| PMS2P1 | 1.767801 | 4.480885 | 2.713084 | 1.31E-97 | 1.61E-95 |  |
| MAPRE1 | 4.102642 | 6.817822 | 2.715181 | 1.24E-97 | 1.61E-95 |  |
| YBX1 | 6.285862 | 9.005124 | 2.719262 | 1.34E-97 | 1.61E-95 |  |
| CCND2 | 2.881246 | 5.605535 | 2.724289 | 3.81E-88 | 2.05E-87 |  |
| HLA-DPB1 | 3.921893 | 6.648277 | 2.726384 | 4.78E-84 | 2.07E-83 |  |
| APOL4 | 0.786659 | 3.514692 | 2.728033 | 1.94E-77 | 6.62E-77 |  |
| PLIN3 | 2.981203 | 5.709805 | 2.728601 | 1.79E-91 | 1.24E-90 |  |
| LAMC1 | 2.051724 | 4.782132 | 2.730408 | 3.07E-96 | 7.87E-95 |  |
| S100A10 | 4.641285 | 7.374215 | 2.73293 | 7.26E-69 | 1.99E-68 |  |
| FJX1 | 2.599995 | 5.338015 | 2.738021 | 7.29E-96 | 1.59E-94 |  |
| RPL10AP6 | 0.827126 | 3.568988 | 2.741862 | 1.75E-97 | 1.61E-95 |  |
| CCNB1 | 1.935881 | 4.678194 | 2.742314 | 4.88E-94 | 5.03E-93 |  |
| FSTL1 | 2.709987 | 5.454185 | 2.744198 | 3.13E-95 | 5.00E-94 |  |
| CFI | 1.344727 | 4.089991 | 2.745264 | 2.31E-85 | 1.07E-84 |  |
| BST2 | 4.059649 | 6.807617 | 2.747968 | 8.62E-77 | 2.89E-76 |  |
| EMP1 | 2.80912 | 5.559192 | 2.750072 | 7.21E-74 | 2.24E-73 |  |
| RPS3AP26 | 1.556413 | 4.315889 | 2.759476 | 1.13E-96 | 3.61E-95 |  |
| LY96 | 1.70731 | 4.466836 | 2.759527 | 2.37E-84 | 1.05E-83 |  |
| MTCO2P2 | 1.072051 | 3.833451 | 2.7614 | 1.11E-91 | 7.91E-91 |  |
| FLNC | 1.392663 | 4.156172 | 2.763509 | 1.90E-80 | 7.10E-80 |  |
| AC093673.1 | 2.471914 | 5.235664 | 2.76375 | 7.56E-96 | 1.63E-94 |  |
| RPL37P2 | 0.412615 | 3.181097 | 2.768482 | ###### | 7.75E-98 |  |
| COL6A2 | 2.764518 | 5.536027 | 2.771509 | 1.45E-73 | 4.45E-73 |  |
| HLA-DOA | 0.989766 | 3.761995 | 2.772229 | 3.07E-90 | 1.91E-89 |  |
| FAUP1 | 0.694439 | 3.467856 | 2.773417 | 3.94E-97 | 1.95E-95 |  |
| SNRPB | 4.582602 | 7.356428 | 2.773826 | 1.22E-97 | 1.61E-95 |  |
| TPT1P4 | 0.266966 | 3.043507 | 2.776542 | 5.01E-98 | 1.61E-95 |  |
| S100A4 | 2.404934 | 5.18241 | 2.777476 | 1.08E-80 | 4.06E-80 |  |
| CXCL16 | 2.775833 | 5.553678 | 2.777845 | 3.80E-90 | 2.34E-89 |  |
| PLEKHA4 | 1.933238 | 4.712632 | 2.779395 | 2.18E-86 | 1.06E-85 |  |
| TMEM255A | 1.93405 | 4.713451 | 2.779401 | 3.59E-81 | 1.38E-80 |  |
| LGALS3BP | 4.602365 | 7.382976 | 2.780611 | 1.04E-86 | 5.17E-86 |  |
| HSPA5 | 5.18187 | 7.963651 | 2.781781 | 3.31E-94 | 3.60E-93 |  |
| AL355472.1 | 0.376915 | 3.159894 | 2.782979 | 7.64E-98 | 1.61E-95 |  |
| MSN | 4.333936 | 7.117697 | 2.783761 | 2.00E-90 | 1.26E-89 |  |
| EMILIN1 | 1.94606 | 4.731704 | 2.785643 | 3.63E-93 | 3.11E-92 |  |
| APLNR | 2.928522 | 5.717597 | 2.789075 | 5.49E-61 | 1.29E-60 |  |
| TYMS | 1.983407 | 4.777574 | 2.794167 | 2.64E-91 | 1.80E-90 |  |
| LINC02587 | 0.153589 | 2.948199 | 2.79461 | 3.00E-85 | 1.37E-84 |  |
| LAPTM4B | 3.562498 | 6.359192 | 2.796695 | 1.60E-97 | 1.61E-95 |  |
| MTND6P4 | 0.290372 | 3.087964 | 2.797593 | 1.07E-97 | 1.61E-95 |  |
| CHCHD2P6 | 0.735688 | 3.533818 | 2.79813 | 1.36E-97 | 1.61E-95 |  |
| YBX1P1 | 0.675393 | 3.476029 | 2.800636 | 1.31E-97 | 1.61E-95 |  |
| RPL5P34 | 0.472591 | 3.274012 | 2.80142 | 1.22E-97 | 1.61E-95 |  |
| FTH1P16 | 0.528972 | 3.331202 | 2.80223 | 1.47E-97 | 1.61E-95 |  |
| AL080243.2 | 0.632603 | 3.440831 | 2.808227 | 1.77E-97 | 1.61E-95 |  |
| RPS29P5 | 0.301354 | 3.11198 | 2.810627 | ###### | ###### |  |
| MDFI | 1.600643 | 4.413279 | 2.812636 | 1.07E-83 | 4.57E-83 |  |
| TNFAIP6 | 0.780092 | 3.595188 | 2.815095 | 9.14E-89 | 5.11E-88 |  |
| CTSS | 1.843248 | 4.658799 | 2.815551 | 4.90E-84 | 2.13E-83 |  |
| AL450405.1 | 2.628519 | 5.44621 | 2.817691 | 1.31E-86 | 6.44E-86 |  |
| MFAP4 | 1.899553 | 4.718297 | 2.818744 | 3.32E-89 | 1.91E-88 |  |
| EMP3 | 3.541444 | 6.364707 | 2.823263 | 1.32E-80 | 4.95E-80 |  |
| TK1 | 1.150293 | 3.978683 | 2.828389 | 5.50E-94 | 5.56E-93 |  |
| NXT1 | 2.298663 | 5.12712 | 2.828457 | 1.40E-97 | 1.61E-95 |  |
| C1QL1 | 3.632345 | 6.463811 | 2.831466 | 3.03E-69 | 8.39E-69 |  |
| CSF1R | 2.724445 | 5.557033 | 2.832588 | 1.51E-81 | 5.88E-81 |  |
| SASH3 | 1.052735 | 3.888298 | 2.835563 | 1.00E-91 | 7.20E-91 |  |
| RAB20 | 1.377003 | 4.213282 | 2.836279 | 5.47E-93 | 4.58E-92 |  |
| IFI6 | 5.8258 | 8.667523 | 2.841723 | 3.78E-83 | 1.58E-82 |  |
| CALR | 6.288096 | 9.13001 | 2.841914 | 1.34E-97 | 1.61E-95 |  |
| PRDX4 | 3.639579 | 6.481683 | 2.842104 | 2.19E-97 | 1.62E-95 |  |
| FKBP10 | 3.266915 | 6.111767 | 2.844852 | 4.86E-97 | 2.11E-95 |  |
| MCUB | 1.140912 | 3.995406 | 2.854493 | 8.93E-94 | 8.66E-93 |  |
| CCL2 | 2.337102 | 5.192759 | 2.855657 | 5.80E-57 | 1.28E-56 |  |
| MDK | 3.788043 | 6.644668 | 2.856626 | 1.85E-87 | 9.58E-87 |  |
| PLA2G2A | 0.632283 | 3.49564 | 2.863356 | 1.68E-73 | 5.16E-73 |  |
| DPYSL3 | 4.390836 | 7.257933 | 2.867096 | 2.72E-92 | 2.07E-91 |  |
| LMNB1 | 1.467446 | 4.335946 | 2.868499 | 1.18E-92 | 9.36E-92 |  |
| IL13RA2 | 0.92261 | 3.79378 | 2.87117 | 1.46E-66 | 3.82E-66 |  |
| ARHGDIB | 3.675653 | 6.549603 | 2.87395 | 5.17E-92 | 3.83E-91 |  |
| F13A1 | 0.987685 | 3.861649 | 2.873964 | 2.74E-73 | 8.39E-73 |  |
| RPL15P3 | 1.832994 | 4.706959 | 2.873966 | 1.98E-97 | 1.61E-95 |  |
| NTN1 | 1.502358 | 4.378973 | 2.876615 | 5.62E-93 | 4.69E-92 |  |
| OLFML2B | 0.911516 | 3.790076 | 2.878561 | 5.93E-94 | 5.96E-93 |  |
| ACKR3 | 2.19778 | 5.088019 | 2.890239 | 8.54E-94 | 8.31E-93 |  |
| AC131235.1 | 0.415672 | 3.30595 | 2.890278 | 1.22E-97 | 1.61E-95 |  |
| CD53 | 2.739173 | 5.629881 | 2.890708 | 1.98E-84 | 8.76E-84 |  |
| AL133415.1 | 0.184752 | 3.076427 | 2.891675 | ###### | 2.29E-97 |  |
| SMIM3 | 2.451683 | 5.343731 | 2.892048 | 1.70E-92 | 1.32E-91 |  |
| CDCA8 | 0.503895 | 3.397284 | 2.893388 | 1.30E-97 | 1.61E-95 |  |
| KIF2C | 0.388816 | 3.283772 | 2.894956 | 1.71E-97 | 1.61E-95 |  |
| SPOCD1 | 0.725449 | 3.628586 | 2.903137 | 4.89E-79 | 1.75E-78 |  |
| CXCL8 | 1.031765 | 3.935478 | 2.903713 | 3.80E-63 | 9.32E-63 |  |
| GPX7 | 1.554295 | 4.458357 | 2.904062 | 1.22E-97 | 1.61E-95 |  |
| TMSB15A | 0.57112 | 3.477031 | 2.905912 | 3.98E-72 | 1.18E-71 |  |
| ODC1 | 3.766435 | 6.675303 | 2.908868 | 2.00E-96 | 5.64E-95 |  |
| FOLR2 | 1.46545 | 4.382636 | 2.917186 | 1.73E-83 | 7.33E-83 |  |
| CD93 | 0.966433 | 3.88414 | 2.917707 | 3.88E-89 | 2.22E-88 |  |
| GAS1 | 1.149454 | 4.067857 | 2.918403 | 2.14E-96 | 5.93E-95 |  |
| MARCKS | 4.854022 | 7.775231 | 2.92121 | 1.42E-97 | 1.61E-95 |  |
| AC004967.1 | 0.271481 | 3.192755 | 2.921274 | 1.41E-98 | 6.18E-96 |  |
| HSPB1P1 | 0.267693 | 3.192105 | 2.924411 | ###### | ###### |  |
| GNG12 | 2.455268 | 5.384854 | 2.929586 | 2.76E-96 | 7.27E-95 |  |
| HK2 | 0.941382 | 3.876321 | 2.93494 | 1.04E-92 | 8.38E-92 |  |
| RPS13P2 | 0.483623 | 3.419086 | 2.935463 | 1.17E-97 | 1.61E-95 |  |
| TPI1P1 | 1.274925 | 4.21518 | 2.940255 | 1.74E-97 | 1.61E-95 |  |
| SOCS3 | 2.330403 | 5.27126 | 2.940857 | 1.35E-62 | 3.27E-62 |  |
| C1R | 2.931215 | 5.875935 | 2.94472 | 2.70E-83 | 1.13E-82 |  |
| EEF1A1P6 | 1.807478 | 4.756059 | 2.948581 | 3.93E-97 | 1.95E-95 |  |
| CDK1 | 0.437281 | 3.386801 | 2.94952 | 3.82E-97 | 1.95E-95 |  |
| CX3CR1 | 1.491541 | 4.442758 | 2.951218 | 1.60E-78 | 5.65E-78 |  |
| IGFBP4 | 3.299889 | 6.251507 | 2.951618 | 2.30E-91 | 1.58E-90 |  |
| ANXA5 | 5.486062 | 8.438166 | 2.952104 | 9.63E-96 | 2.02E-94 |  |
| AP000763.2 | 0.563787 | 3.522546 | 2.958759 | ###### | ###### |  |
| RPS15AP1 | 0.509909 | 3.470745 | 2.960836 | 1.41E-97 | 1.61E-95 |  |
| FTH1P8 | 1.516892 | 4.485549 | 2.968656 | 3.06E-96 | 7.85E-95 |  |
| YBX1P10 | 0.38238 | 3.362105 | 2.979725 | 1.22E-97 | 1.61E-95 |  |
| HLA-C | 5.841006 | 8.829412 | 2.988405 | 7.09E-91 | 4.65E-90 |  |
| SPINK8 | 0.331969 | 3.324468 | 2.992499 | 1.01E-94 | 1.31E-93 |  |
| EEF1A1P19 | 0.251037 | 3.25688 | 3.005843 | 1.22E-97 | 1.61E-95 |  |
| IBSP | 0.122199 | 3.130232 | 3.008033 | 2.04E-94 | 2.37E-93 |  |
| RPL13P12 | 3.083934 | 6.092917 | 3.008983 | 2.86E-72 | 8.51E-72 |  |
| TCIM | 1.825532 | 4.835135 | 3.009603 | 2.08E-87 | 1.07E-86 |  |
| AURKB | 0.157353 | 3.169323 | 3.01197 | 4.58E-98 | 1.61E-95 |  |
| SRGN | 4.269222 | 7.28174 | 3.012518 | 9.72E-80 | 3.57E-79 |  |
| CMTM3 | 2.263242 | 5.275892 | 3.01265 | 8.74E-97 | 3.03E-95 |  |
| AL671277.1 | 0.582184 | 3.595868 | 3.013684 | 1.47E-96 | 4.40E-95 |  |
| CXCR4 | 2.368328 | 5.383221 | 3.014893 | 5.40E-82 | 2.14E-81 |  |
| PLTP | 4.879101 | 7.898117 | 3.019016 | 3.21E-91 | 2.17E-90 |  |
| TMSB4XP4 | 1.38108 | 4.400201 | 3.019121 | 4.42E-97 | 2.07E-95 |  |
| CDKN2C | 2.196855 | 5.216341 | 3.019486 | 5.63E-86 | 2.67E-85 |  |
| LINC02381 | 0.820807 | 3.846144 | 3.025336 | 3.84E-87 | 1.96E-86 |  |
| AEBP1 | 3.820319 | 6.848726 | 3.028407 | 3.65E-73 | 1.11E-72 |  |
| HLA-A | 6.217887 | 9.249289 | 3.031403 | 4.52E-93 | 3.83E-92 |  |
| MCM2 | 1.415687 | 4.452325 | 3.036638 | 2.29E-97 | 1.64E-95 |  |
| AC099850.3 | 0.159476 | 3.205724 | 3.046249 | ###### | ###### |  |
| RNASE2 | 0.783194 | 3.832912 | 3.049718 | 4.18E-85 | 1.91E-84 |  |
| GAL3ST4 | 1.720818 | 4.771488 | 3.050671 | 1.60E-97 | 1.61E-95 |  |
| PI3 | 0.2964 | 3.355506 | 3.059106 | 8.86E-87 | 4.41E-86 |  |
| CD248 | 1.256359 | 4.317842 | 3.061482 | 3.07E-91 | 2.08E-90 |  |
| PTMAP2 | 1.092133 | 4.154623 | 3.06249 | 1.91E-97 | 1.61E-95 |  |
| KLHDC8A | 2.476858 | 5.546447 | 3.069588 | 8.49E-83 | 3.49E-82 |  |
| CNN3 | 5.83036 | 8.902964 | 3.072604 | 2.98E-97 | 1.79E-95 |  |
| VAMP8 | 2.549611 | 5.622698 | 3.073087 | 5.25E-90 | 3.20E-89 |  |
| APLN | 2.663601 | 5.740763 | 3.077162 | 1.77E-90 | 1.12E-89 |  |
| AC080038.1 | 2.116763 | 5.196586 | 3.079823 | 5.11E-91 | 3.39E-90 |  |
| COL1A2 | 2.119055 | 5.201184 | 3.082129 | 1.27E-87 | 6.59E-87 |  |
| MTX1P1 | 0.129885 | 3.220266 | 3.090381 | ###### | ###### |  |
| LY86 | 1.513897 | 4.609601 | 3.095703 | 2.56E-88 | 1.39E-87 |  |
| A2M | 4.831245 | 7.931333 | 3.100088 | 6.54E-94 | 6.49E-93 |  |
| TP53 | 1.817091 | 4.942276 | 3.125185 | 1.11E-96 | 3.57E-95 |  |
| TGFBI | 1.94505 | 5.074417 | 3.129367 | 4.49E-85 | 2.04E-84 |  |
| COL5A2 | 1.270062 | 4.402326 | 3.132264 | 4.19E-90 | 2.57E-89 |  |
| FOXM1 | 0.626649 | 3.759099 | 3.132451 | 2.50E-97 | 1.71E-95 |  |
| MTATP8P1 | 0.679625 | 3.813531 | 3.133906 | ###### | 1.74E-97 |  |
| KIFC1 | 0.222195 | 3.360836 | 3.138642 | 2.00E-97 | 1.61E-95 |  |
| CALU | 3.356252 | 6.495793 | 3.139541 | 1.34E-97 | 1.61E-95 |  |
| ANXA1 | 3.31219 | 6.452473 | 3.140282 | 1.67E-75 | 5.40E-75 |  |
| TPT1P9 | 0.603845 | 3.751543 | 3.147698 | 2.03E-97 | 1.61E-95 |  |
| MYC | 1.230677 | 4.394367 | 3.163691 | 2.99E-96 | 7.71E-95 |  |
| SOX2 | 4.426188 | 7.592324 | 3.166136 | 7.34E-90 | 4.43E-89 |  |
| RPN2 | 4.430055 | 7.604426 | 3.174371 | 1.22E-97 | 1.61E-95 |  |
| RAB32 | 1.687559 | 4.866846 | 3.179287 | 1.57E-95 | 2.90E-94 |  |
| LYZ | 1.399599 | 4.581324 | 3.181725 | 7.16E-86 | 3.38E-85 |  |
| SAA1 | 0.766112 | 3.94864 | 3.182528 | 5.67E-66 | 1.46E-65 |  |
| C21orf62 | 0.350211 | 3.5389 | 3.188689 | 1.60E-92 | 1.25E-91 |  |
| TNFRSF19 | 0.755858 | 3.948699 | 3.192841 | 1.41E-97 | 1.61E-95 |  |
| TUBAP2 | 0.229125 | 3.425499 | 3.196375 | 1.21E-97 | 1.61E-95 |  |
| NNMT | 1.690492 | 4.89439 | 3.203898 | 1.59E-75 | 5.17E-75 |  |
| FCER1G | 3.451728 | 6.6769 | 3.225172 | 2.60E-81 | 1.00E-80 |  |
| CXCL10 | 0.657095 | 3.888205 | 3.23111 | 3.62E-81 | 1.39E-80 |  |
| EEF1A1P12 | 0.105182 | 3.354959 | 3.249777 | 8.22E-98 | 1.61E-95 |  |
| CCNB2 | 0.244357 | 3.502327 | 3.25797 | 1.70E-97 | 1.61E-95 |  |
| ZFP36L2 | 3.261247 | 6.519443 | 3.258195 | 4.42E-97 | 2.07E-95 |  |
| FPR1 | 1.290275 | 4.553156 | 3.262881 | 1.83E-84 | 8.12E-84 |  |
| CD163 | 1.635463 | 4.89875 | 3.263287 | 1.69E-76 | 5.64E-76 |  |
| TMSB4XP2 | 0.537402 | 3.805282 | 3.26788 | ###### | ###### |  |
| PTN | 6.167462 | 9.436937 | 3.269474 | 2.55E-93 | 2.25E-92 |  |
| PCNA | 3.818685 | 7.093539 | 3.274854 | 2.08E-97 | 1.61E-95 |  |
| CHCHD2P9 | 0.130914 | 3.406186 | 3.275272 | ###### | ###### |  |
| VIM | 6.540797 | 9.834792 | 3.293996 | 1.89E-93 | 1.71E-92 |  |
| ACTB | 9.596192 | 12.89445 | 3.298259 | 4.07E-96 | 9.79E-95 |  |
| CYBB | 1.314028 | 4.615776 | 3.301748 | 1.27E-92 | 1.01E-91 |  |
| HLA-DRB6 | 0.488612 | 3.79216 | 3.303548 | 3.02E-87 | 1.54E-86 |  |
| CYTL1 | 1.019996 | 4.332096 | 3.3121 | 3.08E-94 | 3.38E-93 |  |
| AP001324.1 | 1.572013 | 4.889578 | 3.317565 | 2.85E-97 | 1.79E-95 |  |
| MEOX2 | 0.256514 | 3.574408 | 3.317893 | 2.80E-90 | 1.74E-89 |  |
| APOBEC3C | 0.923376 | 4.249423 | 3.326047 | 3.87E-97 | 1.95E-95 |  |
| C3AR1 | 1.271989 | 4.602863 | 3.330874 | 4.88E-92 | 3.62E-91 |  |
| GAPDHP65 | 0.181499 | 3.513563 | 3.332064 | 1.09E-97 | 1.61E-95 |  |
| EEF1A1P9 | 0.101241 | 3.439349 | 3.338108 | 6.36E-98 | 1.61E-95 |  |
| MAFB | 1.455987 | 4.795038 | 3.339052 | 3.28E-95 | 5.20E-94 |  |
| AC073861.1 | 2.660854 | 6.003022 | 3.342167 | 1.67E-97 | 1.61E-95 |  |
| ANXA2P2 | 0.338753 | 3.681367 | 3.342614 | 3.92E-97 | 1.95E-95 |  |
| TAGLN2 | 4.793108 | 8.145486 | 3.352378 | 3.06E-89 | 1.77E-88 |  |
| AC004453.1 | 1.548733 | 4.908074 | 3.359341 | 3.62E-97 | 1.91E-95 |  |
| RPL37AP1 | 0.1514 | 3.517368 | 3.365968 | ###### | ###### |  |
| GPX1P1 | 1.056321 | 4.424031 | 3.367711 | 1.83E-91 | 1.27E-90 |  |
| EEF1A1P11 | 0.31252 | 3.680386 | 3.367866 | 7.89E-97 | 2.83E-95 |  |
| NID1 | 1.458527 | 4.829687 | 3.37116 | 5.22E-97 | 2.16E-95 |  |
| AC007969.1 | 1.880931 | 5.253467 | 3.372536 | 1.23E-97 | 1.61E-95 |  |
| RNASE6 | 1.398276 | 4.795241 | 3.396965 | 1.10E-91 | 7.83E-91 |  |
| CCN2 | 2.624632 | 6.022101 | 3.397469 | 7.97E-92 | 5.78E-91 |  |
| RPS11P5 | 0.565324 | 3.974015 | 3.40869 | 1.20E-97 | 1.61E-95 |  |
| AQP1 | 4.829589 | 8.244402 | 3.414814 | 3.18E-69 | 8.79E-69 |  |
| MEST | 2.424703 | 5.843325 | 3.418621 | 2.33E-90 | 1.46E-89 |  |
| FIBIN | 2.52056 | 5.948812 | 3.428252 | 1.47E-97 | 1.61E-95 |  |
| EGR1 | 3.562308 | 6.991137 | 3.428829 | 3.17E-79 | 1.14E-78 |  |
| PIMREG | 0.253699 | 3.683811 | 3.430112 | 1.77E-96 | 5.09E-95 |  |
| MTND4P12 | 0.862076 | 4.310282 | 3.448206 | 9.70E-92 | 6.96E-91 |  |
| PLP2 | 2.897053 | 6.345691 | 3.448638 | 8.92E-93 | 7.21E-92 |  |
| PLAU | 0.745798 | 4.196043 | 3.450245 | 1.18E-95 | 2.38E-94 |  |
| TMSB10 | 9.08914 | 12.5407 | 3.451556 | 2.24E-92 | 1.72E-91 |  |
| ALOX5AP | 2.284403 | 5.747888 | 3.463485 | 1.91E-84 | 8.46E-84 |  |
| SLPI | 1.122159 | 4.586771 | 3.464611 | 6.22E-84 | 2.69E-83 |  |
| STEAP3 | 1.159665 | 4.624352 | 3.464687 | 1.75E-94 | 2.09E-93 |  |
| SOX4 | 1.449917 | 4.922233 | 3.472316 | 3.03E-97 | 1.79E-95 |  |
| EIF4EBP1 | 2.255358 | 5.728027 | 3.47267 | 6.37E-97 | 2.45E-95 |  |
| AC016739.1 | 2.178229 | 5.652533 | 3.474304 | 2.75E-96 | 7.24E-95 |  |
| MT2P1 | 1.00923 | 4.487792 | 3.478562 | 3.71E-97 | 1.93E-95 |  |
| CLIC4 | 4.062448 | 7.544452 | 3.482004 | 2.93E-96 | 7.62E-95 |  |
| IGFBP7 | 5.676052 | 9.163304 | 3.487252 | 1.92E-94 | 2.25E-93 |  |
| TYROBP | 3.845362 | 7.335736 | 3.490374 | 1.50E-87 | 7.76E-87 |  |
| AC005912.1 | 2.158479 | 5.651346 | 3.492867 | 1.05E-96 | 3.43E-95 |  |
| SRPX2 | 0.41055 | 3.906276 | 3.495726 | 1.81E-96 | 5.19E-95 |  |
| ASF1B | 0.202817 | 3.732975 | 3.530157 | 1.51E-97 | 1.61E-95 |  |
| PTX3 | 0.60451 | 4.151316 | 3.546807 | 6.48E-94 | 6.44E-93 |  |
| CD74 | 6.27545 | 9.825412 | 3.549962 | 1.57E-87 | 8.13E-87 |  |
| EEF1A1P13 | 0.569221 | 4.122491 | 3.55327 | 1.22E-97 | 1.61E-95 |  |
| PDIA4 | 3.176817 | 6.738468 | 3.561651 | 4.34E-97 | 2.05E-95 |  |
| ID3 | 4.314306 | 7.880534 | 3.566228 | 1.14E-92 | 9.12E-92 |  |
| TIMP4 | 2.40691 | 5.979644 | 3.572734 | 1.33E-86 | 6.54E-86 |  |
| GPR34 | 1.289751 | 4.86639 | 3.576639 | 2.02E-93 | 1.81E-92 |  |
| MMP9 | 0.352004 | 3.933258 | 3.581254 | 1.30E-91 | 9.16E-91 |  |
| AL161787.1 | 0.417407 | 4.000374 | 3.582968 | ###### | ###### |  |
| CLIC1 | 3.924797 | 7.525714 | 3.600917 | 3.36E-93 | 2.90E-92 |  |
| POSTN | 0.4726 | 4.079925 | 3.607325 | 3.34E-85 | 1.53E-84 |  |
| BGN | 3.920465 | 7.532077 | 3.611612 | 2.12E-95 | 3.70E-94 |  |
| CKS2 | 2.360775 | 6.0133 | 3.652525 | 6.49E-96 | 1.44E-94 |  |
| AC010343.1 | 0.861369 | 4.564276 | 3.702907 | 1.22E-97 | 1.61E-95 |  |
| IGFBP3 | 2.309504 | 6.037491 | 3.727987 | 3.69E-82 | 1.48E-81 |  |
| RRM2 | 0.164399 | 3.89835 | 3.733952 | 2.19E-97 | 1.62E-95 |  |
| OLFML3 | 1.684853 | 5.423499 | 3.738645 | 4.62E-97 | 2.11E-95 |  |
| FMOD | 0.783892 | 4.543774 | 3.759883 | 8.82E-93 | 7.14E-92 |  |
| FTLP3 | 2.385427 | 6.159654 | 3.774227 | 2.90E-97 | 1.79E-95 |  |
| COL1A1 | 1.246083 | 5.025606 | 3.779523 | 5.44E-90 | 3.31E-89 |  |
| SRPX | 1.989801 | 5.779018 | 3.789217 | 1.41E-94 | 1.73E-93 |  |
| AC018738.1 | 0.27025 | 4.080664 | 3.810414 | ###### | ###### |  |
| SNRPGP2 | 1.02872 | 4.843657 | 3.814937 | 4.66E-98 | 1.61E-95 |  |
| CD44 | 2.456746 | 6.275324 | 3.818578 | 3.69E-84 | 1.61E-83 |  |
| COL4A2 | 2.202385 | 6.035959 | 3.833575 | 6.47E-95 | 9.04E-94 |  |
| FCGR3A | 2.533439 | 6.374482 | 3.841043 | 6.17E-85 | 2.79E-84 |  |
| PLVAP | 0.705313 | 4.566326 | 3.861013 | 3.40E-97 | 1.85E-95 |  |
| FTL | 10.18627 | 14.05877 | 3.872499 | 2.20E-95 | 3.80E-94 |  |
| TPX2 | 0.586574 | 4.46252 | 3.875947 | 1.23E-97 | 1.61E-95 |  |
| CDC20 | 0.539654 | 4.422257 | 3.882603 | 3.28E-97 | 1.82E-95 |  |
| MMP2 | 1.323555 | 5.207061 | 3.883506 | 1.80E-97 | 1.61E-95 |  |
| MTCO1P40 | 0.469478 | 4.366489 | 3.897011 | 5.68E-94 | 5.72E-93 |  |
| PBK | 0.099942 | 4.016219 | 3.916277 | 3.98E-98 | 1.61E-95 |  |
| HMOX1 | 2.352586 | 6.284257 | 3.931671 | 1.98E-92 | 1.53E-91 |  |
| S100A11 | 4.380078 | 8.312706 | 3.932628 | 1.37E-92 | 1.08E-91 |  |
| NUSAP1 | 0.649021 | 4.58857 | 3.939549 | 1.62E-97 | 1.61E-95 |  |
| TIMP1 | 4.56508 | 8.505523 | 3.940443 | 1.61E-88 | 8.90E-88 |  |
| BIRC5 | 0.353638 | 4.315068 | 3.96143 | 3.33E-95 | 5.25E-94 |  |
| PSPHP1 | 0.151161 | 4.119548 | 3.968387 | ###### | ###### |  |
| CD14 | 2.922072 | 6.892794 | 3.970721 | 3.02E-92 | 2.29E-91 |  |
| TMSB4XP1 | 0.47043 | 4.501811 | 4.031381 | ###### | ###### |  |
| RPL13AP25 | 0.213459 | 4.259328 | 4.045869 | 1.50E-98 | 6.40E-96 |  |
| CRISPLD1 | 1.084479 | 5.137727 | 4.053248 | 3.79E-97 | 1.95E-95 |  |
| RPS18P12 | 0.120083 | 4.18875 | 4.068667 | ###### | ###### |  |
| TNFRSF12A | 1.576184 | 5.652119 | 4.075935 | 1.45E-90 | 9.25E-90 |  |
| MYBL2 | 0.086768 | 4.277005 | 4.190237 | 1.20E-97 | 1.61E-95 |  |
| MT-TC | 0.015871 | 4.219662 | 4.203791 | ###### | ###### |  |
| LAPTM5 | 3.343078 | 7.582229 | 4.23915 | 1.37E-92 | 1.08E-91 |  |
| AL355974.2 | 0.986697 | 5.248267 | 4.26157 | 1.40E-93 | 1.30E-92 |  |
| GAPDHP1 | 0.835321 | 5.100981 | 4.265659 | 2.24E-97 | 1.62E-95 |  |
| NMB | 2.32984 | 6.596049 | 4.266208 | 1.83E-94 | 2.16E-93 |  |
| TOP2A | 0.200369 | 4.467971 | 4.267603 | 1.88E-97 | 1.61E-95 |  |
| MIR621 | 0.01358 | 4.349876 | 4.336295 | ###### | ###### |  |
| RPL41P5 | 0.018198 | 4.357408 | 4.33921 | ###### | ###### |  |
| PDPN | 2.157258 | 6.519876 | 4.362617 | 4.61E-94 | 4.79E-93 |  |
| HLA-DRB5 | 1.772226 | 6.143857 | 4.371632 | 4.43E-86 | 2.12E-85 |  |
| IGFBP5 | 3.511295 | 7.882949 | 4.371654 | 4.92E-97 | 2.11E-95 |  |
| EGFR | 1.870025 | 6.265314 | 4.395289 | 3.49E-94 | 3.76E-93 |  |
| MMP14 | 1.694486 | 6.104176 | 4.40969 | 3.70E-97 | 1.93E-95 |  |
| RPLP0P6 | 1.418364 | 5.828971 | 4.410606 | 1.22E-97 | 1.61E-95 |  |
| COL4A1 | 1.673727 | 6.161622 | 4.487895 | 6.15E-96 | 1.38E-94 |  |
| F2R | 0.53313 | 5.0365 | 4.503371 | 1.22E-97 | 1.61E-95 |  |
| C1QB | 4.084112 | 8.605784 | 4.521673 | 6.32E-91 | 4.16E-90 |  |
| TREM2 | 2.187009 | 6.726658 | 4.539648 | 8.42E-95 | 1.12E-93 |  |
| FCGBP | 0.704642 | 5.252312 | 4.547671 | 3.94E-96 | 9.54E-95 |  |
| IGFBP2 | 2.46011 | 7.049289 | 4.589179 | 1.03E-93 | 9.84E-93 |  |
| NES | 2.985022 | 7.584119 | 4.599097 | 4.00E-97 | 1.95E-95 |  |
| AC016596.2 | 0.009659 | 4.648503 | 4.638845 | ###### | ###### |  |
| LTF | 0.724392 | 5.376363 | 4.651971 | 1.89E-90 | 1.19E-89 |  |
| C1QA | 3.456545 | 8.159788 | 4.703243 | 2.64E-93 | 2.32E-92 |  |
| VSIG4 | 1.718179 | 6.431297 | 4.713118 | 2.26E-93 | 2.02E-92 |  |
| CHI3L2 | 1.477703 | 6.197771 | 4.720068 | 6.65E-84 | 2.87E-83 |  |
| COL3A1 | 0.730535 | 5.533206 | 4.80267 | 8.09E-97 | 2.86E-95 |  |
| UBE2C | 0.293597 | 5.104876 | 4.811279 | 1.20E-97 | 1.61E-95 |  |
| CPXM1 | 0.395492 | 5.259772 | 4.864281 | 1.40E-97 | 1.61E-95 |  |
| HLA-DRB1 | 3.481509 | 8.421388 | 4.939879 | 1.16E-95 | 2.35E-94 |  |
| SERPINE1 | 1.029054 | 6.021564 | 4.99251 | 5.25E-94 | 5.35E-93 |  |
| C1QC | 3.497689 | 8.624667 | 5.126978 | 9.52E-95 | 1.24E-93 |  |
| SPP1 | 6.042376 | 11.25553 | 5.213156 | 1.79E-85 | 8.30E-85 |  |
| TNC | 1.092756 | 6.440167 | 5.347411 | 2.98E-97 | 1.79E-95 |  |
| METTL7B | 1.012977 | 6.372524 | 5.359548 | 7.66E-96 | 1.65E-94 |  |
| HLA-DRA | 4.432379 | 9.911258 | 5.478879 | 3.06E-96 | 7.85E-95 |  |
| CHI3L1 | 4.527529 | 10.15093 | 5.623396 | 9.54E-79 | 3.39E-78 |  |
| MIR3682 | 0.022934 | 5.773995 | 5.751061 | ###### | ###### |  |
| RPL41P1 | 0.025663 | 6.49987 | 6.474207 | ###### | ###### |  |
| TMSB4XP8 | 1.586075 | 8.733074 | 7.146999 | ###### | ###### |  |
| MT-TP | 0.047954 | 9.366899 | 9.318944 | ###### | ###### |  |

###### represents the sample with missing data.

**Supplementary Table S3.** The C1R,CCL2 and TNFRSF1A expression levels with the normal and GBM tissues.

| ID | C1R | CCL2 | TNFRSF1A | Type |
| --- | --- | --- | --- | --- |
| GTEX-13QIC-0011-R1a-SM-5O9CJ | 2.43072516 | 3.0598685 | 3.4450667 | Normal |
| GTEX-N7MS-2526-SM-26GMA | 1.03963472 | 0.2463975 | 1.207624 | Normal |
| GTEX-N7MS-2526-SM-26GMR | 0.78214343 | 0.4111304 | 1.4219147 | Normal |
| GTEX-NPJ7-0011-R6a-SM-2I3G7 | 3.09250889 | 0.8456554 | 3.737217 | Normal |
| GTEX-132Q8-3026-SM-5PNVG | 4.92365397 | 1.9332938 | 4.5877127 | Normal |
| GTEX-NPJ7-2726-SM-2I3FT | 4.23544262 | 2.2785808 | 5.0953537 | Normal |
| GTEX-13OVJ-0011-R9b-SM-5L3GD | 3.8180154 | 6.1275613 | 4.6428754 | Normal |
| GTEX-XLM4-0011-R10A-SM-4AT5P | 3.55895275 | 2.8106575 | 3.6168476 | Normal |
| GTEX-12ZZZ-0011-R10a-SM-5P9HC | 1.99691052 | 1.8932489 | 3.0989723 | Normal |
| GTEX-X4XY-0011-R8A-SM-46MVC | 2.9425634 | 5.9724829 | 4.6310697 | Normal |
| GTEX-13QJC-0011-R6a-SM-5S2VI | 2.66134445 | 1.1353085 | 3.9214868 | Normal |
| GTEX-ZUA1-0011-R8b-SM-51MST | 2.41541233 | 2.2215418 | 3.0989723 | Normal |
| GTEX-13FHP-0011-R7b-SM-5LZYI | 3.04542505 | 1.4495555 | 3.4509409 | Normal |
| GTEX-11EMC-3326-SM-5P9JH | 2.05680396 | 0.4143508 | 3.1531519 | Normal |
| GTEX-13112-0011-R7b-SM-5DUVW | 2.35514438 | 1.415191 | 3.2553114 | Normal |
| GTEX-12WSA-0011-R5b-SM-5GU5I | 3.8722518 | 2.1216958 | 3.6505586 | Normal |
| GTEX-ZF28-3026-SM-4WKHP | 3.35232086 | 1.2965625 | 4.111355 | Normal |
| GTEX-ZUA1-0011-R7b-SM-4YCDP | 2.50278562 | 1.4435348 | 2.8136609 | Normal |
| GTEX-11ZVC-0011-R9a-SM-57WC5 | 2.51049677 | 2.7337647 | 3.3838025 | Normal |
| GTEX-WZTO-0011-R4A-SM-3NMC7 | 1.92428855 | 1.4914996 | 2.7315588 | Normal |
| GTEX-NPJ7-0011-R11A-SM-2I3E8 | 2.1159785 | 0.624142 | 2.8979794 | Normal |
| GTEX-13OVL-0011-R6a-SM-5L3G4 | 2.38219028 | 1.5345205 | 2.7878519 | Normal |
| GTEX-13NYB-0011-R1b-SM-5KM4F | 2.51242465 | 2.2389991 | 3.0606112 | Normal |
| GTEX-WHSE-0011-R8A-SM-3P5Z1 | 2.9468442 | 7.1276785 | 4.2814129 | Normal |
| GTEX-T6MN-0011-R4A-SM-32QPG | 2.5159861 | 2.2664287 | 3.7107032 | Normal |
| GTEX-ZDXO-0011-R1a-SM-4WKF4 | 2.64800384 | 2.0872966 | 3.303122 | Normal |
| GTEX-11DXY-3226-SM-5GIDE | 3.17738186 | 2.0001823 | 3.208221 | Normal |
| GTEX-ZV68-0011-R5a-SM-4YCDW | 3.87847059 | 1.448014 | 4.1829693 | Normal |
| GTEX-11GSP-0011-R6b-SM-57WBQ | 3.92024552 | 3.9825367 | 4.0599992 | Normal |
| GTEX-13OVL-0011-R3b-SM-5L3I8 | 2.63607395 | 2.4181315 | 3.2140474 | Normal |
| GTEX-13OW7-0011-R5b-SM-5O9DM | 2.63495509 | 2.0875579 | 3.3346349 | Normal |
| GTEX-11WQK-3126-SM-5EGI2 | 4.08039431 | 4.3996271 | 4.3825767 | Normal |
| GTEX-ZE9C-0011-R11a-SM-4WKGG | 1.37525107 | 1.3364425 | 1.90109 | Normal |
| GTEX-ZZPT-2926-SM-5EQ5S | 3.21591133 | 1.5229064 | 3.1427558 | Normal |
| GTEX-12ZZY-3026-SM-5GCOU | 2.27746974 | 0.8124197 | 2.9002455 | Normal |
| GTEX-WHSE-2926-SM-3NMBG | 2.68832912 | 4.3178208 | 4.412416 | Normal |
| GTEX-12ZZW-2926-SM-5LZUP | 2.99104314 | 0.4974023 | 3.753717 | Normal |
| GTEX-12WSD-0011-R9a-SM-5GU6W | 2.17784305 | 2.1830099 | 3.5410323 | Normal |
| GTEX-YFC4-0011-R11a-SM-4SOK6 | 2.04751579 | 1.4477647 | 2.4416515 | Normal |
| GTEX-X261-0011-R5A-SM-3NMB4 | 4.63591415 | 1.9576833 | 4.7851998 | Normal |
| GTEX-11OC5-0626-SM-5HL6M | 4.15937897 | 3.7218571 | 4.3433327 | Normal |
| GTEX-ZVZQ-0011-R7b-SM-57WBB | 2.29731611 | 2.3086147 | 3.0945479 | Normal |
| GTEX-12ZZX-0011-R8a-SM-5DUW8 | 2.44509235 | 1.3452019 | 3.3841099 | Normal |
| GTEX-13PDP-0011-R1a-SM-5PNX5 | 2.7386493 | 2.9539146 | 4.772397 | Normal |
| GTEX-11TTK-2926-SM-5PNYP | 4.41110611 | 4.4759768 | 5.4486975 | Normal |
| GTEX-N7MS-0011-R3a-SM-5SI8H | 2.22369641 | 1.5950442 | 2.0751584 | Normal |
| GTEX-T6MN-2626-SM-32PMQ | 2.48851001 | 1.1422758 | 2.6197757 | Normal |
| GTEX-12WS9-0011-R5b-SM-5P9EV | 5.31613366 | 7.4171168 | 5.3697896 | Normal |
| GTEX-NL4W-0011-R9a-SM-2I3G1 | 2.99375348 | 4.8135392 | 4.9836716 | Normal |
| GTEX-13X6J-3026-SM-5Q5CU | 2.92467013 | 2.0350578 | 3.1495811 | Normal |
| GTEX-145LS-0011-R10a-SM-5PNUQ | 2.35207941 | 0.9226307 | 3.1507264 | Normal |
| GTEX-13OVL-0011-R10a-SM-5L3GS | 2.40065558 | 1.8231091 | 2.7304667 | Normal |
| GTEX-14BIL-0011-R10a-SM-5SI75 | 2.16541993 | 0.2933898 | 3.2329917 | Normal |
| GTEX-13FLV-0011-R10b-SM-5LZZ2 | 2.76899363 | 1.6793566 | 3.2313765 | Normal |
| GTEX-13FLW-0011-R6b-SM-5L3EN | 2.20179677 | 0.909992 | 2.6083181 | Normal |
| GTEX-NL4W-0011-R10A-SM-2I3DY | 4.77159555 | 4.3724161 | 5.3356748 | Normal |
| GTEX-S7SE-0011-R7A-SM-2XCDI | 2.77895304 | 1.5426233 | 2.7489463 | Normal |
| GTEX-139UC-0011-R7a-SM-5IJCV | 2.63945378 | 1.113873 | 3.268614 | Normal |
| GTEX-RNOR-0011-R4A-SM-3GAD3 | 2.97561567 | 1.9662833 | 3.4767223 | Normal |
| GTEX-13FHO-2926-SM-5L3ES | 2.44333776 | 1.9317116 | 3.4229925 | Normal |
| GTEX-ZE7O-0011-R1a-SM-57WDM | 2.26668109 | 3.1684623 | 3.6957755 | Normal |
| GTEX-11ZUS-2826-SM-5EQKW | 1.64255277 | 0.9868124 | 1.4377391 | Normal |
| GTEX-WVLH-0011-R11A-SM-3MJFO | 1.69085357 | 0.2416441 | 1.5010344 | Normal |
| GTEX-13IVO-0011-R5a-SM-5L3CY | 3.56885856 | 6.0040723 | 5.7568655 | Normal |
| GTEX-11WQC-0011-R5a-SM-5BC74 | 3.12703771 | 2.2361722 | 3.7831389 | Normal |
| GTEX-R55F-1326-SM-5S2V4 | 4.29176343 | 1.9527779 | 4.5078719 | Normal |
| GTEX-T6MN-0011-R7A-SM-5CHSP | 0.01972963 | 0.0197296 | 0.0197296 | Normal |
| GTEX-RNOR-0011-R3A-SM-5SI8E | 2.04165265 | 1.6162598 | 2.3235196 | Normal |
| GTEX-13QIC-0011-R10a-SM-5O9C7 | 2.64220503 | 1.0876462 | 2.7993192 | Normal |
| GTEX-13QJC-0011-R7b-SM-5PNUM | 4.0545288 | 1.2971214 | 4.5310568 | Normal |
| GTEX-12WSD-3126-SM-5HL7P | 2.89632288 | 1.3934979 | 3.717009 | Normal |
| GTEX-13OVL-0011-R4b-SM-5L3HV | 2.7881108 | 2.8797875 | 3.8832968 | Normal |
| GTEX-13X6I-0011-R2b-SM-5PNWQ | 3.92201851 | 3.153291 | 4.559134 | Normal |
| GTEX-1313W-0011-R8a-SM-5DUVM | 2.10796464 | 1.6634262 | 3.0533125 | Normal |
| GTEX-12ZZX-0011-R2a-SM-5EGLG | 3.12470708 | 1.2772999 | 4.2556987 | Normal |
| GTEX-X261-0011-R11A-SM-4E3JY | 2.3575916 | 0.6684106 | 2.2884621 | Normal |
| GTEX-13X6J-0011-R8b-SM-5PNUA | 2.25642129 | 1.8132923 | 3.1608934 | Normal |
| GTEX-ZF28-0011-R11a-SM-4WWEI | 2.30289065 | 0.6449737 | 3.0093281 | Normal |
| GTEX-ZF28-2926-SM-4WKG1 | 2.92943344 | 0.716458 | 3.955986 | Normal |
| GTEX-13OVJ-0011-R3b-SM-5P9H6 | 4.13075083 | 7.557525 | 5.430712 | Normal |
| GTEX-XOTO-0011-R11B-SM-4B64O | 1.40465021 | 0.405114 | 1.8724018 | Normal |
| GTEX-ZVZQ-0011-R9a-SM-51MRH | 3.40201924 | 3.8185533 | 3.877565 | Normal |
| GTEX-X261-0011-R7A-SM-4E3JJ | 4.92192101 | 2.3899559 | 5.3072533 | Normal |
| GTEX-ZYY3-3026-SM-5GIEJ | 2.20165334 | 0.99914 | 3.0517301 | Normal |
| GTEX-12WSM-0011-R1a-SM-5LZW6 | 2.8379511 | 1.6205907 | 3.4163634 | Normal |
| GTEX-13IVO-0011-R3b-SM-5IJBJ | 3.3037319 | 2.9105819 | 5.2045431 | Normal |
| GTEX-13CZV-0011-R10b-SM-5LZYD | 2.27612293 | 1.3185031 | 3.1228726 | Normal |
| GTEX-145MI-0011-R7b-SM-5Q5AZ | 3.49744 | 3.111034 | 4.571736 | Normal |
| GTEX-13OW7-0011-R3a-SM-5O9DA | 3.18712356 | 1.8413139 | 3.7479905 | Normal |
| GTEX-QVJO-0011-R7A-SM-2S1QO | 3.463694 | 0.636576 | 2.911136 | Normal |
| GTEX-RNOR-0011-R1A-SM-5SI8F | 2.52165814 | 1.2289018 | 2.7156022 | Normal |
| GTEX-13JVG-0011-R11a-SM-5KM53 | 1.5592684 | 0.9625552 | 2.2127134 | Normal |
| GTEX-11WQC-0011-R6a-SM-5BC77 | 2.64708798 | 0.9186618 | 3.4333803 | Normal |
| GTEX-13JUV-0011-R9a-SM-5LZX3 | 3.66005732 | 4.9063873 | 4.3949173 | Normal |
| GTEX-WL46-0011-R7A-SM-3LK7X | 3.08548826 | 2.2094051 | 2.8418681 | Normal |
| GTEX-13FTZ-0011-R10b-SM-5KLZS | 2.58799518 | 1.8903821 | 3.4327811 | Normal |
| GTEX-145MH-0011-R10b-SM-5PNUK | 2.41160079 | 0.9299545 | 2.2794925 | Normal |
| GTEX-12WSF-0011-R6b-SM-5HL8W | 4.03296897 | 4.2665181 | 4.3426905 | Normal |
| GTEX-13FXS-0011-R2b-SM-5K7XX | 3.45340435 | 2.9591464 | 4.0045544 | Normal |
| GTEX-RNOR-0011-R5A-SM-2TF4J | 1.80158309 | 1.6806223 | 2.7131941 | Normal |
| GTEX-TSE9-0011-R9A-SM-3DB7Q | 2.80924981 | 2.5962035 | 3.7649137 | Normal |
| GTEX-RVPU-0011-R11A-SM-2XCAF | 3.082371 | 1.475314 | 3.52084 | Normal |
| GTEX-X4EP-0011-R6A-SM-3P629 | 6.26006554 | 6.146102 | 5.974969 | Normal |
| GTEX-Y8DK-0011-R3A-SM-4RTW5 | 2.26328517 | 1.0325952 | 2.8860134 | Normal |
| GTEX-13IVO-2926-SM-5L3CZ | 3.26468086 | 2.2196789 | 5.155974 | Normal |
| GTEX-ZVT3-0011-R11b-SM-57WBI | 1.16832172 | 0.8935996 | 1.7463476 | Normal |
| GTEX-13OVL-3026-SM-5IJF1 | 2.01068006 | 0.6585983 | 2.5261615 | Normal |
| GTEX-1445S-0011-R5a-SM-5PNUS | 4.49363094 | 4.0217265 | 5.6998807 | Normal |
| GTEX-13OVJ-0011-R7a-SM-5L3G1 | 4.42045916 | 5.6031334 | 5.0151167 | Normal |
| GTEX-13OVH-0011-R11b-SM-5KM4X | 1.90769715 | 0.6915038 | 2.1687155 | Normal |
| GTEX-11WQC-0011-R10a-SM-57WCT | 2.60063697 | 0.7769289 | 3.4983657 | Normal |
| GTEX-11NV4-2126-SM-5N9DS | 2.23061256 | 1.5956209 | 3.1427558 | Normal |
| GTEX-13X6I-0011-R3b-SM-5P9HM | 3.90923035 | 1.7662808 | 4.020441 | Normal |
| GTEX-Y111-2826-SM-4TT3O | 1.51097153 | 0.1834469 | 1.6973171 | Normal |
| GTEX-13O3O-0011-R3b-SM-5KM3R | 3.04987481 | 4.2679421 | 3.7982564 | Normal |
| GTEX-ZE9C-0011-R9a-SM-4WWCY | 4.60811963 | 6.0640235 | 4.3210697 | Normal |
| GTEX-13X6K-0011-R11a-SM-5P9F5 | 2.36319196 | 0.9916171 | 2.911008 | Normal |
| GTEX-12ZZX-0011-R10b-SM-5DUWK | 2.24928525 | 0.8055101 | 3.1281768 | Normal |
| GTEX-12WSA-0011-R8a-SM-5P9EX | 3.64125102 | 2.3794876 | 3.9072597 | Normal |
| GTEX-X4XX-0011-R1B-SM-3P622 | 3.67489513 | 3.2400556 | 4.3787188 | Normal |
| GTEX-13JVG-0011-R3b-SM-5LZXF | 3.35455168 | 3.5482073 | 4.3823667 | Normal |
| GTEX-ZAB4-0011-R6a-SM-4SOKD | 2.56124691 | 1.4345614 | 3.5941581 | Normal |
| GTEX-N7MT-0011-R2a-SM-2I3GI | 3.62962767 | 2.4874111 | 3.9919628 | Normal |
| GTEX-13OW6-2926-SM-5KM29 | 1.5648047 | 0.7697909 | 2.2955416 | Normal |
| GTEX-12ZZW-0011-R5a-SM-5DUVN | 2.88362161 | 1.5327349 | 3.7816329 | Normal |
| GTEX-S7SE-0011-R4A-SM-2XCDB | 2.28694888 | 2.2105501 | 3.4380987 | Normal |
| GTEX-ZXG5-0011-R10a-SM-57WDD | 2.2543719 | 0.7687307 | 3.1114723 | Normal |
| GTEX-13FLV-0011-R6a-SM-5KLZ4 | 2.6464241 | 1.8653449 | 3.0854883 | Normal |
| GTEX-ZAB4-0011-R7a-SM-4SOKE | 2.97149537 | 0.99914 | 2.7949953 | Normal |
| GTEX-OHPN-0011-R10A-SM-33HBU | 5.69539141 | 4.8561847 | 5.1828897 | Normal |
| GTEX-13RTJ-0011-R7b-SM-5P9JS | 3.36808087 | 0.8029546 | 3.4722363 | Normal |
| GTEX-12WSC-2926-SM-5BC5Z | 1.23502535 | 0.4135098 | 1.7193451 | Normal |
| GTEX-13OVJ-0011-R10b-SM-5L3HT | 4.08019091 | 6.1504607 | 5.1825679 | Normal |
| GTEX-12ZZX-0011-R5a-SM-5HL89 | 3.07559684 | 1.0637814 | 3.9548881 | Normal |
| GTEX-145LS-0011-R7a-SM-5PNWM | 3.18028848 | 1.1967675 | 3.2645263 | Normal |
| GTEX-N7MT-1226-SM-2TC6K | 2.16818733 | 0.1272075 | 3.0465825 | Normal |
| GTEX-X261-0011-R6B-SM-4E3J8 | 4.40418736 | 2.4731386 | 5.011088 | Normal |
| GTEX-13X6K-0011-R4b-SM-5P9HO | 3.81335027 | 2.9261946 | 4.5261713 | Normal |
| GTEX-1445S-0011-R8a-SM-5PNWX | 3.56502861 | 5.1217223 | 5.8500331 | Normal |
| GTEX-1128S-2826-SM-5N9DI | 2.00730618 | 0.2324607 | 2.7379544 | Normal |
| GTEX-WWYW-0011-R6A-SM-3NB3G | 4.25609646 | 3.0899726 | 4.9635813 | Normal |
| GTEX-147GR-0011-R4a-SM-5S2V2 | 2.42147065 | 3.0162163 | 3.7478254 | Normal |
| GTEX-12WSB-0011-R6b-SM-5GU6J | 3.85601181 | 2.3292557 | 4.2073236 | Normal |
| GTEX-ZAK1-3026-SM-5S2MJ | 2.33624992 | 1.6112302 | 3.2723329 | Normal |
| GTEX-13S7M-0011-R7a-SM-5O9DK | 2.96567307 | 1.1084865 | 3.5853323 | Normal |
| GTEX-ZVT3-0011-R10b-SM-57WB6 | 2.2984392 | 0.7360776 | 3.3059374 | Normal |
| GTEX-QDT8-0011-R6A-SM-32PKI | 2.94373148 | 6.3391504 | 4.4954537 | Normal |
| GTEX-11PRG-2826-SM-5BC54 | 2.14413023 | 0.7425102 | 2.1188815 | Normal |
| GTEX-ZE7O-0011-R6a-SM-57WCI | 2.86560559 | 2.4997402 | 3.6437147 | Normal |
| GTEX-12WSM-0011-R8a-SM-5DUWJ | 2.81883673 | 1.0100972 | 3.9286425 | Normal |
| GTEX-13PDP-0011-R8b-SM-5O9CS | 2.77743996 | 4.7116478 | 4.4900332 | Normal |
| GTEX-13NYB-0011-R8a-SM-5KM4R | 2.21380811 | 1.8203772 | 2.5285393 | Normal |
| GTEX-P44H-0011-R5A-SM-2XCEX | 3.29908323 | 2.5937012 | 4.1477334 | Normal |
| GTEX-13SLW-0011-R10a-SM-5S2UI | 2.88786329 | 2.1768312 | 4.1133116 | Normal |
| GTEX-Q2AG-0011-R9A-SM-2HMJ6 | 2.39187235 | 2.0820732 | 3.4644675 | Normal |
| GTEX-14BIM-0011-R6b-SM-5S2VB | 3.82970202 | 1.7645217 | 3.9188267 | Normal |
| GTEX-144FL-3026-SM-5O99C | 5.96507816 | 5.3262854 | 5.8897536 | Normal |
| GTEX-13FLV-0011-R8a-SM-5LZZE | 3.11561752 | 3.8720738 | 3.8573662 | Normal |
| GTEX-13CF2-0011-R8b-SM-5IJCX | 3.04162051 | 2.5316361 | 3.4537156 | Normal |
| GTEX-131YS-3126-SM-5KLYT | 2.17281547 | 1.0908967 | 3.2529204 | Normal |
| GTEX-13OW7-3026-SM-5L3GY | 2.71140624 | 0.8782326 | 3.4806139 | Normal |
| GTEX-13SLX-0011-R8b-SM-5PNWZ | 2.36305721 | 1.8946064 | 3.281988 | Normal |
| GTEX-139T8-0011-R4a-SM-5HL54 | 3.50040241 | 4.8935338 | 4.5012893 | Normal |
| GTEX-145LU-0011-R3a-SM-5P9K2 | 3.09440925 | 1.2483914 | 3.4363779 | Normal |
| GTEX-WHSE-0011-R2A-SM-3P5ZL | 2.81264648 | 7.268383 | 4.4638956 | Normal |
| GTEX-13FXS-0011-R5b-SM-5LZYE | 2.48584738 | 1.8727737 | 3.0050483 | Normal |
| GTEX-13N2G-0011-R9b-SM-5MR4E | 3.8126634 | 2.744997 | 4.3292624 | Normal |
| GTEX-13O3O-3126-SM-5KM3H | 2.70874459 | 2.4101876 | 3.7744275 | Normal |
| GTEX-13NZA-0011-R11b-SM-5KM4W | 1.39161059 | 0.4464322 | 1.7041315 | Normal |
| GTEX-13FLV-0011-R11a-SM-5LZZ7 | 1.65384639 | 0.3767405 | 2.1279886 | Normal |
| GTEX-13OW8-0011-R3a-SM-5L3I4 | 3.16163681 | 4.0041778 | 4.0975228 | Normal |
| GTEX-S7SE-0011-R5A-SM-2XCDA | 2.98182409 | 1.7590011 | 3.1366774 | Normal |
| GTEX-13X6I-0011-R5a-SM-5PNWW | 4.25733155 | 3.2983149 | 4.3494104 | Normal |
| GTEX-NPJ7-0011-R8a-SM-2I3G2 | 2.69090515 | 1.8152429 | 3.8036125 | Normal |
| GTEX-X4XX-0011-R8B-SM-46MWM | 2.29829556 | 2.4359404 | 3.8145785 | Normal |
| GTEX-WVLH-0011-R5A-SM-3MJFW | 2.67882847 | 1.0690556 | 3.0472991 | Normal |
| GTEX-13FLW-0011-R5a-SM-5LZX6 | 2.45681288 | 1.5816412 | 3.1186922 | Normal |
| GTEX-R55E-2526-SM-2TC6H | 3.87443742 | 3.4606302 | 3.1407578 | Normal |
| GTEX-11ZTS-3226-SM-5EGID | 2.19600493 | 0.5410449 | 2.6906153 | Normal |
| GTEX-13N2G-0011-R4b-SM-5MR54 | 4.92135769 | 5.3341891 | 4.9225048 | Normal |
| GTEX-YJ89-0011-R7a-SM-4V6GO | 3.06590432 | 1.5541098 | 3.2971493 | Normal |
| GTEX-UTHO-0011-R3A-SM-3GIK8 | 1.9425018 | 2.4501717 | 3.6562848 | Normal |
| GTEX-13O3Q-0011-R6a-SM-5KM2V | 2.36415804 | 1.3409697 | 2.9706505 | Normal |
| GTEX-N7MT-0011-R4a-SM-2I3G9 | 3.3323758 | 1.9206684 | 4.3877687 | Normal |
| GTEX-ZXG5-0011-R7b-SM-57WCC | 2.87783061 | 1.8765148 | 3.6507234 | Normal |
| GTEX-ZAB4-0011-R5a-SM-4SOKC | 3.06902196 | 0.9391741 | 3.0087695 | Normal |
| GTEX-X4EP-0011-R7B-SM-4PQZW | 5.75529708 | 4.7086573 | 4.4945184 | Normal |
| GTEX-139UC-0011-R6b-SM-5K7Z1 | 2.6464241 | 1.0435415 | 3.5222658 | Normal |
| GTEX-12WSC-0011-R9a-SM-5GU4U | 3.04148187 | 2.038358 | 3.2761777 | Normal |
| GTEX-P44G-0011-R5A-SM-2I3FA | 3.82883987 | 6.5598245 | 4.8249865 | Normal |
| GTEX-11WQK-0011-R9b-SM-5BC6N | 3.4728512 | 5.7780205 | 4.6811715 | Normal |
| GTEX-X4EP-0011-R2B-SM-3P625 | 6.08047228 | 6.8018633 | 5.479435 | Normal |
| GTEX-13NYB-3226-SM-5J2ND | 1.59319034 | 2.1578534 | 1.7239775 | Normal |
| GTEX-11H98-0011-R8a-SM-5NQ8V | 2.62264363 | 3.4859472 | 3.9797619 | Normal |
| GTEX-N7MS-2526-SM-2D7W3 | 1.25711085 | 0.5133141 | 2.0363787 | Normal |
| GTEX-13N1W-0011-R11a-SM-5MR3D | 2.61546506 | 3.8683582 | 3.4306506 | Normal |
| GTEX-13PLJ-0011-R9b-SM-5O9DV | 2.50371035 | 2.746988 | 3.2037232 | Normal |
| GTEX-13PLJ-0011-R5a-SM-5O9BG | 3.33658771 | 1.9431857 | 4.1138828 | Normal |
| GTEX-144GL-2926-SM-5O99F | 1.78735553 | 0.4525529 | 2.6918705 | Normal |
| GTEX-XOTO-2926-SM-4B65G | 1.64318042 | 1.2309547 | 2.5574883 | Normal |
| GTEX-S7SE-0011-R10A-SM-2XCDF | 2.14743917 | 0.9036212 | 2.2451975 | Normal |
| GTEX-139TT-2626-SM-5LZUB | 2.71686902 | 1.6194533 | 2.8088037 | Normal |
| GTEX-X4XX-0011-R2A-SM-3P623 | 2.90648507 | 2.3466156 | 3.5387877 | Normal |
| GTEX-OHPN-0011-R9A-SM-4DXUH | 4.4641134 | 4.7235093 | 4.619895 | Normal |
| GTEX-ZVZQ-0011-R11a-SM-51MS6 | 1.47647503 | 0.7242553 | 1.3614498 | Normal |
| GTEX-13CF2-0011-R7b-SM-5K7UV | 2.9921848 | 2.3309028 | 3.5584894 | Normal |
| GTEX-13PL6-0011-R6a-SM-5O9C5 | 3.64541256 | 1.802089 | 4.2295754 | Normal |
| GTEX-YFC4-0011-R2a-SM-4V6DZ | 4.67283666 | 5.3161337 | 4.2086989 | Normal |
| GTEX-QVJO-0011-R2A-SM-2S1QK | 3.07863257 | 1.7527407 | 3.8131811 | Normal |
| GTEX-131YS-0011-R9b-SM-5EQLY | 3.75170442 | 4.6026766 | 4.1917238 | Normal |
| GTEX-145MG-0011-R3b-SM-5SI67 | 3.04846393 | 1.7516687 | 3.4269622 | Normal |
| GTEX-OXRO-0011-R2A-SM-3NB1W | 4.18101436 | 4.8048313 | 5.5789987 | Normal |
| GTEX-QVUS-2826-SM-3GADB | 3.06158366 | 0.7054671 | 4.0599992 | Normal |
| GTEX-11GSP-0011-R5a-SM-57WBE | 3.13207208 | 3.8993571 | 3.2918056 | Normal |
| GTEX-ZVT3-0011-R3b-SM-51MTJ | 2.81883673 | 1.8331251 | 3.6287757 | Normal |
| GTEX-Z93S-0011-R11a-SM-4RGNN | 1.76784433 | 0.2524355 | 1.366807 | Normal |
| GTEX-11GS4-3126-SM-5A5LH | 2.41336673 | 1.3733406 | 3.2795774 | Normal |
| GTEX-13FHO-0011-R7b-SM-5LZXD | 2.98070346 | 4.3638406 | 4.5363519 | Normal |
| GTEX-13FHO-3026-SM-5J1O9 | 2.82346445 | 2.7845469 | 3.4755033 | Normal |
| GTEX-P44G-0011-R2A-SM-2XCD2 | 3.48299968 | 5.9467469 | 4.8024921 | Normal |
| GTEX-13RTL-0011-R1a-SM-5PNZ8 | 3.25073557 | 8.6091888 | 4.1576012 | Normal |
| GTEX-12WSF-0011-R11a-SM-5LZVT | 1.50479208 | 2.613407 | 2.5297097 | Normal |
| GTEX-13112-0011-R6b-SM-5DUVK | 2.24269672 | 1.3055557 | 3.0381832 | Normal |
| GTEX-13OVL-0011-R11b-SM-5L3G7 | 1.13810875 | 0.5397363 | 1.1742656 | Normal |
| GTEX-ZAJG-3126-SM-5HL9J | 3.84101951 | 4.3256994 | 4.4641134 | Normal |
| GTEX-P44G-0011-R1A-SM-2I3FE | 3.79185547 | 7.3097387 | 4.7439587 | Normal |
| GTEX-1192X-3126-SM-5N9BY | 3.08926504 | 0.7077362 | 3.8526373 | Normal |
| GTEX-13N2G-0011-R1b-SM-5MR3G | 3.14016415 | 2.2794925 | 3.614262 | Normal |
| GTEX-ZV68-0011-R6a-SM-51MSR | 3.65873331 | 2.0382351 | 3.9967417 | Normal |
| GTEX-QVUS-0011-R3A-SM-3GAFD | 3.14134641 | 1.2035948 | 2.8719253 | Normal |
| GTEX-OHPN-0011-R9A-SM-2YUMN | 4.50902126 | 4.661677 | 4.4965695 | Normal |
| GTEX-13SLW-0011-R1a-SM-5S2W7 | 3.35169569 | 1.9188076 | 4.3557096 | Normal |
| GTEX-N7MT-0011-R7a-SM-2I3FZ | 3.76134195 | 1.1003805 | 4.0124119 | Normal |
| GTEX-13IVO-0011-R8a-SM-5L3DY | 3.30871472 | 5.1934925 | 4.8142992 | Normal |
| GTEX-Q2AG-0011-R11A-SM-2HMKZ | 1.87005037 | 0.5216395 | 2.1259053 | Normal |
| GTEX-144GL-0011-R5b-SM-5PNUJ | 3.21619188 | 1.4020468 | 3.6163681 | Normal |
| GTEX-144GL-0011-R3a-SM-5P9HP | 2.79217403 | 1.0138192 | 3.2986307 | Normal |
| GTEX-R55E-0011-R9A-SM-2TC6C | 2.67126151 | 5.1123342 | 3.450004 | Normal |
| GTEX-13X6J-0011-R11a-SM-5P9HE | 1.48797894 | 0.6791335 | 1.581102 | Normal |
| GTEX-R55F-1226-SM-2TF59 | 2.16375834 | 0.2606239 | 2.1074312 | Normal |
| GTEX-13CZV-0011-R11b-SM-5N9FN | 1.69796515 | 1.6823546 | 2.8802094 | Normal |
| GTEX-144GO-0011-R5a-SM-5PNUR | 3.6666067 | 3.9449105 | 4.5437202 | Normal |
| GTEX-N7MT-0011-R3a-SM-2I3GC | 3.44103881 | 2.1537822 | 3.7816329 | Normal |
| GTEX-132Q8-0011-R10b-SM-5DUWZ | 4.44508763 | 1.0644585 | 3.4975855 | Normal |
| GTEX-13PL6-0011-R9b-SM-5O9CH | 3.15886439 | 3.52084 | 4.2149504 | Normal |
| GTEX-QMR6-0011-R11A-SM-32PKK | 1.69855788 | 0.1583118 | 1.8984442 | Normal |
| GTEX-ZDXO-0011-R8a-SM-4WWD7 | 3.42251662 | 3.298782 | 3.8194224 | Normal |
| GTEX-11EMC-3226-SM-5EGKW | 3.10486978 | 1.1763694 | 3.9583442 | Normal |
| GTEX-P44G-0011-R3A-SM-2I3FC | 2.83510312 | 5.1596539 | 3.930307 | Normal |
| GTEX-12ZZW-0011-R10b-SM-5HL9X | 2.89573262 | 0.5914363 | 3.4658579 | Normal |
| GTEX-12WSE-0011-R6b-SM-5LZV5 | 2.02966876 | 0.8628122 | 3.0982472 | Normal |
| GTEX-13FHP-0011-R1b-SM-5K7XL | 2.65206058 | 2.1610509 | 3.4803 | Normal |
| GTEX-XMD1-0011-R10A-SM-4AT4A | 4.41498881 | 1.7502221 | 4.3582896 | Normal |
| GTEX-12WSD-0011-R3b-SM-5LZWN | 2.37489226 | 1.8410514 | 3.207924 | Normal |
| GTEX-WZTO-0011-R8A-SM-4E3II | 1.97742046 | 1.9743868 | 2.4418034 | Normal |
| GTEX-13FXS-0011-R9a-SM-5K7UI | 3.88702384 | 5.094697 | 4.052624 | Normal |
| GTEX-13O3Q-0011-R10b-SM-5KM39 | 2.29595605 | 1.1773437 | 2.6518045 | Normal |
| GTEX-131YS-0011-R4a-SM-5DUVL | 2.73141689 | 1.4407632 | 3.8082866 | Normal |
| GTEX-13N2G-0011-R10a-SM-5MR34 | 5.67773569 | 7.1421536 | 5.584831 | Normal |
| GTEX-13OW8-0011-R6b-SM-5L3I3 | 3.53123749 | 3.8770714 | 4.5083363 | Normal |
| GTEX-13FLV-0011-R4a-SM-5LZYU | 3.49288575 | 1.7952883 | 4.3212811 | Normal |
| GTEX-ZVZQ-0011-R10b-SM-51MRT | 2.64178207 | 1.4272513 | 2.9447093 | Normal |
| GTEX-131XH-0011-R1a-SM-5DUWA | 2.34435143 | 2.3104175 | 2.9449914 | Normal |
| GTEX-P44H-0011-R10A-SM-2XCEK | 2.66913766 | 1.5015365 | 3.3750265 | Normal |
| GTEX-13OW7-0011-R4b-SM-5O9CX | 3.09640972 | 2.6628976 | 3.9676551 | Normal |
| GTEX-13OVH-0011-R6b-SM-5LUAX | 3.43731953 | 2.5613995 | 4.3679423 | Normal |
| GTEX-13NYS-0011-R9b-SM-5MR44 | 5.28676316 | 5.7138634 | 5.3976901 | Normal |
| GTEX-RU72-0011-R10A-SM-2TF6D | 2.61013095 | 1.4192998 | 4.2872023 | Normal |
| GTEX-13OVJ-2826-SM-5L3GW | 3.99674167 | 6.0728914 | 4.892692 | Normal |
| GTEX-11GSO-3026-SM-5Q5AL | 1.76183647 | 0.523777 | 2.3182625 | Normal |
| GTEX-WHSE-0011-R1A-SM-3P5ZK | 2.94842389 | 7.062313 | 4.2324414 | Normal |
| GTEX-T6MN-0011-R4A-SM-5CHSD | 0.01952402 | 0.019524 | 0.019524 | Normal |
| GTEX-ZUA1-3026-SM-59HJC | 2.31409787 | 1.167637 | 3.2846133 | Normal |
| GTEX-11GSP-0011-R8b-SM-5NQ79 | 3.32641961 | 5.0336857 | 4.2919747 | Normal |
| GTEX-YJ89-0011-R2b-SM-4RGLT | 3.13945778 | 2.9638251 | 3.8849274 | Normal |
| GTEX-Q2AG-0011-R10A-SM-2HMLA | 2.10704346 | 0.7699617 | 2.7073514 | Normal |
| GTEX-WL46-0011-R5A-SM-3LK6V | 2.70847154 | 2.3995451 | 2.7254973 | Normal |
| GTEX-145MG-0011-R7b-SM-5P9JY | 3.27026913 | 1.1236166 | 3.4702135 | Normal |
| GTEX-X4EP-0011-R9B-SM-4QASI | 5.06289365 | 7.4082889 | 5.0616556 | Normal |
| GTEX-13112-2926-SM-5DUWT | 1.57059294 | 0.7392441 | 2.161314 | Normal |
| GTEX-TSE9-0011-R7A-SM-5CHS2 | 0.01959585 | 0.0195959 | 0.0195959 | Normal |
| GTEX-11WQC-0011-R7b-SM-5BC7A | 3.32641961 | 2.370327 | 3.7568876 | Normal |
| GTEX-12ZZZ-0011-R7b-SM-5EGLE | 3.14147467 | 2.6928367 | 3.6685965 | Normal |
| GTEX-13RTL-0011-R3a-SM-5P9JP | 3.32985659 | 5.6855515 | 5.1149273 | Normal |
| GTEX-13FHO-0011-R11b-SM-5LZYQ | 1.51830486 | 1.1325751 | 2.6734345 | Normal |
| GTEX-Q2AG-0011-R5A-SM-2HMJH | 2.728391 | 1.1014418 | 3.5059298 | Normal |
| GTEX-13OVH-0011-R8b-SM-5MR35 | 2.94629904 | 1.4206794 | 3.9237924 | Normal |
| GTEX-147GR-0011-R6b-SM-5S2RO | 2.667463 | 1.3367031 | 3.737217 | Normal |
| GTEX-132Q8-0011-R5a-SM-5IJDM | 5.88975364 | 3.6368318 | 4.6438308 | Normal |
| GTEX-WZTO-0011-R5B-SM-3NMC5 | 2.59788341 | 1.4787005 | 2.6218385 | Normal |
| GTEX-11ONC-0011-R1a-SM-57WD4 | 3.04057606 | 3.3880512 | 4.177856 | Normal |
| GTEX-14BIN-0011-R10a-SM-5S2UA | 2.12298983 | 0.4975695 | 2.5018219 | Normal |
| GTEX-T2IS-0011-R6A-SM-32QP2 | 2.38194215 | 1.1675245 | 2.9958776 | Normal |
| GTEX-144GL-0011-R8a-SM-5Q5B1 | 2.1990113 | 2.2899916 | 3.1581792 | Normal |
| GTEX-13QIC-3026-SM-5LU58 | 1.68811169 | 0.4797888 | 2.0638097 | Normal |
| GTEX-1313W-0011-R10b-SM-5DUXA | 2.33384644 | 1.9922241 | 2.5992901 | Normal |
| GTEX-YFC4-0011-R9a-SM-4SOK4 | 3.62568906 | 4.3313508 | 3.8962773 | Normal |
| GTEX-13O3Q-2926-SM-5KM45 | 2.39242679 | 0.9348976 | 3.2404848 | Normal |
| GTEX-12126-0011-R9b-SM-5BC6P | 3.50216287 | 3.1892852 | 3.4351257 | Normal |
| GTEX-ZXG5-0011-R5b-SM-57WBN | 2.8713668 | 1.050351 | 3.4557018 | Normal |
| GTEX-13OW7-0011-R1b-SM-5L3HF | 2.23873206 | 3.4815653 | 3.4220674 | Normal |
| GTEX-13112-0011-R5b-SM-5DUV8 | 2.87992409 | 1.7379028 | 4.017623 | Normal |
| GTEX-T6MN-0011-R6A-SM-32QP8 | 2.11424501 | 3.5439366 | 2.4276004 | Normal |
| GTEX-12584-0011-R6a-SM-5NQ7C | 3.0885147 | 2.7627412 | 4.0318537 | Normal |
| GTEX-13PDP-2926-SM-5N9E8 | 1.62706647 | 0.7073576 | 3.0226345 | Normal |
| GTEX-ZE9C-0011-R5a-SM-5EGLO | 4.04122813 | 4.8846309 | 4.885728 | Normal |
| GTEX-139UC-0011-R3b-SM-5K7VZ | 2.46714997 | 1.8423792 | 3.4020192 | Normal |
| GTEX-12WSC-0011-R10a-SM-5GU57 | 2.03742001 | 1.658277 | 2.5781928 | Normal |
| GTEX-117XS-3126-SM-5GIDP | 1.68964861 | 0.5577734 | 1.9759851 | Normal |
| GTEX-XOTO-0011-R8A-SM-4B65J | 2.24533099 | 2.5639537 | 3.5156513 | Normal |
| GTEX-11H98-0011-R10b-SM-5NQ98 | 2.36275243 | 0.8268147 | 3.4883979 | Normal |
| GTEX-11OF3-0011-R7a-SM-57WCG | 2.96823033 | 1.884161 | 2.9263339 | Normal |
| GTEX-14ASI-3026-SM-5S2PN | 2.54761476 | 1.1293027 | 2.9522259 | Normal |
| GTEX-NPJ8-2626-SM-26GMI | 2.73836856 | 0 | 3.2343252 | Normal |
| GTEX-13CIG-0011-R9a-SM-5K7VU | 5.40245021 | 6.2289736 | 5.2936846 | Normal |
| GTEX-WWYW-0011-R1A-SM-3TW8G | 3.68346586 | 3.3661439 | 4.4857155 | Normal |
| GTEX-NL4W-0011-R6a-SM-2I3GA | 3.99123214 | 2.4726197 | 5.2108257 | Normal |
| GTEX-NPJ7-0011-R10A-SM-2I3E5 | 4.64775222 | 2.1228555 | 4.9337903 | Normal |
| GTEX-13NYB-0011-R7a-SM-5MR5D | 2.74246121 | 2.1339646 | 3.3495622 | Normal |
| GTEX-13FLW-0011-R8b-SM-5J1MZ | 1.93951637 | 1.590268 | 2.7625938 | Normal |
| GTEX-Q2AG-0011-R8A-SM-2HMK5 | 2.19152089 | 1.1379717 | 3.3774439 | Normal |
| GTEX-13X6K-0011-R3b-SM-5PNUD | 3.29180555 | 2.7409238 | 3.9182865 | Normal |
| GTEX-X585-0011-R1B-SM-46MVE | 2.90124246 | 2.8998225 | 3.4636939 | Normal |
| GTEX-13N2G-0011-R5a-SM-5MR33 | 6.30828148 | 7.1680457 | 6.1839813 | Normal |
| GTEX-RVPU-0011-R6A-SM-2XCAC | 3.756549 | 2.35528 | 4.743693 | Normal |
| GTEX-13X6I-0011-R11a-SM-5P9HN | 2.55222728 | 1.2314237 | 2.863103 | Normal |
| GTEX-13S7M-3126-SM-5RQJQ | 2.99361046 | 0.8763729 | 3.9478159 | Normal |
| GTEX-ZDXO-0011-R3a-SM-4WKFS | 2.63414779 | 1.4835855 | 3.1710864 | Normal |
| GTEX-13RTJ-0011-R3a-SM-5PNX7 | 3.3240555 | 0.9414727 | 3.6482369 | Normal |
| GTEX-139UC-0011-R4a-SM-5K7XM | 2.61288892 | 2.6901964 | 3.8818516 | Normal |
| GTEX-X261-0011-R8A-SM-4E3I5 | 4.97003741 | 5.4622911 | 4.3972554 | Normal |
| GTEX-12126-0926-SM-5FQTW | 1.82310908 | 0.5781716 | 2.3186855 | Normal |
| GTEX-14ASI-0011-R8b-SM-5S2UB | 1.95150633 | 2.0580611 | 2.9770425 | Normal |
| GTEX-14A5I-2826-SM-5SIBF | 2.61779475 | 0.1905235 | 3.2723329 | Normal |
| GTEX-12WSA-0011-R2a-SM-57WDK | 3.62207613 | 2.4469835 | 4.397679 | Normal |
| GTEX-NPJ7-0011-R1a-SM-3GACT | 2.87715012 | 1.1719406 | 4.2485477 | Normal |
| GTEX-13N2G-0011-R6b-SM-5MR3P | 4.81093729 | 3.6148994 | 4.96917 | Normal |
| GTEX-NPJ7-0011-R4a-SM-2I3GJ | 4.18576233 | 3.3229944 | 4.7883297 | Normal |
| GTEX-NL3H-0011-R6a-SM-2I3G8 | 2.59717825 | 1.0008178 | 3.4373195 | Normal |
| GTEX-ZAB4-0011-R10a-SM-4SOKH | 2.60107256 | 0.6318727 | 3.1081195 | Normal |
| GTEX-13SLW-0011-R11a-SM-5SI8Q | 1.35005015 | 0.4022269 | 2.1059957 | Normal |
| GTEX-11WQK-3026-SM-5EQL6 | 4.47144505 | 6.0361692 | 4.6772771 | Normal |
| GTEX-131XW-0011-R6b-SM-5K7XY | 3.5046801 | 1.5570478 | 4.5679887 | Normal |
| GTEX-N7MT-1126-SM-2YUNQ | 4.0267729 | 1.4360498 | 4.2849127 | Normal |
| GTEX-WL46-2926-SM-3LK82 | 2.22481691 | 1.4802016 | 2.7851976 | Normal |
| GTEX-ZDXO-2926-SM-4WKFM | 1.71692183 | 0.3103553 | 1.9639759 | Normal |
| GTEX-1399T-0011-R4b-SM-5DUVX | 3.1865303 | 2.564396 | 3.4966738 | Normal |
| GTEX-12ZZW-0011-R7a-SM-5DUWC | 2.98536326 | 1.7739631 | 3.6750675 | Normal |
| GTEX-1445S-0011-R2b-SM-5PNUH | 3.59499059 | 3.1840545 | 4.9168351 | Normal |
| GTEX-R55E-0011-R11A-SM-2TC6I | 1.98957095 | 3.9150276 | 2.0259628 | Normal |
| GTEX-QMR6-1326-SM-32PLB | 1.86852937 | 0.3932921 | 2.5393685 | Normal |
| GTEX-147F4-0011-R8b-SM-5S2UN | 3.96729279 | 8.4401375 | 5.47609 | Normal |
| GTEX-139TS-0011-R10a-SM-5K7TU | 2.8567188 | 2.2588413 | 3.5173994 | Normal |
| GTEX-12WSB-0011-R7b-SM-5GU6V | 4.1986606 | 4.7583013 | 4.4793529 | Normal |
| GTEX-13IVO-0011-R11b-SM-5LZXQ | 0.9025359 | 0.5118572 | 2.1955712 | Normal |
| GTEX-1212Z-0011-R1b-SM-5BC72 | 3.19743661 | 3.2523465 | 3.7042999 | Normal |
| GTEX-Q2AG-0011-R2A-SM-2HMIT | 2.10612894 | 1.1909865 | 3.0007494 | Normal |
| GTEX-11H98-0011-R5b-SM-57WCF | 3.01195393 | 0.9089568 | 3.9669246 | Normal |
| GTEX-ZV68-0011-R7a-SM-51MT4 | 4.19907198 | 2.3217485 | 4.0701091 | Normal |
| GTEX-12ZZY-0011-R10b-SM-5HL7W | 2.20580395 | 1.3216939 | 2.9850683 | Normal |
| GTEX-12WSC-0011-R5b-SM-5CVNS | 2.10449449 | 1.7699253 | 2.5182277 | Normal |
| GTEX-11GSO-2926-SM-5HL73 | 2.53605225 | 1.3464947 | 3.5340797 | Normal |
| GTEX-13O3O-0011-R10a-SM-5LUA9 | 2.56096756 | 2.1585687 | 3.088248 | Normal |
| GTEX-12WSF-0011-R1a-SM-5DUVI | 2.73268124 | 4.136401 | 3.6546366 | Normal |
| GTEX-12WSM-0011-R7b-SM-5EGLD | 2.96498512 | 0.9388784 | 3.618466 | Normal |
| GTEX-139UC-3126-SM-5J1OY | 2.53011758 | 1.301638 | 3.3138993 | Normal |
| GTEX-11GSP-0011-R10a-SM-5NQ7J | 3.21290334 | 3.2083611 | 3.4108922 | Normal |
| GTEX-ZF28-0011-R8a-SM-4WWFW | 3.94069254 | 1.7029538 | 4.6592382 | Normal |
| GTEX-RVPU-0011-R3A-SM-2XCAE | 3.47467505 | 1.3213885 | 4.1200233 | Normal |
| GTEX-RNOR-0011-R3A-SM-2TF5J | 2.836648 | 2.383031 | 2.59966 | Normal |
| GTEX-13OVJ-0011-R2b-SM-5L3GP | 4.58020403 | 6.7637697 | 5.3509423 | Normal |
| GTEX-QMR6-0011-R5A-SM-32PKT | 3.32746898 | 1.1644476 | 3.5725714 | Normal |
| GTEX-11WQC-0011-R11b-SM-57WD6 | 1.52016953 | 0.67394 | 2.0859567 | Normal |
| GTEX-11DXW-1026-SM-5H11K | 1.90452978 | 0.5919454 | 2.43114 | Normal |
| GTEX-ZE9C-0011-R2a-SM-4WKGH | 3.08769525 | 4.8854577 | 3.870672 | Normal |
| GTEX-14753-0011-R8b-SM-5S2U1 | 2.7478212 | 6.7693245 | 4.2733244 | Normal |
| GTEX-13FHP-3026-SM-5IJBS | 2.30289065 | 0.9082385 | 2.7687313 | Normal |
| GTEX-13VXU-0011-R7b-SM-5SI72 | 2.96397827 | 2.613555 | 3.2804922 | Normal |
| GTEX-12WSE-0011-R7a-SM-5PNWF | 3.2288736 | 1.7029538 | 3.9636314 | Normal |
| GTEX-X585-3026-SM-46MWF | 4.49725398 | 5.0741383 | 3.8105698 | Normal |
| GTEX-12WSF-0011-R10a-SM-5LZVH | 2.83778534 | 2.9273511 | 3.3833542 | Normal |
| GTEX-ZE9C-0011-R4a-SM-4WKH6 | 3.6605619 | 5.0601192 | 4.5539547 | Normal |
| GTEX-14BIN-0011-R11a-SM-5SI79 | 1.56662759 | 0.3742167 | 2.1384237 | Normal |
| GTEX-12ZZX-2926-SM-5GCOQ | 2.55430684 | 0.5270274 | 3.3391227 | Normal |
| GTEX-Y8DK-0011-R11A-SM-4SOK2 | 1.8918599 | 0.7581456 | 1.7743359 | Normal |
| GTEX-WZTO-2926-SM-3NM9I | 3.00962071 | 1.2951555 | 3.0166545 | Normal |
| GTEX-WWYW-0011-R5A-SM-3NB3E | 4.52921877 | 3.3915399 | 5.0663182 | Normal |
| GTEX-13QJC-0011-R2b-SM-5PNUN | 3.51534841 | 0.8410088 | 4.7322202 | Normal |
| GTEX-13SLX-0011-R10a-SM-5P9HV | 2.73743972 | 2.7708536 | 2.7004334 | Normal |
| GTEX-RVPV-0011-R11A-SM-2TF6F | 1.39026863 | 0.567781 | 1.8480177 | Normal |
| GTEX-12WSA-0011-R7b-SM-5GU5U | 3.80987034 | 1.1903 | 3.4595828 | Normal |
| GTEX-1269C-3126-SM-5EGI6 | 3.52352579 | 2.4217349 | 3.7547055 | Normal |
| GTEX-11WQK-0011-R10a-SM-5BC6R | 4.3660003 | 6.055891 | 4.520416 | Normal |
| GTEX-13OVJ-0011-R1b-SM-5L3I6 | 3.9671087 | 6.7943511 | 5.0521148 | Normal |
| GTEX-139TS-0011-R2b-SM-5J1MX | 2.81691148 | 3.950881 | 4.2430358 | Normal |
| GTEX-11OF3-0011-R6b-SM-57WBG | 2.28638947 | 1.3625969 | 2.8984105 | Normal |
| GTEX-13FHO-0011-R3b-SM-5K7YA | 2.9000912 | 4.1432898 | 4.3660003 | Normal |
| GTEX-RU72-0011-R5A-SM-2TF6U | 2.80335816 | 1.5738382 | 3.5751732 | Normal |
| GTEX-13OVL-0011-R1a-SM-5L3H5 | 2.93407815 | 2.872478 | 3.3701804 | Normal |
| GTEX-X4XX-0011-R11A-SM-46MWQ | 2.00345155 | 0.6593034 | 2.1580045 | Normal |
| GTEX-ZF28-0011-R5a-SM-4WWCZ | 4.35260894 | 1.1813771 | 4.3827867 | Normal |
| GTEX-NPJ7-0011-R9a-SM-2TC5R | 3.7802844 | 3.2870043 | 4.7689835 | Normal |
| GTEX-13OW6-0011-R10a-SM-5L3HE | 2.14571468 | 0.9817873 | 2.9100034 | Normal |
| GTEX-T6MN-0011-R1A-SM-32QOY | 1.89171169 | 2.1112187 | 2.0990673 | Normal |
| GTEX-RVPU-0011-R7A-SM-2XCAB | 3.7360222 | 2.0976535 | 4.1541147 | Normal |
| GTEX-11GSP-0011-R7b-SM-57WC3 | 2.80250824 | 3.4969614 | 3.1198733 | Normal |
| GTEX-12ZZX-0011-R6a-SM-5HL8L | 2.5376168 | 0.836395 | 2.9407483 | Normal |
| GTEX-131XW-0011-R10a-SM-5DUVA | 3.2047239 | 1.9509563 | 4.0628746 | Normal |
| GTEX-UTHO-0011-R6A-SM-3GIJW | 2.4238986 | 1.7924342 | 3.1723815 | Normal |
| GTEX-13VXU-3026-SM-5LU3K | 1.98177143 | 0.6199077 | 1.8398656 | Normal |
| GTEX-T2IS-0011-R2A-SM-32QPF | 2.63902084 | 3.9273731 | 4.0148202 | Normal |
| GTEX-131XH-2926-SM-5LZU4 | 1.43079213 | 0.4514819 | 1.6688869 | Normal |
| GTEX-139UC-0011-R5b-SM-5K7VY | 2.76609744 | 2.455855 | 3.347511 | Normal |
| GTEX-QDT8-0011-R5A-SM-32PKN | 2.6192082 | 4.3491994 | 4.0894595 | Normal |
| GTEX-13G51-0011-R10b-SM-5LZYF | 2.09327197 | 1.2815955 | 2.5363529 | Normal |
| GTEX-11DXW-1126-SM-5H12Q | 2.64737217 | 1.1881481 | 3.3038703 | Normal |
| GTEX-145LS-0011-R2a-SM-5PNZI | 3.38578156 | 1.9298729 | 3.9773358 | Normal |
| GTEX-WL46-0011-R10A-SM-3MJFQ | 2.15564971 | 1.4750889 | 2.429768 | Normal |
| GTEX-12WS9-0011-R6a-SM-5P9EW | 4.06781852 | 2.2593916 | 4.8818548 | Normal |
| GTEX-147GR-0011-R7a-SM-5S2RP | 3.33131022 | 1.6009427 | 3.7346561 | Normal |
| GTEX-13PL6-3126-SM-5LUAR | 3.12071567 | 1.7798447 | 3.6767295 | Normal |
| GTEX-12WSH-0011-R11a-SM-5LZVM | 1.39087807 | 0.897511 | 2.1523362 | Normal |
| GTEX-13SLX-0011-R2b-SM-5S2VP | 2.55430684 | 2.7039 | 3.3367349 | Normal |
| GTEX-WHSE-0011-R4A-SM-3P5ZN | 3.33159653 | 7.9046751 | 5.1650331 | Normal |
| GTEX-12ZZW-0011-R3a-SM-5DUWW | 3.07304222 | 1.1395156 | 4.0135236 | Normal |
| GTEX-13X6K-0011-R2b-SM-5P9K3 | 4.64216856 | 2.9749037 | 5.297722 | Normal |
| GTEX-13X6I-0011-R10a-SM-5PNWI | 3.43621463 | 1.3539202 | 3.6064724 | Normal |
| GTEX-14BIM-0011-R11b-SM-5S2RN | 1.92719594 | 0.9694509 | 2.0908379 | Normal |
| GTEX-R55F-0011-R8A-SM-2TF4F | 3.4759423 | 2.524319 | 4.0923721 | Normal |
| GTEX-11UD1-0011-R2b-SM-5BC6O | 3.2410412 | 2.2641237 | 4.2841118 | Normal |
| GTEX-147F4-0011-R6b-SM-5S2UP | 3.6315646 | 6.8584546 | 4.8104213 | Normal |
| GTEX-X585-0011-R11B-SM-46MUZ | 3.4615721 | 2.2223548 | 3.3370467 | Normal |
| GTEX-13SLX-3126-SM-5S2Q5 | 2.50413742 | 2.6185029 | 2.8223208 | Normal |
| GTEX-11NV4-0011-R6a-SM-57WD5 | 2.41090177 | 1.2917705 | 2.9471125 | Normal |
| GTEX-13CZV-0011-R7b-SM-5LZXH | 3.545524 | 3.0194703 | 4.7184494 | Normal |
| GTEX-145LU-0011-R10a-SM-5PNWJ | 2.71780704 | 0.8516215 | 3.0044638 | Normal |
| GTEX-ZAB4-0011-R2a-SM-4RGNP | 2.61410411 | 1.7963136 | 3.3930682 | Normal |
| GTEX-11O72-2926-SM-5BC4V | 3.80173782 | 5.5739353 | 4.6639564 | Normal |
| GTEX-12WSH-0011-R10a-SM-5LZUS | 2.41677615 | 1.1713148 | 3.8351931 | Normal |
| GTEX-13FXS-0011-R6b-SM-5L3F7 | 2.3624699 | 1.3157279 | 2.8556591 | Normal |
| GTEX-1313W-0011-R4b-SM-5KLZV | 2.30673612 | 2.3286931 | 3.4689952 | Normal |
| GTEX-Y8DK-0011-R6A-SM-4V6EG | 2.64164749 | 0.8968821 | 2.75352 | Normal |
| GTEX-R55E-0011-R7A-SM-2TC5Z | 2.28048963 | 1.9054423 | 3.1613441 | Normal |
| GTEX-131YS-0011-R1a-SM-5DUXC | 4.05961108 | 5.7754982 | 4.8233834 | Normal |
| GTEX-13OW6-0011-R7b-SM-5L3H4 | 2.92619465 | 1.9864756 | 3.3938073 | Normal |
| GTEX-ZVT3-3026-SM-5E43N | 2.65513742 | 1.1805414 | 3.4447474 | Normal |
| GTEX-13X6J-0011-R10b-SM-5PNWA | 2.20461344 | 1.2715227 | 2.3824608 | Normal |
| GTEX-11GSP-3126-SM-5A5LL | 1.76978984 | 1.7450308 | 2.5088223 | Normal |
| GTEX-13PL6-0011-R5b-SM-5O9BI | 3.73330651 | 2.8596803 | 4.4318494 | Normal |
| GTEX-11WQC-0011-R8b-SM-57WCH | 2.42359649 | 1.9715782 | 3.3361277 | Normal |
| GTEX-13OVJ-0011-R4b-SM-5P9H5 | 4.47869185 | 6.5199135 | 5.1839165 | Normal |
| GTEX-13RTJ-0011-R2a-SM-5PNW9 | 3.67473408 | 1.5885486 | 4.3610915 | Normal |
| GTEX-13FLW-0011-R7b-SM-5L3EZ | 2.59788341 | 1.3808285 | 2.9253872 | Normal |
| GTEX-XMD1-0011-R1A-SM-4AT4C | 4.08468169 | 1.4883849 | 4.548089 | Normal |
| GTEX-S7PM-0011-R6A-SM-3NM8F | 3.57563471 | 2.1317754 | 4.528986 | Normal |
| GTEX-139UW-0011-R11a-SM-5IJGN | 2.15058328 | 3.8415464 | 3.9998669 | Normal |
| GTEX-1313W-3026-SM-5LZUZ | 1.62293733 | 0.8865633 | 1.5079115 | Normal |
| GTEX-139UC-3226-SM-5N9EZ | 2.09589277 | 0.4796646 | 2.68048 | Normal |
| GTEX-WL46-0011-R9A-SM-3MJFP | 2.49986588 | 1.9331592 | 3.22291 | Normal |
| GTEX-11ZU8-0011-R4a-SM-5BC6Y | 2.9381784 | 2.4258428 | 3.8145785 | Normal |
| GTEX-NL3H-0011-R3a-SM-2I3GL | 2.53855935 | 1.8420775 | 3.0944093 | Normal |
| GTEX-N7MT-0011-R1a-SM-5SI7S | 3.09599575 | 2.3442206 | 3.889333 | Normal |
| GTEX-13N1W-0011-R6a-SM-5MR43 | 3.35770535 | 7.7533706 | 4.6998541 | Normal |
| GTEX-13FLV-0011-R3b-SM-5LZYP | 2.79598263 | 1.8168371 | 3.4411864 | Normal |
| GTEX-T5JC-0011-R6A-SM-5SI89 | 3.62947227 | 5.6767502 | 4.3500683 | Normal |
| GTEX-NPJ8-0011-R8a-SM-2HMLG | 4.49612753 | 1.807836 | 4.7439587 | Normal |
| GTEX-1445S-0011-R10a-SM-5PNWB | 4.02512046 | 4.5891569 | 5.6262694 | Normal |
| GTEX-T2IS-2926-SM-32QPO | 1.97356156 | 1.1716669 | 2.0515458 | Normal |
| GTEX-131XW-0011-R11a-SM-5DUV9 | 1.8936271 | 0.1725512 | 2.9114126 | Normal |
| GTEX-QVJO-1325-SM-2S1QX | 1.94467527 | 0.5954942 | 2.2708021 | Normal |
| GTEX-XMD1-0011-R3B-SM-4AT5R | 4.23851908 | 1.1807306 | 4.6230239 | Normal |
| GTEX-ZE7O-0011-R10a-SM-57WAZ | 2.23912932 | 2.5516687 | 3.3054965 | Normal |
| GTEX-13G51-3026-SM-5IJB8 | 2.14385473 | 1.8051783 | 2.5921125 | Normal |
| GTEX-13FHP-0011-R11b-SM-5LZXP | 1.59489427 | 0.3759004 | 1.658277 | Normal |
| GTEX-WWYW-0011-R11A-SM-3NB38 | 2.60501456 | 1.4482665 | 3.1192705 | Normal |
| GTEX-14ABY-0011-R5b-SM-5SI7B | 2.3951948 | 2.2859733 | 3.1762275 | Normal |
| GTEX-ZXG5-0011-R6a-SM-57WBZ | 2.30139926 | 0.6933664 | 3.1274428 | Normal |
| GTEX-X4EP-3026-SM-3P5YP | 4.63568424 | 3.8939863 | 3.8969842 | Normal |
| GTEX-13OW7-0011-R8b-SM-5L3HR | 1.97425068 | 2.0070674 | 2.7838434 | Normal |
| GTEX-13RTJ-0011-R8b-SM-5O9DL | 2.5634339 | 1.2073813 | 3.2775343 | Normal |
| GTEX-12ZZZ-0011-R4b-SM-5DUV7 | 3.3703169 | 3.9259465 | 3.832547 | Normal |
| GTEX-13CZV-0011-R3a-SM-5KM27 | 2.7277306 | 2.2050013 | 3.8098703 | Normal |
| GTEX-N7MS-0011-R11A-SM-2HMJS | 1.21704661 | 1.0499612 | 1.2349229 | Normal |
| GTEX-12WSE-0011-R10b-SM-5P9JV | 2.35089387 | 0.5783672 | 2.1753594 | Normal |
| GTEX-13SLW-0011-R5b-SM-5SI6Z | 3.74561772 | 1.6271898 | 4.463004 | Normal |
| GTEX-14753-0011-R10b-SM-5S2WE | 2.94074825 | 1.3263252 | 4.3161436 | Normal |
| GTEX-13OW5-0011-R1b-SM-5L3GE | 5.79516606 | 6.5764496 | 5.4593047 | Normal |
| GTEX-14ABY-0011-R4b-SM-5S2VN | 3.01960651 | 2.1643158 | 3.9328724 | Normal |
| GTEX-NL3H-0011-R10A-SM-2I3E9 | 2.38206203 | 0.5886277 | 3.3219466 | Normal |
| GTEX-145LU-2626-SM-5LU9Z | 1.54399822 | 0.1732794 | 1.9983593 | Normal |
| GTEX-WHSE-0011-R6A-SM-3P5ZP | 3.61344062 | 6.5784549 | 4.7493087 | Normal |
| GTEX-NPJ8-0011-R5a-SM-2HMJY | 5.59489456 | 2.1760214 | 5.0997937 | Normal |
| GTEX-13OW6-0011-R8a-SM-5L3GF | 1.97792438 | 1.8419291 | 2.9950238 | Normal |
| GTEX-ZDXO-0011-R7a-SM-4WWCU | 3.34259404 | 2.3970613 | 3.7153014 | Normal |
| GTEX-NPJ8-0011-R11A-SM-2YUMS | 3.23566524 | 0.1027233 | 3.1518579 | Normal |
| GTEX-139TS-0011-R6a-SM-5J2OB | 3.95507476 | 6.1051823 | 5.0104776 | Normal |
| GTEX-11NUK-2926-SM-5A5MD | 1.88481041 | 1.6575979 | 3.489801 | Normal |
| GTEX-131XW-0011-R9a-SM-5N9EO | 2.68144747 | 1.9807062 | 3.7887731 | Normal |
| GTEX-145LU-0011-R1a-SM-5Q5CE | 2.0006957 | 2.0402524 | 3.1247071 | Normal |
| GTEX-145LS-0011-R4b-SM-5S2UT | 3.11768827 | 2.5536227 | 3.8171436 | Normal |
| GTEX-ZF28-0011-R10a-SM-4WWEH | 3.25768176 | 0.7257466 | 3.6651201 | Normal |
| GTEX-144GO-0011-R1a-SM-5PNWO | 2.8445277 | 2.582797 | 4.424607 | Normal |
| GTEX-NPJ8-0011-R7a-SM-2HMJV | 5.26318961 | 4.1169506 | 5.0924783 | Normal |
| GTEX-X4EP-0011-R10B-SM-4QASJ | 6.16036577 | 5.8851569 | 4.6797285 | Normal |
| GTEX-NPJ8-0011-R9a-SM-2YUN5 | 4.24325005 | 2.3588068 | 4.8935338 | Normal |
| GTEX-145MH-0011-R11a-SM-5P9HQ | 1.41978164 | 0.4648972 | 1.5116299 | Normal |
| GTEX-QVUS-0011-R6A-SM-3GACX | 4.27521054 | 1.2735134 | 4.5122602 | Normal |
| GTEX-WVLH-0011-R7A-SM-3MJFB | 3.26789471 | 2.1974661 | 4.3148986 | Normal |
| GTEX-144GL-0011-R4a-SM-5PNUI | 2.58543185 | 2.0034516 | 3.7427406 | Normal |
| GTEX-1445S-0011-R3a-SM-5P9HY | 3.90204431 | 5.1579995 | 5.5930316 | Normal |
| GTEX-14ABY-0011-R11a-SM-5SI7C | 1.70260596 | 0.3639057 | 1.4734538 | Normal |
| GTEX-13OW6-0011-R4a-SM-5O9DX | 2.45763256 | 2.4107651 | 3.6171773 | Normal |
| GTEX-ZVT3-0011-R8b-SM-57WDJ | 2.69046467 | 2.8681465 | 3.5746915 | Normal |
| GTEX-QVJO-0011-R4A-SM-2S1QL | 2.8602165 | 1.8259225 | 4.0310944 | Normal |
| GTEX-X4EP-0011-R5A-SM-3P628 | 6.42031852 | 7.1863037 | 5.1788682 | Normal |
| GTEX-WVLH-0011-R2A-SM-3MJFJ | 2.35736918 | 0.9488443 | 2.6947504 | Normal |
| GTEX-13O3O-0011-R4b-SM-5KM3F | 3.00303745 | 5.0911812 | 4.1430959 | Normal |
| GTEX-13OVJ-0011-R11a-SM-5P9H8 | 2.29745793 | 3.6212618 | 3.1945173 | Normal |
| GTEX-X4XX-0011-R7A-SM-46MWR | 3.865527 | 2.036002 | 3.968749 | Normal |
| GTEX-12WSB-0011-R5b-SM-5GU67 | 4.76299699 | 3.0654637 | 4.6150503 | Normal |
| GTEX-11ONC-0011-R9b-SM-5NQ8J | 2.4427955 | 3.0694509 | 3.8729711 | Normal |
| GTEX-13JUV-0011-R10b-SM-5LZXR | 3.20268112 | 2.2870738 | 3.8252224 | Normal |
| GTEX-13OW6-0011-R1a-SM-5L3HQ | 2.00358937 | 1.8947384 | 2.874862 | Normal |
| GTEX-13OW5-3126-SM-5MR3Y | 3.46990109 | 3.2006519 | 4.2372778 | Normal |
| GTEX-12WSE-2926-SM-5RQJ9 | 1.44000266 | 0.42534 | 1.3833634 | Normal |
| GTEX-TSE9-2926-SM-5CHQO | 0.01943061 | 0.0194306 | 0.0194306 | Normal |
| GTEX-13S7M-3026-SM-5S2QQ | 1.3894551 | 0.28992 | 1.9094156 | Normal |
| GTEX-13JUV-3026-SM-5K7WX | 2.88273371 | 2.4966276 | 3.34824 | Normal |
| GTEX-12ZZX-0011-R11a-SM-5DUVJ | 1.76701511 | 0.827213 | 2.137466 | Normal |
| GTEX-13NYC-2826-SM-5K7WR | 4.27272017 | 6.0763116 | 4.7531564 | Normal |
| GTEX-13O3O-0011-R5b-SM-5KM44 | 2.6118989 | 2.970927 | 3.5469545 | Normal |
| GTEX-145LS-0011-R5a-SM-5SI65 | 2.94738633 | 1.0802638 | 3.2233491 | Normal |
| GTEX-13JVG-0011-R10b-SM-5KM2P | 3.72721646 | 3.5808393 | 4.2911466 | Normal |
| GTEX-131XH-0011-R8a-SM-5EGLL | 2.76469849 | 2.4138935 | 3.4414951 | Normal |
| GTEX-139TT-0011-R5b-SM-5K7W8 | 2.58515519 | 1.4722412 | 2.9622649 | Normal |
| GTEX-1445S-3026-SM-5O9BR | 3.79825641 | 3.5331439 | 5.512429 | Normal |
| GTEX-RU72-3026-SM-5SI7Y | 2.46011773 | 1.1342506 | 3.8838279 | Normal |
| GTEX-N7MS-2425-SM-26GMD | 1.30011983 | 0.8713999 | 2.7004334 | Normal |
| GTEX-N7MT-1226-SM-26GMT | 1.79618366 | 0.0818547 | 2.7573143 | Normal |
| GTEX-N7MT-1226-SM-26GMC | 2.13093554 | 0 | 2.6368003 | Normal |
| GTEX-ZUA1-2926-SM-59HL3 | 1.68824804 | 0.6841097 | 1.7564534 | Normal |
| GTEX-12WSM-2126-SM-5GCOC | 2.07045808 | 0.2947881 | 2.6770383 | Normal |
| GTEX-1445S-0011-R4a-SM-5PNWN | 3.31156926 | 4.1173471 | 5.239252 | Normal |
| GTEX-ZE9C-0011-R7a-SM-4WWCX | 4.17386723 | 5.0549044 | 4.7838905 | Normal |
| GTEX-1313W-0011-R6a-SM-5K7XO | 1.84737525 | 1.2747952 | 2.4043227 | Normal |
| GTEX-13RTL-0011-R9a-SM-5Q5BR | 3.239005 | 6.381337 | 4.962991 | Normal |
| GTEX-13X6J-0011-R7b-SM-5PNUC | 2.48851001 | 1.7262796 | 2.6153135 | Normal |
| GTEX-145LS-0011-R1b-SM-5PNUP | 3.06789519 | 1.9819142 | 3.8526373 | Normal |
| GTEX-13NYB-0011-R5a-SM-5MR45 | 2.88757716 | 1.9258868 | 3.3368908 | Normal |
| GTEX-QVUS-0011-R10A-SM-3GIK3 | 4.21953677 | 2.3466156 | 4.381722 | Normal |
| GTEX-Q2AG-2926-SM-2HMJ3 | 2.651805 | 1.07954 | 3.380924 | Normal |
| GTEX-13X6I-2926-SM-5Q5C3 | 2.75097304 | 1.1454852 | 3.2063135 | Normal |
| GTEX-13FTY-0011-R9b-SM-5KM2U | 2.3575916 | 3.4793346 | 3.2353809 | Normal |
| GTEX-11ZVC-0011-R3a-SM-5BC6V | 2.87742082 | 1.9351564 | 3.7466293 | Normal |
| GTEX-13NYS-3126-SM-5KLYV | 4.69985408 | 2.9785864 | 4.762997 | Normal |
| GTEX-13S7M-0011-R10b-SM-5PNZB | 2.15447887 | 0.8326123 | 2.9732123 | Normal |
| GTEX-13CZV-0011-R4b-SM-5J2NY | 2.77587761 | 3.1590061 | 4.63896 | Normal |
| GTEX-131YS-0011-R7a-SM-5IJDA | 2.60092615 | 1.0976529 | 2.8118175 | Normal |
| GTEX-1477Z-0011-R10b-SM-5S2RF | 3.0420329 | 1.0069604 | 3.8818516 | Normal |
| GTEX-13NYS-0011-R4b-SM-5MR3O | 5.97618658 | 4.9013447 | 6.0090607 | Normal |
| GTEX-X4XX-2926-SM-3NMB1 | 2.02248171 | 0.7167422 | 2.3931061 | Normal |
| GTEX-NPJ8-1526-SM-2D7VU | 4.42853021 | 3.2038767 | 4.4922633 | Normal |
| GTEX-WVLH-0011-R6A-SM-3MJFZ | 2.80004391 | 1.1985393 | 3.202127 | Normal |
| GTEX-14A5I-2926-SM-5Q5CQ | 2.85797185 | 0.9714944 | 3.9086683 | Normal |
| GTEX-13PLJ-0011-R11b-SM-5O9BS | 1.70917739 | 0.5014114 | 2.3755666 | Normal |
| GTEX-11TTK-2826-SM-5GU5K | 3.36205914 | 1.7678443 | 4.186353 | Normal |
| GTEX-13SLX-0011-R9b-SM-5SI6X | 3.21724238 | 4.584426 | 3.6363296 | Normal |
| GTEX-XMD1-2926-SM-4AT42 | 7.10964979 | 3.1641248 | 5.9774148 | Normal |
| GTEX-X585-0011-R5A-SM-46MVI | 3.95415219 | 2.6985202 | 3.9596292 | Normal |
| GTEX-1399T-0011-R8b-SM-5IJD8 | 2.50940561 | 3.564399 | 3.1792663 | Normal |
| GTEX-13O1R-0011-R9b-SM-5KM33 | 4.0378874 | 6.3333466 | 4.1237148 | Normal |
| GTEX-13JVG-0011-R4a-SM-5MR4C | 3.273513 | 3.8614857 | 4.5825422 | Normal |
| GTEX-X4EP-0011-R11B-SM-4QASK | 3.12101713 | 4.0514997 | 3.0481854 | Normal |
| GTEX-YJ89-0011-R5a-SM-4V6GM | 3.18914088 | 1.6203422 | 3.4728512 | Normal |
| GTEX-14BIM-0011-R4b-SM-5S2RK | 4.06152931 | 4.3739342 | 4.5115439 | Normal |
| GTEX-13SLW-0011-R8b-SM-5SI64 | 2.49135434 | 2.3302206 | 3.7989629 | Normal |
| GTEX-13QIC-0011-R8b-SM-5O9CV | 2.11147228 | 2.1598432 | 2.9890547 | Normal |
| GTEX-N7MS-0011-R8a-SM-2YUMK | 2.67042153 | 2.3524935 | 3.7903234 | Normal |
| GTEX-13NYB-0011-R10a-SM-5KM43 | 2.42907425 | 1.3681794 | 2.6150275 | Normal |
| GTEX-14BIN-3326-SM-5Q5E6 | 1.66022825 | 0.405226 | 1.9128595 | Normal |
| GTEX-NPJ8-0011-R10A-SM-2YUMO | 4.54257622 | 2.4879467 | 4.4139415 | Normal |
| GTEX-1313W-0011-R5b-SM-5L3EP | 2.42045537 | 1.9372476 | 2.5384168 | Normal |
| GTEX-QVJO-0011-R10A-SM-2S1QJ | 2.71906495 | 1.0488734 | 3.3709194 | Normal |
| GTEX-Q2AG-0011-R4A-SM-2HMKA | 1.86689483 | 0.8278373 | 3.3443813 | Normal |
| GTEX-RVPU-0011-R4A-SM-2TF6X | 3.577247 | 2.288587 | 4.458119 | Normal |
| GTEX-WHSE-0011-R7A-SM-3P5YZ | 3.23344215 | 6.0749954 | 3.7819857 | Normal |
| GTEX-P44G-0011-R10A-SM-2I3FF | 3.11071931 | 5.8417031 | 4.3271582 | Normal |
| GTEX-YFC4-0011-R1a-SM-4V6EH | 2.55166866 | 3.5105071 | 3.6118269 | Normal |
| GTEX-131XH-0011-R10a-SM-5EGLJ | 3.06115146 | 1.3431195 | 2.6755424 | Normal |
| GTEX-13N2G-0011-R2a-SM-5MR4Q | 3.15172724 | 1.4736845 | 4.0691383 | Normal |
| GTEX-1128S-2726-SM-5H12C | 2.85899614 | 0.717715 | 4.0729967 | Normal |
| GTEX-11ZVC-0011-R4a-SM-5BC6Z | 3.33343568 | 2.5569543 | 4.1798119 | Normal |
| GTEX-11GSO-0011-R3b-SM-57WB2 | 2.32869314 | 0.7825196 | 2.9367421 | Normal |
| GTEX-WVLH-2926-SM-3MJG5 | 1.94812769 | 0.491435 | 2.0885114 | Normal |
| GTEX-RVPU-0011-R8A-SM-5SI8I | 2.9472435 | 1.686438 | 3.8408495 | Normal |
| GTEX-12ZZX-0011-R3b-SM-5EGLH | 2.58362178 | 1.3439312 | 3.4848114 | Normal |
| GTEX-1313W-0011-R2a-SM-5EGLF | 2.22383326 | 2.1687155 | 2.9771859 | Normal |
| GTEX-13O3Q-0011-R1b-SM-5KM3L | 2.36535852 | 1.5893511 | 3.1156175 | Normal |
| GTEX-XLM4-3026-SM-4AT6L | 2.61203711 | 1.1696583 | 3.6337567 | Normal |
| GTEX-12WSI-0011-R11b-SM-5P9EZ | 1.35173195 | 0.6102241 | 1.1954612 | Normal |
| GTEX-N7MS-0011-R4a-SM-2HMKW | 1.87967483 | 1.8833713 | 3.1157552 | Normal |
| GTEX-131YS-3026-SM-5EGHY | 1.71166368 | 0.302435 | 2.1742919 | Normal |
| GTEX-13JUV-0011-R1a-SM-5LZY4 | 4.42569405 | 4.9741897 | 3.9897317 | Normal |
| GTEX-1399T-0011-R2b-SM-5DUXM | 3.4307978 | 2.9869389 | 3.9121395 | Normal |
| GTEX-XOTO-0011-R7B-SM-4B64R | 2.50142316 | 1.8556973 | 3.1608934 | Normal |
| GTEX-12WSD-0011-R10b-SM-5GU79 | 2.6891766 | 1.6224427 | 2.927494 | Normal |
| GTEX-N7MS-0011-R6a-SM-2HMJ4 | 1.68721322 | 1.232804 | 2.3722485 | Normal |
| GTEX-13S7M-0011-R5b-SM-5P9HS | 2.87855936 | 0.7210368 | 3.3727681 | Normal |
| GTEX-12126-0011-R10b-SM-5BC6T | 1.79180203 | 1.2165501 | 2.9866676 | Normal |
| GTEX-QVJO-0011-R1A-SM-2S1QI | 3.28020894 | 2.1157355 | 4.2754209 | Normal |
| GTEX-XMD1-0011-R2B-SM-4AT5N | 4.42765675 | 2.2405247 | 4.9475505 | Normal |
| GTEX-RU72-0011-R11A-SM-2TF6J | 2.38631195 | 1.295686 | 2.4006556 | Normal |
| GTEX-11UD1-0011-R3a-SM-5BC6S | 2.62087903 | 1.4832254 | 3.8367892 | Normal |
| GTEX-XOTO-0011-R1B-SM-4B65C | 2.45370909 | 2.0628624 | 3.223041 | Normal |
| GTEX-13O3O-0011-R1b-SM-5KM2K | 2.02863294 | 4.6240117 | 2.9936105 | Normal |
| GTEX-14753-0011-R5a-SM-5S2VW | 3.5192743 | 2.6187762 | 4.995449 | Normal |
| GTEX-12WS9-2826-SM-5FQU9 | 3.62488315 | 0.6305499 | 5.1407442 | Normal |
| GTEX-145LS-0011-R3a-SM-5S2VJ | 2.83959829 | 1.1659745 | 3.5501351 | Normal |
| GTEX-R55E-0011-R8A-SM-2TC66 | 1.54817979 | 2.0132105 | 3.1189808 | Normal |
| GTEX-14BIM-0011-R5b-SM-5S2RM | 3.89114357 | 0.9032239 | 3.574029 | Normal |
| GTEX-ZF28-0011-R7a-SM-4WKF5 | 4.57829852 | 1.8430709 | 4.6589991 | Normal |
| GTEX-RVPU-0011-R8A-SM-2TF74 | 3.3464688 | 2.6109314 | 3.8708438 | Normal |
| GTEX-12ZZY-0011-R5a-SM-5EGL9 | 2.64695183 | 1.3986699 | 3.4228329 | Normal |
| GTEX-RNOR-0011-R9A-SM-2TF52 | 3.6707894 | 3.9163013 | 4.0251205 | Normal |
| GTEX-NPJ8-1526-SM-26GMH | 3.75100636 | 3.2313765 | 4.4130725 | Normal |
| GTEX-13VXU-0011-R6a-SM-5SI71 | 2.02729369 | 2.0725977 | 2.8542578 | Normal |
| GTEX-NPJ8-1526-SM-26GMY | 3.66843658 | 2.99444 | 4.1481213 | Normal |
| GTEX-12WSF-0011-R3a-SM-5DUW7 | 3.11987334 | 3.9806815 | 4.1272282 | Normal |
| GTEX-12WSD-0011-R7b-SM-5PNWE | 2.32951732 | 0.5510168 | 3.3578532 | Normal |
| GTEX-14BIM-0011-R3b-SM-5S2RL | 3.02189686 | 2.3540171 | 3.1085647 | Normal |
| GTEX-13G51-0011-R1a-SM-5LZYR | 3.28461331 | 3.3839566 | 3.743941 | Normal |
| GTEX-13FLW-0011-R1b-SM-5LZX2 | 1.94788232 | 1.4132597 | 2.4789491 | Normal |
| GTEX-13OW6-0011-R11a-SM-5L3H2 | 1.2934868 | 0.5292147 | 1.4824716 | Normal |
| GTEX-NPJ8-0011-R6a-SM-2HMKB | 4.78202693 | 0.6917388 | 4.3494104 | Normal |
| GTEX-11ZVC-0011-R1b-SM-5BC6M | 3.44816964 | 2.5414253 | 4.110198 | Normal |
| GTEX-WWYW-3026-SM-3NB36 | 2.86034591 | 1.4734538 | 3.8255771 | Normal |
| GTEX-QMR6-0011-R8A-SM-32PKJ | 2.6806161 | 1.5905559 | 3.205721 | Normal |
| GTEX-13RTJ-0011-R9a-SM-5Q5AY | 2.75193843 | 2.0615544 | 3.6136073 | Normal |
| GTEX-13NYS-0011-R1b-SM-5MR55 | 5.05149797 | 5.2015181 | 5.554388 | Normal |
| GTEX-OHPN-0011-R8A-SM-33HBT | 4.79434105 | 4.0803943 | 4.3178208 | Normal |
| GTEX-13O3Q-0011-R2b-SM-5KM3K | 2.84704968 | 1.4296325 | 3.5219494 | Normal |
| GTEX-13X6K-0011-R6a-SM-5P9K6 | 3.16398743 | 1.7555009 | 3.7732639 | Normal |
| GTEX-Y8DK-0011-R1A-SM-4RGLO | 1.99733284 | 1.366807 | 3.2063135 | Normal |
| GTEX-131YS-0011-R11b-SM-5EGLM | 1.6120354 | 0.799463 | 1.6789427 | Normal |
| GTEX-X4XX-3026-SM-3NMB2 | 2.64178207 | 1.9521527 | 3.4133048 | Normal |
| GTEX-11ONC-3026-SM-5985W | 1.88125557 | 0.7998514 | 2.6858986 | Normal |
| GTEX-13NZA-0011-R9b-SM-5MR5I | 4.00754284 | 5.6165152 | 4.0691383 | Normal |
| GTEX-ZVZQ-0011-R3a-SM-57WC7 | 2.35528049 | 1.7796002 | 3.2173799 | Normal |
| GTEX-14BIN-0011-R9a-SM-5SI76 | 2.94779139 | 2.6130074 | 3.4555485 | Normal |
| GTEX-139TT-0011-R7b-SM-5J2MN | 3.12116827 | 1.4574977 | 3.138858 | Normal |
| GTEX-11OF3-0011-R11a-SM-57WCR | 1.47447309 | 0.340498 | 1.277864 | Normal |
| GTEX-145MF-2726-SM-5O995 | 2.40388546 | 1.3691978 | 2.9938951 | Normal |
| GTEX-13G51-0011-R11b-SM-5J2NE | 1.90793387 | 0.6117964 | 1.7754904 | Normal |
| GTEX-13O3Q-0011-R5b-SM-5P9H4 | 2.40628492 | 0.7111867 | 2.8868482 | Normal |
| GTEX-X4XY-0011-R10B-SM-46MWS | 2.4752018 | 3.6150478 | 3.8140656 | Normal |
| GTEX-1313W-3126-SM-5LZUI | 2.26606999 | 1.4286523 | 2.7004334 | Normal |
| GTEX-13FXS-0011-R11a-SM-5L3DM | 1.74477733 | 0.6589466 | 1.8537436 | Normal |
| GTEX-13FHO-0011-R5b-SM-5LZZ3 | 2.92886806 | 3.7795976 | 4.4307348 | Normal |
| GTEX-1445S-0011-R7b-SM-5PNUG | 4.33952864 | 3.6628381 | 5.3157556 | Normal |
| GTEX-RU72-0011-R6A-SM-2TF71 | 2.89350195 | 1.8648004 | 3.367638 | Normal |
| GTEX-UTHO-0011-R10A-SM-3GIJQ | 2.391158 | 2.268347 | 2.788111 | Normal |
| GTEX-145MH-0011-R8a-SM-5PNUL | 2.5422738 | 0.8634531 | 3.2365355 | Normal |
| GTEX-NPJ8-0011-R4a-SM-2HML3 | 4.98162908 | 3.1211683 | 5.3199287 | Normal |
| GTEX-ZYY3-3126-SM-5SI9L | 5.19867665 | 2.4869736 | 4.7505817 | Normal |
| GTEX-139TT-0011-R11b-SM-5LZV1 | 1.52852593 | 0.2534971 | 1.6477376 | Normal |
| GTEX-145LU-0011-R11b-SM-5P9JX | 1.0950487 | 0.0517435 | 1.6266006 | Normal |
| GTEX-13OW7-2926-SM-5MR3Z | 1.76677661 | 0.5541925 | 2.4119907 | Normal |
| GTEX-12WSA-2926-SM-5EQ4D | 4.14348364 | 2.1137139 | 4.3879804 | Normal |
| GTEX-12WSD-0011-R4b-SM-5LZUA | 2.70350942 | 2.337589 | 3.5706004 | Normal |
| GTEX-13S7M-0011-R9a-SM-5O9DW | 3.71530139 | 3.3359758 | 4.1009901 | Normal |
| GTEX-14ASI-0011-R6a-SM-5S2UZ | 2.52381974 | 1.4753138 | 2.8223208 | Normal |
| GTEX-13OVL-0011-R5b-SM-5L3FU | 2.87302654 | 1.6415995 | 3.3819793 | Normal |
| GTEX-1313W-0011-R7b-SM-5DUWL | 2.36290482 | 1.7939516 | 2.578789 | Normal |
| GTEX-144GL-0011-R6a-SM-5P9HW | 1.91526361 | 0.6373044 | 2.9467068 | Normal |
| GTEX-R55F-1326-SM-2TF5F | 4.402459 | 2.839046 | 4.475076 | Normal |
| GTEX-1477Z-0011-R3b-SM-5PNX9 | 3.20602211 | 1.1287387 | 4.4351194 | Normal |
| GTEX-13X6I-0011-R8a-SM-5PNZF | 4.19787978 | 3.7862002 | 4.5384179 | Normal |
| GTEX-12ZZX-2826-SM-5BC6K | 1.4825978 | 0.6866353 | 2.114245 | Normal |
| GTEX-13X6J-0011-R5b-SM-5PNWT | 2.32555979 | 1.4116277 | 2.8800607 | Normal |
| GTEX-NPJ8-0011-R1a-SM-5SI8B | 4.79093428 | 2.0503004 | 4.974493 | Normal |
| GTEX-XLM4-0011-R8A-SM-4AT44 | 3.41803413 | 3.5034202 | 4.158772 | Normal |
| GTEX-139UW-0011-R9b-SM-5LZYT | 2.39650932 | 3.5603828 | 3.7258576 | Normal |
| GTEX-13RTJ-0011-R1a-SM-5O9D9 | 3.50434668 | 2.0028662 | 4.3561125 | Normal |
| GTEX-ZUA1-0011-R5b-SM-51MTG | 2.6185029 | 1.7947347 | 2.8809068 | Normal |
| GTEX-11ZUS-2926-SM-5FQSL | 2.00706744 | 0.9096959 | 2.8563259 | Normal |
| GTEX-117XS-3026-SM-5N9CA | 2.06339159 | 1.5322465 | 3.2391524 | Normal |
| GTEX-13NYS-0011-R5a-SM-5MR4D | 5.64798567 | 3.6358312 | 5.2733213 | Normal |
| GTEX-13OVJ-2726-SM-5IJG6 | 2.68032498 | 3.9406925 | 3.9698531 | Normal |
| GTEX-Z93S-0011-R5b-SM-4RGNI | 2.28005878 | 0.517319 | 2.2275242 | Normal |
| GTEX-QDT8-3026-SM-32PKB | 1.28861527 | 0.2801217 | 2.3708956 | Normal |
| GTEX-NPJ8-0011-R3a-SM-2HMIW | 4.90495912 | 1.3250832 | 4.7966735 | Normal |
| GTEX-13OVH-0011-R4a-SM-5KM3W | 2.91366323 | 1.4838339 | 4.3351257 | Normal |
| GTEX-11ONC-2926-SM-5P9JM | 3.54615202 | 2.1222383 | 3.7479905 | Normal |
| GTEX-WWYW-0011-R7A-SM-3NB3H | 4.61652102 | 4.7436928 | 5.4507488 | Normal |
| GTEX-RNOR-2226-SM-2TF5O | 2.27773905 | 0.9496333 | 2.4484627 | Normal |
| GTEX-WHSE-0011-R3A-SM-3P5ZM | 3.42896915 | 6.3813367 | 4.5912758 | Normal |
| GTEX-131XH-0011-R6a-SM-5KM2G | 2.2543719 | 0.7001999 | 2.8284109 | Normal |
| GTEX-12WS9-0011-R10a-SM-57WBV | 3.77838407 | 7.0009481 | 4.4766479 | Normal |
| GTEX-131YS-0011-R10b-SM-5EQ5N | 2.19043772 | 1.5309822 | 2.8771501 | Normal |
| GTEX-13VXU-0011-R3b-SM-5O9CU | 2.17863122 | 2.8574366 | 2.6227692 | Normal |
| GTEX-OXRO-0011-R9A-SM-5S2W3 | 4.86090666 | 5.7366316 | 5.2860351 | Normal |
| GTEX-13X6J-0011-R3b-SM-5P9K5 | 2.3035623 | 1.2826994 | 2.8567188 | Normal |
| GTEX-11ONC-0011-R8b-SM-5NQ87 | 2.48225458 | 2.295422 | 3.4214251 | Normal |
| GTEX-12126-0011-R11a-SM-5BC6X | 1.82567055 | 0.5200059 | 2.0115539 | Normal |
| GTEX-RU72-0011-R7A-SM-2TF5U | 3.1651301 | 2.7096043 | 3.7171812 | Normal |
| GTEX-11H98-0011-R11b-SM-5NQ6U | 1.89091012 | 0.7808922 | 2.5089748 | Normal |
| GTEX-WZTO-0011-R11A-SM-4E3K9 | 1.80950145 | 0.5452596 | 1.8964251 | Normal |
| GTEX-14BIM-3126-SM-5Q5C7 | 3.49321073 | 1.8434688 | 3.2042892 | Normal |
| GTEX-ZAB4-0011-R3a-SM-4RGNQ | 2.60554526 | 1.09145 | 2.8807624 | Normal |
| GTEX-13OVL-0011-R8a-SM-5L3HH | 2.00682187 | 1.9144345 | 2.9453984 | Normal |
| GTEX-14ABY-0011-R9a-SM-5PNZK | 3.05554464 | 4.4607635 | 3.5893476 | Normal |
| GTEX-NL4W-0011-R8a-SM-2I3G4 | 4.55372837 | 4.3302984 | 4.925341 | Normal |
| GTEX-11GSO-0011-R2a-SM-57WDF | 2.79077957 | 2.1206937 | 4.0052988 | Normal |
| GTEX-NL3H-0011-R1a-SM-48TDJ | 2.9933238 | 2.5994158 | 3.8126634 | Normal |
| GTEX-X261-0011-R10B-SM-4E3JT | 4.42853021 | 2.3024875 | 4.7825614 | Normal |
| GTEX-11ZTS-0011-R3a-SM-5BC78 | 2.77670868 | 2.2793793 | 4.017623 | Normal |
| GTEX-XLM4-0011-R6A-SM-4AT4B | 2.4256938 | 3.9814325 | 3.4624606 | Normal |
| GTEX-12WSB-0011-R11b-SM-57WBJ | 2.03917954 | 0.6144352 | 2.6666371 | Normal |
| GTEX-N7MT-0011-R6a-SM-2I3G3 | 3.68462655 | 1.3933728 | 4.0369291 | Normal |
| GTEX-T5JC-2326-SM-32PMR | 1.67214504 | 1.7555009 | 2.0838203 | Normal |
| GTEX-145MH-0011-R5a-SM-5P9JT | 2.13439728 | 0.7817954 | 2.7173689 | Normal |
| GTEX-13NZ8-3026-SM-5L3D4 | 1.66646815 | 0.7039145 | 2.7061464 | Normal |
| GTEX-13OVH-3026-SM-5MR4N | 2.98947838 | 1.4652452 | 3.790482 | Normal |
| GTEX-YJ89-0011-R11a-SM-4SOKA | 1.40266339 | 0.2634643 | 1.455038 | Normal |
| GTEX-13N1W-0011-R10b-SM-5MR4H | 2.7430099 | 5.7677376 | 3.4140698 | Normal |
| GTEX-145MI-3126-SM-5Q5CF | 3.31524037 | 5.0284371 | 3.5919194 | Normal |
| GTEX-13OW8-0011-R10a-SM-5L3H6 | 3.16675239 | 3.780781 | 3.9698531 | Normal |
| GTEX-13OW8-0011-R8a-SM-5L3G5 | 3.6168476 | 5.229848 | 4.7005915 | Normal |
| GTEX-QVUS-0011-R8A-SM-3GAD7 | 3.15256919 | 1.8876633 | 3.5002511 | Normal |
| GTEX-P44H-0011-R4A-SM-2XCEW | 2.79723801 | 1.580744 | 3.6043454 | Normal |
| GTEX-RU72-0011-R2A-SM-2TF6O | 1.98314136 | 3.1078703 | 3.6365001 | Normal |
| GTEX-X585-0011-R8A-SM-46MUX | 2.73252654 | 3.1228726 | 3.121461 | Normal |
| GTEX-145MH-0011-R3b-SM-5P9JQ | 1.77060622 | 1.2320381 | 2.7069578 | Normal |
| GTEX-11ZUS-0011-R8a-SM-5BC73 | 1.70117364 | 0.9763119 | 1.8314169 | Normal |
| GTEX-NPJ8-2626-SM-2D7W2 | 3.25754414 | 0.1695219 | 3.7848312 | Normal |
| GTEX-YFC4-0011-R4a-SM-4RGLQ | 3.21129156 | 3.8676427 | 3.8676427 | Normal |
| GTEX-13NZA-0011-R8b-SM-5KM2Q | 2.91651383 | 3.999503 | 3.6648076 | Normal |
| GTEX-Y8DK-0011-R5B-SM-4RTW6 | 2.83566497 | 1.9082203 | 3.009048 | Normal |
| GTEX-11ZTS-0011-R1a-SM-5BC71 | 3.06860716 | 3.5899971 | 3.8391162 | Normal |
| GTEX-YJ89-0011-R9a-SM-4SOK7 | 2.76065714 | 3.1390166 | 3.4578637 | Normal |
| GTEX-139TU-0011-R5b-SM-5L3E1 | 3.89521132 | 3.37684 | 5.0613395 | Normal |
| GTEX-NL3H-0011-R11A-SM-2I3E6 | 1.63373241 | 0.7358487 | 2.0998747 | Normal |
| GTEX-N7MS-0011-R2a-SM-2HML6 | 1.99434622 | 2.2363183 | 2.7300593 | Normal |
| GTEX-13FTY-0011-R1a-SM-5LZXE | 2.47329047 | 1.6132671 | 3.9220185 | Normal |
| GTEX-12WSF-0011-R4b-SM-5HL88 | 3.97424718 | 5.2940525 | 4.7058595 | Normal |
| GTEX-11ZTS-0011-R2b-SM-5BC75 | 2.72337839 | 3.2503123 | 3.7292735 | Normal |
| GTEX-T5JC-0011-R11A-SM-32PMB | 1.72056388 | 2.1195356 | 2.0707235 | Normal |
| GTEX-1399T-3026-SM-5KLZC | 2.93701337 | 1.5763077 | 3.5491521 | Normal |
| GTEX-12WS9-0011-R2a-SM-5CVNE | 4.61578376 | 4.1444488 | 5.4773483 | Normal |
| GTEX-139T8-0011-R1a-SM-5HL75 | 3.06144164 | 1.4388043 | 4.0444629 | Normal |
| GTEX-X4XX-0011-R4B-SM-46MWL | 3.04671921 | 2.43919 | 3.6896192 | Normal |
| GTEX-NPJ8-2626-SM-26GMZ | 2.53924577 | 0.1153011 | 2.7714048 | Normal |
| GTEX-T5JC-2426-SM-3NMDB | 2.8107928 | 2.3665725 | 3.1883788 | Normal |
| GTEX-11ZUS-0011-R11b-SM-5BC7B | 1.38582408 | 0.6924545 | 1.2772999 | Normal |
| GTEX-13RTJ-3126-SM-5S2Q4 | 2.13274323 | 0.5021305 | 2.7057457 | Normal |
| GTEX-13CZV-0011-R9a-SM-5LZXT | 3.82832912 | 6.8672592 | 5.0196512 | Normal |
| GTEX-13CZV-0011-R5a-SM-5K7XA | 3.04217424 | 3.7930888 | 4.446592 | Normal |
| GTEX-13OW6-0011-R5a-SM-5O9BK | 3.03832027 | 2.1905686 | 3.4826591 | Normal |
| GTEX-12WSM-0011-R6b-SM-5EQ6N | 2.34807233 | 0.5525419 | 3.4450667 | Normal |
| GTEX-13N1W-0011-R5b-SM-5MR5Q | 4.11483998 | 6.8614104 | 4.4459479 | Normal |
| GTEX-ZZPT-3026-SM-5GZXH | 5.54119507 | 3.0603077 | 4.8175296 | Normal |
| GTEX-12ZZY-0011-R6b-SM-5EGLA | 2.60719776 | 1.4168844 | 3.4157565 | Normal |
| GTEX-145MI-0011-R4b-SM-5PNZH | 4.65066967 | 4.760401 | 5.1299576 | Normal |
| GTEX-13QIC-0011-R11b-SM-5O9BU | 1.22951564 | 0.3104789 | 1.5244186 | Normal |
| GTEX-WVLH-0011-R1A-SM-4MVOK | 2.62034058 | 1.6406356 | 3.7650743 | Normal |
| GTEX-13OVJ-0011-R5a-SM-5P9H7 | 4.46322755 | 4.9592348 | 5.2657471 | Normal |
| GTEX-11GSP-3226-SM-5986O | 3.33400821 | 4.2657013 | 4.2540787 | Normal |
| GTEX-13NYS-0011-R8a-SM-5MR5H | 5.57123792 | 4.7378094 | 5.6234578 | Normal |
| GTEX-13OW6-3026-SM-5J2MI | 2.31080884 | 1.262527 | 3.2297454 | Normal |
| GTEX-ZE7O-0011-R7a-SM-57WCU | 3.2096871 | 2.103186 | 3.945101 | Normal |
| GTEX-ZUA1-0011-R3a-SM-4YCEE | 2.28624783 | 2.3126864 | 3.1048698 | Normal |
| GTEX-XMD1-0011-R6A-SM-4AT5K | 4.45701345 | 0.5840617 | 4.6797285 | Normal |
| GTEX-13X6K-0011-R10a-SM-5P9JR | 3.26290828 | 1.8398656 | 3.6069471 | Normal |
| GTEX-ZF28-0011-R1a-SM-4WWD1 | 3.73688685 | 0.9214401 | 4.4333974 | Normal |
| GTEX-ZF28-0011-R6a-SM-4WKHI | 5.16669339 | 4.4388929 | 5.1737686 | Normal |
| GTEX-131XH-0011-R2b-SM-5DUVY | 2.77364848 | 1.6343771 | 3.8733447 | Normal |
| GTEX-13RTJ-0011-R11a-SM-5O9CK | 2.10080915 | 0.3648373 | 2.057078 | Normal |
| GTEX-13OW5-0011-R4b-SM-5L3HS | 5.44992927 | 6.6464169 | 5.9083836 | Normal |
| GTEX-RVPV-0011-R5A-SM-2TF69 | 3.91502761 | 2.9750431 | 3.7323142 | Normal |
| GTEX-12WSF-0011-R7b-SM-5HL99 | 4.58988237 | 3.6828314 | 4.3136738 | Normal |
| GTEX-WZTO-0011-R1B-SM-3NMAR | 2.23802534 | 1.7193451 | 2.7894937 | Normal |
| GTEX-WL46-0011-R1A-SM-3LK6M | 2.65330271 | 2.2506034 | 3.0946865 | Normal |
| GTEX-UTHO-2926-SM-3P5Z9 | 2.02312407 | 0.4116968 | 2.3577186 | Normal |
| GTEX-12WSE-0011-R1b-SM-5GU4V | 1.97425068 | 1.1787701 | 2.9203192 | Normal |
| GTEX-ZE9C-0011-R6a-SM-4WWCW | 3.82084041 | 4.3796063 | 4.7436928 | Normal |
| GTEX-144GL-0011-R1a-SM-5PNZ5 | 1.75401529 | 1.5854449 | 3.0621422 | Normal |
| GTEX-13O3Q-0011-R8b-SM-5KM2J | 3.2047239 | 2.3669791 | 3.251476 | Normal |
| GTEX-PWO3-0011-R2A-SM-2S1OX | 3.96401029 | 2.3880963 | 4.899664 | Normal |
| GTEX-12ZZY-2926-SM-5DUXO | 1.68412609 | 0.4136058 | 1.9034944 | Normal |
| GTEX-11OF3-3126-SM-5GU5E | 2.11206543 | 1.1654007 | 3.3744228 | Normal |
| GTEX-Z93S-0011-R6b-SM-4RGNJ | 2.28286624 | 1.0730186 | 2.6333198 | Normal |
| GTEX-139UC-0011-R11a-SM-5KM3J | 2.48794668 | 0.6843169 | 2.8633881 | Normal |
| GTEX-13G51-0011-R2b-SM-5LZXS | 2.54100031 | 2.6295546 | 3.7811158 | Normal |
| GTEX-YJ89-0011-R6a-SM-4V6GN | 2.80488963 | 1.8447978 | 2.9266326 | Normal |
| GTEX-ZDXO-0011-R11a-SM-4WWD9 | 1.32039943 | 0.5580877 | 1.4318835 | Normal |
| GTEX-14BIL-0011-R9a-SM-5SI7A | 4.72045512 | 5.3894096 | 4.9504678 | Normal |
| GTEX-Z93S-0011-R2a-SM-4RGNG | 1.55764655 | 1.0536883 | 2.042318 | Normal |
| GTEX-12ZZZ-0011-R5a-SM-5EQ4M | 3.39711339 | 1.9724637 | 3.624719 | Normal |
| GTEX-13OW8-0011-R7a-SM-5L3FP | 3.4617295 | 2.9264833 | 3.9347155 | Normal |
| GTEX-13FHP-0011-R6b-SM-5LZY6 | 1.96412071 | 0.9632313 | 3.1683123 | Normal |
| GTEX-13S7M-0011-R11b-SM-5P9HU | 1.26242905 | 0.2231826 | 1.6731264 | Normal |
| GTEX-TSE9-3026-SM-3DB76 | 2.53911005 | 1.1961826 | 2.9907411 | Normal |
| GTEX-12WSC-3026-SM-5GCNF | 2.39996402 | 1.8054301 | 3.034008 | Normal |
| GTEX-QVUS-0011-R4A-SM-3GAE7 | 3.545364 | 4.5185632 | 3.9585321 | Normal |
| GTEX-11UD1-0011-R5b-SM-5P9FP | 3.18275015 | 1.1870583 | 4.2933985 | Normal |
| GTEX-13FLW-1426-SM-5K7YE | 2.52446995 | 1.355677 | 2.6237793 | Normal |
| GTEX-13JVG-0011-R7b-SM-5MR3Q | 4.39470343 | 3.3164454 | 4.5506684 | Normal |
| GTEX-145MH-0011-R7a-SM-5PNWH | 2.51752315 | 1.1843113 | 2.7209243 | Normal |
| GTEX-139TS-0011-R7a-SM-5K7X7 | 4.2205705 | 4.619895 | 4.8348653 | Normal |
| GTEX-ZVZQ-0011-R1a-SM-57WBU | 2.6180896 | 1.8357499 | 3.2841917 | Normal |
| GTEX-QMR6-0011-R10A-SM-32PKO | 2.77683823 | 0.9018201 | 3.1366774 | Normal |
| GTEX-WL46-0011-R11A-SM-3MJFT | 1.96848996 | 1.2871307 | 1.5855829 | Normal |
| GTEX-12WSD-0011-R6b-SM-5P9JU | 1.44550844 | 1.541086 | 2.6863221 | Normal |
| GTEX-13X6J-0011-R4b-SM-5P9K4 | 2.66201882 | 2.103186 | 3.4289692 | Normal |
| GTEX-11GSP-0011-R11b-SM-5NQ7V | 1.3593472 | 1.3824075 | 1.7767352 | Normal |
| GTEX-X4EP-0011-R4B-SM-3P627 | 7.57202747 | 6.4294438 | 5.6622551 | Normal |
| GTEX-WVLH-0011-R10A-SM-3MJFM | 2.26155085 | 0.642028 | 2.140745 | Normal |
| GTEX-Q2AG-0011-R1A-SM-2HMJI | 1.99278788 | 1.3114627 | 2.9445679 | Normal |
| GTEX-X261-3126-SM-4PQZC | 2.8946031 | 0.3636468 | 2.7559134 | Normal |
| GTEX-Z93S-2926-SM-57WB9 | 2.80474047 | 0.4455312 | 2.7631588 | Normal |
| GTEX-13OVJ-0011-R6b-SM-5L3GH | 3.81855326 | 4.8426565 | 4.8442684 | Normal |
| GTEX-13IVO-0011-R10a-SM-5LZY3 | 3.2481424 | 1.7577056 | 4.7214646 | Normal |
| GTEX-11DZ1-2926-SM-5A5KI | 3.78028437 | 1.4702695 | 3.5424884 | Normal |
| GTEX-13X6I-0011-R7b-SM-5PNWP | 3.94124094 | 2.8850417 | 4.4940695 | Normal |
| GTEX-RVPU-0011-R9A-SM-3NM8E | 3.396236 | 2.181438 | 3.917567 | Normal |
| GTEX-13NZA-0011-R1b-SM-5LUAF | 3.20387668 | 3.2614172 | 3.7311265 | Normal |
| GTEX-13NZA-0011-R5b-SM-5KM4K | 4.01969976 | 2.3970613 | 4.5703405 | Normal |
| GTEX-13OVH-2926-SM-5IJFH | 1.92110281 | 0.5580272 | 2.601219 | Normal |
| GTEX-P44H-0011-R1A-SM-3NM8J | 2.88332118 | 1.5214003 | 3.6235907 | Normal |
| GTEX-13CIG-0011-R5b-SM-5L3F2 | 3.53106919 | 3.71307 | 4.6981138 | Normal |
| GTEX-13OW5-0011-R11b-SM-5L3GG | 2.87938733 | 2.608903 | 3.6981285 | Normal |
| GTEX-13JVG-0011-R6a-SM-5MR3E | 3.75858332 | 2.8860134 | 4.3845151 | Normal |
| GTEX-11ZU8-0011-R3a-SM-5BC6U | 2.80665742 | 2.0207604 | 3.3282455 | Normal |
| GTEX-132Q8-0011-R2b-SM-5EQ5Z | 4.53958783 | 3.1314932 | 4.542111 | Normal |
| GTEX-YFC4-3026-SM-5IFJK | 3.47402574 | 2.1517841 | 3.4905763 | Normal |
| GTEX-145MH-2926-SM-5Q5D2 | 1.3362878 | 0.6719182 | 1.5545237 | Normal |
| GTEX-13JUV-0011-R6b-SM-5LZYG | 3.45723831 | 3.583215 | 4.3076297 | Normal |
| GTEX-T6MN-0011-R5A-SM-32QPD | 2.27858084 | 2.1235123 | 2.6826484 | Normal |
| GTEX-139TT-0011-R3b-SM-5K7VL | 2.49371435 | 1.4080022 | 3.1097268 | Normal |
| GTEX-13CF2-0011-R11b-SM-5LZXO | 1.44217275 | 0.5275184 | 1.8715051 | Normal |
| GTEX-144GL-3026-SM-5Q5CW | 2.40104899 | 1.0619091 | 3.0744585 | Normal |
| GTEX-QDT8-2926-SM-32PKC | 2.63691854 | 2.5531933 | 3.4550743 | Normal |
| GTEX-11NV4-0011-R7a-SM-57WDH | 2.59167108 | 1.3491722 | 2.6199166 | Normal |
| GTEX-14ASI-0011-R1b-SM-5S2TY | 2.48266799 | 1.4987778 | 3.1974366 | Normal |
| GTEX-ZAB4-0011-R11a-SM-4SOKI | 1.62195087 | 0.1590534 | 1.7252376 | Normal |
| GTEX-WZTO-2826-SM-3NM8P | 1.79659012 | 0.5577734 | 1.8239567 | Normal |
| GTEX-XLM4-0011-R2B-SM-4AT5Z | 3.25798465 | 4.0911883 | 4.4176099 | Normal |
| GTEX-QDT8-0011-R9A-SM-32PKH | 3.731814 | 3.082941 | 3.645082 | Normal |
| GTEX-ZYFD-2926-SM-5GID9 | 2.4390479 | 5.0077993 | 3.8288399 | Normal |
| GTEX-X585-0011-R4B-SM-46MVH | 3.62439257 | 3.9150276 | 4.2766459 | Normal |
| GTEX-QMR6-0011-R6A-SM-32PKP | 2.59494024 | 0.6944869 | 2.8331471 | Normal |
| GTEX-13112-0011-R1b-SM-5HL7K | 1.81765937 | 2.1657758 | 2.99444 | Normal |
| GTEX-Z93S-0011-R3b-SM-4RGNH | 1.95585357 | 0.6788938 | 2.2465493 | Normal |
| GTEX-T5JC-0011-R10A-SM-32PM2 | 2.84704968 | 2.9311174 | 3.4450667 | Normal |
| GTEX-X585-0011-R2B-SM-46MVF | 3.84597701 | 4.4748528 | 4.3076297 | Normal |
| GTEX-WL46-0011-R3A-SM-3TW8E | 2.21338513 | 1.7853232 | 3.028758 | Normal |
| GTEX-13X6I-0011-R1b-SM-5PNZC | 3.28520957 | 2.7390642 | 3.8852914 | Normal |
| GTEX-R55E-0011-R5A-SM-2TC5N | 2.2181611 | 1.227265 | 3.1137209 | Normal |
| GTEX-WVLH-3026-SM-3MJG9 | 2.38275138 | 0.7984279 | 2.587546 | Normal |
| GTEX-13OW5-0011-R5b-SM-5L3FR | 5.03643792 | 5.0536755 | 5.5976562 | Normal |
| GTEX-Y8DK-0826-SM-4TT3T | 2.22294817 | 1.6568416 | 2.9966101 | Normal |
| GTEX-ZUA1-0011-R11b-SM-51MTI | 1.91323547 | 1.0702872 | 1.8210398 | Normal |
| GTEX-13CF2-0011-R1a-SM-5LZY1 | 2.4308658 | 3.2316676 | 3.9321445 | Normal |
| GTEX-12WSH-3126-SM-5LZW8 | 1.80034144 | 0.5117327 | 2.4661512 | Normal |
| GTEX-12696-3026-SM-5FQTU | 2.86421508 | 5.4095602 | 3.8267841 | Normal |
| GTEX-WL46-0011-R6A-SM-3LK6X | 2.38875616 | 2.5394913 | 3.0684722 | Normal |
| GTEX-139UC-0011-R9a-SM-5K7W7 | 5.12009069 | 5.7886946 | 4.901915 | Normal |
| GTEX-N7MS-0011-R10A-SM-2HMJK | 2.44780355 | 1.6780838 | 2.6883291 | Normal |
| GTEX-13SLX-0011-R11b-SM-5O9C8 | 1.26456326 | 0.7743668 | 1.248903 | Normal |
| GTEX-147GR-0011-R2b-SM-5S2RQ | 2.73085825 | 1.7565638 | 3.5280808 | Normal |
| GTEX-145MG-3026-SM-5RQJA | 2.51339066 | 1.7864809 | 3.2560748 | Normal |
| GTEX-13O3O-0011-R11b-SM-5KM4G | 1.64197587 | 1.4348127 | 2.5045845 | Normal |
| GTEX-YFC4-0011-R5a-SM-4RGLR | 3.10299403 | 3.4700573 | 4.111553 | Normal |
| GTEX-13PVQ-0011-R9b-SM-5LU3B | 3.75944631 | 5.6271737 | 4.6305759 | Normal |
| GTEX-12WSD-0011-R2a-SM-5LZWB | 2.2269703 | 2.0298093 | 3.5347056 | Normal |
| GTEX-12WSB-0011-R1b-SM-5P9EY | 3.50764866 | 2.118297 | 3.6543091 | Normal |
| GTEX-12ZZW-0011-R4a-SM-5DUX9 | 2.959283 | 1.4480139 | 4.192321 | Normal |
| GTEX-ZE7O-0011-R5a-SM-57WBO | 3.1441993 | 1.6986663 | 3.6045165 | Normal |
| GTEX-WZTO-0011-R3B-SM-3NMC6 | 1.99406177 | 0.8826392 | 2.4285926 | Normal |
| GTEX-13VXU-0011-R4b-SM-5O9CI | 2.39559711 | 3.6356719 | 3.8091773 | Normal |
| GTEX-11EQ8-2826-SM-5N9C1 | 2.11812816 | 1.453871 | 2.6877802 | Normal |
| GTEX-QVUS-2926-SM-3GIJB | 1.82142891 | 0 | 2.3349743 | Normal |
| GTEX-P44H-0011-R3A-SM-2XCEQ | 3.8332483 | 1.9509563 | 4.1715287 | Normal |
| GTEX-WVLH-0011-R4A-SM-3MJFS | 2.75631012 | 1.6021607 | 3.4408913 | Normal |
| GTEX-XOTO-3026-SM-4B65M | 2.53785385 | 1.350151 | 3.0714856 | Normal |
| GTEX-13SLW-0011-R3b-SM-5SI6Y | 3.06061118 | 1.8975406 | 3.9899179 | Normal |
| GTEX-13JVG-0011-R5a-SM-5MR4O | 4.15051313 | 3.0401579 | 4.5499654 | Normal |
| GTEX-111FC-3126-SM-5GZZ2 | 2.17697621 | 1.0429891 | 2.7751898 | Normal |
| GTEX-1117F-3226-SM-5N9CT | 2.97946095 | 1.4022969 | 3.948722 | Normal |
| GTEX-13JUV-0011-R7b-SM-5LZZG | 4.1371957 | 2.7413311 | 4.5998462 | Normal |
| GTEX-145MI-0011-R5a-SM-5Q5B4 | 4.3879804 | 8.0465248 | 5.0663182 | Normal |
| GTEX-13JVG-3226-SM-5IJGJ | 2.54747589 | 2.2212552 | 3.0699247 | Normal |
| GTEX-WHSE-0011-R11A-SM-3P5YY | 2.24956629 | 3.5547935 | 3.6901056 | Normal |
| GTEX-13JVG-0011-R8a-SM-5KM3E | 2.99074109 | 3.2606687 | 4.4585696 | Normal |
| GTEX-QDT8-0011-R9A-SM-5SI8J | 3.48359468 | 3.2369769 | 3.8123199 | Normal |
| GTEX-13OW5-0011-R6b-SM-5O9BW | 4.16509452 | 5.3421585 | 4.41915 | Normal |
| GTEX-OXRO-0011-R9A-SM-3NB1X | 4.7003413 | 5.7434174 | 5.2571333 | Normal |
| GTEX-RNOR-2326-SM-2TF4I | 2.10117903 | 0.7141242 | 2.9467068 | Normal |
| GTEX-WL46-2826-SM-3LK81 | 1.51398998 | 0.4963011 | 1.4038854 | Normal |
| GTEX-YJ89-0011-R1a-SM-4RGLS | 2.91794337 | 2.7854536 | 3.6992892 | Normal |
| GTEX-13NYB-0011-R6b-SM-5MR51 | 2.54858266 | 1.2685431 | 3.14712 | Normal |
| GTEX-11WQK-0011-R8a-SM-5BC7C | 3.0958641 | 6.3520082 | 4.8976873 | Normal |
| GTEX-WL46-0011-R2A-SM-3LK6O | 2.48323368 | 2.4403002 | 2.8636668 | Normal |
| GTEX-RU72-0011-R8A-SM-2TF61 | 2.17456039 | 2.0841 | 3.3187778 | Normal |
| GTEX-13N2G-0011-R11a-SM-5MR3F | 2.32841639 | 0.7023617 | 2.4818577 | Normal |
| GTEX-ZVZQ-0011-R6b-SM-57WAY | 2.43167078 | 1.4749712 | 3.4123746 | Normal |
| GTEX-ZVT3-0011-R5a-SM-51MSI | 3.36434082 | 2.2042371 | 4.0282666 | Normal |
| GTEX-12ZZZ-3126-SM-5LZU6 | 1.775999 | 0.8149357 | 1.9373901 | Normal |
| GTEX-RVPU-0011-R4A-SM-5SI8K | 3.2683346 | 1.5281719 | 4.2016265 | Normal |
| GTEX-13NZA-0011-R10b-SM-5KM54 | 2.98609073 | 1.9813522 | 4.0953883 | Normal |
| GTEX-XMD1-0011-R5A-SM-4AT47 | 4.85976975 | 2.101179 | 4.8217987 | Normal |
| GTEX-14ABY-0011-R6a-SM-5PNWR | 2.33595498 | 1.1515178 | 2.7768382 | Normal |
| GTEX-WWYW-0011-R10A-SM-3NB35 | 3.34169156 | 2.157418 | 4.1381669 | Normal |
| GTEX-T6MN-0011-R2A-SM-32QOW | 2.33102667 | 2.3095736 | 3.9216735 | Normal |
| GTEX-NL4W-0011-R11A-SM-2I3DW | 2.74670717 | 2.0128056 | 3.105157 | Normal |
| GTEX-1313W-0011-R3a-SM-5EGLI | 2.07196888 | 1.7541272 | 2.5791876 | Normal |
| GTEX-13VXU-0011-R5a-SM-5O9D7 | 2.60203324 | 3.4525018 | 3.0872492 | Normal |
| GTEX-12WSD-0011-R1b-SM-5LZVY | 1.79528825 | 1.8885934 | 2.9544761 | Normal |
| GTEX-P44H-0011-R3A-SM-5SI8A | 3.13359783 | 1.83408 | 4.0122277 | Normal |
| GTEX-TSE9-0011-R10A-SM-3DB7O | 2.44471597 | 1.8587661 | 3.3028212 | Normal |
| GTEX-1445S-0011-R6b-SM-5PNUF | 4.01112605 | 3.7279018 | 5.3882335 | Normal |
| GTEX-ZV68-0011-R2a-SM-4YCDK | 3.36614386 | 2.608903 | 4.163523 | Normal |
| GTEX-XLM4-0011-R9A-SM-4AT45 | 2.93647873 | 4.0386357 | 4.4576774 | Normal |
| GTEX-13JUV-0011-R11a-SM-5LZYH | 2.15204866 | 1.0835789 | 2.6486765 | Normal |
| GTEX-S7PM-0011-R5A-SM-3NM8G | 3.68529424 | 1.938597 | 4.3349064 | Normal |
| GTEX-13FXS-0011-R1a-SM-5K7U6 | 2.87416005 | 2.3256914 | 3.3201585 | Normal |
| GTEX-144GL-0011-R11b-SM-5P9F6 | 1.45046419 | 0.6089459 | 1.8974059 | Normal |
| GTEX-X4XX-0011-R5A-SM-46MWN | 3.78708057 | 3.0033083 | 4.3003097 | Normal |
| GTEX-145LU-0011-R9b-SM-5SI66 | 2.32245068 | 3.2361116 | 3.2536988 | Normal |
| GTEX-13OW6-0011-R2a-SM-5L3HG | 2.34312935 | 2.00081 | 3.4607924 | Normal |
| GTEX-14DAQ-3026-SM-5S2QZ | 1.52303742 | 0.4303784 | 2.4633343 | Normal |
| GTEX-13FHO-0011-R1b-SM-5KLZG | 3.205721 | 4.8467669 | 4.5804328 | Normal |
| GTEX-13N1W-0011-R4a-SM-5MR52 | 3.30771273 | 8.0600409 | 4.5731431 | Normal |
| GTEX-X4EP-0011-R1A-SM-3P624 | 6.60170772 | 7.0538961 | 5.6013666 | Normal |
| GTEX-12WS9-0011-R4b-SM-5P9EU | 3.52540744 | 2.8670049 | 4.9301056 | Normal |
| GTEX-13RTJ-0011-R10b-SM-5O9CW | 2.98651449 | 1.0552032 | 2.9046587 | Normal |
| GTEX-13S7M-0011-R6b-SM-5O9D8 | 2.30931952 | 0.6249716 | 3.3460318 | Normal |
| GTEX-13O3O-0011-R6b-SM-5P9GY | 3.04629373 | 3.6613893 | 3.7884571 | Normal |
| GTEX-12WSH-0011-R9b-SM-5LZUG | 3.85719934 | 4.4757536 | 4.538879 | Normal |
| GTEX-12126-1026-SM-5P9JJ | 3.09928078 | 2.2280471 | 3.1463855 | Normal |
| GTEX-WWYW-3126-SM-3NB39 | 3.58370674 | 2.5598219 | 4.8142992 | Normal |
| GTEX-13CF2-0011-R5a-SM-5LZWS | 3.42602625 | 2.3390631 | 3.9674739 | Normal |
| GTEX-13VXU-2926-SM-5LU5C | 2.60288593 | 2.2708021 | 3.0293352 | Normal |
| GTEX-ZAB4-0011-R8b-SM-4SOKF | 2.7672114 | 1.6070307 | 3.6888027 | Normal |
| GTEX-13NYB-3026-SM-5IJD7 | 2.50925578 | 1.6406356 | 2.8118175 | Normal |
| GTEX-13CIG-0011-R8b-SM-5LZX5 | 4.43889288 | 5.3836068 | 4.8127369 | Normal |
| GTEX-UTHO-0011-R5A-SM-3GIJD | 2.4233486 | 1.764402 | 3.1549795 | Normal |
| GTEX-13O3O-0011-R8b-SM-5LUAL | 3.05944659 | 2.8833212 | 3.8197945 | Normal |
| GTEX-X4XX-0011-R3B-SM-46MWK | 2.95180214 | 1.9790325 | 3.1968578 | Normal |
| GTEX-NPJ7-0011-R2a-SM-2I3GF | 3.50671335 | 2.5155665 | 4.4364402 | Normal |
| GTEX-ZE9C-0011-R3a-SM-4WKGT | 3.24254465 | 3.7387296 | 4.0799875 | Normal |
| GTEX-13QJC-0011-R8b-SM-5S2U6 | 2.32018729 | 0.5964246 | 4.1589761 | Normal |
| GTEX-12WS9-0011-R3a-SM-5CVNF | 4.94637791 | 3.7409424 | 5.3172927 | Normal |
| GTEX-ZUA1-0011-R6b-SM-4YCDD | 2.76666222 | 2.6170284 | 3.5563957 | Normal |
| GTEX-13PVQ-0011-R7a-SM-5L3G3 | 3.81561305 | 7.0639942 | 4.4541098 | Normal |
| GTEX-13NYS-0011-R3b-SM-5MR41 | 5.35481853 | 3.3550216 | 5.4012486 | Normal |
| GTEX-13X6K-0011-R7b-SM-5P9K7 | 3.82014684 | 2.0094422 | 4.0305228 | Normal |
| GTEX-1399T-0011-R5b-SM-5IJDK | 3.3047529 | 2.7233784 | 3.2750131 | Normal |
| GTEX-145MH-3026-SM-5Q5DZ | 2.18922025 | 1.3061086 | 2.855234 | Normal |
| GTEX-Y8DK-0011-R10A-SM-4SOK1 | 2.10331534 | 1.028882 | 2.4409691 | Normal |
| GTEX-13OW8-0011-R1a-SM-5L3HI | 3.61504784 | 3.47656 | 4.6189206 | Normal |
| GTEX-14753-0011-R6b-SM-5S2UD | 3.1303277 | 2.460816 | 4.2733244 | Normal |
| GTEX-13X6K-0011-R5b-SM-5PNUE | 3.75554744 | 2.0309141 | 3.8069291 | Normal |
| GTEX-NL3H-0011-R7a-SM-2I3G5 | 3.94510095 | 3.1691813 | 4.6752841 | Normal |
| GTEX-ZDXO-0011-R5a-SM-4WKG5 | 3.55707513 | 3.6202731 | 3.9623468 | Normal |
| GTEX-X4EP-0011-R3A-SM-3P626 | 7.33900327 | 7.2805522 | 5.6423216 | Normal |
| GTEX-13N2G-0011-R3b-SM-5MR5G | 5.11751847 | 4.7859813 | 5.5359686 | Normal |
| GTEX-T2IS-3026-SM-32QPM | 1.82567055 | 1.1765996 | 2.582797 | Normal |
| GTEX-14BIN-0011-R6a-SM-5S2RH | 1.88167139 | 0.6021245 | 2.4665766 | Normal |
| GTEX-13OVH-0011-R3a-SM-5KM49 | 3.13538883 | 2.0085461 | 4.3815026 | Normal |
| GTEX-P44G-0011-R4A-SM-2I3FB | 3.620758 | 7.6831986 | 5.3076141 | Normal |
| GTEX-13PDP-0011-R6b-SM-5O9D5 | 3.3372066 | 2.2073658 | 5.0911812 | Normal |
| GTEX-NPJ8-0011-R1a-SM-2HMLC | 4.87492295 | 1.8477702 | 4.7844169 | Normal |
| GTEX-N7MS-0011-R7a-SM-2HMKN | 2.97746896 | 1.8944743 | 3.2238722 | Normal |
| GTEX-13SLW-0011-R2b-SM-5O9C6 | 3.52889416 | 1.8630747 | 5.131912 | Normal |
| GTEX-ZAJG-3026-SM-5HL92 | 2.6661055 | 2.1932711 | 3.8923768 | Normal |
| GTEX-WHSE-0011-R5A-SM-3P5ZO | 3.51629836 | 7.0401387 | 4.9060932 | Normal |
| GTEX-11ZUS-0011-R9b-SM-5BC76 | 2.02715546 | 2.2234444 | 2.7980881 | Normal |
| GTEX-RU72-2926-SM-2TF66 | 3.42864622 | 1.8850642 | 3.6643346 | Normal |
| GTEX-Q2AG-0011-R6A-SM-2HML7 | 2.33919511 | 0.8481921 | 3.2269735 | Normal |
| GTEX-11EI6-2926-SM-5985U | 4.07563457 | 1.7540153 | 3.4566327 | Normal |
| GTEX-R55F-0011-R5A-SM-2TF5L | 5.387037 | 2.955478 | 5.177188 | Normal |
| GTEX-13FXS-0011-R10a-SM-5J2MA | 2.44165152 | 0.8677081 | 2.711928 | Normal |
| GTEX-13O3Q-0011-R4a-SM-5P9H2 | 2.22086468 | 1.5350345 | 3.3155275 | Normal |
| GTEX-132Q8-2926-SM-5IFFP | 3.37107756 | 0.7002746 | 3.3074305 | Normal |
| GTEX-NL3H-0011-R5a-SM-2I3GB | 3.95035995 | 2.3994098 | 4.6138475 | Normal |
| GTEX-1399T-0011-R11a-SM-5J2O1 | 1.39087807 | 0.6974999 | 1.6512163 | Normal |
| GTEX-ZAB4-2926-SM-57WCV | 2.14031105 | 0.2735739 | 2.7325265 | Normal |
| GTEX-13N1W-0011-R9b-SM-5MR4R | 2.44016147 | 4.834058 | 4.1788146 | Normal |
| GTEX-13OVL-0011-R9a-SM-5P9GX | 1.79370808 | 1.9530595 | 2.7996127 | Normal |
| GTEX-13FXS-0011-R8a-SM-5J1MN | 2.32366114 | 2.1390686 | 3.0969691 | Normal |
| GTEX-ZAB4-0011-R9a-SM-4SOKG | 5.23507624 | 3.2667525 | 4.317176 | Normal |
| GTEX-S7SE-0011-R2A-SM-2XCDC | 2.389016 | 2.1159785 | 3.1990513 | Normal |
| GTEX-139TT-0011-R1a-SM-5LZVD | 2.19327111 | 1.6750648 | 2.582797 | Normal |
| GTEX-12696-2926-SM-5FQTG | 3.36357961 | 4.3552792 | 4.3565422 | Normal |
| GTEX-N7MT-0011-R10A-SM-2I3E1 | 3.1071907 | 0.8206313 | 3.4065728 | Normal |
| GTEX-13NYS-0011-R10a-SM-5MR4S | 4.71642469 | 3.5026414 | 4.4314039 | Normal |
| GTEX-11NV4-0011-R5a-SM-5NQ88 | 2.7409238 | 1.7975088 | 2.5919679 | Normal |
| GTEX-13CF2-0011-R3b-SM-5LZZ6 | 2.79108248 | 1.3916106 | 3.3854695 | Normal |
| GTEX-ZVZQ-0011-R4b-SM-57WCZ | 2.70834281 | 2.3043934 | 3.4826591 | Normal |
| GTEX-13RTJ-0011-R4b-SM-5PNX1 | 3.7216863 | 1.3435832 | 4.4139415 | Normal |
| GTEX-13NYS-0011-R11b-SM-5MR4P | 3.78743455 | 1.1862379 | 4.2448946 | Normal |
| GTEX-T6MN-0011-R10A-SM-32QP7 | 2.22154182 | 1.3292993 | 2.4906052 | Normal |
| GTEX-NPJ8-0011-R1a-SM-33HCB | 4.28307567 | 2.6800404 | 4.759913 | Normal |
| GTEX-13SLW-0011-R4b-SM-5S2W2 | 3.61102283 | 3.4318515 | 4.9886241 | Normal |
| GTEX-PVOW-2526-SM-2XCF7 | 1.83436014 | 1.5597084 | 2.7556056 | Normal |
| GTEX-OHPN-0011-R10A-SM-5S2U3 | 5.705309 | 4.837028 | 5.180212 | Normal |
| GTEX-13FHP-0011-R5b-SM-5LZY2 | 3.12229107 | 1.2365484 | 3.9050765 | Normal |
| GTEX-139T8-0011-R3b-SM-5HL4R | 3.39365081 | 2.2818023 | 4.2463 | Normal |
| GTEX-ZE7O-0011-R11a-SM-57WBC | 1.33812323 | 0.6500587 | 1.9090051 | Normal |
| GTEX-N7MT-0011-R11A-SM-2I3DZ | 3.22932144 | 0.7697909 | 3.9490865 | Normal |
| GTEX-NL4W-0011-R5a-SM-2I3GD | 4.49136055 | 3.2566778 | 5.3184623 | Normal |
| GTEX-147GR-0011-R10b-SM-5S2UM | 2.44644339 | 1.1100783 | 3.0905286 | Normal |
| GTEX-ZVZQ-0011-R8a-SM-51MR5 | 2.10704346 | 1.9469427 | 3.0784797 | Normal |
| GTEX-13CIG-0011-R3b-SM-5K7XF | 4.00734108 | 5.9095741 | 4.973008 | Normal |
| GTEX-13SLX-0011-R5a-SM-5O9BV | 2.67540705 | 1.7925858 | 2.8996849 | Normal |
| GTEX-13PVQ-0011-R5b-SM-5O9DI | 3.89097616 | 6.1489808 | 4.7510997 | Normal |
| GTEX-RU72-0011-R11A-SM-5SI8G | 1.68360745 | 0.9464816 | 2.2027555 | Normal |
| GTEX-S7SE-0011-R6A-SM-2XCD9 | 2.91807979 | 1.7464734 | 2.7594064 | Normal |
| GTEX-12WSA-0011-R4a-SM-57WB7 | 3.37942241 | 2.0930561 | 3.9418023 | Normal |
| GTEX-12WSH-0011-R1a-SM-5GU5V | 2.07084606 | 2.310298 | 3.7292735 | Normal |
| GTEX-145MI-0011-R11a-SM-5P9JZ | 2.65551783 | 3.1284644 | 2.8665791 | Normal |
| GTEX-12WSF-0011-R5a-SM-5HL8K | 5.02055515 | 4.6467942 | 4.5492754 | Normal |
| GTEX-PVOW-0011-R3A-SM-32PKX | 1.67289704 | 1.5438723 | 2.1625935 | Normal |
| GTEX-145LS-3126-SM-5Q5BY | 3.43748712 | 1.1495768 | 3.5339156 | Normal |
| GTEX-QVJO-0011-R6A-SM-2S1QN | 2.66719353 | 0.923506 | 3.2571332 | Normal |
| GTEX-145LS-0011-R8b-SM-5PNXA | 2.74543714 | 1.7081495 | 3.3416916 | Normal |
| GTEX-13OW8-2926-SM-5L3FV | 2.0349216 | 1.4603429 | 2.898975 | Normal |
| GTEX-ZVT3-0011-R7a-SM-57WCO | 3.27724884 | 1.6516168 | 4.3347008 | Normal |
| GTEX-QMR6-0011-R4A-SM-32PKU | 3.2170994 | 2.0819242 | 3.4696198 | Normal |
| GTEX-P44H-0011-R6A-SM-2XCF3 | 3.23712972 | 3.8468806 | 4.1660764 | Normal |
| GTEX-P44G-0011-R7A-SM-2I3F9 | 3.9926894 | 6.6860365 | 5.2798774 | Normal |
| GTEX-R55F-0011-R6A-SM-2TF4L | 4.7099371 | 1.8138359 | 4.7291185 | Normal |
| GTEX-YJ89-0011-R8b-SM-4SOK8 | 2.41267054 | 2.6695726 | 3.2718831 | Normal |
| GTEX-TSE9-0011-R1A-SM-3DB7E | 2.96708055 | 1.7637735 | 3.5424884 | Normal |
| GTEX-12ZZW-0011-R6a-SM-5DUVZ | 2.94946667 | 0.4345422 | 3.7563748 | Normal |
| GTEX-N7MS-0011-R1a-SM-2HMJG | 1.84318204 | 2.1110618 | 2.6199166 | Normal |
| GTEX-Q2AG-0011-R3A-SM-2HMJ9 | 2.4320791 | 0.8177788 | 2.6982167 | Normal |
| GTEX-T6MN-0011-R6A-SM-5CHQD | 0.01941244 | 0.0194124 | 0.0194124 | Normal |
| GTEX-12WSB-3026-SM-59HKM | 2.64867653 | 0.7790428 | 3.6284669 | Normal |
| GTEX-Z93S-2826-SM-57WBL | 1.64827414 | 0.1580471 | 1.7512957 | Normal |
| GTEX-ZE9C-3026-SM-4WKHB | 3.18756493 | 3.2591768 | 4.0191339 | Normal |
| GTEX-ZF28-0011-R3a-SM-4WWDA | 3.77872697 | 1.3785552 | 4.4681291 | Normal |
| GTEX-13SLX-0011-R6a-SM-5PNX4 | 2.77758333 | 2.4516386 | 3.2931394 | Normal |
| GTEX-13N1W-0011-R3a-SM-5MR5E | 2.6315043 | 6.6039194 | 3.8380732 | Normal |
| GTEX-XOTO-0011-R3A-SM-4B64W | 2.51325407 | 1.5366068 | 2.7666622 | Normal |
| GTEX-ZV68-0011-R10a-SM-51MT7 | 3.49758546 | 1.687094 | 4.1208022 | Normal |
| GTEX-13SLX-0011-R4a-SM-5O9BJ | 2.92619465 | 2.4996146 | 3.627328 | Normal |
| GTEX-147F4-0011-R10b-SM-5S2WC | 3.71932138 | 5.5077145 | 4.4017898 | Normal |
| GTEX-WZTO-0011-R7B-SM-4E3IS | 2.65714932 | 1.5861846 | 2.6156166 | Normal |
| GTEX-WHSE-3026-SM-3P5ZH | 3.12071567 | 5.7865153 | 4.2597871 | Normal |
| GTEX-WZTO-0011-R10B-SM-4E3KB | 2.55890421 | 1.1134567 | 2.2363183 | Normal |
| GTEX-145MI-0011-R10a-SM-5PNZD | 5.17886817 | 5.762046 | 5.8217213 | Normal |
| GTEX-13NYB-0011-R2a-SM-5MR3C | 3.15929572 | 3.347511 | 3.7504717 | Normal |
| GTEX-ZUA1-0011-R1a-SM-4YCE2 | 2.66146338 | 3.0501715 | 3.4837498 | Normal |
| GTEX-12ZZZ-0011-R3b-SM-5DUXK | 2.66569806 | 1.8476434 | 3.5021629 | Normal |
| GTEX-13O3O-0011-R7b-SM-5P9GZ | 3.18378503 | 2.87066 | 3.8683582 | Normal |
| GTEX-WVLH-0011-R8A-SM-3MJFC | 3.0886566 | 2.2761229 | 3.9436133 | Normal |
| GTEX-131XH-0011-R4b-SM-5DUWB | 2.28624783 | 2.6369185 | 3.1957041 | Normal |
| GTEX-147GR-0011-R9a-SM-5S2UY | 3.25531137 | 3.6989526 | 3.762006 | Normal |
| GTEX-13O3Q-3026-SM-5IJGD | 1.57794397 | 0.4704009 | 2.1454278 | Normal |
| GTEX-ZVT3-0011-R6a-SM-51MSU | 2.91436966 | 1.5531939 | 4.0251205 | Normal |
| GTEX-X4EP-0011-R8B-SM-4QASL | 5.99651434 | 6.5118781 | 4.4967985 | Normal |
| GTEX-YJ89-2926-SM-5P9IT | 1.65494347 | 0.5471015 | 2.2902367 | Normal |
| GTEX-ZAK1-2926-SM-5HL9S | 1.53111025 | 0.3532588 | 2.157418 | Normal |
| GTEX-11TUW-3126-SM-5GU4Y | 4.74369276 | 7.8381327 | 4.99337 | Normal |
| GTEX-XLM4-0011-R11A-SM-4AT46 | 4.38929603 | 3.1251311 | 4.2816182 | Normal |
| GTEX-T5JC-0011-R1A-SM-32PM6 | 2.80766365 | 5.2163963 | 4.1678242 | Normal |
| GTEX-13QIC-0011-R4a-SM-5PNX8 | 3.6347074 | 2.3811221 | 4.2303832 | Normal |
| GTEX-13CF2-0011-R2a-SM-5L3DC | 3.52494759 | 2.5342584 | 4.2360358 | Normal |
| GTEX-N7MS-0011-R5a-SM-2HMK8 | 2.22531978 | 1.0612752 | 2.8093831 | Normal |
| GTEX-ZDXO-0011-R2b-SM-4WKFG | 3.14814031 | 5.4441802 | 4.3750155 | Normal |
| GTEX-13G51-0011-R7b-SM-5LZXG | 2.01787898 | 0.8189755 | 2.334425 | Normal |
| GTEX-X585-0011-R3B-SM-46MVG | 3.47550328 | 2.9557675 | 3.6063133 | Normal |
| GTEX-13FLV-0011-R5a-SM-5LZWT | 3.32824546 | 2.4408446 | 3.8032764 | Normal |
| GTEX-13FHP-2926-SM-5IJG9 | 1.97328333 | 0.332615 | 2.3262816 | Normal |
| GTEX-12WSC-0011-R3a-SM-5P9F1 | 2.15907224 | 1.1145788 | 2.5862318 | Normal |
| GTEX-12WSA-0011-R10b-SM-5P9ET | 4.48339849 | 2.1961179 | 3.9926894 | Normal |
| GTEX-11O72-2826-SM-5HL6W | 2.63915895 | 3.9651217 | 3.4055221 | Normal |
| GTEX-11EI6-3026-SM-5GZZO | 3.45310739 | 2.445624 | 4.367089 | Normal |
| GTEX-148VJ-0011-R9b-SM-5SI77 | 2.4626317 | 2.3915883 | 3.458788 | Normal |
| GTEX-N7MT-1226-SM-2D7W4 | 2.43594041 | 0.2154842 | 3.0075929 | Normal |
| GTEX-T6MN-0011-R10A-SM-5CHQC | 0.0194635 | 0.0194635 | 0.0194635 | Normal |
| GTEX-11WQK-0011-R11a-SM-5BC6W | 2.29377974 | 2.5800154 | 2.1559059 | Normal |
| GTEX-12WSC-0011-R7b-SM-5LU9L | 2.55208935 | 1.3031085 | 2.5536227 | Normal |
| GTEX-12WSH-3026-SM-5CVNI | 2.16042118 | 0.7546641 | 4.2086989 | Normal |
| GTEX-XLM4-0011-R4B-SM-4AT5C | 2.5634339 | 2.2754532 | 3.7628549 | Normal |
| GTEX-13OVJ-0011-R8b-SM-5L3FS | 4.47485277 | 7.514044 | 5.3188213 | Normal |
| GTEX-13OW5-0011-R8a-SM-5L3GQ | 4.91827562 | 4.7948705 | 5.217448 | Normal |
| GTEX-X4XX-0011-R10B-SM-46MWO | 2.69529789 | 1.6944813 | 3.2419239 | Normal |
| GTEX-145MG-3126-SM-5Q5D3 | 1.94131841 | 0.8494826 | 2.1080992 | Normal |
| GTEX-13NZ8-0011-R8b-SM-5KM48 | 3.26420509 | 3.3498774 | 4.841092 | Normal |
| GTEX-12WSH-0011-R2a-SM-5GU68 | 3.2406305 | 4.1597745 | 4.9674112 | Normal |
| GTEX-131YS-0011-R3b-SM-5DUXB | 2.6720425 | 2.0622172 | 3.1123142 | Normal |
| GTEX-12ZZZ-3026-SM-5BC67 | 2.47188043 | 1.7627299 | 3.2705667 | Normal |
| GTEX-XMD1-0011-R8A-SM-4AT48 | 4.52290974 | 3.8203174 | 4.9580388 | Normal |
| GTEX-QDT8-0011-R11A-SM-32PKD | 1.32929933 | 0.6032932 | 2.1237995 | Normal |
| GTEX-145LS-0011-R6b-SM-5PNWL | 2.3394622 | 0.7786836 | 3.1647186 | Normal |
| GTEX-11OF3-0011-R8b-SM-57WCS | 2.57225121 | 1.8482385 | 3.1860853 | Normal |
| GTEX-PVOW-0011-R1A-SM-32PL6 | 1.8634563 | 1.9196599 | 2.4261149 | Normal |
| GTEX-QVJO-0011-R8A-SM-447C7 | 2.86406608 | 2.4165217 | 3.7479905 | Normal |
| GTEX-QVJO-0011-R5A-SM-2S1QM | 3.0437489 | 1.6395033 | 3.5879212 | Normal |
| GTEX-WZTO-0011-R6B-SM-4E3J6 | 2.36059708 | 1.0533344 | 2.9651173 | Normal |
| GTEX-13O3O-3026-SM-5KM42 | 1.94545658 | 2.1725444 | 3.0330131 | Normal |
| GTEX-13X6J-0011-R6a-SM-5PNUB | 2.27015465 | 1.1099741 | 2.5187612 | Normal |
| GTEX-ZYFD-3026-SM-5E44C | 3.16134408 | 7.0107539 | 4.2791263 | Normal |
| GTEX-13QIC-0011-R3a-SM-5P9HT | 2.85769646 | 1.7203131 | 3.5060942 | Normal |
| GTEX-13NZ8-0011-R1b-SM-5KM3V | 2.98721489 | 3.403535 | 4.9801679 | Normal |
| GTEX-14BMV-3026-SM-5S2PQ | 2.37155628 | 1.718087 | 2.9131337 | Normal |
| GTEX-13NYS-0011-R6b-SM-5MR3R | 5.11170722 | 1.6607395 | 4.8003864 | Normal |
| GTEX-13OW8-0011-R11a-SM-5L3GT | 1.86689483 | 1.4580459 | 2.5574883 | Normal |
| GTEX-139TS-3126-SM-5LZWX | 1.80046773 | 0.9791608 | 2.6824099 | Normal |
| GTEX-XOTO-0011-R6B-SM-4B65X | 2.45515206 | 1.2793779 | 3.3596699 | Normal |
| GTEX-RNOR-0011-R7A-SM-2TF4V | 2.2277925 | 1.3420639 | 1.8668948 | Normal |
| GTEX-X4XX-0011-R6B-SM-46MWP | 3.32641961 | 1.5596011 | 3.6628381 | Normal |
| GTEX-13O3Q-0011-R11b-SM-5KM2W | 1.5399073 | 0.2878792 | 1.4818899 | Normal |
| GTEX-145MH-0011-R1b-SM-5PNWY | 1.78153512 | 1.1516278 | 3.0611515 | Normal |
| GTEX-ZE7O-3126-SM-5HL5X | 2.97816704 | 1.7622239 | 3.5887175 | Normal |
| GTEX-13G51-3126-SM-5IJG7 | 1.99549926 | 0.3016187 | 2.1627231 | Normal |
| GTEX-1477Z-0011-R5b-SM-5PNUO | 4.50106872 | 2.9802833 | 4.7207091 | Normal |
| GTEX-RVPV-0011-R3A-SM-2TF63 | 2.38329034 | 1.3571027 | 3.103695 | Normal |
| GTEX-13FXS-3026-SM-5LZYO | 1.53941928 | 0.8862609 | 2.299092 | Normal |
| GTEX-ZDXO-0011-R10a-SM-4WWD8 | 2.35387347 | 1.7470986 | 3.0896884 | Normal |
| GTEX-144GO-0011-R6b-SM-5S2V7 | 3.22476488 | 0.9588306 | 3.9038209 | Normal |
| GTEX-13PLJ-0011-R3a-SM-5O9DT | 2.87632424 | 1.3203994 | 4.0288154 | Normal |
| GTEX-13QIC-2926-SM-5J2NF | 2.82709312 | 1.3147025 | 3.67342 | Normal |
| GTEX-145MI-0011-R8b-SM-5PNZ9 | 4.86145395 | 5.8664067 | 5.5060338 | Normal |
| GTEX-12ZZX-0011-R7b-SM-5DUVV | 2.81337448 | 0.87394 | 3.9987456 | Normal |
| GTEX-111FC-3326-SM-5GZYV | 2.44617362 | 1.2552837 | 2.4461736 | Normal |
| GTEX-Z93S-0011-R10a-SM-4RGNM | 2.59929012 | 0.3668411 | 2.1621508 | Normal |
| GTEX-YFC4-0011-R6b-SM-4V6E1 | 3.15944355 | 3.9013468 | 4.0079144 | Normal |
| GTEX-13OW7-0011-R11a-SM-5O9DY | 1.17851273 | 0.7617596 | 1.5033251 | Normal |
| GTEX-T2IS-0011-R11A-SM-32QPC | 1.2795622 | 0.5773162 | 1.39587 | Normal |
| GTEX-XOTO-0011-R5A-SM-4B657 | 2.92831433 | 2.06185 | 3.4835947 | Normal |
| GTEX-139T4-0011-R9b-SM-5HL6S | 3.62585243 | 4.0151821 | 4.2645127 | Normal |
| GTEX-13VXU-0011-R11b-SM-5O9DJ | 1.42363491 | 0.9795824 | 1.6408856 | Normal |
| GTEX-139TS-0011-R5b-SM-5J2MZ | 3.65956357 | 4.0568078 | 4.5969427 | Normal |
| GTEX-11OF3-0011-R5a-SM-57WB4 | 2.58543185 | 2.2531278 | 3.1273072 | Normal |
| GTEX-Q2AG-0011-R7A-SM-2HMJP | 2.50498593 | 0.8717817 | 3.2617089 | Normal |
| GTEX-13X6K-2826-SM-5O9DS | 2.10641748 | 0.3810126 | 3.2449969 | Normal |
| GTEX-1399T-0011-R1b-SM-5DUWN | 3.05726496 | 3.2209948 | 3.7712164 | Normal |
| GTEX-XLM4-2926-SM-4AT59 | 1.8263843 | 0.7459051 | 2.7669516 | Normal |
| GTEX-131XH-0011-R3b-SM-5DUWM | 2.17067476 | 0.9338085 | 2.8149183 | Normal |
| GTEX-1192X-3226-SM-5987D | 2.25116194 | 0.3506118 | 2.8290885 | Normal |
| GTEX-ZVZQ-0011-R5b-SM-57WDC | 3.03733877 | 2.1786312 | 3.6638436 | Normal |
| GTEX-13X6K-0011-R8b-SM-5PNZ4 | 3.29639326 | 1.9274716 | 3.6944527 | Normal |
| GTEX-12ZZZ-0011-R6b-SM-5EQ5B | 2.36832807 | 1.1967675 | 3.1068904 | Normal |
| GTEX-13G51-0011-R6b-SM-5LZX4 | 2.43508194 | 2.4002502 | 3.174265 | Normal |
| GTEX-YFC4-0011-R10a-SM-4SOK5 | 2.5187612 | 2.2395274 | 2.8178657 | Normal |
| GTEX-13112-0011-R4b-SM-5DUXL | 2.3958595 | 1.9260247 | 3.515197 | Normal |
| GTEX-1313W-0011-R1b-SM-5EQ4A | 2.02327278 | 2.23762 | 2.7479599 | Normal |
| GTEX-Q2AG-2826-SM-2HMJQ | 1.28486242 | 0.836395 | 2.1420544 | Normal |
| TCGA-06-0681-11A | 2.88938047 | 4.9027222 | 3.805034 | Normal |
| TCGA-06-AABW-11A | 2.81941572 | 4.1219627 | 4.1184821 | Normal |
| TCGA-06-0678-11A | 3.48948733 | 7.1023768 | 4.7089099 | Normal |
| TCGA-06-0675-11A | 3.78198568 | 2.5436493 | 3.9186543 | Normal |
| TCGA-06-0680-11A | 3.15630815 | 4.1348627 | 3.545204 | Normal |
| TCGA-06-0878-01A | 7.48594743 | 8.819736 | 6.2459494 | Tumor |
| TCGA-26-5135-01A | 4.72146459 | 5.7286228 | 6.2032885 | Tumor |
| TCGA-06-5859-01A | 3.38820827 | 5.6253556 | 5.9248149 | Tumor |
| TCGA-06-2563-01A | 5.65277442 | 3.609884 | 5.6049557 | Tumor |
| TCGA-41-2571-01A | 4.101182 | 3.535351 | 4.536125 | Tumor |
| TCGA-28-5207-01A | 3.78266233 | 2.1962309 | 4.9394941 | Tumor |
| TCGA-14-0871-01A | 4.10923343 | 3.3833542 | 4.7114034 | Tumor |
| TCGA-16-1045-01B | 7.04013871 | 5.3801751 | 6.2951941 | Tumor |
| TCGA-32-2632-01A | 6.55154064 | 5.5658783 | 6.1204902 | Tumor |
| TCGA-28-2514-01A | 4.136802 | 3.683813 | 5.013251 | Tumor |
| TCGA-06-0141-01A | 6.279208 | 6.381337 | 5.751161 | Tumor |
| TCGA-06-2569-01A | 4.083901 | 3.383957 | 4.955693 | Tumor |
| TCGA-02-2485-01A | 5.35939184 | 5.4426056 | 5.13061 | Tumor |
| TCGA-06-0138-01A | 4.7891016 | 7.020599 | 5.6748566 | Tumor |
| TCGA-06-2564-01A | 6.35366983 | 5.6884798 | 6.0296171 | Tumor |
| TCGA-16-0846-01A | 6.11772525 | 4.2990328 | 6.0309317 | Tumor |
| TCGA-76-4928-01B | 6.5000858 | 4.9360605 | 6.3969567 | Tumor |
| TCGA-76-4931-01A | 5.971276 | 3.348374 | 5.868116 | Tumor |
| TCGA-14-0817-01A | 5.69539141 | 5.7962418 | 5.7345841 | Tumor |
| TCGA-19-2620-01A | 5.96888604 | 4.0644101 | 5.939352 | Tumor |
| TCGA-26-5132-01A | 5.856762 | 5.277362 | 5.529896 | Tumor |
| TCGA-06-0125-02A | 7.35571888 | 5.7865153 | 6.2728701 | Tumor |
| TCGA-06-2558-01A | 4.346664 | 4.686393 | 4.990972 | Tumor |
| TCGA-06-5414-01A | 7.1457262 | 5.7133792 | 6.6586412 | Tumor |
| TCGA-26-5133-01A | 4.58939833 | 1.3087218 | 6.1191206 | Tumor |
| TCGA-32-4213-01A | 7.10610174 | 6.4716276 | 6.412951 | Tumor |
| TCGA-06-0210-01A | 6.445783 | 7.657547 | 6.503922 | Tumor |
| TCGA-41-2572-01A | 7.33900327 | 5.0474004 | 6.0969737 | Tumor |
| TCGA-06-5417-01A | 3.99451385 | 2.3088966 | 3.7727582 | Tumor |
| TCGA-19-2624-01A | 4.695431 | 4.408307 | 4.771338 | Tumor |
| TCGA-14-1823-01A | 7.58185008 | 8.4251686 | 6.8526013 | Tumor |
| TCGA-02-0055-01A | 7.52347584 | 7.2520813 | 6.1404171 | Tumor |
| TCGA-32-1982-01A | 5.71944979 | 4.6934352 | 5.991378 | Tumor |
| TCGA-06-2570-01A | 3.544581 | 4.205519 | 4.634431 | Tumor |
| TCGA-06-0745-01A | 4.818052 | 4.59458 | 5.686516 | Tumor |
| TCGA-14-1402-02A | 5.22331467 | 6.2382511 | 5.2940525 | Tumor |
| TCGA-14-0781-01B | 7.07781847 | 8.2569802 | 6.619993 | Tumor |
| TCGA-32-5222-01A | 5.1430373 | 4.1073303 | 5.5008338 | Tumor |
| TCGA-28-5204-01A | 5.95543723 | 3.8688812 | 5.7604844 | Tumor |
| TCGA-06-5410-01A | 7.22042923 | 7.737216 | 6.4829566 | Tumor |
| TCGA-14-1829-01A | 5.54032712 | 5.6865158 | 5.3671007 | Tumor |
| TCGA-02-0047-01A | 5.97863 | 5.024787 | 5.927196 | Tumor |
| TCGA-15-0742-01A | 5.61697994 | 3.4067289 | 5.9862154 | Tumor |
| TCGA-19-2619-01A | 5.721426 | 5.357116 | 6.458526 | Tumor |
| TCGA-06-0168-01A | 8.3362351 | 7.6936397 | 6.8614104 | Tumor |
| TCGA-19-1390-01A | 2.925781 | 1.506909 | 3.908846 | Tumor |
| TCGA-41-4097-01A | 7.070746 | 5.110414 | 6.182454 | Tumor |
| TCGA-06-0649-01B | 6.561861 | 5.44418 | 5.815693 | Tumor |
| TCGA-27-2526-01A | 6.19286566 | 4.8069589 | 6.2713409 | Tumor |
| TCGA-27-2523-01A | 4.87379177 | 3.832898 | 6.0256551 | Tumor |
| TCGA-06-5412-01A | 7.523476 | 6.63439 | 6.531596 | Tumor |
| TCGA-19-4065-01A | 6.921959 | 5.904722 | 6.050602 | Tumor |
| TCGA-06-0644-01A | 6.48854444 | 6.9944598 | 6.3605932 | Tumor |
| TCGA-12-0821-01A | 4.9522 | 6.191371 | 5.753236 | Tumor |
| TCGA-28-1753-01A | 6.0014793 | 5.0555221 | 5.8366007 | Tumor |
| TCGA-12-0619-01A | 6.965389 | 2.724935 | 6.198792 | Tumor |
| TCGA-14-0789-01A | 8.46977928 | 6.8087937 | 6.758192 | Tumor |
| TCGA-32-2634-01A | 5.054904 | 4.624274 | 5.208662 | Tumor |
| TCGA-06-0158-01A | 6.044053 | 5.333445 | 6.094233 | Tumor |
| TCGA-06-0152-02A | 6.407594 | 5.873709 | 6.185542 | Tumor |
| TCGA-12-0618-01A | 4.045417 | 2.068489 | 4.454791 | Tumor |
| TCGA-26-5139-01A | 6.73525415 | 6.553681 | 6.4981108 | Tumor |
| TCGA-06-0747-01A | 6.225882 | 3.306393 | 6.106617 | Tumor |
| TCGA-32-2616-01A | 5.018727 | 3.156028 | 6.648891 | Tumor |
| TCGA-06-0129-01A | 3.825921 | 2.450438 | 3.919332 | Tumor |
| TCGA-06-0125-01A | 6.458526 | 4.738308 | 6.064024 | Tumor |
| TCGA-06-0157-01A | 5.258571 | 5.264306 | 5.971276 | Tumor |
| TCGA-28-5220-01A | 3.78266233 | 5.1365054 | 5.1001195 | Tumor |
| TCGA-12-5295-01A | 5.26281249 | 4.2409837 | 5.7994402 | Tumor |
| TCGA-06-0645-01A | 7.04699779 | 7.8093854 | 6.4773112 | Tumor |
| TCGA-76-4929-01A | 5.80589828 | 4.2079217 | 5.6347706 | Tumor |
| TCGA-32-2615-01A | 6.599466 | 6.553681 | 6.073583 | Tumor |
| TCGA-32-1980-01A | 6.606013 | 5.583941 | 5.838828 | Tumor |
| TCGA-26-1442-01A | 5.134496 | 3.828668 | 5.582149 | Tumor |
| TCGA-06-0187-01A | 6.91599976 | 6.8555633 | 6.8827554 | Tumor |
| TCGA-14-0790-01B | 5.99651434 | 3.0000711 | 5.9467469 | Tumor |
| TCGA-14-2554-01A | 6.89180133 | 7.1569814 | 6.617686 | Tumor |
| TCGA-28-2510-01A | 4.926445 | 5.277362 | 5.076022 | Tumor |
| TCGA-06-2565-01A | 4.256514 | 5.379402 | 5.830981 | Tumor |
| TCGA-06-0156-01A | 7.131348 | 7.081223 | 5.758434 | Tumor |
| TCGA-14-1825-01A | 4.30073383 | 3.5891863 | 4.5724346 | Tumor |
| TCGA-12-1597-01B | 3.09440925 | 2.9560493 | 4.9845534 | Tumor |
| TCGA-19-4065-02A | 6.101046 | 6.811568 | 5.765721 | Tumor |
| TCGA-41-3915-01A | 7.601231 | 4.859479 | 6.486572 | Tumor |
| TCGA-06-2561-01A | 6.050602 | 6.355387 | 6.936819 | Tumor |
| TCGA-19-1787-01B | 5.678714 | 2.490334 | 6.537503 | Tumor |
| TCGA-27-1830-01A | 7.606213 | 6.841004 | 6.06133 | Tumor |
| TCGA-28-1747-01C | 7.13134841 | 4.869387 | 5.9307661 | Tumor |
| TCGA-14-1034-02B | 6.64641689 | 4.0858054 | 5.9130502 | Tumor |
| TCGA-28-2499-01A | 5.68651578 | 4.8793522 | 5.9504635 | Tumor |
| TCGA-06-2567-01A | 6.326611 | 7.704614 | 6.134719 | Tumor |
| TCGA-06-0686-01A | 4.68092464 | 4.5047189 | 5.230876 | Tumor |
| TCGA-27-2528-01A | 5.77903639 | 3.9182865 | 5.9418533 | Tumor |
| TCGA-06-0184-01A | 7.1457262 | 4.1647131 | 7.1205131 | Tumor |
| TCGA-06-0744-01A | 5.311703 | 3.612786 | 5.409962 | Tumor |
| TCGA-06-0171-02A | 8.053161 | 6.343359 | 6.661163 | Tumor |
| TCGA-14-0787-01A | 6.82898548 | 5.0493034 | 6.2918963 | Tumor |
| TCGA-06-0178-01A | 5.26281249 | 4.9685656 | 5.6974173 | Tumor |
| TCGA-06-2557-01A | 4.737809 | 5.470629 | 5.360156 | Tumor |
| TCGA-06-0211-02A | 6.98482907 | 7.6113263 | 6.3571988 | Tumor |
| TCGA-06-0210-02A | 6.671387 | 8.03338 | 6.400511 | Tumor |
| TCGA-76-4927-01A | 6.37426671 | 6.1389816 | 6.0348891 | Tumor |
| TCGA-06-5858-01A | 5.780057 | 3.335241 | 6.154721 | Tumor |
| TCGA-12-3653-01A | 6.61533961 | 2.3832903 | 5.9047224 | Tumor |
| TCGA-12-3650-01A | 4.19787978 | 2.6946253 | 5.3801751 | Tumor |
| TCGA-32-2638-01A | 7.284779 | 5.380175 | 6.636737 | Tumor |
| TCGA-12-3652-01A | 5.92603314 | 3.6573951 | 5.920067 | Tumor |
| TCGA-26-5134-01A | 4.77343111 | 4.779717 | 4.6232674 | Tumor |
| TCGA-27-2524-01A | 7.1096498 | 8.1926828 | 6.3742667 | Tumor |
| TCGA-06-5856-01A | 5.714348 | 4.52363 | 5.920067 | Tumor |
| TCGA-06-1804-01A | 4.120802 | 3.513295 | 5.432717 | Tumor |
| TCGA-26-5136-01B | 5.49157978 | 7.0173427 | 5.3364091 | Tumor |
| TCGA-06-0646-01A | 6.326611 | 7.737216 | 6.204816 | Tumor |
| TCGA-28-5218-01A | 8.34347075 | 6.5315963 | 6.7746714 | Tumor |
| TCGA-02-2483-01A | 5.1299576 | 2.9801517 | 4.7073747 | Tumor |
| TCGA-06-2562-01A | 6.28242455 | 7.4215532 | 6.7083436 | Tumor |
| TCGA-27-1837-01A | 6.4903996 | 5.0364379 | 6.1533055 | Tumor |
| TCGA-06-2559-01A | 5.637538 | 4.82072 | 5.722458 | Tumor |
| TCGA-06-0221-02A | 3.488248 | 1.8064 | 3.256837 | Tumor |
| TCGA-06-0211-01A | 7.1457262 | 6.0217541 | 6.7609114 | Tumor |
| TCGA-06-5416-01A | 3.51958 | 4.697354 | 4.940086 | Tumor |
| TCGA-06-0238-01A | 4.191518 | 4.289062 | 4.915718 | Tumor |
| TCGA-14-1034-01A | 7.27232347 | 6.4221624 | 6.3761193 | Tumor |
| TCGA-19-2629-01A | 5.5043269 | 3.9095895 | 5.2708144 | Tumor |
| TCGA-19-2625-01A | 6.97859 | 5.278088 | 6.460448 | Tumor |
| TCGA-06-5413-01A | 5.61968409 | 5.6603356 | 5.5517111 | Tumor |
| TCGA-12-5299-01A | 6.36566636 | 4.4552202 | 6.4385543 | Tumor |
| TCGA-06-0882-01A | 5.97373414 | 6.6945239 | 6.1794561 | Tumor |
| TCGA-06-0130-01A | 6.749408 | 8.609189 | 6.224395 | Tumor |
| TCGA-76-4925-01A | 5.08931 | 5.326657 | 6.121884 | Tumor |
| TCGA-06-5418-01A | 6.72428253 | 6.2459494 | 6.112181 | Tumor |
| TCGA-06-0190-01A | 6.86725916 | 8.026568 | 6.4548892 | Tumor |
| TCGA-27-1835-01A | 4.35739346 | 2.5605821 | 5.1260282 | Tumor |
| TCGA-28-5216-01A | 5.72655548 | 5.380922 | 5.4706288 | Tumor |
| TCGA-06-5411-01A | 4.145796 | 5.011393 | 4.747539 | Tumor |
| TCGA-27-2519-01A | 6.33669074 | 7.4446218 | 6.1943343 | Tumor |
| TCGA-28-5213-01A | 6.90084431 | 7.6575475 | 6.0115135 | Tumor |
| TCGA-28-5209-01A | 6.56590763 | 3.810208 | 6.2412975 | Tumor |
| TCGA-76-4926-01B | 6.144733 | 4.904959 | 5.860134 | Tumor |
| TCGA-06-0219-01A | 4.52547309 | 6.8175386 | 5.4319201 | Tumor |
| TCGA-02-2486-01A | 8.06004094 | 8.753705 | 6.1928657 | Tumor |
| TCGA-06-0190-02A | 8.29331368 | 6.7609114 | 6.4257833 | Tumor |
| TCGA-06-0750-01A | 7.63148432 | 6.5618608 | 6.3690474 | Tumor |
| TCGA-15-1444-01A | 5.060426 | 4.128208 | 5.242116 | Tumor |
| TCGA-27-1832-01A | 8.365622 | 7.64699 | 6.456733 | Tumor |
| TCGA-19-0957-02A | 5.990096 | 4.798018 | 5.232286 | Tumor |
| TCGA-06-0174-01A | 3.92900948 | 4.2479353 | 5.3425359 | Tumor |
| TCGA-12-0616-01A | 5.69837414 | 4.270883 | 5.4180743 | Tumor |
| TCGA-27-1834-01A | 5.426207 | 4.498846 | 5.551711 | Tumor |
| TCGA-32-1970-01A | 5.394124 | 1.85022 | 5.683543 | Tumor |
| TCGA-14-0736-02A | 5.61790579 | 6.7718808 | 5.8134948 | Tumor |
| TCGA-28-5215-01A | 6.191371 | 5.028437 | 5.648996 | Tumor |
| TCGA-06-0132-01A | 5.79189005 | 6.2107155 | 5.8123941 | Tumor |
| TCGA-06-5408-01A | 7.03358813 | 5.8455495 | 5.9977517 | Tumor |
| TCGA-28-5208-01A | 6.18841637 | 5.1046064 | 6.0653823 | Tumor |
| TCGA-06-0211-01B | 5.75116132 | 2.9500747 | 6.102421 | Tumor |
| TCGA-28-2509-01A | 6.18993983 | 4.9315061 | 6.6343902 | Tumor |
| TCGA-19-5960-01A | 3.158309 | 1.307292 | 4.641692 | Tumor |
| TCGA-41-5651-01A | 3.800507 | 1.926025 | 4.055265 | Tumor |
| TCGA-27-2521-01A | 3.73653558 | 4.0803943 | 5.6479857 | Tumor |
| TCGA-28-2513-01A | 7.83216967 | 6.7609114 | 6.7637697 | Tumor |
| TCGA-06-0743-01A | 4.950468 | 5.918904 | 5.655574 | Tumor |
| TCGA-76-4932-01A | 5.95543723 | 3.810208 | 6.2243947 | Tumor |
| TCGA-08-0386-01A | 3.823484 | 3.835726 | 5.077958 | Tumor |
| TCGA-06-0749-01A | 5.39175093 | 4.7311987 | 5.3663281 | Tumor |
| TCGA-27-1831-01A | 5.81569331 | 7.1569814 | 5.6518471 | Tumor |
| TCGA-06-0139-01A | 7.16060739 | 7.9999906 | 6.3350749 | Tumor |
| TCGA-19-1389-02A | 9.10285352 | 8.6013737 | 6.7024779 | Tumor |

**Supplementary Table S4.** The 62 nodes interacting with C1R, CCL2 and TNFRSF1A.

| node1 | node2 | combined_score |  |
| --- | --- | --- | --- |
| A2M | CCL2 | 0.405 |  |
| A2M | VCAM1 | 0.406 |  |
| A2M | CXCL8 | 0.764 |  |
| A2M | RBP4 | 0.431 |  |
| A2M | SAA1 | 0.461 |  |
| A2M | PLAU | 0.507 |  |
| A2M | AGT | 0.525 |  |
| A2M | C1R | 0.577 |  |
| A2M | MMP9 | 0.862 |  |
| ACKR3 | CCL2 | 0.414 |  |
| ACKR3 | TAC1 | 0.412 |  |
| ACKR3 | CXCL10 | 0.422 |  |
| ACKR3 | CXCL8 | 0.45 |  |
| ACKR3 | EGFR | 0.72 |  |
| ACKR3 | CXCR4 | 0.967 |  |
| AGT | NAMPT | 0.459 |  |
| AGT | CCL2 | 0.498 |  |
| AGT | C3 | 0.419 |  |
| AGT | VCAM1 | 0.508 |  |
| AGT | CASP3 | 0.411 |  |
| AGT | TAC1 | 0.523 |  |
| AGT | F2R | 0.803 |  |
| AGT | JUN | 0.413 |  |
| AGT | SPP1 | 0.438 |  |
| AGT | RHOA | 0.447 |  |
| AGT | MMP9 | 0.507 |  |
| AGT | GFAP | 0.527 |  |
| AGT | CTGF | 0.589 |  |
| AGT | SAA1 | 0.592 |  |
| AGT | RBP4 | 0.61 |  |
| AGT | CYBB | 0.647 |  |
| AGT | CXCR4 | 0.911 |  |
| C1R | C3 | 0.822 |  |
| C1R | PTX3 | 0.488 |  |
| C1R | C3AR1 | 0.582 |  |
| C1R | CALR | 0.429 |  |
| C1R | C5AR1 | 0.614 |  |
| C1R | HLA-A | 0.472 |  |
| C3 | CD4 | 0.609 |  |
| C3 | CCL2 | 0.529 |  |
| C3 | CXCL10 | 0.401 |  |
| C3 | MMP9 | 0.407 |  |
| C3 | SAA1 | 0.419 |  |
| C3 | VCAM1 | 0.42 |  |
| C3 | FCGR3A | 0.46 |  |
| C3 | RBP4 | 0.46 |  |
| C3 | TLR2 | 0.538 |  |
| C3 | CXCL8 | 0.59 |  |
| C3 | PTX3 | 0.799 |  |
| C3 | C5AR1 | 0.935 |  |
| C3 | ITGB2 | 0.968 |  |
| C3 | C3AR1 | 0.997 |  |
| C3AR1 | CD4 | 0.588 |  |
| C3AR1 | CCL2 | 0.436 |  |
| C3AR1 | TLR2 | 0.581 |  |
| C3AR1 | MSR1 | 0.414 |  |
| C3AR1 | TYROBP | 0.675 |  |
| C3AR1 | CSF1R | 0.748 |  |
| C3AR1 | VCAM1 | 0.536 |  |
| C3AR1 | CCR1 | 0.637 |  |
| C3AR1 | CYBB | 0.491 |  |
| C3AR1 | FCGR3A | 0.508 |  |
| C3AR1 | CD14 | 0.575 |  |
| C3AR1 | CD86 | 0.598 |  |
| C3AR1 | C5AR1 | 0.706 |  |
| C3AR1 | ITGB2 | 0.742 |  |
| C5AR1 | CD4 | 0.559 |  |
| C5AR1 | CCL2 | 0.51 |  |
| C5AR1 | TLR2 | 0.987 |  |
| C5AR1 | TYROBP | 0.5 |  |
| C5AR1 | CSF1R | 0.523 |  |
| C5AR1 | VCAM1 | 0.581 |  |
| C5AR1 | CXCL8 | 0.571 |  |
| C5AR1 | CD86 | 0.487 |  |
| C5AR1 | TNFSF13B | 0.418 |  |
| C5AR1 | FCGR3A | 0.49 |  |
| C5AR1 | ITGB2 | 0.616 |  |
| CALR | CD4 | 0.559 |  |
| CALR | GRN | 0.434 |  |
| CALR | TLR2 | 0.41 |  |
| CALR | MSR1 | 0.642 |  |
| CALR | EGFR | 0.45 |  |
| CALR | CASP3 | 0.5 |  |
| CALR | JUN | 0.44 |  |
| CALR | CD86 | 0.459 |  |
| CALR | HSPA1A | 0.591 |  |
| CALR | MMP9 | 0.803 |  |
| CALR | HLA-A | 0.99 |  |
| CASP3 | CD4 | 0.677 |  |
| CASP3 | TNFRSF1A | 0.705 |  |
| CASP3 | HMOX1 | 0.762 |  |
| CASP3 | CCL2 | 0.639 |  |
| CASP3 | TLR2 | 0.545 |  |
| CASP3 | EGFR | 0.759 |  |
| CASP3 | VCAM1 | 0.554 |  |
| CASP3 | TNFRSF21 | 0.426 |  |
| CASP3 | CXCL10 | 0.469 |  |
| CASP3 | CXCL8 | 0.668 |  |
| CASP3 | TNFRSF1B | 0.404 |  |
| CASP3 | CD86 | 0.409 |  |
| CASP3 | SOCS3 | 0.416 |  |
| CASP3 | SDC1 | 0.473 |  |
| CASP3 | CTGF | 0.478 |  |
| CASP3 | FGF13 | 0.51 |  |
| CASP3 | CXCR4 | 0.557 |  |
| CASP3 | SPP1 | 0.588 |  |
| CASP3 | CYBB | 0.615 |  |
| CASP3 | HSPA1A | 0.644 |  |
| CASP3 | STAT1 | 0.701 |  |
| CASP3 | GFAP | 0.739 |  |
| CASP3 | MMP9 | 0.772 |  |
| CASP3 | JUN | 0.837 |  |
| CASP3 | TNFRSF25 | 0.943 |  |
| CASP3 | RHOA | 0.957 |  |
| CCL2 | CD74 | 0.415 |  |
| CCL2 | CD4 | 0.816 |  |
| CCL2 | TNFRSF1A | 0.63 |  |
| CCL2 | HMOX1 | 0.686 |  |
| CCL2 | PROCR | 0.548 |  |
| CCL2 | NAMPT | 0.598 |  |
| CCL2 | GDF15 | 0.401 |  |
| CCL2 | S100A9 | 0.404 |  |
| CCL2 | TYROBP | 0.408 |  |
| CCL2 | CD14 | 0.416 |  |
| CCL2 | F2R | 0.459 |  |
| CCL2 | RBP4 | 0.469 |  |
| CCL2 | TAC1 | 0.469 |  |
| CCL2 | ITGB2 | 0.469 |  |
| CCL2 | RHOA | 0.475 |  |
| CCL2 | MSR1 | 0.481 |  |
| CCL2 | SYK | 0.486 |  |
| CCL2 | ISG15 | 0.489 |  |
| CCL2 | SDC1 | 0.489 |  |
| CCL2 | TNFRSF12A | 0.493 |  |
| CCL2 | TNFSF13B | 0.505 |  |
| CCL2 | PLAU | 0.519 |  |
| CCL2 | EGFR | 0.527 |  |
| CCL2 | SAA1 | 0.539 |  |
| CCL2 | FGF13 | 0.563 |  |
| CCL2 | TNFRSF1B | 0.563 |  |
| CCL2 | PTX3 | 0.57 |  |
| CCL2 | OLR1 | 0.578 |  |
| CCL2 | GFAP | 0.581 |  |
| CCL2 | SOCS3 | 0.603 |  |
| CCL2 | CYBB | 0.609 |  |
| CCL2 | CTGF | 0.64 |  |
| CCL2 | CSF1R | 0.653 |  |
| CCL2 | STAT1 | 0.672 |  |
| CCL2 | CXCL16 | 0.684 |  |
| CCL2 | CD86 | 0.685 |  |
| CCL2 | SPP1 | 0.69 |  |
| CCL2 | FCGR3A | 0.69 |  |
| CCL2 | TLR2 | 0.776 |  |
| CCL2 | MMP9 | 0.787 |  |
| CCL2 | CSF1 | 0.789 |  |
| CCL2 | VCAM1 | 0.899 |  |
| CCL2 | CXCR4 | 0.921 |  |
| CCL2 | CX3CR1 | 0.941 |  |
| CCL2 | LIF | 0.951 |  |
| CCL2 | JUN | 0.968 |  |
| CCL2 | CCR1 | 0.987 |  |
| CCL2 | CXCL10 | 0.991 |  |
| CCL2 | CXCL8 | 0.995 |  |
| CCR1 | CD74 | 0.46 |  |
| CCR1 | CD4 | 0.838 |  |
| CCR1 | TLR2 | 0.588 |  |
| CCR1 | TYROBP | 0.622 |  |
| CCR1 | CSF1R | 0.609 |  |
| CCR1 | CXCL16 | 0.802 |  |
| CCR1 | VCAM1 | 0.523 |  |
| CCR1 | S100A9 | 0.432 |  |
| CCR1 | CD14 | 0.446 |  |
| CCR1 | CSF1 | 0.465 |  |
| CCR1 | STAT1 | 0.486 |  |
| CCR1 | FCGR3A | 0.593 |  |
| CCR1 | CYBB | 0.594 |  |
| CCR1 | MMP9 | 0.595 |  |
| CCR1 | ITGB2 | 0.607 |  |
| CCR1 | CD86 | 0.699 |  |
| CCR1 | CXCL8 | 0.859 |  |
| CCR1 | CXCL10 | 0.911 |  |
| CCR1 | TNFRSF1B | 0.941 |  |
| CD14 | CD4 | 0.644 |  |
| CD14 | TLR2 | 0.997 |  |
| CD14 | TYROBP | 0.849 |  |
| CD14 | CSF1R | 0.851 |  |
| CD14 | TNFRSF1B | 0.401 |  |
| CD14 | CYBB | 0.406 |  |
| CD14 | MMP9 | 0.417 |  |
| CD14 | CXCL10 | 0.418 |  |
| CD14 | HSPA1A | 0.43 |  |
| CD14 | CX3CR1 | 0.444 |  |
| CD14 | CXCL8 | 0.457 |  |
| CD14 | CSF1 | 0.48 |  |
| CD14 | S100A9 | 0.607 |  |
| CD14 | CD86 | 0.677 |  |
| CD14 | FCGR3A | 0.772 |  |
| CD14 | ITGB2 | 0.899 |  |
| CD4 | CD74 | 0.632 |  |
| CD4 | RHOA | 0.412 |  |
| CD4 | MSR1 | 0.424 |  |
| CD4 | GFAP | 0.45 |  |
| CD4 | ISG15 | 0.47 |  |
| CD4 | CXCL16 | 0.476 |  |
| CD4 | S100A9 | 0.479 |  |
| CD4 | HMOX1 | 0.514 |  |
| CD4 | SPP1 | 0.521 |  |
| CD4 | EGFR | 0.567 |  |
| CD4 | CYBB | 0.572 |  |
| CD4 | SOCS3 | 0.609 |  |
| CD4 | TNFRSF1B | 0.622 |  |
| CD4 | TYROBP | 0.649 |  |
| CD4 | MMP9 | 0.66 |  |
| CD4 | TNFRSF25 | 0.661 |  |
| CD4 | ITGB2 | 0.679 |  |
| CD4 | JUN | 0.691 |  |
| CD4 | HLA-A | 0.696 |  |
| CD4 | TNFRSF1A | 0.697 |  |
| CD4 | TNFSF13B | 0.71 |  |
| CD4 | CSF1R | 0.718 |  |
| CD4 | CSF1 | 0.719 |  |
| CD4 | SDC1 | 0.736 |  |
| CD4 | CX3CR1 | 0.736 |  |
| CD4 | VCAM1 | 0.738 |  |
| CD4 | STAT1 | 0.766 |  |
| CD4 | TLR2 | 0.808 |  |
| CD4 | CXCL10 | 0.825 |  |
| CD4 | CXCL8 | 0.832 |  |
| CD4 | FCGR3A | 0.937 |  |
| CD4 | SYK | 0.975 |  |
| CD4 | CD86 | 0.995 |  |
| CD4 | CXCR4 | 0.999 |  |
| CD74 | HSPA1A | 0.401 |  |
| CD74 | VCAM1 | 0.405 |  |
| CD74 | CX3CR1 | 0.407 |  |
| CD74 | CXCL8 | 0.427 |  |
| CD74 | TLR2 | 0.428 |  |
| CD74 | CXCL10 | 0.439 |  |
| CD74 | EGFR | 0.456 |  |
| CD74 | FCGR3A | 0.498 |  |
| CD74 | CYBB | 0.5 |  |
| CD74 | HLA-A | 0.612 |  |
| CD74 | CSF1R | 0.613 |  |
| CD74 | CD86 | 0.643 |  |
| CD74 | TYROBP | 0.699 |  |
| CD74 | ITGB2 | 0.711 |  |
| CD74 | CXCR4 | 0.991 |  |
| CD86 | TNFRSF1A | 0.507 |  |
| CD86 | TLR2 | 0.808 |  |
| CD86 | MSR1 | 0.547 |  |
| CD86 | TYROBP | 0.703 |  |
| CD86 | CSF1R | 0.731 |  |
| CD86 | VCAM1 | 0.557 |  |
| CD86 | CXCL10 | 0.726 |  |
| CD86 | CXCL8 | 0.705 |  |
| CD86 | CSF1 | 0.96 |  |
| CD86 | SOCS3 | 0.48 |  |
| CD86 | JUN | 0.411 |  |
| CD86 | S100A9 | 0.428 |  |
| CD86 | TNFRSF1B | 0.454 |  |
| CD86 | MMP9 | 0.508 |  |
| CD86 | STAT1 | 0.603 |  |
| CD86 | SYK | 0.603 |  |
| CD86 | SDC1 | 0.628 |  |
| CD86 | CX3CR1 | 0.639 |  |
| CD86 | CXCR4 | 0.642 |  |
| CD86 | TNFSF13B | 0.655 |  |
| CD86 | ITGB2 | 0.657 |  |
| CD86 | CYBB | 0.657 |  |
| CD86 | FCGR3A | 0.829 |  |
| CD86 | HLA-A | 0.951 |  |
| CSF1 | TNFRSF1A | 0.442 |  |
| CSF1 | LIF | 0.497 |  |
| CSF1 | TLR2 | 0.579 |  |
| CSF1 | MSR1 | 0.503 |  |
| CSF1 | TYROBP | 0.514 |  |
| CSF1 | EGFR | 0.83 |  |
| CSF1 | CSF1R | 0.999 |  |
| CSF1 | VCAM1 | 0.551 |  |
| CSF1 | CXCL10 | 0.683 |  |
| CSF1 | CXCL8 | 0.744 |  |
| CSF1 | TNFRSF1B | 0.404 |  |
| CSF1 | PLAU | 0.416 |  |
| CSF1 | SOCS3 | 0.449 |  |
| CSF1 | JUN | 0.492 |  |
| CSF1 | SPP1 | 0.522 |  |
| CSF1 | FCGR3A | 0.523 |  |
| CSF1 | SYK | 0.558 |  |
| CSF1 | STAT1 | 0.568 |  |
| CSF1 | MMP9 | 0.615 |  |
| CSF1 | CX3CR1 | 0.625 |  |
| CSF1 | CXCR4 | 0.649 |  |
| CSF1R | TLR2 | 0.649 |  |
| CSF1R | MSR1 | 0.541 |  |
| CSF1R | TYROBP | 0.995 |  |
| CSF1R | EGFR | 0.419 |  |
| CSF1R | SYK | 0.413 |  |
| CSF1R | JUN | 0.421 |  |
| CSF1R | S100A9 | 0.425 |  |
| CSF1R | SOCS3 | 0.455 |  |
| CSF1R | STAT1 | 0.462 |  |
| CSF1R | CXCL8 | 0.473 |  |
| CSF1R | CXCL10 | 0.497 |  |
| CSF1R | CXCR4 | 0.537 |  |
| CSF1R | MMP9 | 0.572 |  |
| CSF1R | CYBB | 0.696 |  |
| CSF1R | VCAM1 | 0.698 |  |
| CSF1R | SDC1 | 0.703 |  |
| CSF1R | CX3CR1 | 0.799 |  |
| CSF1R | FCGR3A | 0.803 |  |
| CSF1R | ITGB2 | 0.839 |  |
| CTGF | HMOX1 | 0.407 |  |
| CTGF | GDF15 | 0.557 |  |
| CTGF | EGFR | 0.827 |  |
| CTGF | VCAM1 | 0.508 |  |
| CTGF | CXCL8 | 0.556 |  |
| CTGF | FGF13 | 0.517 |  |
| CTGF | PLAU | 0.426 |  |
| CTGF | CYBB | 0.439 |  |
| CTGF | CXCR4 | 0.47 |  |
| CTGF | RHOA | 0.568 |  |
| CTGF | JUN | 0.582 |  |
| CTGF | SPP1 | 0.624 |  |
| CTGF | MMP9 | 0.698 |  |
| CTGF | ITGB2 | 0.923 |  |
| CX3CR1 | TLR2 | 0.585 |  |
| CX3CR1 | TYROBP | 0.622 |  |
| CX3CR1 | CXCL16 | 0.827 |  |
| CX3CR1 | VCAM1 | 0.508 |  |
| CX3CR1 | CXCL10 | 0.835 |  |
| CX3CR1 | CXCL8 | 0.815 |  |
| CX3CR1 | MMP9 | 0.462 |  |
| CX3CR1 | ITGB2 | 0.484 |  |
| CX3CR1 | CYBB | 0.505 |  |
| CX3CR1 | GFAP | 0.588 |  |
| CX3CR1 | FCGR3A | 0.754 |  |
| CXCL10 | TNFRSF1A | 0.528 |  |
| CXCL10 | HMOX1 | 0.487 |  |
| CXCL10 | LIF | 0.94 |  |
| CXCL10 | TLR2 | 0.739 |  |
| CXCL10 | CXCL16 | 0.663 |  |
| CXCL10 | VCAM1 | 0.74 |  |
| CXCL10 | CYBB | 0.406 |  |
| CXCL10 | GFAP | 0.437 |  |
| CXCL10 | SAA1 | 0.447 |  |
| CXCL10 | FGF13 | 0.459 |  |
| CXCL10 | SPP1 | 0.469 |  |
| CXCL10 | TNFRSF1B | 0.508 |  |
| CXCL10 | JUN | 0.514 |  |
| CXCL10 | SOCS3 | 0.536 |  |
| CXCL10 | TNFSF13B | 0.611 |  |
| CXCL10 | FCGR3A | 0.633 |  |
| CXCL10 | MMP9 | 0.651 |  |
| CXCL10 | ISG15 | 0.811 |  |
| CXCL10 | CXCR4 | 0.875 |  |
| CXCL10 | STAT1 | 0.922 |  |
| CXCL10 | CXCL8 | 0.955 |  |
| CXCL16 | TLR2 | 0.428 |  |
| CXCL16 | MSR1 | 0.422 |  |
| CXCL16 | MMP9 | 0.414 |  |
| CXCL16 | VCAM1 | 0.428 |  |
| CXCL16 | OLR1 | 0.495 |  |
| CXCL16 | CXCL8 | 0.58 |  |
| CXCL16 | CXCR4 | 0.829 |  |
| CXCL8 | GRN | 0.401 |  |
| CXCL8 | TNFRSF1A | 0.728 |  |
| CXCL8 | HMOX1 | 0.673 |  |
| CXCL8 | NAMPT | 0.618 |  |
| CXCL8 | LIF | 0.956 |  |
| CXCL8 | GDF15 | 0.446 |  |
| CXCL8 | TLR2 | 0.861 |  |
| CXCL8 | EGFR | 0.654 |  |
| CXCL8 | VCAM1 | 0.821 |  |
| CXCL8 | PTX3 | 0.586 |  |
| CXCL8 | TNFRSF12A | 0.411 |  |
| CXCL8 | HSPA1A | 0.433 |  |
| CXCL8 | OLR1 | 0.443 |  |
| CXCL8 | ISG15 | 0.471 |  |
| CXCL8 | SYK | 0.501 |  |
| CXCL8 | RHOA | 0.502 |  |
| CXCL8 | CYBB | 0.52 |  |
| CXCL8 | TNFSF13B | 0.53 |  |
| CXCL8 | S100A9 | 0.532 |  |
| CXCL8 | ITGB2 | 0.535 |  |
| CXCL8 | TAC1 | 0.563 |  |
| CXCL8 | TNFRSF1B | 0.577 |  |
| CXCL8 | PLAU | 0.597 |  |
| CXCL8 | FGF13 | 0.608 |  |
| CXCL8 | FCGR3A | 0.614 |  |
| CXCL8 | SOCS3 | 0.618 |  |
| CXCL8 | SPP1 | 0.622 |  |
| CXCL8 | SAA1 | 0.628 |  |
| CXCL8 | STAT1 | 0.679 |  |
| CXCL8 | F2R | 0.824 |  |
| CXCL8 | SDC1 | 0.843 |  |
| CXCL8 | MMP9 | 0.871 |  |
| CXCL8 | CXCR4 | 0.913 |  |
| CXCL8 | JUN | 0.957 |  |
| CXCR4 | TNFRSF1A | 0.432 |  |
| CXCR4 | LIF | 0.412 |  |
| CXCR4 | TLR2 | 0.976 |  |
| CXCR4 | EGFR | 0.914 |  |
| CXCR4 | VCAM1 | 0.751 |  |
| CXCR4 | FGF13 | 0.511 |  |
| CXCR4 | SOCS3 | 0.631 |  |
| CXCR4 | STAT1 | 0.963 |  |
| CXCR4 | FCGR3A | 0.59 |  |
| CXCR4 | JUN | 0.501 |  |
| CXCR4 | MMP9 | 0.723 |  |
| CXCR4 | PLAU | 0.45 |  |
| CXCR4 | SYK | 0.468 |  |
| CXCR4 | TNFSF13B | 0.468 |  |
| CXCR4 | SDC1 | 0.559 |  |
| CXCR4 | SPP1 | 0.715 |  |
| CXCR4 | ITGB2 | 0.547 |  |
| CXCR4 | GFAP | 0.468 |  |
| CXCR4 | RHOA | 0.51 |  |
| CYBB | TNFRSF1A | 0.495 |  |
| CYBB | HMOX1 | 0.687 |  |
| CYBB | NAMPT | 0.842 |  |
| CYBB | TLR2 | 0.946 |  |
| CYBB | TYROBP | 0.621 |  |
| CYBB | EGFR | 0.684 |  |
| CYBB | VCAM1 | 0.956 |  |
| CYBB | OLR1 | 0.578 |  |
| CYBB | TAC1 | 0.407 |  |
| CYBB | STAT1 | 0.481 |  |
| CYBB | FCGR3A | 0.539 |  |
| CYBB | S100A9 | 0.761 |  |
| CYBB | JUN | 0.509 |  |
| CYBB | MMP9 | 0.86 |  |
| CYBB | SYK | 0.534 |  |
| CYBB | RHOA | 0.446 |  |
| CYBB | ITGB2 | 0.695 |  |
| EGFR | TNFRSF1A | 0.847 |  |
| EGFR | LIF | 0.735 |  |
| EGFR | GDF15 | 0.412 |  |
| EGFR | TLR2 | 0.833 |  |
| EGFR | HLA-A | 0.426 |  |
| EGFR | ISG15 | 0.47 |  |
| EGFR | VCAM1 | 0.472 |  |
| EGFR | SYK | 0.526 |  |
| EGFR | GFAP | 0.563 |  |
| EGFR | FGF13 | 0.583 |  |
| EGFR | SPP1 | 0.6 |  |
| EGFR | ITGB2 | 0.731 |  |
| EGFR | MMP9 | 0.733 |  |
| EGFR | SDC1 | 0.737 |  |
| EGFR | JUN | 0.764 |  |
| EGFR | HSPA1A | 0.798 |  |
| EGFR | SOCS3 | 0.863 |  |
| EGFR | PLAU | 0.962 |  |
| EGFR | RHOA | 0.968 |  |
| EGFR | STAT1 | 0.996 |  |
| F2R | PROCR | 0.977 |  |
| F2R | SYK | 0.448 |  |
| F2R | MMP9 | 0.459 |  |
| F2R | RHOA | 0.834 |  |
| FCGR3A | TNFRSF1A | 0.404 |  |
| FCGR3A | TLR2 | 0.799 |  |
| FCGR3A | MSR1 | 0.424 |  |
| FCGR3A | TYROBP | 0.894 |  |
| FCGR3A | VCAM1 | 0.437 |  |
| FCGR3A | STAT1 | 0.448 |  |
| FCGR3A | TNFRSF1B | 0.418 |  |
| FCGR3A | MMP9 | 0.449 |  |
| FCGR3A | TNFSF13B | 0.49 |  |
| FCGR3A | SDC1 | 0.519 |  |
| FCGR3A | HLA-A | 0.536 |  |
| FCGR3A | S100A9 | 0.693 |  |
| FCGR3A | ITGB2 | 0.742 |  |
| FCGR3A | SYK | 0.963 |  |
| FGF13 | LIF | 0.585 |  |
| FGF13 | VCAM1 | 0.407 |  |
| FGF13 | SDC1 | 0.452 |  |
| FGF13 | JUN | 0.477 |  |
| FGF13 | TNFRSF12A | 0.504 |  |
| FGF13 | SPP1 | 0.507 |  |
| FGF13 | MMP9 | 0.584 |  |
| FGF13 | GFAP | 0.619 |  |
| GDF15 | PTX3 | 0.444 |  |
| GDF15 | SPP1 | 0.468 |  |
| GFAP | HMOX1 | 0.51 |  |
| GFAP | LIF | 0.509 |  |
| GFAP | TAC1 | 0.446 |  |
| GFAP | JUN | 0.432 |  |
| GFAP | MMP9 | 0.471 |  |
| GRN | TYROBP | 0.439 |  |
| GRN | TNFRSF25 | 0.799 |  |
| GRN | TNFRSF1B | 0.897 |  |
| GRN | TNFRSF1A | 0.993 |  |
| HLA-A | TYROBP | 0.433 |  |
| HLA-A | STAT1 | 0.937 |  |
| HLA-A | JUN | 0.912 |  |
| HLA-A | TNFRSF25 | 0.747 |  |
| HLA-A | ISG15 | 0.916 |  |
| HMOX1 | TNFRSF1A | 0.502 |  |
| HMOX1 | STAT1 | 0.455 |  |
| HMOX1 | SPP1 | 0.505 |  |
| HMOX1 | HSPA1A | 0.523 |  |
| HMOX1 | TLR2 | 0.598 |  |
| HMOX1 | VCAM1 | 0.624 |  |
| HMOX1 | MMP9 | 0.637 |  |
| HMOX1 | JUN | 0.967 |  |
| HSPA1A | TLR2 | 0.985 |  |
| HSPA1A | OLR1 | 0.603 |  |
| HSPA1A | JUN | 0.547 |  |
| HSPA1A | RHOA | 0.571 |  |
| ISG15 | TLR2 | 0.442 |  |
| ISG15 | SOCS3 | 0.411 |  |
| ISG15 | STAT1 | 0.985 |  |
| ITGB2 | TLR2 | 0.719 |  |
| ITGB2 | TYROBP | 0.915 |  |
| ITGB2 | VCAM1 | 0.996 |  |
| ITGB2 | S100A9 | 0.59 |  |
| ITGB2 | MMP9 | 0.955 |  |
| ITGB2 | PLAU | 0.92 |  |
| ITGB2 | SYK | 0.804 |  |
| ITGB2 | TNFRSF1B | 0.436 |  |
| ITGB2 | RHOA | 0.945 |  |
| JUN | TNFRSF1A | 0.626 |  |
| JUN | TLR2 | 0.628 |  |
| JUN | VCAM1 | 0.565 |  |
| JUN | SOCS3 | 0.593 |  |
| JUN | STAT1 | 0.839 |  |
| JUN | PLAU | 0.502 |  |
| JUN | SPP1 | 0.521 |  |
| JUN | RHOA | 0.692 |  |
| JUN | MMP9 | 0.849 |  |
| JUN | SYK | 0.951 |  |
| LIF | STAT1 | 0.523 |  |
| LIF | SOCS3 | 0.595 |  |
| LIF | SAA1 | 0.905 |  |
| LIF | MMP9 | 0.932 |  |
| MMP9 | TNFRSF1A | 0.533 |  |
| MMP9 | TLR2 | 0.775 |  |
| MMP9 | TYROBP | 0.472 |  |
| MMP9 | VCAM1 | 0.728 |  |
| MMP9 | PTX3 | 0.426 |  |
| MMP9 | OLR1 | 0.455 |  |
| MMP9 | TAC1 | 0.448 |  |
| MMP9 | SOCS3 | 0.546 |  |
| MMP9 | STAT1 | 0.537 |  |
| MMP9 | S100A9 | 0.528 |  |
| MMP9 | TNFSF13B | 0.408 |  |
| MMP9 | TNFRSF1B | 0.447 |  |
| MMP9 | RHOA | 0.614 |  |
| MMP9 | SPP1 | 0.717 |  |
| MMP9 | PLAU | 0.738 |  |
| MMP9 | SAA1 | 0.94 |  |
| MMP9 | SDC1 | 0.984 |  |
| MSR1 | TLR2 | 0.52 |  |
| MSR1 | SAA1 | 0.565 |  |
| MSR1 | OLR1 | 0.657 |  |
| NAMPT | RBP4 | 0.732 |  |
| OLR1 | TLR2 | 0.704 |  |
| OLR1 | VCAM1 | 0.623 |  |
| OLR1 | SAA1 | 0.435 |  |
| PLAU | VCAM1 | 0.404 |  |
| PLAU | RHOA | 0.425 |  |
| PLAU | SPP1 | 0.485 |  |
| PROCR | VCAM1 | 0.466 |  |
| PTX3 | TLR2 | 0.415 |  |
| PTX3 | VCAM1 | 0.422 |  |
| PTX3 | SPP1 | 0.404 |  |
| RHOA | TNFRSF1A | 0.448 |  |
| RHOA | VCAM1 | 0.478 |  |
| RHOA | SYK | 0.822 |  |
| RHOA | SPP1 | 0.938 |  |
| S100A9 | TLR2 | 0.671 |  |
| S100A9 | TYROBP | 0.621 |  |
| SAA1 | TLR2 | 0.572 |  |
| SAA1 | VCAM1 | 0.484 |  |
| SAA1 | TAC1 | 0.508 |  |
| SDC1 | VCAM1 | 0.424 |  |
| SDC1 | TNFRSF21 | 0.578 |  |
| SDC1 | TNFSF13B | 0.661 |  |
| SOCS3 | TNFRSF1A | 0.405 |  |
| SOCS3 | TLR2 | 0.597 |  |
| SOCS3 | STAT1 | 0.982 |  |
| SPP1 | TLR2 | 0.464 |  |
| SPP1 | VCAM1 | 0.63 |  |
| SPP1 | SYK | 0.92 |  |
| STAT1 | TNFRSF1A | 0.958 |  |
| STAT1 | TLR2 | 0.659 |  |
| STAT1 | VCAM1 | 0.458 |  |
| STAT1 | TNFRSF1B | 0.413 |  |
| STAT1 | TNFSF13B | 0.593 |  |
| STAT1 | SYK | 0.688 |  |
| SYK | TNFRSF1A | 0.72 |  |
| SYK | TLR2 | 0.793 |  |
| SYK | TYROBP | 0.999 |  |
| SYK | TNFSF13B | 0.567 |  |
| TLR2 | TNFRSF1A | 0.645 |  |
| TLR2 | TNFRSF1B | 0.481 |  |
| TLR2 | VCAM1 | 0.541 |  |
| TLR2 | TNFSF13B | 0.676 |  |
| TLR2 | TYROBP | 0.823 |  |
| TNFRSF12A | TNFRSF1A | 0.705 |  |
| TNFRSF12A | TNFRSF21 | 0.445 |  |
| TNFRSF12A | TNFRSF25 | 0.508 |  |
| TNFRSF12A | TNFSF13B | 0.745 |  |
| TNFRSF12A | TNFRSF1B | 0.77 |  |
| TNFRSF1A | TNFRSF25 | 0.418 |  |
| TNFRSF1A | TNFSF13B | 0.556 |  |
| TNFRSF1A | TNFRSF6B | 0.556 |  |
| TNFRSF1A | VCAM1 | 0.595 |  |
| TNFRSF1A | TNFRSF21 | 0.642 |  |
| TNFRSF1A | TNFRSF1B | 0.998 |  |
| TNFRSF1B | VCAM1 | 0.47 |  |
| TNFRSF1B | TNFSF13B | 0.763 |  |
| TNFRSF1B | TNFRSF25 | 0.478 |  |
| TNFRSF21 | TNFRSF25 | 0.909 |  |
| TNFRSF25 | TNFRSF6B | 0.634 |  |
| TNFSF13B | VCAM1 | 0.517 |  |

**Supplementary Table S5.** The correlation between three key DEGs and gene biomarkers of immune cells in GBM.

| Description | Gene markers | GBM | | | | | |
| --- | --- | --- | --- | --- | --- | --- | --- |
|  |  | C1R | | CCL2 | | TNFRSF1A | |
|  |  | Cor | P-value | Cor | P-value | Cor | P-value |
| CD8+ T cell | CD8A | 0.074 | 0.360 | 0.213 | ** | -0.047 | 0.565 |
|  | CD8B | 0.160 | * | 0.307 | *** | 0.016 | 0.843 |
| T cell (general) | CD3D | 0.300 | *** | 0.471 | *** | 0.130 | 0.109 |
|  | CD3E | 0.344 | *** | 0.406 | *** | 0.201 | * |
|  | CD2 | 0.363 | *** | 0.436 | *** | 0.203 | * |
| B cell | CD19 | 0.063 | 0.436 | 0.173 | * | 0.035 | 0.672 |
|  | CD79A | 0.054 | 0.508 | 0.133 | 0.102 | -0.078 | 0.399 |
| Monocyte | CD86 | 0.438 | *** | 0.509 | *** | 0.258 | ** |
|  | CD115(CSF1R) | 0.513 | *** | 0.457 | *** | 0.406 | *** |
| TAM | CCL2 | 0.586 | *** | 1 | *** | 0.478 | *** |
|  | CD68 | 0.498 | *** | 0.489 | *** | 0.337 | *** |
|  | IL10 | 0.401 | *** | 0.629 | *** | 0.24 | ** |
| M1 Macrophage | IRF5 | 0.348 | *** | 0.383 | *** | 0.233 | ** |
|  | COX2(PTGS2) | 0.46 | *** | 0.564 | *** | 0.362 | *** |
| M2 Macrophage | CD163 | 0.614 | *** | 0.576 | *** | 0.466 | *** |
|  | VSIG4 | 0.5 | *** | 0.519 | *** | 0.314 | *** |
|  | MS4A4A | 0.39 | *** | 0.4 | *** | 0.26 | *** |
| Neutrophils | CD11b (ITGAM) | 0.483 | *** | 0.601 | *** | 0.298 | *** |
|  | CCR7 | 0.251 | ** | 0.367 | *** | 0.148 | 0.068 |
| Natural killer cell | KIR2DL1 | -0.007 | 0.929 | 0.041 | 0.611 | -0.059 | 0.471 |
|  | KIR2DL3 | 0.011 | 0.890 | -0.078 | 0.341 | 0.003 | 0.968 |
|  | KIR2DL4 | 0.335 | *** | 0.232 | ** | 0.218 | ** |
|  | KIR2DS4 | 0.147 | 0.07 | 0.188 | * | 0.116 | 0.154 |
| Dendritic cell | HLA-DPB1 | 0.449 | *** | 0.448 | *** | 0.31 | *** |
|  | HLA-DQB1 | 0.341 | ** | 0.289 | *** | 0.251 | ** |
|  | HLA-DRA | 0.464 | *** | 0.539 | *** | 0.281 | *** |
|  | HLA-DPA1 | 0.401 | *** | 0.396 | *** | 0.239 | ** |
|  | BDCA-1(CD1C) | 0.156 | 0.053 | 0.408 | *** | 0.079 | 0.333 |
|  | BDCA-4(NRP1) | 0.543 | *** | 0.391 | *** | 0.501 | *** |
|  | CD11c (ITGAX) | 0.135 | 0.095 | 0.18 | * | 0.046 | 0.573 |
| Th1 | T-bet (TBX21) | -0.117 | 0.150 | -0.009 | 0.912 | -0.194 | * |
|  | STAT4 | 0.124 | 0.128 | 0.34 | *** | -0.049 | 0.53 |
|  | STAT1 | 0.233 | ** | 0.037 | 0.652 | 0.184 | * |
|  | TNF-a (TNF) | 0.038 | 0.636 | 0.314 | *** | -0.065 | 0.421 |
| Th2 | GATA3 | 0.162 | * | 0.148 | 0.068 | 0.213 | ** |
|  | STAT6 | 0.357 | *** | 0.359 | *** | 0.318 | *** |
|  | STAT5A | 0.46 | *** | 0.25 | ** | 0.46 | *** |
|  | IL13 | -0.16 | * | -0.13 | 0.095 | -0.23 | ** |
| Tfh | BCL6 | 0.258 | ** | 0.097 | 0.235 | 0.177 | * |
|  | IL21 | -0.02 | 0.802 | -0.02 | 0.802 | 0.009 | 0.908 |
| Th17 | STAT3 | 0.435 | *** | 0.213 | ** | 0.568 | *** |
| Treg | FOXP3 | 0.18 | * | 0.135 | 0.097 | 0.142 | 0.080 |
|  | STAT5B | -0.079 | 0.329 | -0.175 | * | 0.007 | 0.936 |
|  | TGFb (TGFB1) | 0.48 | *** | 0.331 | *** | 0.517 | *** |
| T cell exhaustion | PD-1 (PDCD1) | 0.162 | * | 0.15 | 0.065 | 0.024 | 0.765 |
|  | CTLA4 | 0.289 | *** | 0.343 | *** | 0.136 | 0.092 |
|  | LAG3 | −0.047 | 0.561 | -0.047 | 0.567 | -0.183 | * |
|  | TIM-3 (HAVCR2) | 0.351 | *** | 0.484 | *** | 0.215 | ** |
|  | GZMB | 0.353 | *** | 0.415 | *** | 0.187 | * |
| *p < 0.05, **p < 0.01, ***p < 0.001. | | | | | | | |
